# Intramolecular OH⋅⋅⋅Fluorine Hydrogen Bonding in Saturated, Acyclic Fluorohydrins: The γ-Fluoropropanol Motif

**DOI:** 10.1002/chem.201503253

**Published:** 2015-10-23

**Authors:** Bruno Linclau, Florent Peron, Elena Bogdan, Neil Wells, Zhong Wang, Guillaume Compain, Clement Q Fontenelle, Nicolas Galland, Jean-Yves LeQuestel, Jérôme Graton

**Affiliations:** a Department of Chemistry, University of Southampton Highfield, Southampton SO17 1BJ (UK), Fax: (+44) 23-8059-6805; b CEISAM UMR CNRS 6230, Faculté des Sciences et des Techniques Université de Nantes, 2, rue de la Houssinière - BP 92208, 44322 NANTES Cedex 3 (France), Fax: (+3) 2-51-12-54-02

**Keywords:** fluorination, intramolecular interactions, hydrogen bonds, NMR spectroscopy, quantum calculations

## Abstract

Fluorination is commonly exercised in compound property optimization. However, the influence of fluorination on hydrogen-bond (HB) properties of adjacent functional groups, as well as the HB-accepting capacity of fluorine itself, is still not completely understood. Although the formation of OH⋅⋅⋅F intramolecular HBs (IMHBs) has been established for conformationally restricted fluorohydrins, such interaction in flexible compounds remained questionable. Herein is demonstrated for the first time—and in contrast to earlier reports—the occurrence of OH⋅⋅⋅F IMHBs in acyclic saturated γ-fluorohydrins, even for the parent 3-fluoropropan-1-ol. The relative stereochemistry is shown to have a crucial influence on the corresponding ^h1^*J*_OH⋅⋅⋅F_ values, as illustrated by *syn*- and *anti-*4-fluoropentan-2-ol (6.6 and 1.9Hz). The magnitude of OH⋅⋅⋅F IMHBs and their strong dependence on the overall molecular conformational profile, fluorination motif, and alkyl substitution level, is rationalized by quantum chemical calculations. For a given alkyl chain, the “rule of shielding” applies to OH⋅⋅⋅F IMHB energies. Surprisingly, the predicted OH⋅⋅⋅F IMHB energies are only moderately weaker than these of the corresponding OH⋅⋅⋅OMe. These results provide new insights of the impact of fluorination of aliphatic alcohols, with attractive perspectives for rational drug design.

## Introduction

The question of whether organofluorines are effective hydrogen-bond (H-bond, HB) acceptors or not has been a heavily debated topic over the years.^1^ Key experimental evidence for intermolecular OH⋅⋅⋅F H-bonding includes IR^2^ and NMR^3^-based measurements between 4-fluorophenol and fluoroalkanes in solution. The conclusion of these studies is that organofluorines are able to act as HB acceptors, albeit with a weaker affinity than the usual oxygen- and nitrogen-based HB acceptors. Computational studies using various theoretical approaches (e.g. quantum theory of atoms in molecules, intermolecular perturbation theory) also support the occurrence of OH⋅⋅⋅F H-bonding.1d, 4

**Figure 1:**
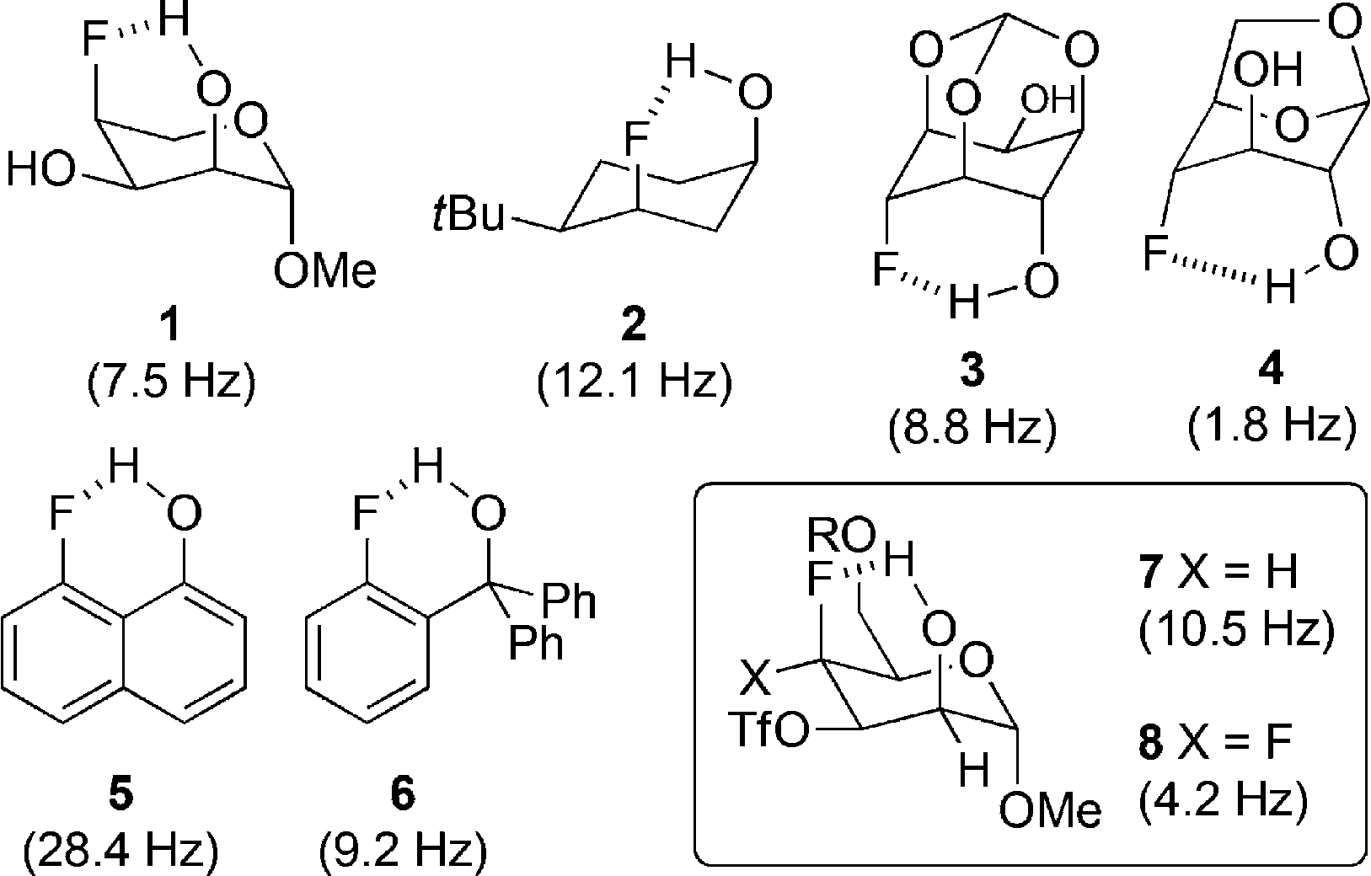
γ-Fluorohydrin (3-fluoroalkanol)-containing structures with their ^h1^*J*_OH⋅⋅⋅F_ coupling constants (CDCl_3_ or CD_2_Cl_2_).

In the case of intramolecular HBs (IMHBs), ambiguities about observed contacts being true HBs or forced consequences of the molecular structure are complicating factors.^5^ Only a modest number of examples have described IM OH⋅⋅⋅F interactions in the solution phase, typically with the observation of a “through-space” ^h1^*J*_OH⋅⋅⋅F_ coupling.^6^ In all of these cases, there is a significant degree of conformational restriction, promoting or fixing the proximity between the OH and F groups.^7^ Examples featuring γ-fluorohydrin motifs (Figure1) are restricted to monocyclic carbohydrates (e.g., **1**),^8^ conformationally restricted cyclohexanes, (e.g., **2**,^9^ **3**^10^), and bicyclic levoglucosan derivatives (e.g., **4**),^11^ all of which display 1,3-coaxial C–O/C–F bonds. The *peri*-substituted naphthalene derivative **5**^12^ and α,α-diphenyl-*o*-fluorobenzyl alcohol **6** also show a significant ^h1^*J*_OH⋅⋅⋅F_ coupling, whereas it was not detected for an *o*-fluorobenzyl alcohol motif.^13^ Where applicable, the ^3^*J*_OH–H_ value gives further positional information of the O-H proton relative to the fluorine atom. A nice recent illustration involves **7** and **8**, in which the reduced ^h1^*J*_OH⋅⋅⋅F_ and ^3^*J*_OH–H_ values for **8** indicate CF–F as a weaker HB acceptor than CH–F, and thus less able to compete with the ring oxygen.^14^

In contrast, to our knowledge, there are no examples of experimentally demonstrated OH⋅⋅⋅F IMHB as part of a flexible, fully saturated, acyclic 1,3-fluorohydrin in the solution state. Through gas-phase electron diffraction, the IMHB conformer of 3-fluoropropan-1-ol was identified as a secondary conformer, with rather low relative populations.^15^ Despite the presence of a bond critical point (BCP) demonstrated through atoms in molecules (AIM)^16^ analysis, 3-fluoropropan-1-ol was recently reported not to feature a ^h1^*J*_OH⋅⋅⋅F_ coupling, either in CD_2_Cl_2_ or in [D_12_]cyclohexane.^17^ It was attributed to the low calculated population of the IMHB conformation (11 % in CH_2_Cl_2_). In CDCl_3_, the 3-fluoropropan-1-ol ^h1^*J*_OH⋅⋅⋅F_ coupling was also not observed.^18^ Similarly, for 3-fluoro-1,2-propanediol, no ^h1^*J*_OH⋅⋅⋅F_ coupling could be observed by ^1^HNMR spectroscopy.^19^

**Figure 2:**
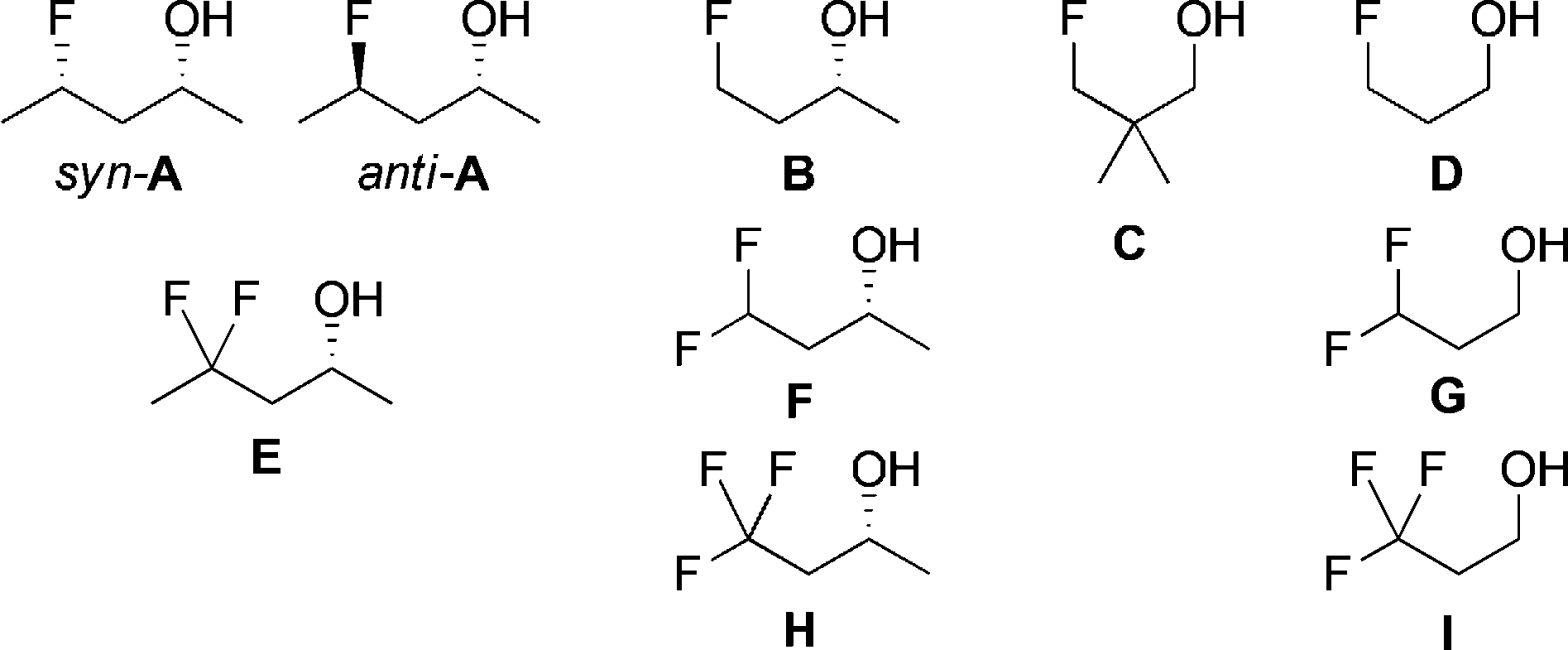
List of (racemic) γ-fluorohydrins under study.

Herein we describe an extensive combined NMR spectroscopic and computational analysis of a range of γ-fluorinated alcohols (Figure2). We provide evidence of IMHB between fluorine and alcohol groups as part of an acyclic chain in solution. For the first time, NMR ^h1^*J*_OH⋅⋅⋅F_ couplings have been experimentally evidenced and quantified for flexible fluorohydrins, even for 3-fluoro-, 3,3-difluoro- and 3,3,3-trifluoropropanol. A thorough analysis of the fluorohydrin conformational profile and the OH⋅⋅⋅F IMHB energies is provided, as well as an assessment of relative IMHB strengths with corresponding OH⋅⋅⋅OMe interactions.

## Results and Discussion

### Conformational analysis reveals very different conformer populations for the investigated fluorohydrins

The main minimum-energy conformers of the monofluorinated fluorohydrins are shown in Table1, where the various C-C-O-H rotamers have generally been grouped together for the sake of clarity. Dihedral angle definitions and detailed results are provided in the Supporting Information (SI1, TablesS1–S10). No significant differences were observed between MP2- and MPWB1K-calculated populations and therefore, only the MP2 results are given. Computed IMHB conformation properties are summarized in Table2.

**Table 1:** γ-Fluorohydrins investigated, with major populated conformations at 25 °C/−50 °C.

Fluorohydrin	Major conformations [a] in CHCl_3_[b]
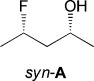	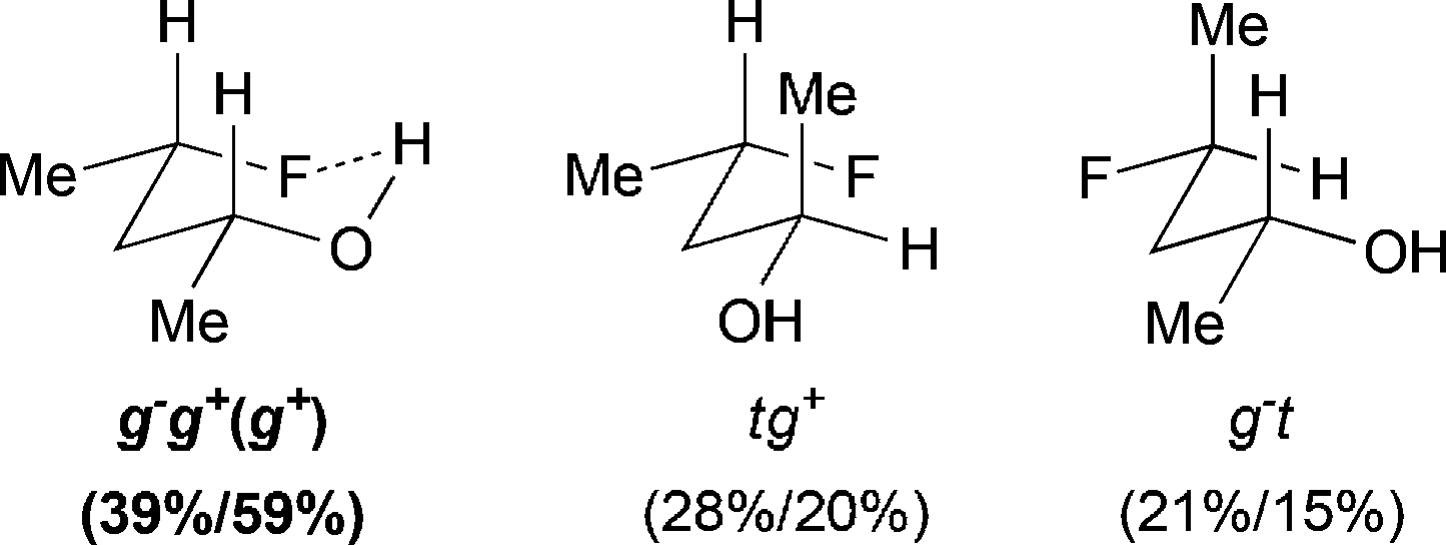
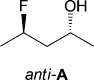	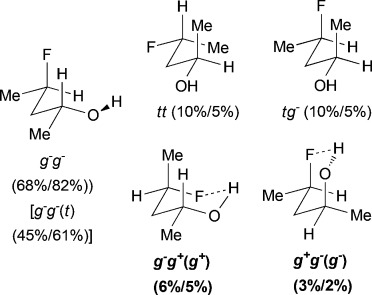
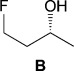	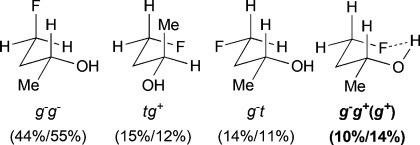
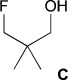	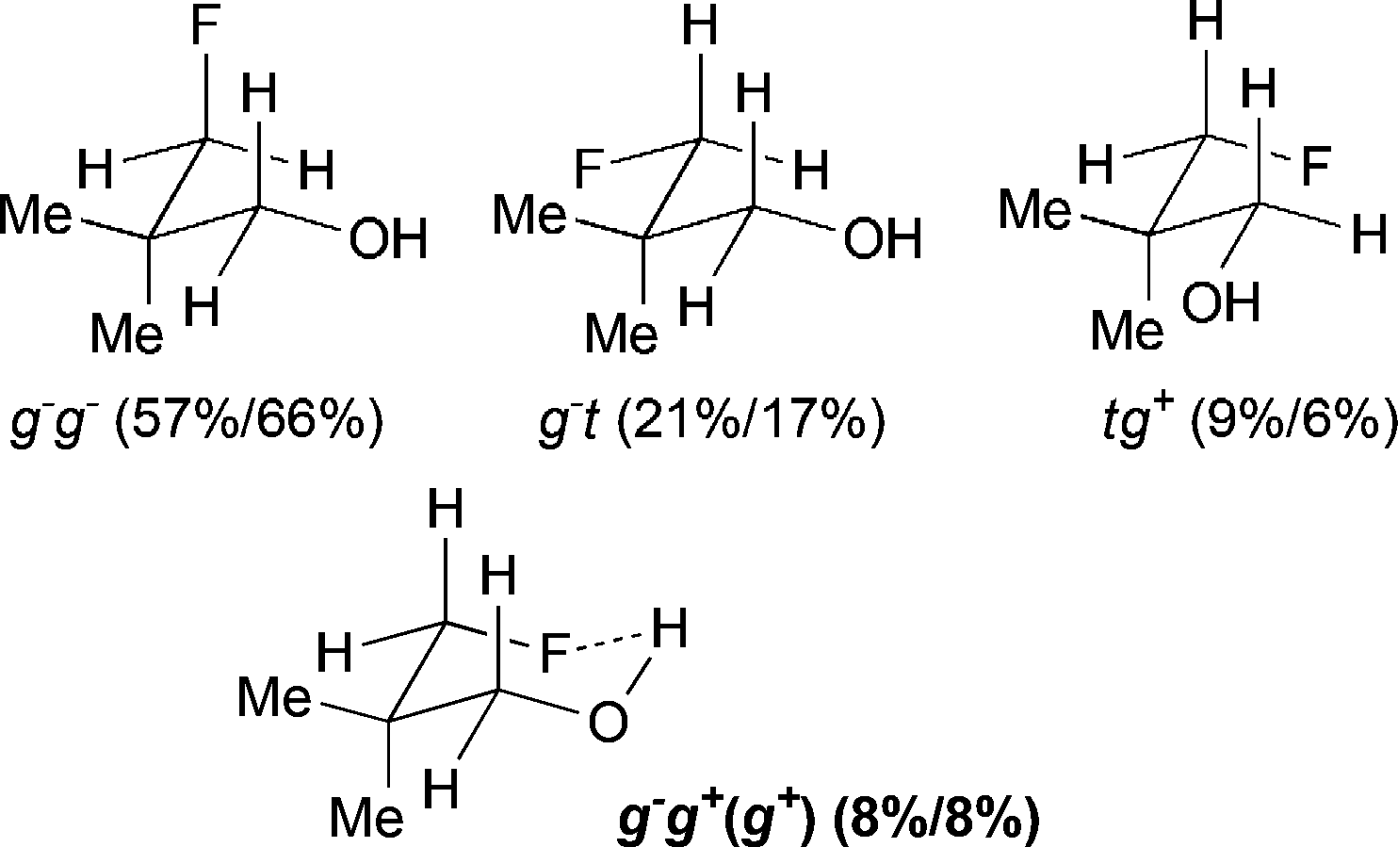
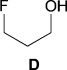	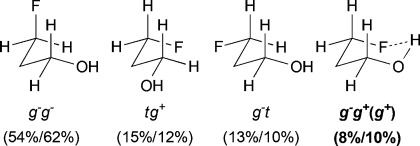

[a] Unless shown, all OH(*ψ*) rotamers are grouped together and figures represent combined populations;

[b] calculated at MP2/6-311++G(2d,p)//MPWB1 K/6-31+G(d,p)) level of theory.

**Table 2:** Computed features of the IM H-bonded conformations of all fluorohydrins.

Compound	Conformation	*p*_i_ [%]	*d*_OH⋅⋅⋅F_ [Å][a]	*ρ*_bcp_ [e bohr^−3^][b]	*E*_HB_ [kJ mol^−1^][c]	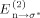 [kJ mol^−1^][d]
*syn*-**A**	*g*^−^ *g*^*+*^ (*g*^*+*^)	39	2.000	0.0206	24.4	25.1
*anti*-**A**	*g*^−^ *g*^*+*^ (*g*^*+*^)	6	2.008	0.0202	23.7	24.7
	g^*+*^ g^−^(g^*+*^)	3	2.056	0.0186	21.6	20.1
**B**	*g*^−^ *g*^*+*^ (*g*^*+*^)	10	2.037	0.0190	22.0	22.3
**C**	*g*^−^ *g*^*+*^ (*g*^*+*^)	8	2.065	0.0184	21.4	19.2
**D**	*g*^−^ *g*^*+*^ (*g*^*+*^)	8	2.074	0.0178	20.4	20.0
**E**	*g*^−^ *g*^*+*^ *g*^−^ (*g*^*+*^)	31	2.062	0.0185	21.7	17.8
	*g*^−^ *g*^*+*^ *t* (*g*^*+*^)	13	2.050	0.0187	21.7	19.4
**F**	*g*^−^ *g*^*+*^ *g*^−^ (*g*^*+*^)	6	2.133	0.0161	18.4	13.8
	*g*^−^ *g*^*+*^ *t* (*g*^*+*^)	5	2.090	0.0173	19.8	16.9
**G**	*g*^−^ *g*^*+*^ *t* (*g*^*+*^)	5	2.175	0.0148	16.6	9.0
	*g*^−^ *g*^*+*^ *g*^−^ (*g*^*+*^)	4	2.201	0.0142	16.1	10.5
**H**	*g*^−^ (*g*^*+*^)	47	2.172	0.0151	17.1	10.9
**I**	*g*^−^ (*g*^*+*^)	23	2.234	0.0136	15.4	8.3

[a] IMHB OH⋅⋅⋅F distance;

[b] electron density at the bond critical points from AIM analysis;

[c] HB energy at the MP2/6-311++G(2d,p) level;

[d] Interaction energies from the n_F_ fluorine lone pair to the σ^*^_OH_ antibonding orbital at the MPWB1 K/6-31+G(d,p) level.

For *syn*-4-fluoropentan-2-ol (*syn-***A**), the most stable conformer, *g*^−^ *g*^*+*^ (*g*^*+*^), showing an OH⋅⋅⋅F IMHB, is stabilized by 2.7kJ mol^−1^ towards the first secondary minimum at 25 °C, and represents 39 % of the whole population. The *d*_OH⋅⋅⋅F_ distance is 2.00Å, which is well below the sum of the van der Waals radii (2.57Å).^20^ The next stable conformations are *tg*^*+*^ (rotation around the C2–C3 bond) and *g*^−^*t* (rotation around the C3–C4 bond), their combined populations (49 %) exceeding that of *g*^−^ *g*^*+*^ (*g*^*+*^). The conformational profile of *anti*-**A** is very different: the dominance of the major conformers (*g*^−^ *g*^−^) is even more pronounced, but they do not exhibit any IMHB. Indeed, the conformations with the linear (zigzag) pentyl chain represent 68 % of the population, and the conformers featuring an IMHB are only slightly populated (6 % and 3 %). Hence, the extent of IM H-bonding significantly depends on relative fluorohydrin stereochemistry. Compared to *anti*-**A**, the *g*^−^ *g*^−^conformer remains the absolute minimum for 4-fluorobutan-2-ol (**B**), but is much less populated (44 %). In contrast, the amount represented by the *g*^−^ *g*^*+*^ (*g*^*+*^) IMHB conformer is raised, resulting in a 10 % population. The conformational profile for the 3-fluoropropan-1-ols **C** and **D** shows that the geminal dimethyl group does not have a large effect. In both cases, the *g*^−^ *g*^−^conformer appears consistently as the largest populated one, with the IMHB conformation representing only 8 % with a lengthening of the OH⋅⋅⋅F distances (2.07Å) with respect to compounds **A** (2.00Å) and **B** (2.04Å). For comparison, Cormanich etal.^17^ calculated a population of 11 % for 3-fluoropropan-1-ol (**D**) in CH_2_Cl_2_ at the MP2/aug-cc-pVDZ level, and Badawi and co-workers^18^ predicted 13 % (B3LYP/6-311+G(d,p)) and 10 % (MP2/6-311+G(d,p)) of such IMHB conformers in the gas phase.

The difluorinated alcohols **E** and **F**, both featuring diastereotopic fluorine atoms, display the same set of conformational minima, but the presence of the C5 methyl group significantly affects their relative populations (Table3). For **E**, *g*^−^ *g*^−^ *t* is the main conformer. This is the only instance of a significant stable conformation with a *syn* OH/CH_3_ relationship. Consistently, the *g*^−^ *g*^−^ *t* conformation is also the major conformer for **F** and is much more populated without the C5 methyl substituent. The IMHB conformers for **E** represent 44 % of the whole population, which is notably greater than the corresponding value calculated for **F**. For instance, the *g*^−^ *g*^*+*^ *g*^−^ (*g*^*+*^) conformation is 5 times more populated in **E**. In addition, the *syn*-fluorine of **E** appears significantly more chelated than the *anti*-fluorine, whereas the populations of the IM H-bonded conformations of the diastereotopic fluorines are very similar for **F**. For 3,3-difluoropropanol **G**, the *g*^−^ *g*^−^ *t* conformation remains the major conformer. Both its IM H-bonded conformers are populated in small amounts and the OH⋅⋅⋅F distances are significantly longer than in the previous compounds.

**Table 3:** γ,γ-Difluoro- and γ,γ,γ-trifluorohydrins investigated, with major populated conformations at 25 °C/−50 °C.

Fluorohydrin	Major conformations[a] in CHCl_3_[b]
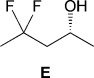	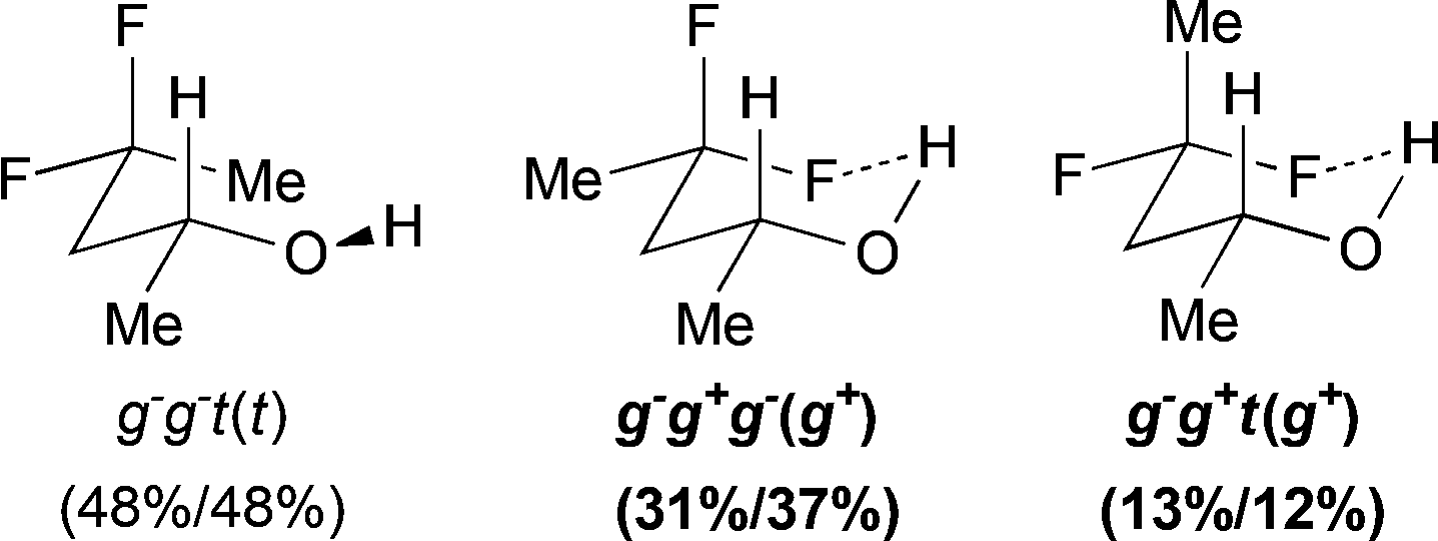
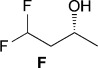	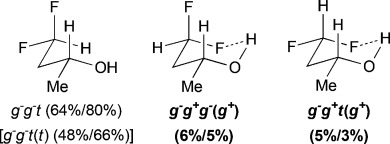
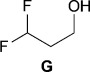	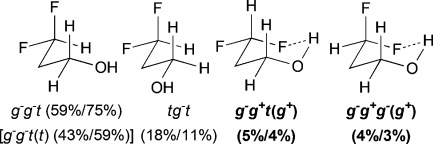
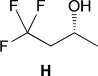	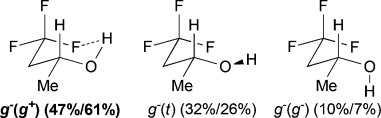
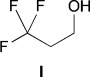	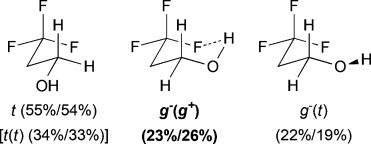

[a] Unless shown, all OH (*ψ*) rotamers are grouped together, and figures represent combined populations;

[b] calculated at MP2/6-311++G(2d,p)//MPWB1 K/6-31+G(d,p)) level of theory.

The three lowest energy minima of the trifluorinated **H** show a *g*^−^ 2-butanol chain, with the major conformer featuring an IMHB. Finally, in 3,3,3-trifluoropropanol **I**, the population of the IM H-bonded conformer is now significantly lower, and the OH⋅⋅⋅F distance observed (2.23Å) is the longest of the series. The main conformer now has a *trans* dihedral angle between the OH and the fluorinated group, a feature that is not observed with such a high population in any of the other motifs (except for the *t* *g*^−^ *t* conformation in **G**).

Within the whole substrate series, from CCl_4_ to CH_2_Cl_2_ (for full data, see the Supporting Information, SI1), it is worth noting that our analysis shows that an increase in the solvent polarity favors the non-chelated conformers.

### The change of temperature is predicted to strongly affect the fluorohydrin conformational profile

The influence of temperature on the conformational distribution was also studied (Table1, Table3). For the compounds with a significantly populated IMHB conformation (*syn-***A**, **E**, and **H**), a population increase is calculated upon temperature decrease. This is expected since lowering the temperature increases the probability to populate the lowest energy conformers. But, the change is more subtle for the IMHB conformers weakly populated at 25 °C. For **B**, **C**, **D**, and **I**, the population of the IMHB conformations slightly increases at −50 °C, whereas, for *anti*-**A**, **F**, and **G**, a population decrease is predicted. Interestingly, the two chelated conformers of 4,4-difluoropentan-2-ol **E** have opposite behaviors: the *g*^−^ *g*^*+*^ *g*^−^(*g*^*+*^) conformer, chelated with the *syn*-fluorine, is more populated at low temperature, whereas the *g*^−^ *g*^*+*^ *t* (*g*^*+*^) conformer, chelated with the *anti*-fluorine, is slightly less populated. This is consistent with the observations for *syn*-**A** and *anti*-**A**, respectively.

### NMR experiments reveal OH⋅⋅⋅F coupling constants for all investigated substrates

**Figure 3:**
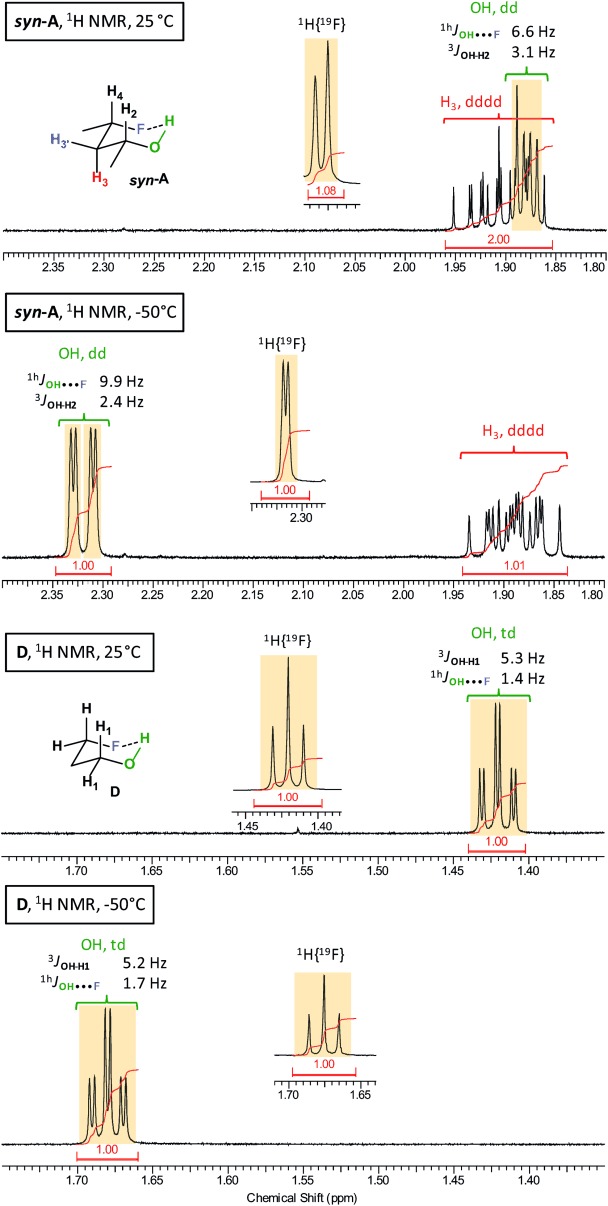
Details of the ^1^HNMR spectra of *syn*-A and D at 25 °C and −50 °C, showing the changes in *δ*(OH) and ^h1^*J*_OH⋅⋅⋅F_.

**Figure 4:**
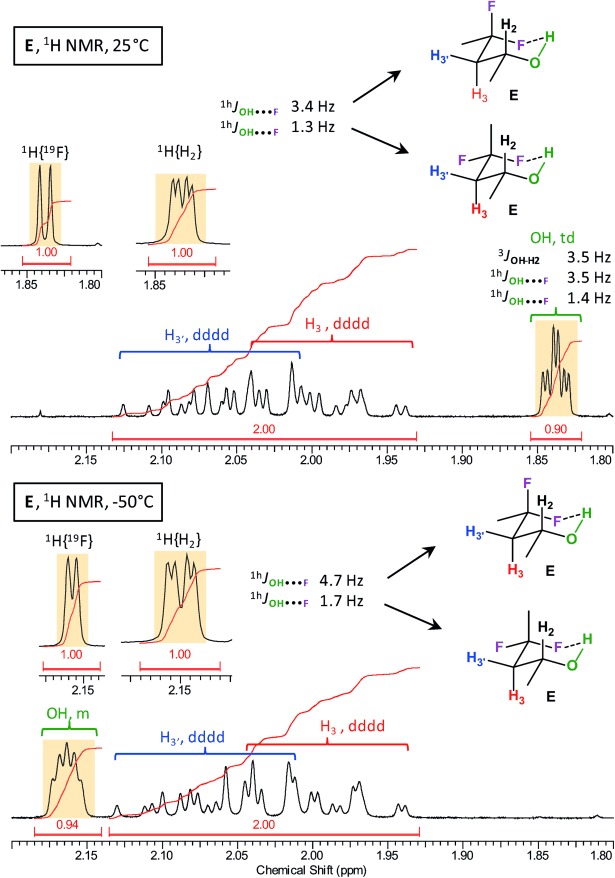
Details of the ^1^HNMR spectra of E at 25 °C and −50 °C, showing the changes in *δ*(OH) and ^h1^*J*_OH⋅⋅⋅F_.

NMR analysis focused on the multiplicity, ^h1^*J*_OH⋅⋅⋅F_ value, and chemical shift of the alcohol protons at 25 °C and −50 °C, taking into account that the observed values are averaged over the conformer populations. The results are given in Table4 and spectral details for *syn*-4-fluoropentan-2-ol (*syn*-**A**), 3-fluoropropan-1-ol (**D**), and 4,4-difluoropentan-2-ol (**E**) are shown in Figures3 and 4 (for detailed relevant spectra, see the Supporting Information, SI2). The results are compared with those obtained for the rigid 3-fluorocyclohexanol **2** (^h1^*J*_OH⋅⋅⋅F_=12.1Hz, 93 % IMHB conformer population in CHCl_3_).^9^

**Table 4:** Experimental and computed NMR data obtained in CDCl_3_.

Compound	^h1^*J*_OH⋅⋅⋅F_ @25 °C [Hz]			^h1^*J*_OH⋅⋅⋅F_ @−50 °C [Hz]			Δ*δ*[ppm][a]
	exp.[b]	calc.[c]		exp.[b]	calc.[c]		exp.
*syn*-**A**	6.6	−7.9		9.9	−11.9		0.48
*anti*-**A**	1.9	−1.5		1.8	−1.2		0.22
**B**	2.2	−1.8			−2.4
**C**	1.7	−1.2			−1.3
**D**	1.4	−1.2		1.7	−1.4		0.26
**E**	3.5	−4.9		4.7	−5.9		0.32
	1.4	−1.2		1.7	−1.0		
**F**	0.6	−0.2			−0.3		
	0.6	−0.4		−0.2
**G**	0.4(t)	−0.3			−0.2		
		−0.1		0.1
**H**	0.7(q)	−1.3			−1.7
**I**	0.3(q)	−0.1			−0.2

[a] Chemical shift difference upon cooling to −50 °C;

[b] sign not determined;

[c] calculated at the B97-2/pcJ-2 level of theory.

For *syn-***A**, OH appeared as a doublet of doublets, with ^1^H{^19^F} analysis proving a 6.6Hz coupling to fluorine (Figure3). Its magnitude is in the expected range, based on the calculated population of 39 %. The computed value (−7.9Hz), weighted according to the Boltzmann distribution of the various conformers in chloroform, agrees well with the experimental coupling. At −50 °C, the ^1^HNMR spectrum shows both a significant increase in ^h1^*J*_OH⋅⋅⋅F_ value (9.9Hz, Figure3) and a chemical shift change (Δ*δ*=0.48ppm), attributed to a population increase of the IMHB conformer. This is supported by theoretical calculations with the higher predicted population of such conformers (20 %), and the large increase of the computed weighted coupling constants (about 4Hz). The observed ^h1^*J*_OH⋅⋅⋅F_ value is much smaller, but clearly observable for *anti*-**A** (see the Supporting Information, SI2, 5.3.2), with a computed combined population of IMHB conformers of 9 %. No increase in coupling constant magnitude is then observed at −50 °C, in line with the computational predictions, and a weaker chemical shift change (Δ*δ*=0.22ppm) is measured. Similar coupling constants were found for **B** (2.2Hz) and **C** (1.7Hz), in reasonable agreement with the corresponding theoretical ^h1^*J*_OH⋅⋅⋅F_ values.

The 3-fluoropropan-1-ol (**D**) deserves special attention, given previous communications reporting that no ^h1^*J*_OH⋅⋅⋅F_ value could be observed for this compound in CD_2_Cl_2_, CDCl_3_, or [D_12_]cyclohexane.^17^, ^18^ With an exceptionally resolved NMR spectrum (Figure3), we have succeeded to detect an OH signal clearly appearing as a triplet of doublets in CDCl_3_, and the ^1^H{^19^F} analysis proved that there is a 1.4Hz coupling to fluorine. This coupling constant remained essentially the same upon cooling to −50 °C (1.7Hz). These observations are supported by the weighted theoretical ^h1^*J*_OH⋅⋅⋅F_ values of −1.2 and −1.4Hz, computed at 25 and −50 °C, respectively. Furthermore, the ^1^HNMR spectrum of **D** in the more polar CD_2_Cl_2_ also shows the presence of an IMHB at 25 °C (see the Supporting Information, SI2, 5.6.8), with a decreased ^h1^*J*_OH⋅⋅⋅F_ value (1.0Hz). The computed value is similarly slightly weaker in CD_2_Cl_2_ (1.1Hz) owing to the slightly less populated IMHB conformer. It is moreover in good agreement with the weighted value computed by Cormanich et al (1.68Hz).^17^

For the di- and trifluorinated derivatives, multiple couplings of the alcohol hydrogen atom with the fluorine atoms were expected. The conformational analysis of 4,4-difluoropentan-2-ol (**E**) revealed this compound as an interesting case with the prediction of a significant difference in ^h1^*J*_OH⋅⋅⋅F_ values (−4.9 and −1.2Hz) for its two diastereotopic fluorine atoms. We were delighted to be able to observe such a distinction experimentally (Figure4): a triplet of doublets was seen for the OH group, with ^h1^*J*_OH⋅⋅⋅F_ values of 3.5 and 1.4Hz. In addition, the predicted increase of the former coupling at −50 °C (+1.0Hz) was also observed experimentally (+1.2Hz, with ^h1^*J*_OH⋅⋅⋅F_ value of 4.7Hz). For **F**, an apparent triplet was observed with a very small coupling constant (0.6Hz). Equally, for **G** a triplet was observed with a likewise low *J*value. Finally, it was surprising to observe a doublet of quartets for the OH groups in both trifluorinated fluorohydrins **H** and **I** (see the Supporting Information, SI2, 5.10–11), even if the magnitude of the coupling constants is very small (0.3–0.7Hz).

### ^1^HNMR coupling constant analysis confirms the chair-like conformation of *syn*-A

**Figure 5:**
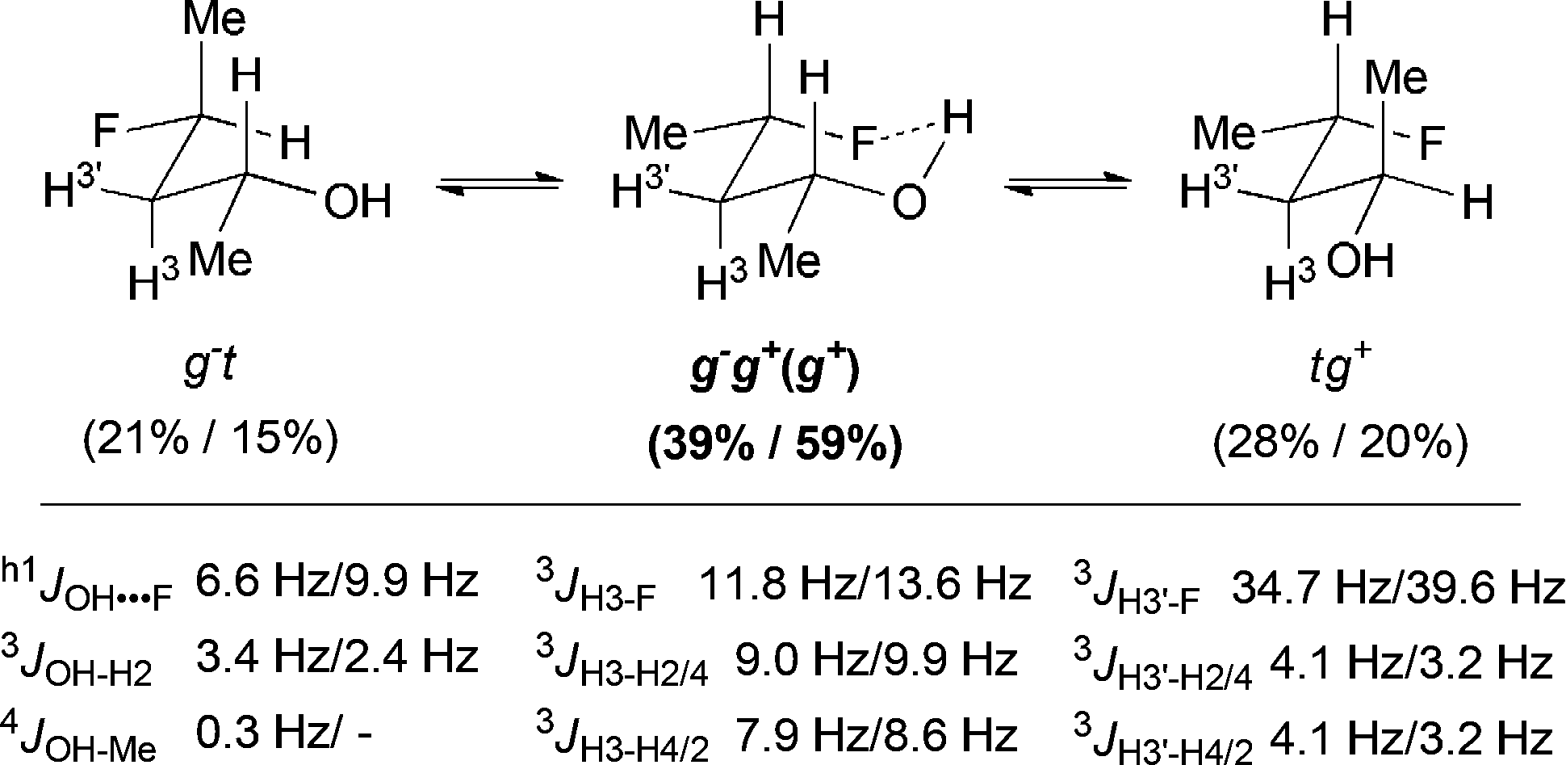
Conformer populations, with relevant coupling constants of *syn*-A at 25 °C and −50 °C

The chair-like IMHB conformation for *syn*-**A** was confirmed by further NMR spectroscopic analysis (Figure5). An “axial” H^3^ and “equatorial” H^3′^ can clearly be identified; the former with two large ax–ax coupling constants (9.0, 7.9Hz), and the latter with two smaller eq–ax coupling constants (4.1, 4.1Hz), to H^2^ and H^4^. Upon cooling to −50 °C, these values increase or decrease in accordance with the population increase in the IMHB conformation compared to *t* *g*^*+*^ and *g*^−^ *t*. The increase in ^3^*J*_H3′–F_ of approximately 5Hz upon cooling is interpreted similarly, and the ^3^*J*_OH–H2_ coupling value corresponds to a *gauche* dihedral angle. Its calculated value is in remarkable coherence with experiment (both 3.4Hz).

### AIM analyses provide evidence for IMHB in all cases and allow quantification of their energies

For all the compounds under study, BCPs between the H(O) and F atoms were systematically found through AIM analyses on their IMHB conformations (Table2), validating the presence of IMHB interactions. Beyond the difficulties to estimate the strength of intramolecular interactions, less properly defined than intermolecular interactions through the super molecule approach, the estimation of the HB energy (*E*_HB_), based on the potential energy density *V*_b_ at the BCP, is however informative, with the caveat that it overestimates the actual HB strength.^21^ For this reason, the following comparison will focus on the relative trends calculated rather than on the absolute values. The highest value of the series is calculated for *syn*-**A** (24.4kJ mol^−1^), which corresponds to the structure exhibiting the shortest IMHB (2.00Å). Conversely, 3,3,3-trifluoropropan-1-ol (**I**) shows the weakest HB energy (15.4kJ mol^−1^) and the longest IMHB (2.23Å), these two energetic and structural parameters being strongly correlated (*r*^2^=0.983). It is interesting to note that the IMHB strengths are of the same order of magnitude in *anti*-**A** and *syn*-**A**, despite an IMHB conformation 6times less populated for the former. In the same vein, the OH⋅⋅⋅F distances (2.06 and 2.05Å) and the HB energies (21.6 and 21.7kJ mol^−1^) are very similar for the *g*^−^ *g*^*+*^ *g*^−^ (*g*^*+*^) and *g*^−^ *g*^*+*^ *t* (*g*^*+*^) conformers in **E**, whereas their relative populations differ significantly (31 and 13 %, respectively). It is worth noting that the electron density values at the BCP, also commonly used as HB strength descriptor, are strongly correlated to *E*_HB_ (*r*^2^=0.998).

**Figure 6:**
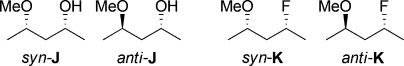
Reference compounds included for comparison with the fluorohydrins.

The effective strength of the OH⋅⋅⋅F IMHB deserves comparison with conventional OH⋅⋅⋅O IMHB energies. In β-diketones, strong OH⋅⋅⋅O IMHBs involve a conjugated system between the carbonyl and the hydroxy groups with a calculated *E*_HB_ of around 100kJ mol^−1^.^22^ The OH⋅⋅⋅F HB strengths found in the current work are indeed 4–6times weaker. In contrast, comparison of *syn*-**A** and *anti*-**A** with the corresponding 4-methoxypentan-2-ol diastereomers *syn*-**J** and *anti*-**J** (Figure6 and TableS11 in the Supporting Information, SI1) showed a different picture. The calculated *E*_HB_ for both diastereomers of **J** are 30.7 and 29.7kJ mol^−1^, which is only 25 % higher than the *E*_HB_ values of *syn*-**A** and *anti*-**A** (24.4 and 23.7kJ mol^−1^). These relative energies correlate with the intramolecular distances, which are shorter for OH⋅⋅⋅OMe (1.92Å for both **J** diastereomers) than for OH⋅⋅⋅F (2.00Å). The impact of the OH⋅⋅⋅OMe IMHB on the populations of the corresponding structures is consistent with the increased *E*_HB_ values: the IMHB structures of *anti*-**J** represent almost 60 % of the total population conformers (9 % for *anti*-**A**), whereas those of *syn*-**J** are as high as 95 % (39 % for *syn*-**A**).

Conversely, comparisons with other weak interactions demonstrate the stronger fluorohydrin OH⋅⋅⋅F interactions in compounds **A**–**I**. The CH⋅⋅⋅O IMHBs of adenosine derivatives were computed to range from 7 to 16kJ mol^−1^ using *E*_HB_ descriptor.^23^ Similarly, in short intermolecular CH⋅⋅⋅F H-bonds identified in crystalline organic fluorine structures *E*_HB_ reached 12kJ mol^−1^ and the complexation energies of small organofluorine molecules were calculated at high levels of theory to be lower than 10kJ mol^−1^.^4^, ^24^

### Further analyses give insight into the various stabilizing and destabilizing interactions

**Figure 7:**
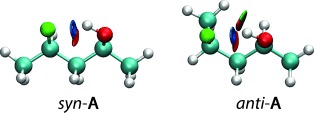
NCI isosurface plots of *g*^−^ *g*^+^ (*g*^*+*^) conformers of *syn*-A and *anti*-A compounds drawn with a reduced density gradient (RDG) value of 0.6 and the blue-green-red values ranging from −0.02 to 0.01a.u.

Both noncovalent interaction (NCI) and natural bond orbital (NBO) analyses corroborate the AIM results for the chelated conformers. With NCI, an attractive contribution relative to the interaction between the C–F and C–O(H) groups systematically outweighs the repulsive counterpart associated with the parallel orientation of the two corresponding dipoles (see the Supporting Information, TablesS14–S22), hence corresponding to an effective IMHB, as illustrated by the blue isosurfaces shown in Figure7. The NBO interaction energies 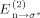 from the n_F_ fluorine lone pairs to the σ*_OH_ antibonding orbital, describing the charge transfer component of the interaction, range from 25 to 8kJ mol^−1^ (Table2), with variations in agreement with those observed with the *E*_HB_ descriptor.

In addition to the OH⋅⋅⋅F IMHB, other secondary interactions contribute to the stabilization or destabilization of the various conformers. For example, the *g*^−^ *g*^−^ conformations of *anti*-**A**, representing almost 70 % of the whole population, show simultaneously a CH⋅⋅⋅O and a CH⋅⋅⋅F 5-membered interaction, yielding conformers more stabilized than the *g*^−^ *g*^*+*^ (*g*^*+*^) IM H-bonded conformer. Conversely, these secondary interactions cannot operate simultaneously in *syn*-**A**, with only CH⋅⋅⋅F in the *t* *g*^*+*^ or CH⋅⋅⋅O in the *g*^−^ *t* conformers, resulting in the IMHB conformer becoming the global energetic minimum. These features, which are not detectable through AIM analysis, are corroborated by the NBO calculations. Indeed, weak interaction energies, from 1.0 to 2.5kJ mol^−1^, are found for *syn*-**A** and *anti*-**A** between either the n_F_ fluorine or n_O_ oxygen lone pairs and the σ*_CH_ antibonding orbitals. It may rationalize why, despite similar IMHB characteristics (d_OH⋅⋅⋅F_ and *E*_HB_), the corresponding relative populations differ significantly in *syn*-**A** and *anti*-**A**. Moreover, the H-bonded conformers of *anti*-**A** undergo an additional CH⋅⋅⋅CH_3_ destabilizing interaction (a classic *gauche*–butane interaction), whereas such repulsive contributions rather occur in *t* *g*^*+*^ and *g*^−^ *t* conformers of *syn*-**A**. The higher population of non-chelated conformers for *anti*-**J** compared to *syn-***J** is similarly explained. With 4,4-difluoropentan-2-ol **E**, the higher stabilization of the *g*^−^ *g*^*+*^ *g*^−^ (*g*^*+*^) vs. *g*^−^ *g*^*+*^ *t* (*g*^*+*^) conformers is explained by the attractive CH⋅⋅⋅F contribution in the former replaced by a repulsive CH⋅⋅⋅CH_3_ interaction in the latter.

Considering the whole data set of conformations, it appears that the structures that are more stable than the IMHB conformers contain systematically at least two CH⋅⋅⋅X stabilizing interactions. This is consistent with the conformational profiles of *syn*- and *anti-*2-fluoro-4-methoxypentanes (*syn*-**K** and *anti*-**K**; see the Supporting Information, TableS12, SI1). The linear (zigzag) pentane conformation is clearly preferred for *anti*-**K** (7kJ mol^−1^, 87 %), as found for *anti*-**A** for which the three first linear conformers represented 68 % of the whole population. These *anti*-**K** conformations have short CH_3_⋅⋅⋅F or CH_3_⋅⋅⋅O intramolecular distances (2.34–2.43Å). With *syn*-**K**, the linear conformation represents only 2 % of the whole population due to the significant repulsion between the fluorine and methoxy groups, whereas it reaches 40 % of the population in *syn*-**A**, due to its ability to establish an IMHB. This analysis is also consistent with the conformational profile of other 2,4-disubstituted pentanes, as reported by Hoffmann etal.^25^

### Comparison of IMHB between the monofluorinated and di-/trifluorinated derivatives

Dalvit and Vulpetti^3^, 26a demonstrated the impact of the fluorine environment on its HB-accepting capacity in the context of intermolecular OH⋅⋅⋅F interactions, with CHF>CF_2_>CF_3_ as a general HB-accepting trend. This ranking is consistent with the evolution of the electron density on fluorine, as displayed by their respective fluorine chemical shift values [*δ*_F_(CF)<*δ*_F_(CF_2_)<*δ*_F_(CF_3_)] and referred to as the “rule of shielding”. Furthermore, Bernet and Gouverneur^14^ demonstrated that the OH⋅⋅⋅F IMHB is weaker when a CF_2_ motif is involved than it is with a CHF motif. This effect was also invoked by Suhm and co-workers to explain the hydrogen-bonding properties of progressively fluorinated ethanol molecules with water.^27^

This trend is consistent with the computed *E*_HB_ energies for **A**–**I**, which range from 24.4 to 20.4kJ mol^−1^ for the monofluorinated compounds, from 21.7 to 16.1kJ mol^−1^ for the difluorinated compounds, and from 17.1 to 15.4kJ mol^−1^ for the trifluorinated compounds (Table2), provided the alkyl chain is strictly conserved. Indeed, variations as subtle as methylation can lead to an overlap of these three energetic ranges: the difluorinated **E** has larger *E*_HB_ values than the monofluorinated **C** and **D**, and the same is true when comparing the trifluorinated **H** to the difluorinated **G**. Similar conclusions can be drawn by considering the NBO interaction energies 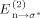. These behaviors are not in line with the “rule of shielding” proposed by Dalvit and Vulpetti 26a (e.g., **E**: *δ*_F_=−90.3, −89.5ppm with *E*_HB_=−21.7kJ mol^−1^; vs. **D**: *δ*_F_=−221.8ppm with *E*_HB_=−20.4kJ mol^−1^).

These observations are confirmed by the calculation of relevant electrostatic potential descriptors. The *V*_min_ descriptor is related to HB acceptor ability, and was thus shown by Dalvit and Vulpetti to reflect the reduced HB-accepting capacity from mono- to trifluoro derivatives.^3^ On the other hand, the *V*_α_(r) descriptor is related to HB donor ability.^28^ As it is not possible to calculate these descriptors for the chelated conformations due to the perturbation of the IMHB, the descriptors calculated for the *t* *t* (*t*) conformer of compounds *anti*-**A**, **B**, and **D**–**I** were used for illustration, as shown in Table5.

**Table 5:** Electrostatic potential values [kJ mol^−1^] calculated for the *t* *t* (*t*) conformers on the OH [*V*_α_(*r*)] and F [*V*_min_] sites at the MPWB1 K/6-31+G(d,p) level of theory.[a]

	Monofluoro[b]			Difluoro[c,d]			Trifluoro[d,e]	
	*V*_α_(*r*)	*V*_min_		*V*_α_(r)	*V*_min_		*V*_α_(r)	*V*_min_
pentanol	860.4	−143.0		868.5	−120.0			
butanol	863.8	−135.5		872.2	−106.7		882.2	−68.5
propanol	870.4	−132.7		880.9	−104.5		891.4	−66.0

[a] The higher *V*_α_(*r*), the better the HB-donating capacity; the lower the *V*_min_, the better the F HB-accepting capacity;

[b] *anti*-**A9**, **B17**, and **C10**/**D10**;

[c] **E7**, **F5**, **G3**;

[d] *V*_min_ values given for the *trans* fluorine in the polyfluorinated derivatives;

[e] **H6**, **I1**.

It can be clearly seen that, although additional fluorination indeed reduces the fluorine HB-accepting capacity, there is a concomitant, but weaker, increase in OH HB donating capacity. Furthermore, it is evident from Table5 that both fluorine and OH HB properties depend on the alkane chain length, in a significant way. As a consequence, the OH⋅⋅⋅F IMHB strength encountered in the current series significantly depends on interlinked competing electronic factors, leading to small energy changes, and resulting in an overlapping energy range between mono- and difluorinated fluorohydrins, and between di- and trifluorinated fluorohydrins depending on the alkyl chain length. Interestingly, in the difluorinated structures, compared to the *V*_min_ value of the *trans*-fluorine atom shown in Table5, the *V*_min_ values of the second fluorine atom are significantly decreased (Δ*V*≈20kJ mol^−1^; not shown) suggesting a much weaker HB-accepting ability. In contrast, for the trifluorinated alcohols, the difference between the H-bond basicity of the *trans* fluorine and the two other fluorine atoms found is much less pronounced (Δ*V*≈3kJ mol^−1^; not shown).

## Conclusion

This work introduces compelling experimental evidence of the occurrence of OH⋅⋅⋅F intramolecular hydrogen bonding in fully saturated acyclic compounds containing a 1,3-fluorohydrin motif. The presence of ^h1^*J*_OH⋅⋅⋅F_ coupling constants, sometimes of considerable magnitude (up to 6.6Hz at 25 °C; 9.9Hz at −50 °C), was demonstrated. The experimental NMR data were fully consistent with DFT calculations. The comparison between the 4-fluoropentan-2-ol and 4-methoxypentan-2-ol systems highlights the significance of the OH⋅⋅⋅F interaction, indicating that the IMHB energy of the former reaches almost 80 % of the latter. Following the “rule of shielding” reported by Dalvit and Vulpetti, 26a as well as the findings of Bernet and Gouverneur,^14^ di- and trifluorination leads to a reduction in H-bond strength. However, the effect was found to be moderate and could be easily overcompensated by other electronic effects, such as the concomitant increase in alcohol HB donating capacity. Finally, the rule appears to be limited to a given alkyl chain. Significantly, the absence of conformational rigidity removes any ambiguity about the OH⋅⋅⋅F interaction being the result of a forced contact, allowing for an unbiased study of the multitude of often opposing effects that determine the extent of IMHB. Fluorination of alkanols at the γ-position resulted in a complex conformational profile, with the influence of the fluorination operating simultaneously through OH⋅⋅⋅F IMHB, attractive C–H⋅⋅⋅F interactions, and steric considerations such as repulsive contributions of C–Me with C–H and C–F, or C–O/C–F dipole-mediated interactions. As an illustration, the very low population (2 %) of the linear (zigzag) alkyl chain in *syn*-4-fluoro-2-methoxypentane is raised to 39 % by introducing OH⋅⋅⋅F IMHB, as in *syn*-4-fluoropentan-2-ol, and to 95 % for *syn*-4-methoxy-pentan-2-ol.

This work thus provides significant new insights on OH⋅⋅⋅F IM H-bonding in fluoroalkanols, and shows that these are much more important than previously assumed. The advances reported herein will not only contribute to a better understanding of the impact of aliphatic fluorination, currently increasingly exercised in property optimization of organic materials and bioactive compounds, but will also be of interest to the many areas where hydrogen bonding is of importance, for example rational drug design, where the existence of OH⋅⋅⋅F IMHB in non-aqueous environments can be exploited: the formation of IMHB has a pronounced effect on important ligand molecular properties, including membrane permeability.26, 29 Further investigations about the influence of the fluorination on the intermolecular hydrogen-bonding properties of acyclic 1,3-fluorohydrins are in progress.

## Experimental Section

### Computational details

All DFT calculations were performed by using version D.01 of the Gaussian 09 program.^30^ The conformational landscape of the fluorohydrins was exhaustively investigated at the MPWB1 K/6–31+G(d,p) level in CCl_4_ medium through, in a first step, simultaneous rigid scans of their two *ϕ*(C-C-C-X) dihedral angles from 0° to 360° in steps of 30°, by considering in addition three different orientations of the *ϕ*(H-O-C-H) dihedral angle (180°, 60°, and −60°). Solvent effects were systematically introduced by means of the polarizable continuum model (PCM) within the integral equation formalism. The geometry optimization and the frequency calculation of the various energetic minima were then carried out at the same level of theory. Eventually, single-point calculations at the MP2/6–311++G(2d,p) level were carried out in CCl_4_, CHCl_3_, and CH_2_Cl_2_ solvents. The electronic energies were then converted into Gibbs free energies by using standard thermodynamic corrections from the MPWB1 K/6–31+G(d,p) frequency calculations. The high flexibility of the investigated compounds generates significant amounts of secondary conformers. In the Tables S1–S12 (see the Supporting Information, SI1), the relative energies and Boltzmann populations are given for all conformations within 12kJ mol^−1^ from the global energy minimum, for each fluorohydrin, together with a detailed explanation of the used nomenclature.

The spin–spin coupling constants (*J*) were estimated from the previous optimized geometries by using the gauge-invariant atomic orbital (GIAO) method. The hybrid B97–2 functional^31^ and the pcJ-2 basis set, specifically designed for the calculation of these NMR parameters,^23^ were used. Again, solvent (CHCl_3_) effects were introduced through the PCM model. Calculated *J* values were averaged over all conformers according to their relative populations in CHCl_3_ at 298K and 223K.

To gain more insights on the IMHB interactions at work in relevant conformers of the various compounds, AIM topological analyses^16^, ^32^ of the PCM/MP2/6–311++G(2d,p) wave functions were carried out using the AIM2000 program.^33^ Electron density values, *ρ*_bcp_, are computed at the BCP, and the corresponding HB energies *E*_HB_ are estimated from the potential energy densities *V*_b_.^34^ In addition, NCI^35^ analyses of the same wavefunctions were also performed by using the NCIPLOT 3.0 program,^36^ to detect additional secondary interactions and to estimate their contributions. Finally, the NBO^37^ method was applied at the PCM/MPWB1 K/6–31+G(d,p) level to provide a complementary description of the IMHB. Its strength is related to the charge transfer between the n_F_ fluorine lone pairs and the σ* HB-donor antibonding orbitals by using the corresponding 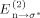 interaction energies computed from the second-order perturbation theory.

### NMR spectroscopy

For all substrates, the ^1^H, ^19^F, and ^1^H{^19^F} NMR spectra were collected after rigorous drying of the solutions (9–15mm) with activated molecular sieves, which is required to suppress water–solute interactions that would interfere with the OH⋅⋅⋅F IMHB. A detailed procedure is provided in the Supporting Information (SI2).

### Fluorohydrin synthesis

The synthesis of the novel compounds *syn*- and *anti*-**A**, **B, C**, **E**, **F**, and **G** is detailed in the Supporting Information (SI3 and SI4). The other compounds were commercially available and used without purification.

## Supporting Information

As a service to our authors and readers, this journal provides supporting information supplied by the Such materials are peer reviewed and may be re-organized for online delivery authors. but are not copy-edited or typeset. Technical support issues arising from supporting information (other than missing files) should be addressed to the authors.

**miscellaneous_information**
