# Supplementary material for: Intramolecular OH⋅⋅⋅Fluorine Hydrogen Bonding in Saturated, Acyclic Fluorohydrins: The γ-Fluoropropanol Motif
Source: Chemistry. 2015 Oct 23;21(49):17808–16. doi: 10.1002/chem.201503253 (PMC4676915; doi:10.1002/chem.201503253)

CHEMISTRY
A European Journal

Supporting Information

Intramolecular OH···Fluorine Hydrogen Bonding in Saturated, Acyclic Fluorohydrins: The γ-Fluoropropanol Motif

Bruno Linclau,*[a] Florent Peron,[a] Elena Bogdan,[b] Neil Wells,[a] Zhong Wang,[a] Guillaume Compain,[a] Clement Q. Fontenelle,[a] Nicolas Galland,[b] Jean-Yves Le Questel,[b] and Jérôme Graton*[b]

chem_201503253_sm_miscellaneous_information.pdf

SUPPORTING INFORMATION 1
Computational studies

Table of Contents

1 Conformational descriptors....4

2 Conformational analysis....5

2.1 Energetic distribution of analyzed compounds....5

2.1.1 Table S1. Free energy differences (ΔG, kJ mol^{-1}) and conformational distribution (p_i, %) of *syn*-4-fluoropentan-2-ol (*syn*-A)....5

2.1.2 Table S2. Free energy differences (ΔG, kJ mol^{-1}) and conformational distribution (p_i, %) of *anti*-4-fluoropentan-2-ol (*anti*-A)....6

2.1.3 Table S3. Free energy differences (ΔG, kJ mol^{-1}) and conformational distribution (p_i, %) of 4-fluorobutan-2-ol (B)....7

2.1.4 Table S4. Free energy differences (ΔG, kJ mol^{-1}) and conformational distribution (p_i, %) of 2,2-dimethyl-3-fluoropropan-1-ol (C) 8

2.1.5 Table S5. Free energy differences (ΔG, kJ mol^{-1}) and conformational distribution (p_i, %) of 3-fluoropropan-1-ol (D)....9

2.1.6 Table S6. Free energy differences (ΔG, kJ mol^{-1}) and conformational distribution (p_i, %) of 4,4-difluoropentan-2-ol (E)....10

2.1.7 Table S7. Free energy differences (ΔG, kJ mol^{-1}) and conformational distribution (p_i, %) of 4,4-difluorobutan-2-ol (F)....11

2.1.8 Table S8. Free energy differences (ΔG, kJ mol^{-1}) and conformational distribution (p_i, %) of 3,3-difluoropropan-1-ol (G)....12

2.1.9 Table S9. Free energy differences (ΔG, kJ mol^{-1}) and conformational distribution (p_i, %) of 4,4,4-trifluorobutan-2-ol (H)....13

2.1.10 Table S10. Free energy differences (ΔG, kJ mol^{-1}) and conformational distribution (p_i, %) of 3,3,3-trifluoropropan-1-ol (I)....13

2.1.11 Table S11. Free energy differences (ΔG, kJ mol^{-1}) and conformational distribution (p_i, %) of *syn*-4-methoxypentan-2-ol (*syn*-J) and *anti*-4-methoxypentan-2-ol (*anti*-J)....14

2.1.12 Table S12. Free energy differences (ΔG, kJ mol^{-1}) and conformational distribution (p_i, %) of *syn*-2-fluoro-4-methoxypentane (*syn*-K) and *anti*-2-fluoro-4-methoxypentane (*anti*-K)....14

2.2 Depiction of the optimized structures at the IEFPCM-MPWB1K/6-31+G(d,p) level of theory....15

2.2.1 *syn*-4-fluoropentan-2-ol (*syn*-A) 15
2.2.2 *anti*-4-fluoropentan-2-ol (*anti*-A) 16
2.2.3 4-fluorobutan-2-ol (B) 17
2.2.4 2,2-dimethyl-3-fluoropropan-1-ol (C) 18
2.2.5 3-fluoropropan-1-ol (D) 18
2.2.6 4,4-difluoropentan-2-ol (E) 19
2.2.7 3,3-difluorobutan-2-ol (F) 19
2.2.8 3,3-difluoropropan-1-ol (G) 20
2.2.9 4,4,4-trifluorobutan-2-ol (H) 20
2.2.10 3,3,3-trifluoropropan-1-ol (I) 20
2.2.11 *syn*-4-methoxypentan-2-ol (*syn*-J) 21
2.2.12 *anti*-4-methoxypentan-2-ol (*anti*-J) 21
2.2.13 *syn*-2-fluoro-4-methoxypentane (*syn*-K) 21
2.2.14 *anti*-2-fluoro-4-methoxypentane (*anti*-K) 22

2.3 Table S13. Characteristic properties of the OH•••F IMHB encountered in the relevant conformers of compounds A-I. AIM electron density (ρ, e bohr^{-3}) at the bond critical points, energy of H-bond (E_{HB}, kJ·mol^{-1}), NCI attractive and repulsive contributions of the OH•••F interaction (sign(λ_2)ρ, e bohr^{-3}) and NBO interaction energy ($E^{(2)}_{n\rightarrow\sigma^*}$, kJ mol^{-1}) 23

3 NCI (Non-Covalent Interaction) analysis 24

3.1 Table S14. NCI isosurfaces and electron densities features, sign(λ_2)ρ and reduced density gradient (RDG) on IEFPCM-MP2/6-311++G(2d,p) wavefunctions in CCl_4 for the main conformers of compound *syn*-A. 24

3.2 Table S15. NCI isosurfaces and electron densities features, sign(λ_2)ρ and reduced density gradient (RDG) on IEFPCM-MP2/6-311++G(2d,p) wavefunctions in CCl_4 for the main conformers of compound *anti*-A. 25

3.3 Table S16. NCI isosurfaces and electron densities features, sign(λ_2)ρ and reduced density gradient (RDG) on IEFPCM-MP2/6-311++G(2d,p) wavefunctions in CCl_4 for the main conformers of compound B. 26

3.4 Table S17. NCI isosurfaces and electron densities features, sign(λ_2)ρ and reduced density gradient (RDG) on IEFPCM-MP2/6-311++G(2d,p) wavefunctions in CCl_4 for the main conformers of compound C. 27

3.5 Table S18. NCI isosurfaces and electron densities features, sign(λ_2)ρ and reduced density gradient (RDG) on IEFPCM-MP2/6-311++G(2d,p) wavefunctions in CCl_4 for the main conformers of compound D. 28

3.6 Table S19. NCI isosurfaces and electron densities features, sign(λ_2)ρ and reduced density gradient (RDG) on IEFPCM-MP2/6-311++G(2d,p) wavefunctions in CCl_4 for the main conformers of compound E. 28

3.7 Table S20. NCI isosurfaces and electron densities features, sign(λ_2)ρ and reduced density gradient (RDG) on IEFPCM-MP2/6-311++G(2d,p) wavefunctions in CCl_4 for the main conformers of compound F. 29

3.8 Table S21. NCI isosurfaces and electron densities features, sign(λ_2)ρ and reduced density gradient (RDG) on IEFPCM-MP2/6-311++G(2d,p) wavefunctions in CCl_4 for the main conformers of compound G. 30

3.9 Table S22. NCI isosurfaces and electron densities features, sign(λ_2)ρ and reduced density gradient (RDG) on IEFPCM-MP2/6-311++G(2d,p) wavefunctions in CCl_4 for the main conformers of compound H. 30

3.10 Table S23. NCI isosurfaces and electron densities features, sign(λ_2)ρ and reduced density gradient (RDG) on IEFPCM-MP2/6-311++G(2d,p) wavefunctions in CCl_4 for the main conformers of compound I. 31

4 Table S24: Calculated NMR coupling constants.. 32

1 Conformational descriptors

For ease of comparison between the different compounds, the following dihedral angles χ, φ, ψ are defined as shown in below. For each conformation, these dihedrals are indicated in this order, with the latter between brackets.

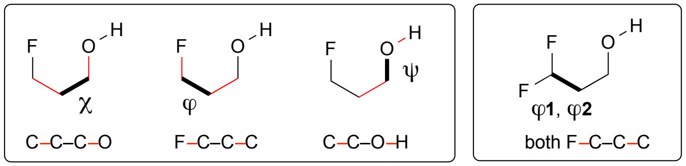

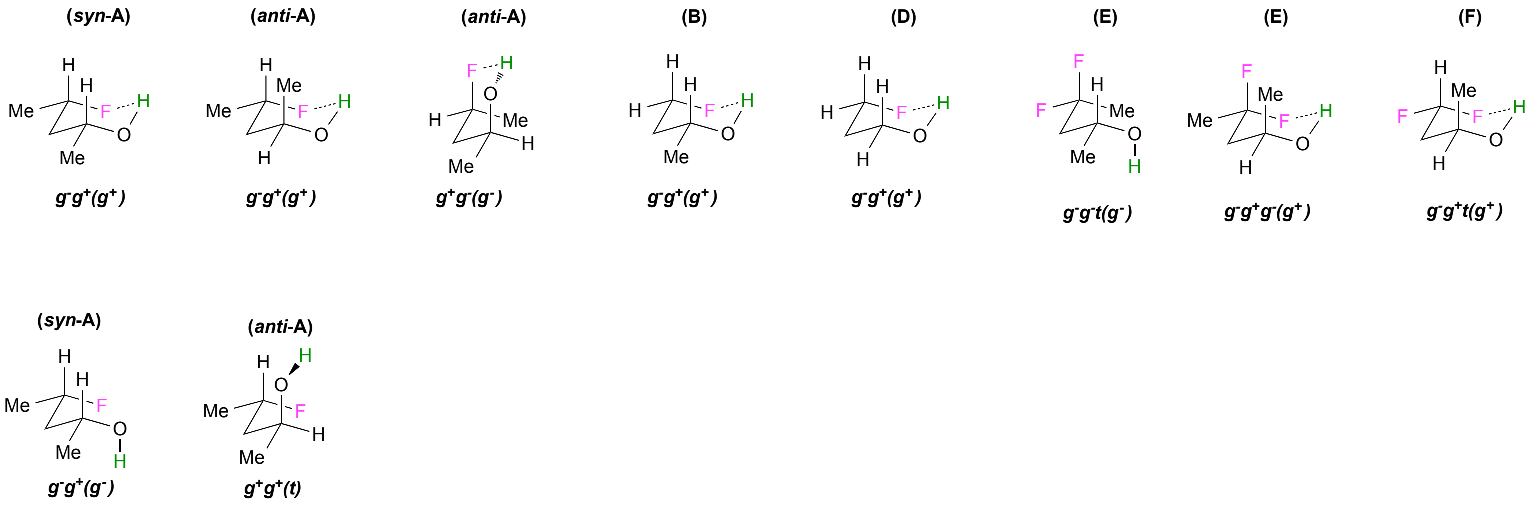

For 4,4-difluoropentan-2-ol (**E**), 4,4-difluorobutanol (**F**), and 3,3-difluoropropanol (**G**), the two φ-dihedrals need to be provided (no distinction is necessary). In many cases non-relevant C-O rotamers are grouped together. For the trifluoroderivatives, only two dihedrals are required.

2 Conformational analysis

2.1 Energetic distribution of analyzed compounds

2.1.1 Table S1. Free energy differences (ΔG, kJ mol^{-1}) and conformational distribution (p_i, %) of *syn*-4-fluoropentan-2-ol (*syn*-A)

in various solvents (CCl_4, $CHCl_3$ and CH_2Cl_2) at various temperatures (298K and 223K) calculated at the IEFPCM-MP2/6-311++G(2d,p)//MPWB1K/6-31+G(d,p) level of theory.

		CCl_4	298K	$CHCl_3$	298K	CH_2Cl_2	298K	$CHCl_3$	223K		$CHCl_3$	298K
		ΔG	p_i	ΔG	p_i	ΔG	p_i	ΔG	p_i		μ (D)	
***syn*-A1**	$g^-g^+(g^+)$	0.0	44.7%	0.0	38.9%	0.0	36.0%	0.0	59.0%		4.002	
***syn*-A2**	$tg^+(g^+)$	3.4	11.2%	2.7	12.9%	2.4	13.7%	3.4	9.7%		2.132	
***syn*-A3**	$tg^+(t)$	3.8	9.5%	3.2	10.6%	2.9	11.0%	3.7	8.0%		1.828	
***syn*-A4**	$g^-t(g^-)$	4.4	7.7%	3.8	8.5%	3.5	8.6%	4.2	6.2%		2.339	
***syn*-A5**	$g^-t(t)$	4.4	7.6%	4.0	7.7%	3.9	7.6%	4.0	6.8%		4.122	
***syn*-A6**	$g^-g^-(t)$	6.2	3.6%	6.3	3.1%	6.3	2.8%	6.6	1.7%		1.737	
***syn*-A7**	$g^-t(g^+)$	6.2	3.6%	5.2	4.8%	4.6	5.6%	6.0	2.3%		2.625	
***syn*-A8**	$tg^+(g^-)$	6.3	3.5%	5.3	4.6%	4.8	5.3%	5.8	2.6%		3.896	
***syn*-A9**	$g^+g^+(t)$	6.9	2.7%	7.1	2.2%	7.2	2.0%	7.1	1.3%		1.923	
***syn*-A10**	$g^+g^+(g^-)$	7.3	2.3%	7.2	2.2%	7.1	2.0%	8.0	0.8%		0.863	
***syn*-A11**	$g^-g^-(g^-)$	8.7	1.3%	8.3	1.4%	8.1	1.4%	8.8	0.5%		3.427	
***syn*-A12**	$g^+g^+(g^+)$	10.1	0.8%	9.4	0.9%	9.1	0.9%	9.2	0.4%		3.498	
***syn*-A13**	$g^-g^+(t)$	10.8	0.6%	8.4	1.3%	7.1	2.1%	9.3	0.4%		4.195	
***syn*-A14**	$g^+t(g^+)$	11.3	0.5%	10.8	0.5%	10.6	0.5%	10.8	0.2%		1.932	
***syn*-A15**	$tg^-(g^-)$	11.6	0.4%	10.9	0.5%	10.6	0.5%	10.9	0.2%		1.242	
										$\bar{\mu}$(D)	3.109	

2.1.2 Table S2. Free energy differences (Δ*G*, kJ mol^{-1}) and conformational distribution (p_i, %) of *anti*-4-fluoropentan-2-ol (*anti*-A) in various solvents (CCl_4, $CHCl_3$ and CH_2Cl_2) at various temperatures (298K and 223K) calculated at the IEFPCM-MP2/6-311++G(2d,p)//MPWB1K/6-31+G(d,p) level of theory.

		CCl_4	298K	$CHCl_3$	298K	CH_2Cl_2	298K	$CHCl_3$	223K		$CHCl_3$	298K
		ΔG	p_i	ΔG	p_i	ΔG	p_i	ΔG	p_i		μ (D)	
***anti*-A1**	*$g^-g^-(t)$*	0.0	49.0%	0.0	45.2%	0.0	42.4%	0.0	60.7%		1.938	
***anti*-A2**	*$g^-g^-(g^-)$*	3.2	13.3%	2.5	16.2%	2.1	18.2%	2.5	15.7%		3.840	
***anti*-A3**	*$g^-g^-(g^+)$*	4.5	8.0%	4.9	6.2%	5.2	5.2%	4.5	5.3%		2.237	
***anti*-A4**	*$g^-g^+(g^+)$*	5.0	6.4%	5.1	5.8%	5.1	5.5%	4.8	4.6%		4.022	
***anti*-A5**	*$tg^-(t)$*	5.9	4.6%	5.6	4.7%	5.4	4.8%	5.9	2.5%		1.636	
***anti*-A6**	*$tt(g^+)$*	6.8	3.2%	5.9	4.3%	5.3	5.0%	6.2	2.2%		2.166	
***anti*-A7**	*$g^+g^-(g^-)$*	7.0	2.9%	7.1	2.6%	7.1	2.4%	6.4	2.0%		3.945	
***anti*-A8**	*$g^-t(t)$*	7.2	2.7%	7.2	2.5%	7.2	2.3%	7.2	1.3%		3.706	
***anti*-A9**	*$tt(t)$*	7.9	2.0%	6.7	3.0%	6.1	3.6%	6.9	1.5%		4.215	
***anti*-A10**	*$tg^-(g^+)$*	8.0	1.9%	7.0	2.7%	6.4	3.2%	7.2	1.2%		3.715	
***anti*-A11**	*$tg^-(g^-)$*	8.0	1.9%	7.5	2.2%	7.3	2.3%	7.8	0.9%		1.398	
***anti*-A12**	*$tt(g^-)$*	8.2	1.8%	7.2	2.5%	6.6	2.9%	7.4	1.1%		2.335	
***anti*-A13**	*$g^-t(g^-)$*	8.3	1.7%	8.0	1.8%	7.8	1.8%	8.1	0.8%		2.308	
***anti*-A14**	*$g^+g^-(g^+)$*	11.8	0.4%	11.9	0.4%	11.9	0.4%	11.6	0.1%		3.546	
										$\bar{\mu}$(D)	2.604	

2.1.3 Table S3. Free energy differences (ΔG, kJ mol^{-1}) and conformational distribution (p_i, %) of 4-fluorobutan-2-ol (B)

in various solvents (CCl_4, $CHCl_3$ and CH_2Cl_2) at various temperatures (298K and 223K) calculated at the IEFPCM-MP2/6-311++G(2d,p)//MPWB1K/6-31+G(d,p) level of theory.

		**CCl_4**	**298K**	**$CHCl_3$**	**298K**	**CH_2Cl_2**	**298K**	**$CHCl_3$**	**223K**		**$CHCl_3$**	**298K**
		**ΔG**	**p_i**	**ΔG**	**p_i**	**ΔG**	**p_i**	**ΔG**	**p_i**		**μ (D)**	
**B1**	$g^-g^-(t)$	0.0	25.2%	0.0	22.5%	0.0	20.7%	0.0	32.7%		2.126	
**B2**	$g^-g^-(g^+)$	1.1	16.0%	1.3	13.3%	1.4	11.8%	1.7	13.0%		1.570	
**B3**	$g^-g^+(g^+)$	2.0	11.3%	2.0	9.9%	2.0	9.3%	1.6	13.7%		3.771	
**B4**	$g^-g^-(g^+)$	3.1	7.3%	2.4	8.5%	2.0	9.4%	2.4	9.0%		3.813	
**B5**	$g^-t(t)$	3.1	7.2%	2.8	7.4%	2.6	7.3%	2.8	7.1%		4.089	
**B6**	$tg^+(t)$	3.5	6.2%	2.9	7.0%	2.6	7.3%	3.2	5.7%		1.986	
**B7**	$tg^+(g^+)$	4.2	4.6%	3.6	5.3%	3.2	5.6%	3.9	4.0%		1.774	
**B8**	$g^-t(g^+)$	5.4	2.8%	4.4	3.9%	3.7	4.6%	5.2	2.0%		2.456	
**B9**	$g^-t(g^-)$	5.5	2.7%	4.9	3.1%	4.6	3.2%	5.0	2.3%		2.599	
**B10**	$tg^-(g^+)$	5.7	2.5%	4.7	3.4%	4.1	4.0%	5.2	2.0%		3.502	
**B11**	$tg^+(g^-)$	5.9	2.3%	4.9	3.1%	4.3	3.6%	5.3	1.9%		3.769	
**B12**	$tg^-(t)$	6.2	2.0%	5.9	2.0%	5.8	2.0%	6.0	1.3%		1.636	
**B13**	$tg^-(g^-)$	6.7	1.7%	6.1	1.9%	5.8	2.0%	6.5	1.0%		1.098	
**B14**	$g^+t(t)$	7.3	1.3%	6.9	1.4%	6.7	1.4%	7.2	0.7%		4.174	
**B15**	$g^+g^+(t)$	7.8	1.1%	8.0	0.9%	8.1	0.8%	7.6	0.5%		2.116	
**B16**	$g^+g^+(g^-)$	8.0	1.0%	7.9	0.9%	7.8	0.9%	8.2	0.4%		0.665	
**B17**	$tt(t)$	8.2	0.9%	7.1	1.3%	6.5	1.5%	7.4	0.6%		4.030	
**B18**	$g^+g^-(g^-)$	8.4	0.9%	8.4	0.8%	8.4	0.7%	7.7	0.5%		3.728	
**B19**	$tt(g^+)$	8.6	0.8%	7.7	1.0%	7.1	1.2%	7.8	0.5%		1.727	
**B20**	$g^+t(g^+)$	8.8	0.7%	8.2	0.8%	7.9	0.8%	8.2	0.4%		2.299	
**B21**	$tt(g^-)$	9.8	0.5%	8.7	0.7%	8.1	0.8%	8.9	0.3%		2.432	
**B22**	$g^+g^-(g^+)$	10.0	0.4%	10.0	0.4%	9.9	0.4%	10.1	0.1%		3.413	
**B23**	$g^+g^+(g^+)$	11.0	0.3%	10.4	0.3%	10.0	0.4%	9.8	0.2%		3.495	
**B24**	$g^+t(g^-)$	12.0	0.2%	10.9	0.3%	10.3	0.3%	11.3	0.1%		2.980	
										$\bar{\mu}$(D)	2.633	

2.1.4 Table S4. Free energy differences (Δ*G*, kJ mol^{-1}) and conformational distribution (p_i, %) of 2,2-dimethyl-3-fluoropropan-1-ol (C) in various solvents (CCl_4, $CHCl_3$ and CH_2Cl_2) at various temperatures (298K and 223K) calculated at the IEFPCM-MP2/6-311++G(2d,p)//MPWB1K/6-31+G(d,p) level of theory.

		CCl_4	298K	$CHCl_3$	298K	CH_2Cl_2	298K	$CHCl_3$	223K		$CHCl_3$	298K
		ΔG	p_i	ΔG	p_i	ΔG	p_i	ΔG	p_i		μ (D)	
**C1**	$g^-g^-(g^+)$	0.0	30.1%	0.0	26.5%	0.0	24.3%	0.2	28.1%		1.598	
**C2**	$g^-g^-(t)$	0.2	27.2%	0.1	25.0%	0.1	23.7%	0.0	31.5%		1.956	
**C3**	$g^-t(t)$	2.8	9.9%	2.4	10.0%	2.2	9.9%	2.3	9.0%		3.826	
**C4**	$g^-g^+(g^+)$	3.4	7.5%	3.1	7.6%	2.9	7.5%	2.5	8.2%		3.613	
**C5**	$g^-t(g^-)$	3.8	6.4%	3.1	7.5%	2.8	8.0%	2.9	6.4%		2.455	
**C6**	$tg^+(t)$	4.8	4.4%	4.5	4.3%	4.4	4.2%	4.2	3.2%		1.638	
**C7**	$g^-g^-(g^-)$	5.1	3.8%	4.0	5.3%	3.4	6.3%	3.4	5.0%		3.900	
**C8**	$tg^+(g^+)$	5.2	3.7%	4.5	4.4%	4.1	4.7%	4.2	3.2%		1.543	
**C9**	$g^-t(g^+)$	6.1	2.5%	4.9	3.6%	4.2	4.4%	5.1	2.0%		2.457	
**C10**	$tt(t)$	6.4	1.1%	5.7	1.3%	5.3	1.4%	5.8	0.7%		3.598	
**C11**	$tg^-(g^+)$	6.5	2.2%	5.4	3.0%	4.8	3.6%	5.1	2.0%		3.706	
**C12**	$tt(g^+)$	8.8	0.9%	7.9	1.1%	7.3	1.3%	7.6	0.5%		1.950	
**C13**	$g^-g^+(t)$	11.8	0.3%	9.7	0.5%	8.5	0.8%	9.9	0.1%		3.944	
										$\bar{\mu}$(D)	2.385	

2.1.5 Table S5. Free energy differences (ΔG, kJ mol^{-1}) and conformational distribution (p_i, %) of 3-fluoropropan-1-ol (D) in various solvents (CCl_4, $CHCl_3$ and CH_2Cl_2) at various temperatures (298K and 223K) calculated at the IEFPCM-MP2/6-311++G(2d,p)//MPWB1K/6-31+G(d,p) level of theory.

		CCl_4	298K	$CHCl_3$	298K	CH_2Cl_2	298K	$CHCl_3$	223K		$CHCl_3$	298K
		ΔG	p_i	ΔG	p_i	ΔG	p_i	ΔG	p_i		μ (D)	
**D1**	$g^-g^-(t)$	0.0	29.9%	0.0	25.4%	0.0	22.8%	0.0	33.9%		1.986	
**D2**	$g^-g^-(g^+)$	1.1	19.1%	1.0	16.8%	1.0	15.4%	1.4	15.7%		1.369	
**D3**	$g^-g^-(g^-)$	2.8	9.5%	1.9	11.7%	1.4	13.2%	1.9	12.4%		3.881	
**D4**	$g^-g^+(g^+)$	2.9	9.4%	2.7	8.4%	2.6	7.9%	2.3	9.6%		3.778	
**D5**	$tg^+(t)$	3.0	8.9%	2.4	9.7%	2.0	10.0%	2.6	8.4%		1.810	
**D6**	$g^-t(t)$	3.6	7.1%	3.1	7.2%	2.9	7.1%	3.2	6.1%		4.007	
**D7**	$tg^-(g^-)$	4.3	5.2%	3.5	6.3%	3.0	6.8%	3.6	4.8%		1.692	
**D8**	$g^-t(g^-)$	4.9	4.2%	4.1	4.8%	3.7	5.1%	4.2	3.6%		2.363	
**D9**	$tg^+(g^-)$	5.4	3.5%	4.1	4.9%	3.3	5.9%	4.2	3.5%		3.854	
**D10**	$tt(t)$	7.0	0.9%	5.8	1.2%	5.1	1.4%	6.2	0.6%		3.880	
**D11**	$tt(g^-)$	8.0	1.2%	6.7	1.7%	6.0	2.0%	7.0	0.8%		1.954	
**D12**	$g^-t(g^+)$	8.8	0.9%	7.5	1.2%	6.8	1.5%	7.7	0.5%		2.460	
**D13**	$g^-g^+(t)$	11.8	0.3%	9.6	0.5%	8.2	0.8%	9.7	0.2%		4.051	
										$\bar{\mu}$(D)	2.514	

2.1.6 Table S6. Free energy differences (ΔG, kJ mol^{-1}) and conformational distribution (p_i, %) of 4,4-difluoropentan-2-ol (E) in various solvents (CCl_4, and $CHCl_3$) at various temperatures (298K and 223K) calculated at the IEFPCM-MP2/6-311++G(2d,p)//MPWB1K/6-31+G(d,p) level of theory.

		CCl_4	298K	$CHCl_3$	298K	$CHCl_3$	223K		$CHCl_3$	298K
		ΔG	p_i	ΔG	p_i	ΔG	p_i		μ (D)	
**E1**	$g^-g^-t(t)$	0.0	49.5%	0.0	48.2%	0.0	47.8%		3.594	
**E2**	$g^-g^+g^-(g^+)$	1.0	33.0%	1.1	30.6%	0.5	36.9%		3.468	
**E3**	$g^-g^+t(g^+)$	3.4	12.7%	3.2	13.3%	2.6	12.0%		3.526	
**E4**	$g^-g^+g^-(t)$	8.7	1.5%	7.4	2.4%	7.3	0.9%		2.639	
**E5**	$tg^+g^-(t)$	9.0	1.3%	8.8	1.4%	8.1	0.6%		1.030	
**E6**	$g^-g^+t(t)$	9.9	0.9%	8.1	1.8%	7.3	0.9%		5.074	
**E7**	$tg^-t(t)$	10.6	0.7%	9.6	1.0%	8.7	0.4%		4.073	
**E8**	$g^-g^+g^-(g^-)$	11.2	0.5%	9.1	1.2%	9.2	0.3%		4.816	
								$\bar{\mu}$(D)	3.534	

2.1.7 Table S7. Free energy differences (ΔG, kJ mol^{-1}) and conformational distribution (p_i, %) of 4,4-difluorobutan-2-ol (F) in various solvents (CCl4, and CHCl3) at various temperatures (298K and 223K) calculated at the IEFPCM-MP2/6-311++G(2d,p)//MPWB1K/6-31+G(d,p) level of theory.

		CCl_4	298K	$CHCl_3$	298K	$CHCl_3$	223K		$CHCl_3$	298K
		ΔG	p_i	ΔG	p_i	ΔG	p_i		μ (D)	
**F1**	$g^-g^-t(t)$	0.0	53.0%	0.0	47.9%	0.0	65.5%		3.899	
**F2**	$g^-g^-t(g^-)$	3.4	13.6%	2.8	15.6%	2.8	14.7%		3.893	
**F3**	$g^-g^+g^-(g^+)$	5.2	6.6%	5.3	5.5%	5.0	4.5%		2.965	
**F4**	$g^-g^+t(g^+)$	5.9	4.8%	5.9	4.5%	5.5	3.4%		2.987	
**F5**	$tg^+t(t)$	6.5	3.8%	5.9	4.5%	6.1	2.5%		3.063	
**F6**	$g^+g^+g^-(t)$	6.6	3.7%	6.7	3.2%	6.7	1.7%		4.019	
**F7**	$tg^+t(g^+)$	6.9	3.3%	6.2	3.9%	6.4	2.1%		0.976	
**F8**	$tg^-t(t)$	8.0	2.1%	7.5	2.4%	7.9	0.9%		2.820	
**F9**	$g^-g^+t(t)$	8.0	2.1%	6.2	3.9%	7.0	1.5%		1.441	
**F10**	$tg^+t(g^-)$	9.0	1.4%	7.9	2.0%	8.1	0.8%		3.591	
**F11**	$g^+g^+g^-(g^+)$	9.4	1.2%	8.8	1.4%	8.6	0.6%		3.425	
**F12**	$tg^+g^-(t)$	9.5	1.1%	9.4	1.1%	9.5	0.4%		1.043	
**F13**	$tg^-t(g^-)$	10.1	0.9%	9.3	1.1%	9.7	0.3%		1.441	
**F14**	$g^-g^+g^-(t)$	10.1	0.9%	8.7	1.5%	9.3	0.4%		3.174	
**F15**	$tg^+g^-(g^-)$	10.8	0.7%	10.3	0.8%	10.6	0.2%		2.669	
**F16**	$tg^-t(g^+)$	10.8	0.7%	9.6	1.0%	9.9	0.3%		2.875	
								$\bar{\mu}$(D)	3.437	

2.1.8 Table S8. Free energy differences (ΔG, kJ mol^{-1}) and conformational distribution (p_i, %) of 3,3-difluoropropan-1-ol (G) in various solvents (CCl_4, and $CHCl_3$) at various temperatures (298K and 223K) calculated at the IEFPCM-MP2/6-311++G(2d,p)//MPWB1K/6-31+G(d,p) level of theory.

		CCl_4	298K	$CHCl_3$	298K	$CHCl_3$	223K		$CHCl_3$	298K
		**ΔG**	p_i	**ΔG**	p_i	**ΔG**	p_i		**μ (D)**	
**G1**	$g^-g^-t(t)$	0.0	51.2%	0.0	43.3%	0.0	58.7%		3.697	
**G2**	$g^-g^-t(g^-)$	3.4	13.2%	2.5	15.7%	2.4	15.9%		3.734	
**G3**	$tg^-t(t)$	4.4	8.6%	3.7	9.9%	4.1	6.6%		3.343	
**G4**	$g^-g^+t(g^+)$	5.8	4.9%	5.5	4.8%	5.1	3.8%		2.919	
**G5**	$tg^+g^-(t)$	4.3	4.5%	3.9	4.4%	4.2	3.0%		1.000	
**G6**	$g^-g^+g^-(g^+)$	6.0	4.6%	6.0	3.9%	5.5	3.1%		2.910	
**G7**	$tg^-t(g^-)$	6.7	3.4%	5.7	4.3%	5.9	2.5%		1.002	
**G8**	$tg^+g^-(g^-)$	6.8	3.3%	5.8	4.1%	6.1	2.2%		3.086	
**G9**	$tg^-t(g^+)$	7.6	2.3%	6.3	3.5%	6.4	1.8%		3.369	
**G10**	$g^-g^+t(g^-)$	9.3	1.2%	8.2	1.6%	8.3	0.7%		2.886	
**G11**	$g^-g^+t(t)$	9.4	1.2%	7.4	2.2%	7.6	1.0%		4.805	
**G12**	$g^-g^+g^-(t)$	9.5	1.1%	8.1	1.7%	8.5	0.6%		2.998	
**G13**	$g^-g^+g^-(g^-)$	12.1	0.4%	10.0	0.8%	10.3	0.2%		4.647	
								$\bar{\mu}$(D)	3.334	

2.1.9 Table S9. Free energy differences (Δ*G*, kJ mol^{-1}) and conformational distribution (p_i, %) of 4,4,4-trifluorobutan-2-ol (H)

in various solvents (CCl_4, $CHCl_3$ and CH_2Cl_2) at various temperatures (298K and 223K) calculated at the IEFPCM-MP2/6-311++G(2d,p)//MPWB1K/6-31+G(d,p) level of theory.

		**CCl_4**	**298K**	**$CHCl_3$**	**298K**	**CH_2Cl_2**	**298K**	**$CHCl_3$**	**223K**		**$CHCl_3$**	**298K**
		**ΔG**	**p_i**	**ΔG**	**p_i**	**ΔG**	**p_i**	**ΔG**	**p_i**		**μ (D)**	
**H1**	$g^-(g^+)$	0.0	61.8%	0.0	47.1%	0.0	39.2%	0.0	60.7%		2.036	
**H2**	$g^-(t)$	2.6	21.4%	1.0	31.7%	0.1	37.1%	1.6	25.9%		4.427	
**H3**	$g^-(g^-)$	5.8	6.0%	3.8	10.1%	2.8	12.9%	4.1	6.5%		4.339	
**H4**	$t(g^+)$	5.9	5.8%	5.0	6.3%	4.5	6.3%	5.2	3.7%		2.085	
**H5**	$t(g^-)$	7.6	2.9%	6.9	3.0%	6.5	2.9%	6.8	1.5%		2.521	
**H6**	$t(t)$	8.4	2.1%	8.0	1.8%	7.9	1.6%	6.6	1.7%		2.928	
										$\bar{\mu}$(D)	3.059	

2.1.10 Table S10. Free energy differences (Δ*G*, kJ mol^{-1}) and conformational distribution (p_i, %) of 3,3,3-trifluoropropan-1-ol (I)

in various solvents (CCl_4, $CHCl_3$ and CH_2Cl_2) at various temperatures (298K and 223K) calculated at the IEFPCM-MP2/6-311++G(2d,p)//MPWB1K/6-31+G(d,p) level of theory.

		**CCl_4**	**298K**	**$CHCl_3$**	**298K**	**CH_2Cl_2**	**298K**	**$CHCl_3$**	**223K**		**$CHCl_3$**	**298K**
		**ΔG**	**p_i**	**ΔG**	**p_i**	**ΔG**	**p_i**	**ΔG**	**p_i**		**μ (D)**	
**I1**	$t(t)$	0.0	34.3%	0.0	30.7%	0.0	28.1%	0.0	33.3%		2.758	
**I2**	$g^-(g^+)$	2.2	28.0%	2.5	22.5%	2.6	19.7%	1.7	26.5%		1.882	
**I3**	$t(g^+)$	2.9	21.3%	2.3	24.4%	1.9	25.9%	2.1	21.0%		2.465	
**I4**	$g^-(t)$	3.6	16.3%	2.5	22.4%	1.9	26.2%	2.3	19.2%		4.136	
										$\bar{\mu}$(D)	2.798	

2.1.11 Table S11. Free energy differences (Δ*G*, kJ mol^{-1}) and conformational distribution (p_i, %) of *syn*-4-methoxypentan-2-ol (*syn*-J) and *anti*-4-methoxypentan-2-ol (*anti*-J)

in various solvents (CCl_4, $CHCl_3$) calculated at the IEFPCM-MP2/6-311++G(2d,p)//MPWB1K/6-31+G(d,p) level of theory.

	CCl_4	298K	$CHCl_3$	298K		CCl_4	298K	$CHCl_3$	298K
	ΔG	p_i	ΔG	p_i		ΔG	p_i	ΔG	p_i
***syn*-J1**	0.0	94.7%	0.0	93.9%	***anti*-J1**	0.0	31.3%	0.0	33.4%
***syn*-J2**	9.2	2.3%	11.6	0.9%	***anti*-J2**	0.1	29.8%	0.4	28.4%
***syn*-J3**	9.5	2.0%	11.7	0.8%	***anti*-J3**	0.9	21.9%	1.1	21.2%
***syn*-J4**	11.4	0.9%	7.6	4.4%	***anti*-J4**	3.3	8.3%	3.7	7.5%
					***anti*-J5**	3.6	7.3%	3.5	8.1%
					***anti*-J6**	7.6	1.5%	7.9	1.4%

2.1.12 Table S12. Free energy differences (Δ*G*, kJ mol^{-1}) and conformational distribution (p_i, %) of *syn*-2-fluoro-4-methoxypentane (*syn*-K) and *anti*-2-fluoro-4-methoxypentane (*anti*-K)

in various solvents (CCl_4, $CHCl_3$) calculated at the IEFPCM-MP2/6-311++G(2d,p)//MPWB1K/6-31+G(d,p) level of theory.

	CCl_4	298K	$CHCl_3$	298K		CCl_4	298K	$CHCl_3$	298K
	ΔG	p_i	ΔG	p_i		ΔG	p_i	ΔG	p_i
***syn*-K1**	0.0	44.7%	0.0	44.3%	***anti*-K1**	0.0	88.3%	0.0	86.6%
***syn*-K2**	1.9	20.7%	1.8	21.2%	***anti*-K2**	6.9	5.5%	6.8	5.5%
***syn*-K3**	2.9	14.0%	2.7	15.1%	***anti*-K3**	9.1	2.2%	8.5	2.9%
***syn*-K4**	3.6	10.3%	4.2	8.2%	***anti*-K4**	9.5	1.9%	9.2	2.1%
***syn*-K5**	7.0	2.6%	7.4	2.3%	***anti*-K5**	10.7	1.2%	9.7	1.7%
***syn*-K6**	7.2	2.4%	7.2	2.5%	***anti*-K6**	11.5	0.8%	10.5	1.3%
***syn*-K7**	7.5	2.2%	7.5	2.2%					
***syn*-K8**	8.0	1.7%	8.0	1..7%					
***syn*-K9**	8.6	1.4%	7.0	2.6%					

2.2 Depiction of the optimized structures at the IEFPCM-MPWB1K/6-31+G(d,p) level of theory

2.2.1 *syn*-4-fluoropentan-2-ol (*syn*-A)

***syn*-A1**

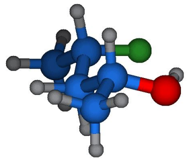

***syn*-A2**

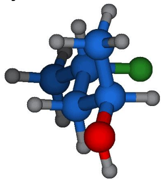

***syn*-A3**

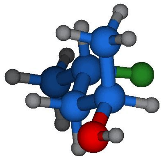

***syn*-A4**

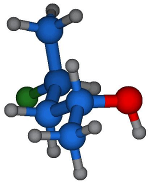

***syn*-A5**

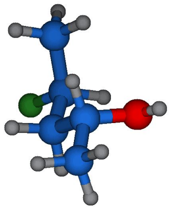

***syn*-A6**

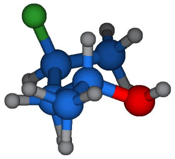

***syn*-A7**

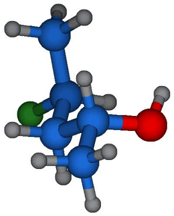

***syn*-A8**

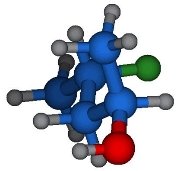

***syn*-A9**

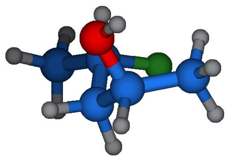

***syn*-A10**

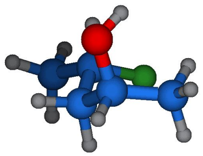

***syn*-A11**

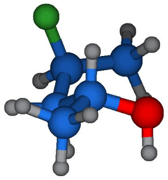

***syn*-A12**

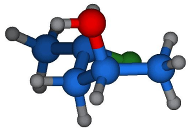

***syn*-A13**

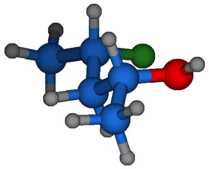

***syn*-A14**

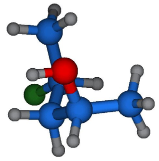

***syn*-A15**

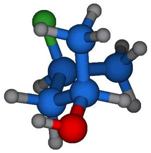

2.2.2 *anti*-4-fluoropentan-2-ol (*anti*-A)

***anti*-A1**

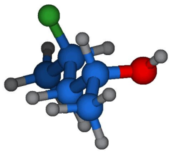

***anti*-A2**

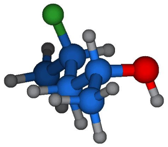

***anti*-A3**

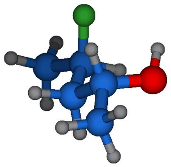

***anti*-A4**

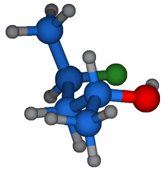

***anti*-A5**

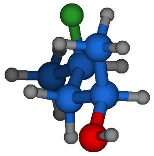

***anti*-A6**

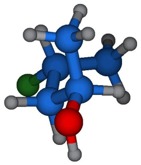

***anti*-A7**

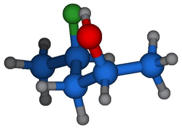

***anti*-A8**

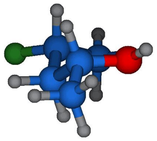

***anti*-A9**

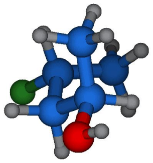

***anti*-A10**

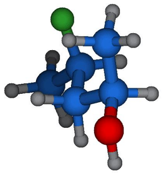

***anti*-A11**

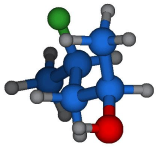

***anti*-A12**

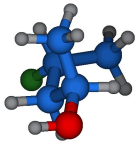

***anti*-A13**

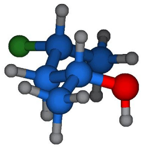

***anti*-A14**

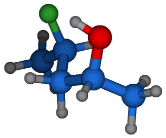

2.2.3 4-fluorobutan-2-ol (B)

B1
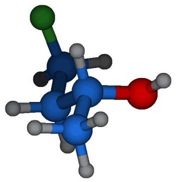
B2
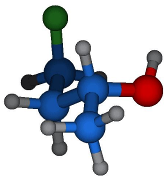
B3
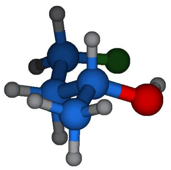
B4
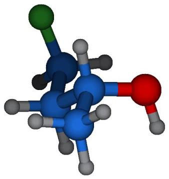
B5
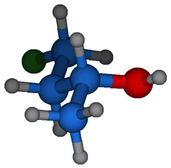
B6
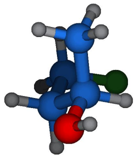
B7
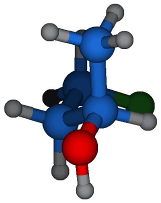
B8
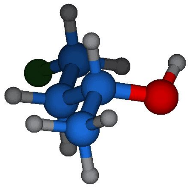
B9
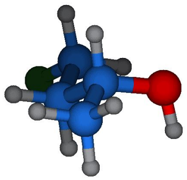
B10
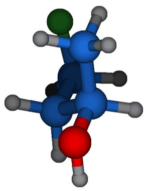
B11
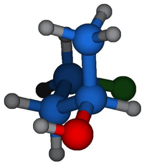
B12
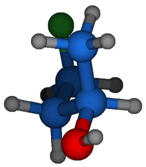
B13
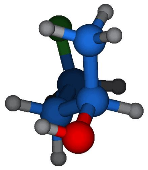
B14
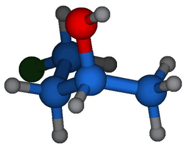
B15
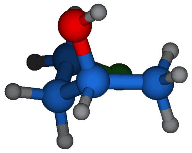
B16
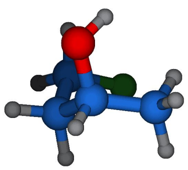
B17
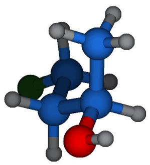
B18
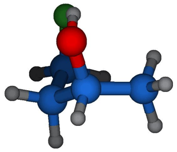
B19
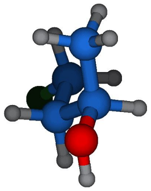
B20
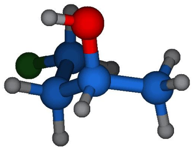
B21
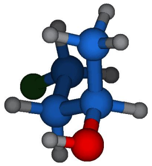
B22
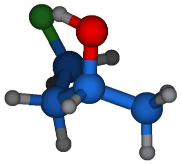
B23
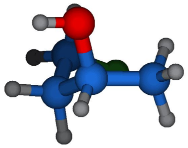
B24
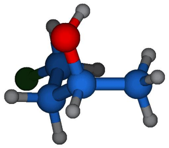

2.2.4 2,2-dimethyl-3-fluoropropan-1-ol (C)

C1
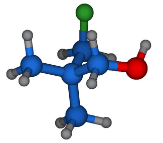
C2
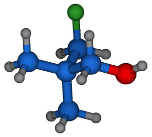
C3
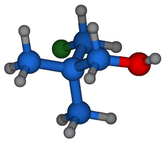
C4
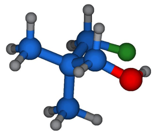
C5
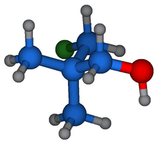
C6
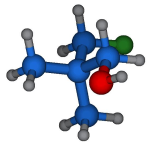
C7
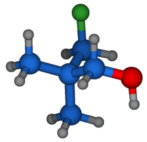
C8
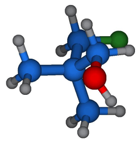
C9
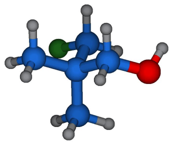
C10
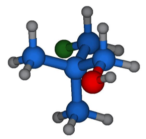
C11
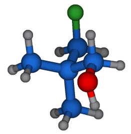
C12
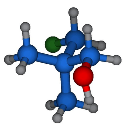
C13
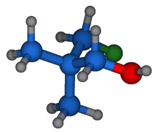

2.2.5 3-fluoropropan-1-ol (D)

D1
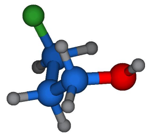
D2
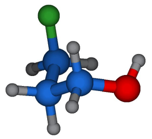
D3
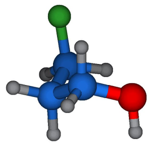
D4
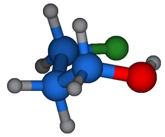
D5
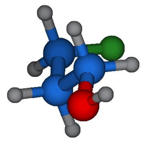
D6
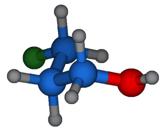
D7
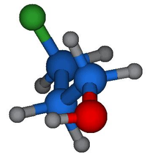
D8
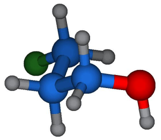
D9
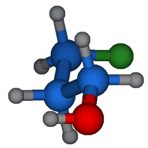
D10
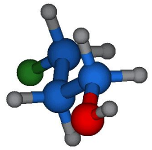
D11
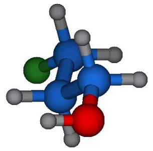
D12
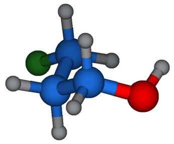
D13
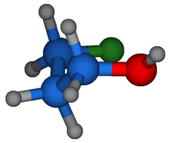

2.2.6 4,4-difluoropentan-2-ol (E)

E1

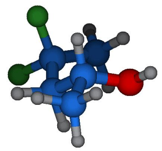

E2

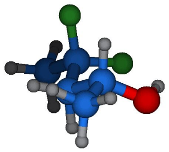

E3

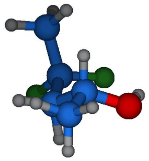

E4

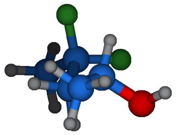

E5

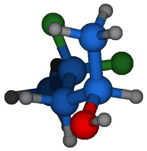

E6

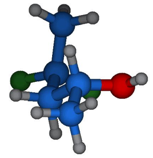

E7

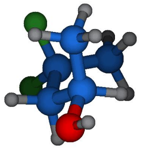

E8

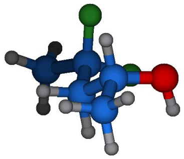

2.2.7 3,3-difluorobutan-2-ol (F)

F1

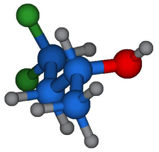

F2

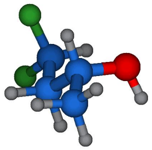

F3

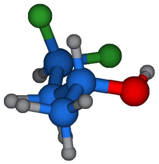

F4

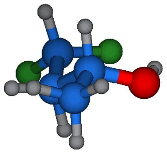

F5

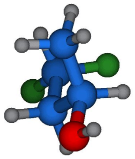

F6

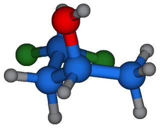

F7

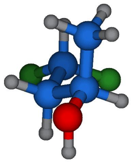

F8

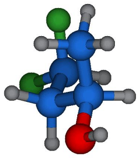

F9

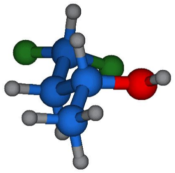

F10

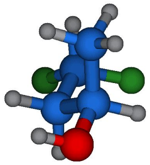

F11

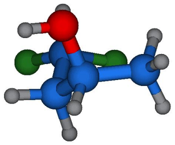

F12

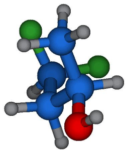

F13

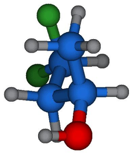

F14

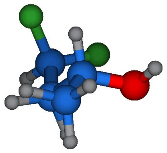

F15

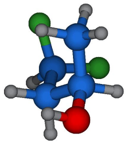

F16

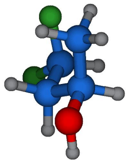

2.2.8 3,3-difluoropropan-1-ol (G)

G1
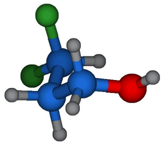
G2
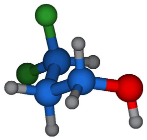
G3
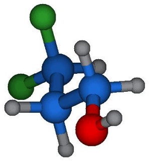
G4
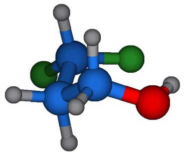
G5
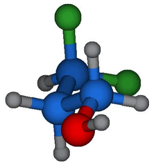
G6
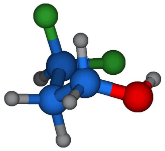
G7
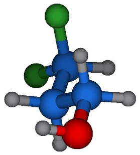
G8
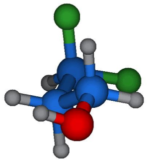
G9
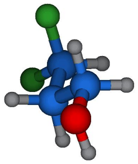
G10
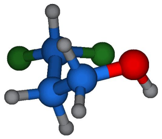
G11
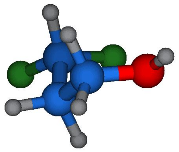
G12
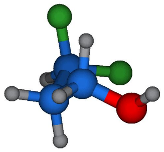
G13
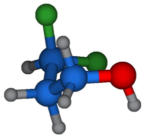

2.2.9 4,4,4-trifluorobutan-2-ol (H)

H1
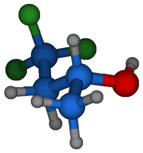
H2
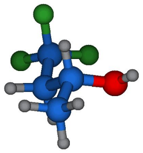
H3
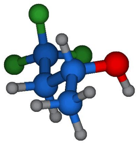
H4
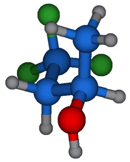
H5
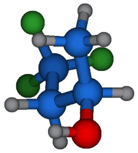
H6
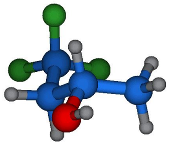

2.2.10 3,3,3-trifluoropropan-1-ol (I)

I1
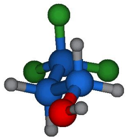
I2
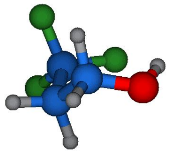
I3
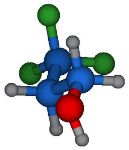
I4
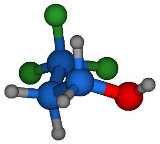

2.2.11 *syn*-4-methoxypentan-2-ol (*syn*-J)

***syn*-J1**

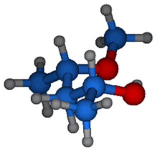

***syn*-J2**

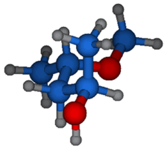

***syn*-J3**

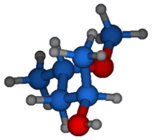

***syn*-J4**

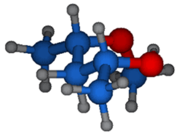

2.2.12 *anti*-4-methoxypentan-2-ol (*anti*-J)

***anti*-J1**

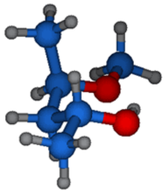

***anti*-J2**

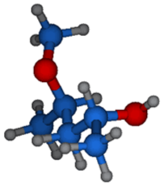

***anti*-J3**

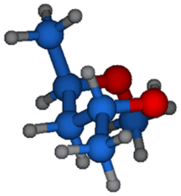

***anti*-J4**

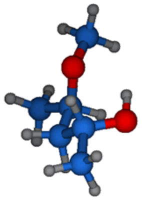

***anti*-J5**

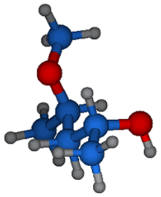

***anti*-J6**

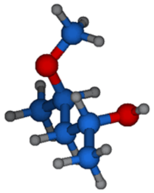

2.2.13 *syn*-2-fluoro-4-methoxypentane (*syn*-K)

***syn*-K1**

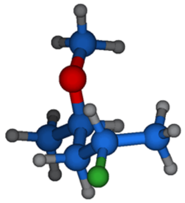

***syn*-K2**

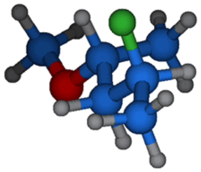

***syn*-K3**

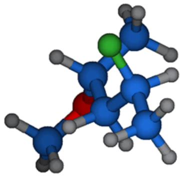

***syn*-K4**

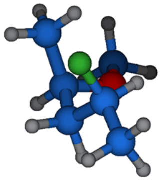

***syn*-K5**

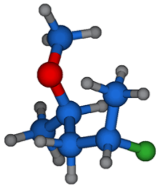

***syn*-K6**

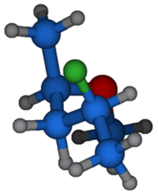

***syn*-K7**

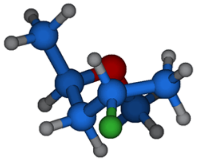

***syn*-K8**

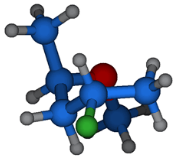

***syn*-K9**

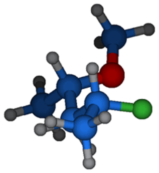

2.2.14 *anti*-2-fluoro-4-methoxypentane (*anti*-K)

***anti*-K1**

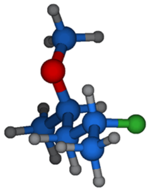

***anti*-K2**

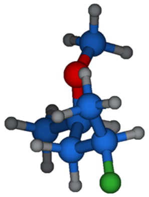

***anti*-K3**

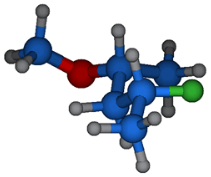

***anti*-K4**

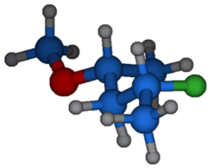

***anti*-K5**

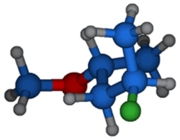

***anti*-K6**

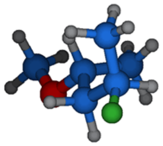

2.3 Table S13. Characteristic properties of the OH•••F IMHB encountered in the relevant conformers of compounds A-I. AIM electron density (ρ, e bohr^{-3}) at the bond critical points, energy of H-bond (E_{HB}, kJ·mol^{-1}), NCI attractive and repulsive contributions of the OH•••F interaction (sign(λ_2)ρ, e bohr^{-3}) and NBO interaction energy ($E^{(2)}_{n\to\sigma^*}$, kJ mol^{-1})

Compound	Conformer	$d_{(OH\cdots F)}$[a,b]	ρ[c]	E_{HB}[c]	sign(λ_2)ρ[c,d]	sign(λ_2)ρ[c,d]	$E^{(2)}_{n\to\sigma^*}$[a]
Syn-A	syn-A1	2.000	0.0206	24.4	-0.0206	0.0142	25.1
Anti-A	anti_A4	2.008	0.0202	23.7	-0.0199	0.0139	24.7
	anti_A7	2.056	0.0186	21.6	-0.0186	0.0139	20.1
B	B3	2.037	0.0190	22.0	-0.0188	0.0134	22.3
	B18	2.091	0.0173	19.8			18.2
C	C4	2.065	0.0184	21.4	-0.0182	0.0134	19.2
D	D4	2.074	0.0178	20.4	-0.0178	0.0132	20.0
E	E2	2.062	0.0185	21.7	-0.0182	0.0142	17.8
	E3	2.050	0.0187	21.7	-0.0184	0.0140	19.4
F	F3	2.133	0.0161	18.4	-0.0161	0.0128	13.8
	F4	2.090	0.0173	19.8	-0.0162	0.0146	16.9
G	G4	2.175	0.0148	16.6	-0.0128	0.0121	9.0
	G6	2.201	0.0142	16.1	-0.0132	0.0118	10.5
H	H1	2.172	0.0151	17.1	-0.0142	0.0126	10.9
I	I2	2.234	0.0136	15.4	-0.0136	0.0125	8.3

[a] At the IEFPCM-MPWB1K/6-31+G(d,p) level. [b] in Å. [c] At the IEFPCM-MP2/6-311++G(2d,p) level. [d] Negative sign(λ_2)ρ values indicate attractive contributions to non-covalent interactions, while positive sign(λ_2)ρ values indicate repulsive contributions

3 NCI (Non-Covalent Interaction) analysis

3.1 Table S14. NCI isosurfaces and electron densities features, sign(λ_2)ρ and reduced density gradient (RDG) on IEFPCM-MP2/6-311++G(2d,p) wavefunctions in CCl_4 for the main conformers of compound *syn*-A.

***syn*-A1**

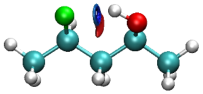

	sign(λ_2)ρ	RDG	sign(λ_2)ρ	RDG
OH···F	-0.0206	0.0720	0.0142	0.0549

***syn*-A2**

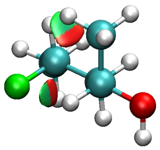

	sign(λ_2)ρ	RDG	sign(λ_2)ρ	RDG
CH···F	-0.0101	0.3014	0.0112	0.2718
CH···H	-0.0095	0.0720	0.0098	0.1455

***syn*-A3**

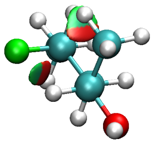

CH···F	-0.0106	0.2847	0.0117	0.2751
CH···H	-0.0096	0.0959	0.0101	0.1061

***syn*-A4**

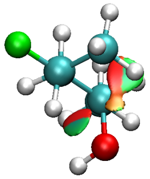

CH···O	-0.0109	0.2488	0.0121	0.2321
CH···H	-0.0094	0.1406	0.0098	0.1145

***syn*-A5**

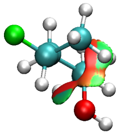

CH···O	-0.0113	0.2132	0.0118	0.2140
CH···H	-0.0096	0.1253	0.0098	0.1415

***syn*-A7**

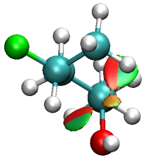

CH···O	-0.0091	0.2627	0.0098	0.2274
CH···H	-0.0091	0.1091	0.0096	0.1307

***syn*-A8**

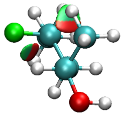

CH···F	-0.0104	0.2850	0.0108	0.2843
CH···H	-0.0095	0.1259	0.0098	0.0838

3.2 Table S15. NCI isosurfaces and electron densities features, sign(λ_2)ρ and reduced density gradient (RDG) on IEFPCM-MP2/6-311++G(2d,p) wavefunctions in CCl_4 for the main conformers of compound *anti*-A.

***anti*-A1**

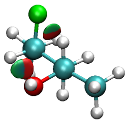

	sign(λ_2)ρ	RDG	sign(λ_2)ρ	RDG
CH···F	-0.0105	0.2834	0.113	0.2638
CH···O	-0.0119	0.2126	0.0123	0.1836

***anti*-A2**

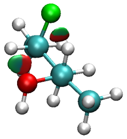

	sign(λ_2)ρ	RDG	sign(λ_2)ρ	RDG
CH···F	-0.0102	0.3066	0.0117	0.2816
CH···O	-0.0116	0.2439	0.0122	0.2094

***anti*-A3**

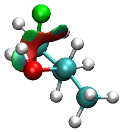

	sign(λ_2)ρ	RDG	sign(λ_2)ρ	RDG
CH···O	-0.0118	0.3111	0.0130	0.2720
OH···F	-0.0085	0.2112	0.0089	0.1965

***anti*-A4**

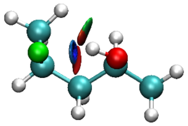

	sign(λ_2)ρ	RDG	sign(λ_2)ρ	RDG
OH···F	-0.0199	0.0594	0.0139	0.0726
CH···H	-0.0097	0.1025	0.0101	0.0717

***anti*-A7**

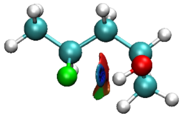

	sign(λ_2)ρ	RDG	sign(λ_2)ρ	RDG
OH···F	-0.0186	0.0521	0.0139	0.0718
CH···H	-0.0098	0.1025	0.0101	0.0718

3.3 Table S16. NCI isosurfaces and electron densities features, sign(λ_2)ρ and reduced density gradient (RDG) on IEFPCM-MP2/6-311++G(2d,p) wavefunctions in CCl_4 for the main conformers of compound B.

		sign(λ_2)ρ	RDG	sign(λ_2)ρ	RDG			sign(λ_2)ρ	RDG	sign(λ_2)ρ	RDG
**B1** 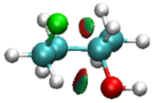	CH···F	-0.0099	0.4455	0.0105	0.4480	**B2** 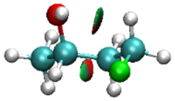	CH···F	-0.0097	0.4073	0.0110	0.3903
	CH···O	-0.0114	0.2511	0.0125	0.2122		CH···O	-0.0104	0.2800	0.0116	0.2321
**B3** 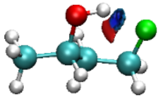	OH···F	-0.0188	0.0998	0.0134	0.1047	**B4** 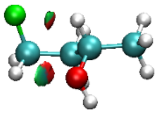	CH···F	-0.0091	0.4844	0.0098	0.4821
							CH···O	-0.0013	0.2264	0.0119	0.2571
**B5** 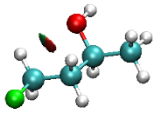	CH···O	-0.0113	0.2355	0.0117	0.2210	**B6** 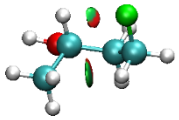	CH···F	-0.0092	0.1948	0.0094	0.3389
							H···H	-0.0095	0.0965	0.0099	0.1013

3.4 Table S17. NCI isosurfaces and electron densities features, sign(λ_2)ρ and reduced density gradient (RDG) on IEFPCM-MP2/6-311++G(2d,p) wavefunctions in CCl_4 for the main conformers of compound C.

		sign(λ_2)ρ	RDG	sign(λ_2)ρ	RDG
**C1**					
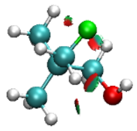	CH···F	-0.0082	0.5529	0.0112	0.4344
		-0.0111	0.3166	0.0142	0.3423
	CH···O	-0.0107	0.3067	0.0129	0.3246
		-0.0097	0.3154	0.0112	0.3160
**C3**					
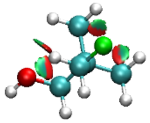	CH···F	-0.0085	0.5168	0.0126	0.3396
		-0.0102	0.4243	0.0112	0.4023
	CH···O	-0.0111	0.2878	0.0116	0.2805
		-0.0116	0.3039	0.0130	0.2601
**C5**					
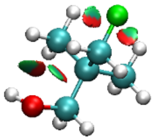	CH···F	-0.0081	0.5566	0.0111	0.3329
		-0.0105	0.4094	0.0110	0.3908
	CH···O	-0.0108	0.2792	0.0111	0.2109
		-0.0100	0.2496	0.0111	0.2293

		sign(λ_2)ρ	RDG	sign(λ_2)ρ	RDG
**C2**					
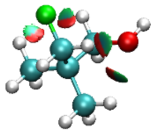	CH···F	-0.0108	0.4102	0.0115	0.2872
		-0.0102	0.3705	0.0117	0.2997
	CH···O	-0.0117	0.2626	0.0136	0.2417
		-0.0114	0.2378	0.0127	0.2050
**C4**					
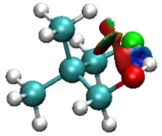	OH···F	-0.0182	0.1172	0.0134	0.1359
	CH···F/	-0.0121	0.3020	0.0135	0.3348
	CH···O				

3.5 Table S18. NCI isosurfaces and electron densities features, sign(λ_2)ρ and reduced density gradient (RDG) on IEFPCM-MP2/6-311++G(2d,p) wavefunctions in CCl_4 for the main conformers of compound D.

D1

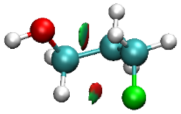

	sign(λ_2)ρ	RDG	sign(λ_2)ρ	RDG
CH···F	-0.0089	0.3842	0.0098	0.3397
CH···O	-0.0105	0.2574	0.0113	0.2196

D2

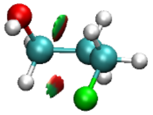

	sign(λ_2)ρ	RDG	sign(λ_2)ρ	RDG
CH···F	-0.0093	0.4047	0.0097	0.3580
CH···O	-0.0096	0.3012	0.0101	0.2805

D3

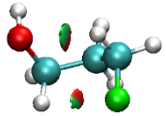

CH···F	-0.0084	0.3962	0.0094	0.3627
CH···O	-0.0104	0.2888	0.1061	0.2798

D4

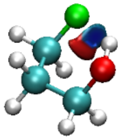

OH···F	-0.01775	0.1254	0.0132	0.1844

D5

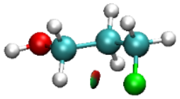

CH···F	-0.0081	0.4824	0.0095	0.3890

D6

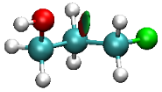

CH···O	-0.0100	0.3106	0.0105	0.2859

3.6 Table S19. NCI isosurfaces and electron densities features, sign(λ_2)ρ and reduced density gradient (RDG) on IEFPCM-MP2/6-311++G(2d,p) wavefunctions in CCl_4 for the main conformers of compound E.

E1

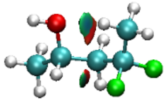

	sign(λ_2)ρ	RDG	sign(λ_2)ρ	RDG
CH···F	-0.0105	0.3471	0.0098	0.0603
CH···O	-0.0122	0.1169	0.0105	0.5618

E2

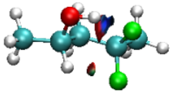

	sign(λ_2)ρ	RDG	sign(λ_2)ρ	RDG
OH···F	-0.0182	0.2476	0.0142	0.2442
CH···F	-0.0101	0.3743	0.0139	0.1434

E3

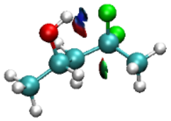

OH···F	-0.0184	0.1207	0.0140	0.2607
H···H	-0.0084	0.2774	0.0096	0.1740

3.7 Table S20. NCI isosurfaces and electron densities features, sign(λ_2)ρ and reduced density gradient (RDG) on IEFPCM-MP2/6-311++G(2d,p) wavefunctions in CCl_4 for the main conformers of compound F.

F1

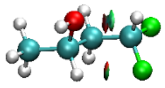

	sign(λ_2)ρ	RDG	sign(λ_2)ρ	RDG
CH···F	-0.0103	0.5832	0.0122	0.3701
CH···O	-0.0119	0.3651	0.0123	0.2761

F2

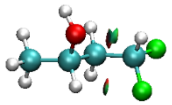

	sign(λ_2)ρ	RDG	sign(λ_2)ρ	RDG
CH···F	-0.0090	0.5849	0.0103	0.4210
CH···O	-0.0106	0.6768	0.0138	0.4144

F3

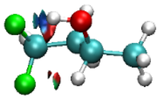

	sign(λ_2)ρ	RDG	sign(λ_2)ρ	RDG
OH···F	-0.0161	0.1575	0.0128	0.1105
CH···F	-0.0097	0.3333	0.0108	0.2888

F4

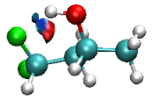

	sign(λ_2)ρ	RDG	sign(λ_2)ρ	RDG
OH···F	-0.0162	0.2283	0.0146	0.2822

F5

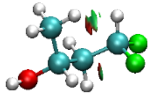

	sign(λ_2)ρ	RDG	sign(λ_2)ρ	RDG
CH···F	-0.0082	0.3061	0.0096	0.2586
H···H	-0.0087	0.2807	0.0093	0.1989

F6

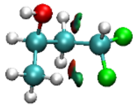

	sign(λ_2)ρ	RDG	sign(λ_2)ρ	RDG
CH···F	-0.0103	0.2946	0.0095	0.3889
CH···O	-0.0106	0.2815	0.0119	0.2689

3.8 Table S21. NCI isosurfaces and electron densities features, sign(λ_2)ρ and reduced density gradient (RDG) on IEFPCM-MP2/6-311++G(2d,p) wavefunctions in CCl_4 for the main conformers of compound G.

G1

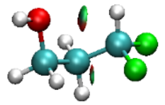

	sign(λ_2)ρ	RDG	sign(λ_2)ρ	RDG
CH···F	-0.0094	0.3878	0.0111	0.3659
CH···O	-0.0102	0.5095	0.0128	0.4789

G2

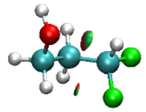

	sign(λ_2)ρ	RDG	sign(λ_2)ρ	RDG
CH···F	-0.0086	0.4718	0.0103	0.4104
CH···O	-0.0098	0.3505	0.0107	0.3308

G3

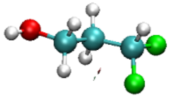

CH···F	-0.0082	0.5871	0.0117	0.5507

G4

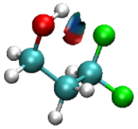

OH···F	-0.0128	0.4121	0.0121	0.1394

G5

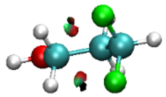

CH···F	-0.0091	0.3737	0.0101	0.3459
CH···F	-0.0091	0.3737	0.0101	0.3459

G6

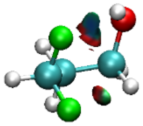

OH···F	-0.0132	0.3355	0.0118	0.2023
CH···F	-0.0096	0.3962	0.0109	0.3751

3.9 Table S22. NCI isosurfaces and electron densities features, sign(λ_2)ρ and reduced density gradient (RDG) on IEFPCM-MP2/6-311++G(2d,p) wavefunctions in CCl_4 for the main conformers of compound H.

H1

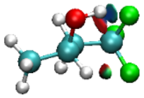

	sign(λ_2)ρ	RDG	sign(λ_2)ρ	RDG
OH···F	-0.0142	0.1790	0.0126	0.2121
CH···F	-0.0086	0.6183	0.0126	0.1326

H2

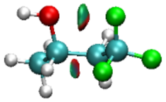

	sign(λ_2)ρ	RDG	sign(λ_2)ρ	RDG
CF···O	-0.0107	0.3356	0.0104	0.3418
CH···F	-0.0101	0.2742	0.0104	0.1204

3.10 Table S23. NCI isosurfaces and electron densities features, sign(λ_2)ρ and reduced density gradient (RDG) on IEFPCM-MP2/6-311++G(2d,p) wavefunctions in CCl_4 for the main conformers of compound I.

I1						I2					
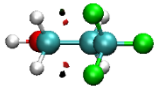		sign(λ_2)ρ	RDG	sign(λ_2)ρ	RDG	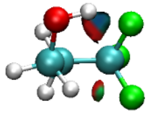		sign(λ_2)ρ	RDG	sign(λ_2)ρ	RDG
	CH···F	-0.0082	0.6511	0.0099	0.4866		OH···F	-0.0136	0.0614	0.0125	0.3142
	CH···F	-0.0082	0.6511	0.0099	0.4866		CH···F	-0.0093	0.5321	0.0104	0.4239

4 Table S24: Calculated NMR coupling constants

Experimental and theoretical values of coupling constants:

syn_FPentOH	exp in CDCl3		theo in CHCl3	
**syn_A**	+25°C	-50°C	+25°C	-50°C
OH … F	6.6	9.9	-7.9	-11.9
OH – H_2	3.4	2.4	3.4	2.2
OH – Me	0.3		0.5	0.7
H_3 – F	13.6	11.8	10.8	9.6
H_3 – H_4	9.0	9.9	9.3	9.9
H_3 – H_2	7.9	8.6	7.7	8.2
H_3’ – F	34.7	39.6	35.1	38.8
H_3' – H_4	4.1	3.3	3.9	3.4
H_3' – H_2	4.1	3.3	4.2	3.2
H_3 – H_3'	14.5	14.8	-13.6	-13.8
H_4 – F	49.5	49.8	50.7	51.1

anti_FPentOH	exp in CDCl3		theo in CHCl3	
**anti_A**	+25°C	-50°C	+25°C	-50°C
**OH … F**	1.9	1.8	-1.5	-1.2
OH – H_2	4.9	4.7	5.0	4.5
OH – H_3	0.5	0.7	0.6	0.8
H_3 – F	15.7	14.7	14.4	13.8
H_3 – H_{4a}	9.3	9.9	9.1	9.7
H_3 – H_{2g}	3.0	2.5	3.5	2.6
H_3' – F	36.0	39.1	34.0	36.4
H_3' – H_{2a}	9.3	10.0	9.2	10.0
H_3' – H_{4g}	2.7	2.3	2.9	2.2
H_4 – F	49.4	49.7	50.3	50.4

FBuOH	exp in CDCl3		theo in CHCl3	
**B**	+25°C	-50°C	+25°C	-50°C
**OH … F**	**2.2**		**-1.8**	**-2.4**
OH – H_2	4.5		4.5	4.0
OH – H_3'	0.3		0.0	0.1
H_3 – H_3'	14.8		-13.7	-13.8
H_3 – H_2	4.3		4.2	3.4
H_3 – H_4	5.0		5.2	4.8
H_3 – H_4'	7.5		8.0	8.5
H_3 – F	25.0		22.7	22.6
$H_{3'}$ – H_2	8.1		8.4	9.2
$H_{3'}$ – H_4	6.0		5.7	5.4
$H_{3'}$ – $H_{4'}$	4.6		4.5	3.9

**B,** *cont’d*	+25°C	-50°C	+25°C	-50°C
$H_{3'}$ – F	29.3		29.2	32.2
F – Me	0.5		0.7	0.5
F – H_4	47.2		48.0	47.9
F – $H_{4'}$	47.2		48.2	48.2

Me_2FPrOH	exp in CDCl3		theo in CHCl3	
**C**	+25°C	-50°C	+25°C	-50°C
OH … F	1.7		**-1.2**	**-1.3**
OH – H_1	5.9		5.9	5.6
F – H_1	1.3		2.3	2.4
F – H_3	47.8		39.8	42.5
F – Me'	1.8		1.4	1.5

FPrOH **D**	exp in CDCl3 +25°C	exp in CDCl3 -50°C	theo in CHCl3 +25°C	theo in CHCl3 -50°C
OH … F	1.4	1.7	-1.2	-1.4
$H_1 – H_2$	6.0	6.0	6.0	5.8
H_1 – OH	5.3	5.2	5.5	5.1
H_2 – F	27.0	28.4	26.5	27.5
$H_2 – H_3$	5.8	5.5	5.7	5.5
H_3 – F	47.0	47.1	48.0	48.0

F_2BuOH **F**	exp in CDCl3 +25°C	exp in CDCl3 -50°C	theo in CHCl3 +25°C	theo in CHCl3 -50°C
OH … F_1	0.6		-0.2	-0.3
OH … F_2	0.6		-0.4	-0.2
OH – H_2	4.5		4.0	3.6
$H_2 – H_3$	8.4		9.0	9.9
$H_2 – H_3'$	4.4		3.6	3.0
H_3 - H_3'	14.5		-13.5	-13.4
F_1 - H_3	14.5		8.8	6.3
F_1 - H_3'	14.5		11.1	10.3
F_2 - H_3	20.9		30.1	32.9
F_2 - H_3'	16.3		16.5	15.2
H_4 - H_3	3.6		2.3	1.9
H_4 - H_3'	5.5		7.3	8.0
$H_4 – F_1$	56.8		56.8	57.0
$H_4 – F_2$	56.8		58.1	58.4

F_2PentOH **E**	exp in CDCl3 +25°C	exp in CDCl3 -50°C	theo in CHCl3 +25°C	theo in CHCl3 -50°C
OH … F_1	1.4	1.7	-1.2	-1.0
OH … F_2	3.5	4.7	-4.9	-5.9
OH – H_2	3.5		1.4	1.2
$H_3 – H_2$	3.0	2.4	1.2	1.0
$H_3 – H_3'$	15.0	15.0	-14.1	-14.1
$H_3 – F_1$	13.7	13.7	14.0	13.3
$H_3 – F_2$	20.0	21.7	16.6	17.6
$H_3' – H_2$	8.6	9.2	9.7	9.8
$H_3' – F_2$a	19.3	21.1	17.7	17.2
$H_3' – F_1$g	13.3	11.6	13.4	14.2

F_2PrOH **G**	exp in CDCl3 +25°C	exp in CDCl3 -50°C	theo in CHCl3 +25°C	theo in CHCl3 -50°C
OH … F_1	0.4		-0.3	-0.2
OH … F_2	0.4		0.1	0.1
H_1 – OH	5.1		4.2	3.6
$H_1 – H_2$	6.0		6.2	5.9
$H_2 – F_1$	17.0		23.0	23.8
$H_2 – F_2$	17.0		10.0	8.5
$H_2 – H_3$	4.6		4.8	5.0
$H_3 – F_1$	57.0		57.9	58.2
$H_3 – F_2$	57.0		56.7	56.8

F_3BuOH **H**	exp in CDCl3 +25°C	exp in CDCl3 -50°C	theo in CHCl3 +25°C	theo in CHCl3 -50°C
OH … F_1	0.7		-4.5	-5.8
OH … F_2	0.7		0.2	0.3
OH … F_3	0.7		0.4	0.3
OH – H_2	4.2		3.7	3.0
H_2 – H_3	4.0		2.2	1.7
H_2 – H_3'	8.0		9.5	9.7
H_3 – F	11.2		10.6	10.7
H_3' – F	10.8		9.5	9.6

F_3PrOH **I**	exp in CDCl3 +25°C	exp in CDCl3 -50°C	theo in CHCl3 +25°C	theo in CHCl3 -50°C
OH … F_1	0.3		1.3	1.3
OH … F_2	0.3		0.0	0.0
OH … F_3	0.3		-1.5	-1.9
F – H_2	10.8		9.9	9.9
OH – H_1	5.8		5.0	5.0

SUPPORTING INFORMATION 2

NMR investigations of the fluorohydrins **A-I**

Table of Contents

5 Detailed NMR analysis of all fluorohydrins 38
5.1 Experimental procedure for sprectra acquisition and FID reprocessing 38
5.2 (±)-*syn*-4-Fluoropentan-2-ol (*syn*-A) 39
5.2.1 ^{1}H NMR spectrum ($CDCl_3$, 500 MHz, 25 °C) 39
5.2.2 Detail of ^{1}H and ^{1}H[^{19}F] NMR of OH, H-3 and H-3' signals ($CDCl_3$, 500 MHz, 25 °C) (*syn*-A) 40
5.2.3 Comparison of ^{1}H NMR of OH, H-3 and H-3' signals at 25 °C and -50 °C ($CDCl_3$, 500 MHz) (*syn*-A) 41
5.2.4 Detail of ^{1}H and ^{1}H[^{19}F] NMR of OH, H-3 and H-3' signals at -50 °C ($CDCl_3$, 500 MHz) (*syn*-A) 42
5.2.5 ^{19}F NMR spectrum ($CDCl_3$, 470 MHz, 25 °C) (*syn*-A) 43
5.2.6 ^{19}F NMR spectrum ($CDCl_3$, 470 MHz, –50 °C) (*syn*-A) 44
5.3 (±)-*anti*-4-fluoropentan-2-ol (*anti*-A) 45
5.3.1 ^{1}H NMR spectrum ($CDCl_3$, 500 MHz, 25 °C) 45
5.3.2 Detail of ^{1}H and ^{1}H[^{19}F] NMR of OH, H-3 and H-3' signals ($CDCl_3$, 500 MHz, 25 °C) (*anti*-A) 46
5.3.3 Comparison of ^{1}H NMR of OH, H-3 and H-3' signals at 25 °C and -50 °C ($CDCl_3$, 500 MHz) (*anti*-A) 47
5.3.4 Detail of ^{1}H and ^{1}H[^{19}F] NMR of OH, H-3 and H-3' signals at -50 °C ($CDCl_3$, 500 MHz) (*anti*-A) 48
5.3.5 ^{19}F NMR spectrum ($CDCl_3$, 470 MHz, 25 °C) (*anti*-A) 49
5.3.6 ^{19}F NMR spectrum ($CDCl_3$, 470 MHz, –50 °C) (*anti*-A) 50
5.4 4-fluorobutan-2-ol (B) 51
5.4.1 ^{1}H NMR spectrum ($CDCl_3$, 500 MHz, 25 °C) 51
5.4.2 Detail of ^{1}H and ^{1}H[^{19}F] NMR of OH, H-3 and H-3' signals ($CDCl_3$, 500 MHz, 25 °C) (B) 52
5.4.3 ^{19}F NMR spectrum ($CDCl_3$, 470 MHz, 25 °C) (B) 53
5.5 3-fluoro-2,2-dimethylpropan-1-ol (C) 54
5.5.1 ^{1}H NMR spectrum ($CDCl_3$, 500 MHz, 25 °C) 54
5.5.2 Detail of ^{1}H and ^{1}H[^{19}F] NMR of OH signal ($CDCl_3$, 500 MHz, 25 °C) l (C) 55

5.5.3 ^{19}F NMR spectrum ($CDCl_3$, 470 MHz, 25 °C) (C) 56
5.6 3-fluoropropan-1-ol (D) 57
5.6.1 ^{1}H NMR spectrum ($CDCl_3$, 500 MHz, 25 °C) 57
5.6.2 Detail of ^{1}H and ^{1}H[^{19}F] NMR of OH signal ($CDCl_3$, 500 MHz, 25 °C) (D) 58
5.6.3 Comparison of ^{1}H NMR of OH signal at 25 °C and -50 °C ($CDCl_3$, 500 MHz) (D) 59
5.6.4 Detail of ^{1}H and ^{1}H[^{19}F] NMR of OH signals at -50 °C ($CDCl_3$, 500 MHz) (D) 60
5.6.5 ^{19}F NMR spectrum ($CDCl_3$, 470 MHz, 25 °C) (D) 61
5.6.6 ^{19}F NMR spectrum ($CDCl_3$, 470 MHz, –50 °C) (D) 62
5.6.7 ^{1}H NMR spectrum (CD_2Cl_2, 500 MHz, 25 °C) (D) 63
5.6.8 Detail of ^{1}H and ^{1}H[^{19}F] NMR of OH signal (CD_2Cl_2, 500 MHz, 25 °C) (D) 64
5.6.9 ^{19}F NMR spectrum (CD_2Cl_2, 470 MHz, 25 °C) (D) 65
5.7 4,4-difluoropentan-2-ol (E) 66
5.7.1 ^{1}H NMR spectrum ($CDCl_3$, 500 MHz, 25 °C) 66
5.7.2 Detail of ^{1}H and ^{1}H[^{19}F] NMR of OH, H-3 and H-3' signals ($CDCl_3$, 500 MHz, 25 °C) (E) 67
5.7.3 Homodecoupling of H-2 ($CDCl_3$, 500 MHz, 25 °C) (E) 68
5.7.4 Comparison of ^{1}H NMR of OH, H-3' and H-3 signal at 25 °C and -50 °C ($CDCl_3$, 500 MHz) (E) 69
5.7.5 Detail of ^{1}H and ^{1}H[^{19}F] NMR of OH, H-3' and H-3 signals at -50 °C ($CDCl_3$, 500 MHz) (E) 70
5.7.6 Homodecoupling of H-2 ($CDCl_3$, 500 MHz, -50 °C) (E) 71
5.7.7 ^{19}F NMR spectrum ($CDCl_3$, 470 MHz, 25 °C) (E) 72
5.7.8 ^{19}F NMR spectrum ($CDCl_3$, 470 MHz, –50 °C) (E) 73
5.8 4,4-difluorobutan-2-ol (F) 74
5.8.1 ^{1}H NMR spectrum ($CDCl_3$, 500 MHz, 25 °C) 74
5.8.2 Detail of ^{1}H and ^{1}H[^{19}F] NMR of OH, H-3 and H-3' signals ($CDCl_3$, 500 MHz, 25 °C) (F) 75
5.8.3 Detail of ^{1}H and ^{1}H[^{19}F] NMR of OH signal ($CDCl_3$, 500 MHz, 25 °C) (F) 76
5.8.4 ^{19}F NMR spectrum ($CDCl_3$, 470 MHz, 25 °C) (F) 77
5.9 3,3-difluoropropan-1-ol (G) 78
5.9.1 ^{1}H NMR spectrum ($CDCl_3$, 500 MHz, 25 °C) 78
5.9.2 Detail of ^{1}H and ^{1}H[^{19}F] NMR of OH signal ($CDCl_3$, 500 MHz, 25 °C) (G) 79
5.9.3 Detail of ^{1}H (enhanced resolution spectra) and ^{1}H[^{19}F] NMR of OH signal ($CDCl_3$, 500 MHz, 25 °C) (G) 80
5.9.4 ^{19}F NMR spectrum ($CDCl_3$, 470 MHz, 25 °C) (G) 81
5.10 4,4,4-trifluorobutan-2-ol (H) 82
5.10.1 ^{1}H NMR spectrum ($CDCl_3$, 500 MHz, 25 °C) 82
5.10.2 Detail of ^{1}H and ^{1}H[^{19}F] NMR of OH, H-3 and H-3' signals ($CDCl_3$, 500 MHz, 25 °C) (H) 83
5.10.3 Detail of enhanced resolution spectra of ^{1}H and ^{1}H[^{19}F] NMR of OH signal ($CDCl_3$, 500 MHz, 25 °C) (H) 84
5.10.4 ^{19}F NMR spectrum ($CDCl_3$, 470 MHz, 25 °C) (H) 85
5.11 3,3,3-trifluoropropan-1-ol (I) 86
5.11.1 ^{1}H NMR spectrum ($CDCl_3$, 500 MHz, 25 °C) 86
5.11.2 Detail of ^{1}H and ^{1}H[^{19}F] NMR of OH signal ($CDCl_3$, 500 MHz, 25 °C) (I) 87

5.11.3 Detail of ^{1}H (enhanced resolution spectra) and $^{1}H[^{19}F]$ NMR of OH signal ($CDCl_3$, 500 MHz, 25 °C) (I) 88
5.11.4 ^{19}F NMR spectrum ($CDCl_3$, 470 MHz, 25 °C) (I)....89

5 Detailed NMR analysis of all fluorohydrins

5.1 Experimental procedure for sample preparation, sprectra acquisition and FID reprocessing

Molecular sieves 3Å (2g, beads 4-8 mesh) were activated by heating at 160 °C under reduced pressure (0.15 mmHg) for 16 hours. After cooling, the activated molecular sieves (3 spatulas) were placed under argon atmosphere and then added to a dry solution of fluorohydrin (**A-I**) in $CDCl_3$ (1.2 mg/mL, (0.009M-0.015M)). The resulting solution was left standing for 40 minutes under argon and then 0.6 mL of the supernatant solution was added to the NMR tube which was previously flame-dried under reduced pressure and placed under argon. The NMR tube was closed with a valve and immediately submitted for data acquisition.

NMR data were collected on a Bruker AVIII HD 500 MHz NMR spectrometer. Samples were shimmed until $w_{½}$ for residual $CDCl_3$ solvent signal was 0.5 Hz or better through a combination of sequential iterations of “TopShim” gradient shimming with additional manual intervention as required. ^{1}H spectra were collected with TD = 131,072 points (zero-filled to 262,144) and SW = 14 ppm (o1p = 5.0 ppm). ^{19}F spectra were collected with TD = 262,144 points (zero-filled to 524,288) and SW = 50 ppm (o1p proximal to ^{19}F signal). ^{19}F(^{1}H) spectra were collected with TD = 262,144 points (zero-filled to 524,288) and SW = 200 ppm (o1p proximal to ^{19}F signal; inverse-gated decoupling with o2p = 5.0 ppm). ^{1}H(^{19}F) spectra were collected with TD = 65,536 points (zero-filled to 196,608) and SW = 14 ppm (o1p = 5.0 ppm; adiabatic inverse-gated decoupling with o2p proximal to ^{19}F signal).

5.2 (±)-*syn*-4-Fluoropentan-2-ol (*syn*-A)

5.2.1 ^{1}H NMR spectrum ($CDCl_3$, 500 MHz, 25 °C)

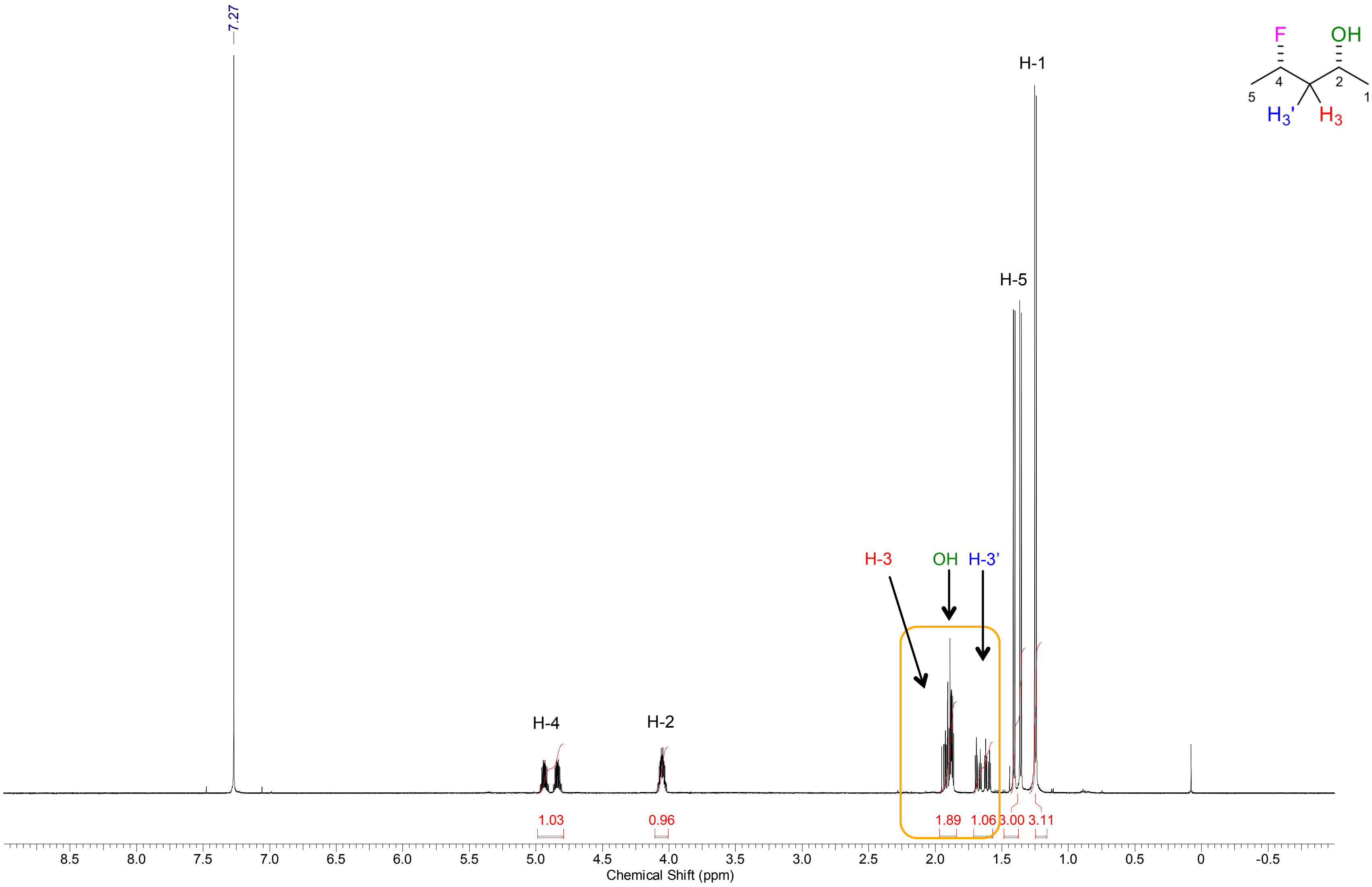

5.2.2 Detail of ^{1}H and $^{1}H[^{19}F]$ NMR of OH, H-3 and H-3' signals ($CDCl_3$, 500 MHz, 25 °C) (*syn*-A)

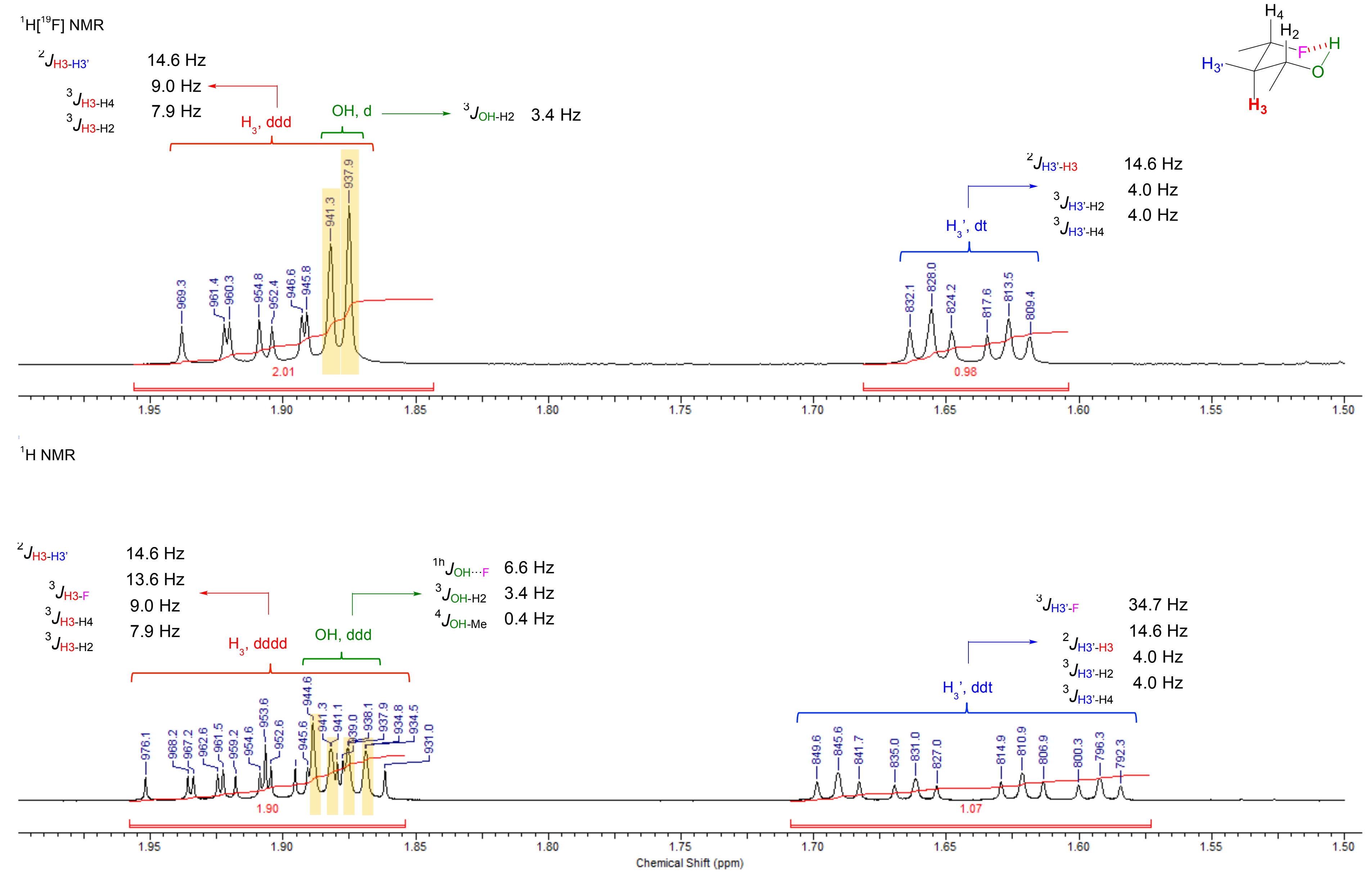

5.2.3 Comparison of ^{1}H NMR of OH, H-3 and H-3' signals at 25 °C and -50 °C ($CDCl_3$, 500 MHz) (*syn*-A)

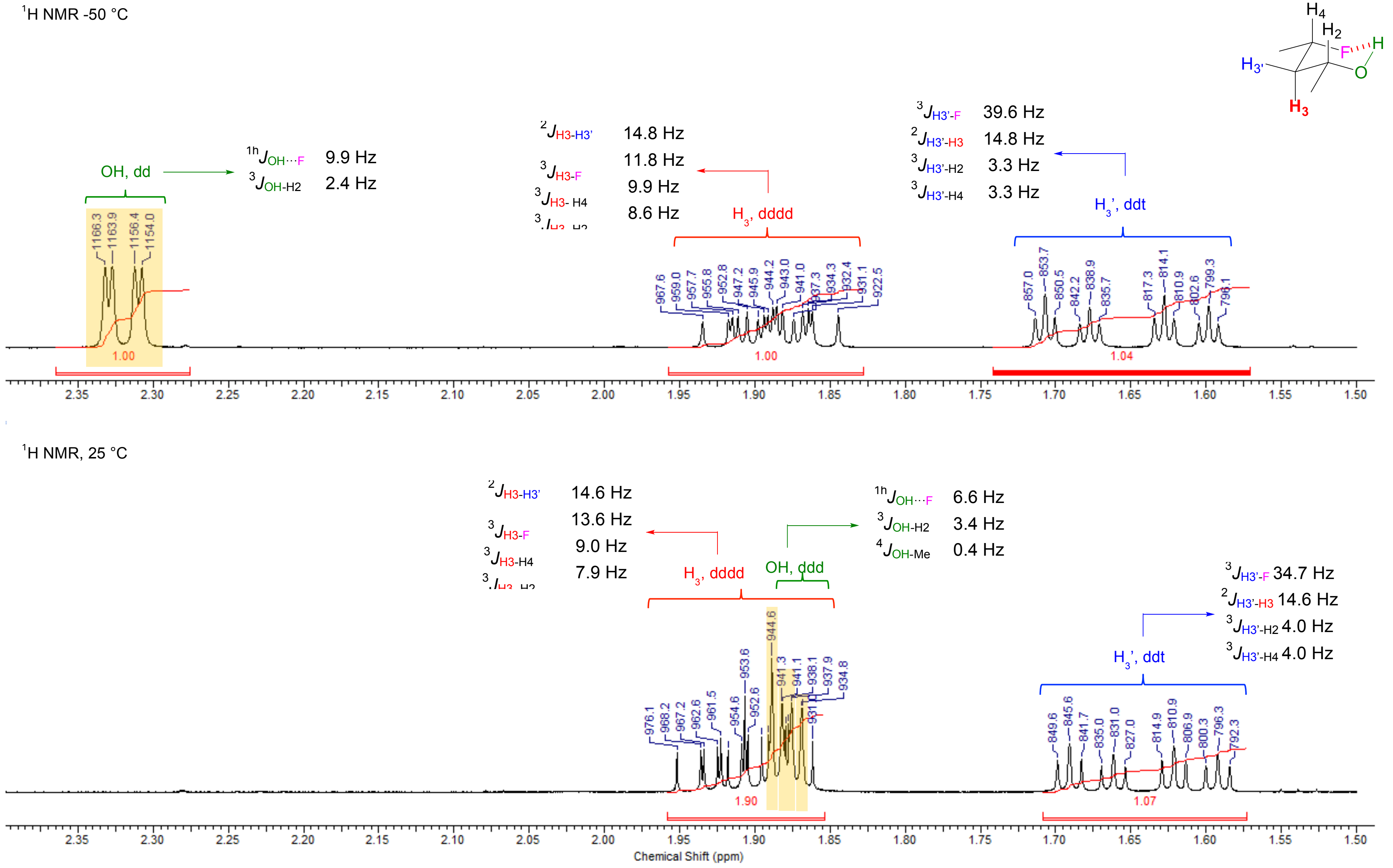

5.2.4 Detail of ^{1}H and ^{1}H[^{19}F] NMR of OH, H-3 and H-3' signals at -50 °C ($CDCl_3$, 500 MHz) (*syn*-A)

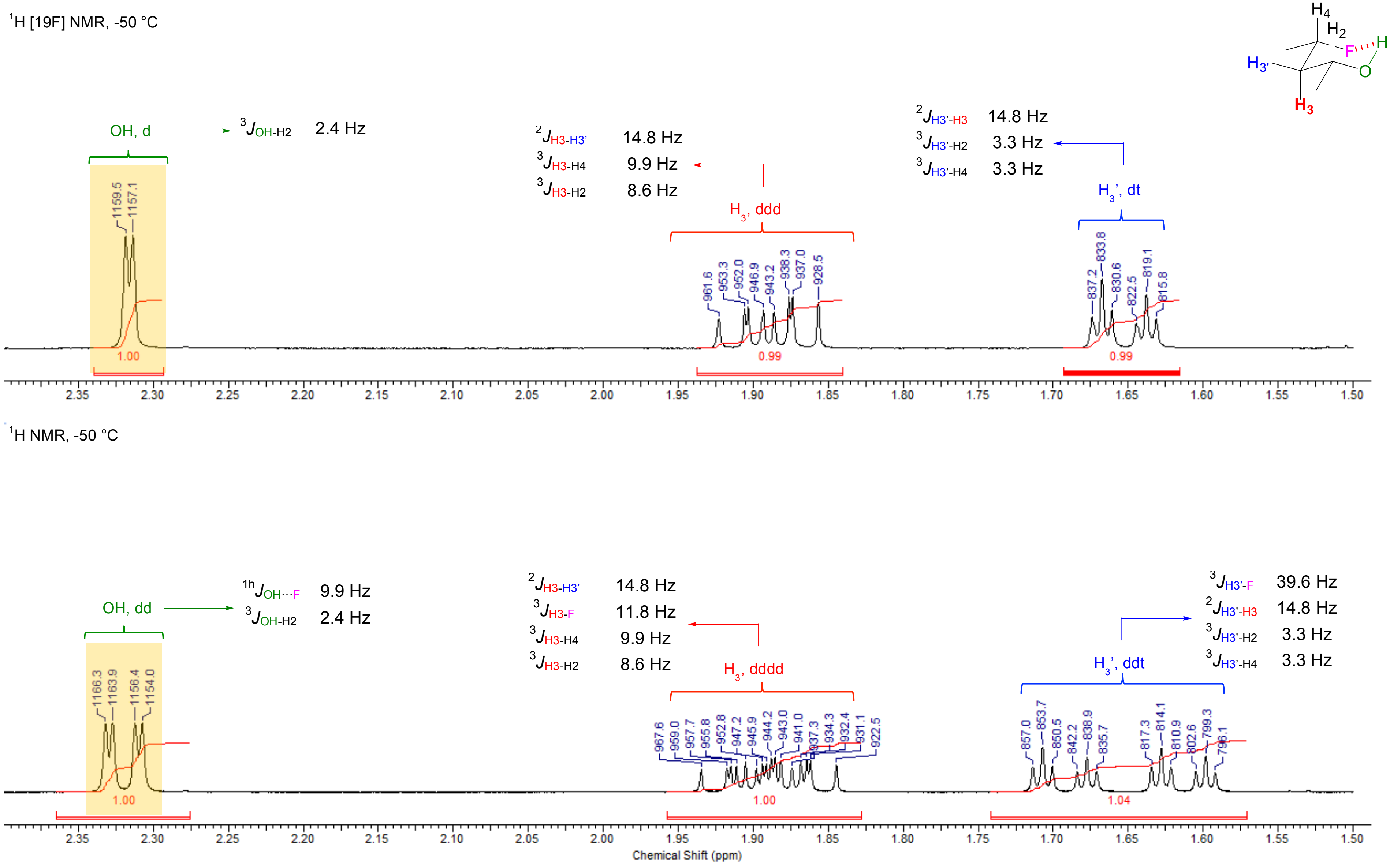

5.2.5 ^{19}F NMR spectrum ($CDCl_3$, 470 MHz, 25 °C) (*syn*-A)

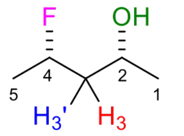

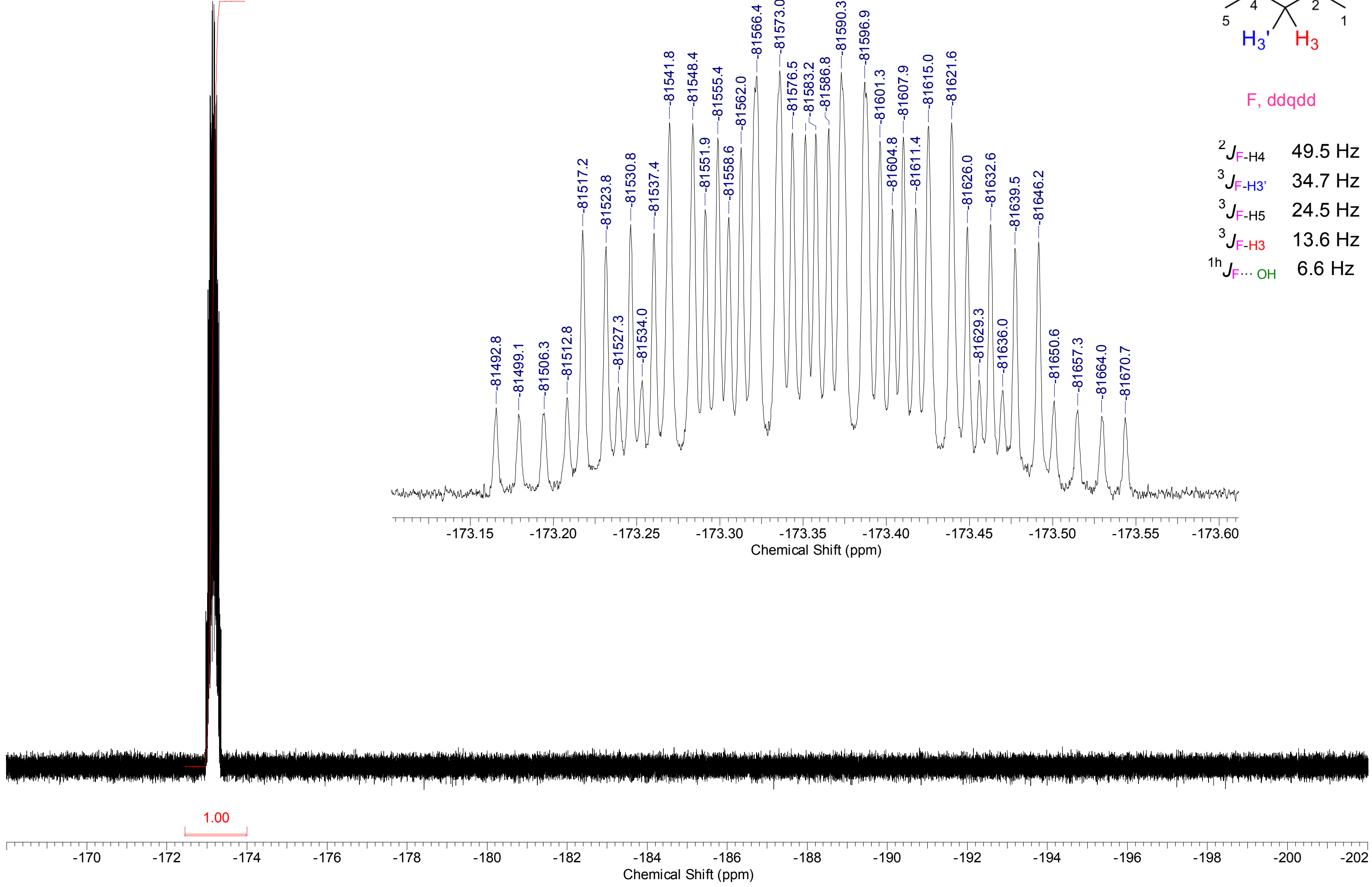

5.2.6 ^{19}F NMR spectrum (CDCl$_3$, 470 MHz, –50 °C) (*syn*-A)

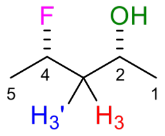

F, ddqdd

$^{2}J_{F\text{-}H4}$ 49.8 Hz
$^{3}J_{F\text{-}H3'}$ 39.6 Hz
$^{3}J_{F\text{-}H5}$ 25.2 Hz
$^{3}J_{F\text{-}H3}$ 11.8 Hz
$^{1h}J_{F\cdots OH}$ 9.9 Hz

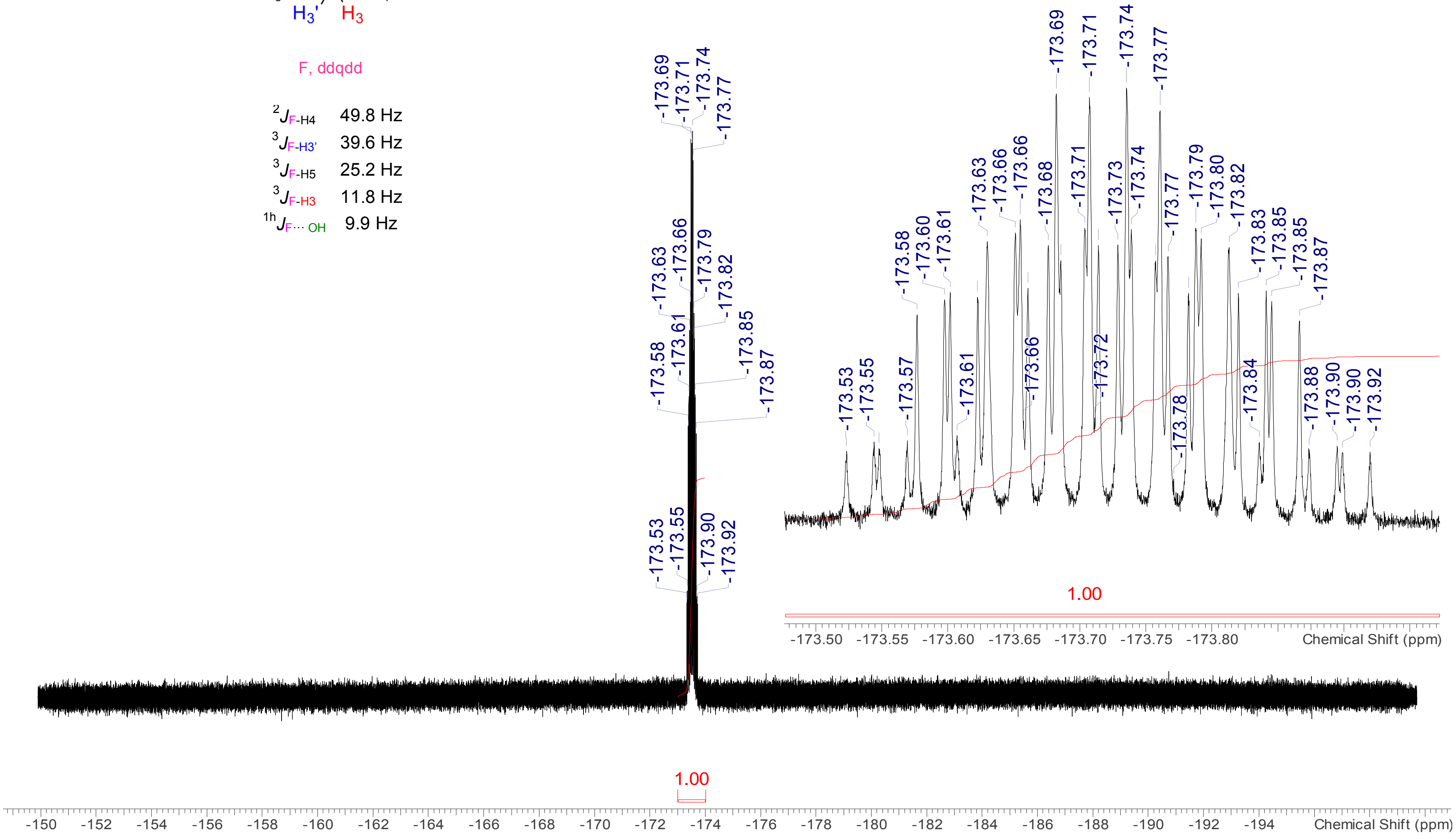

5.3 (±)-*anti*-4-fluoropentan-2-ol (*anti*-A)

5.3.1 ^{1}H NMR spectrum (CDCl$_3$, 500 MHz, 25 °C)

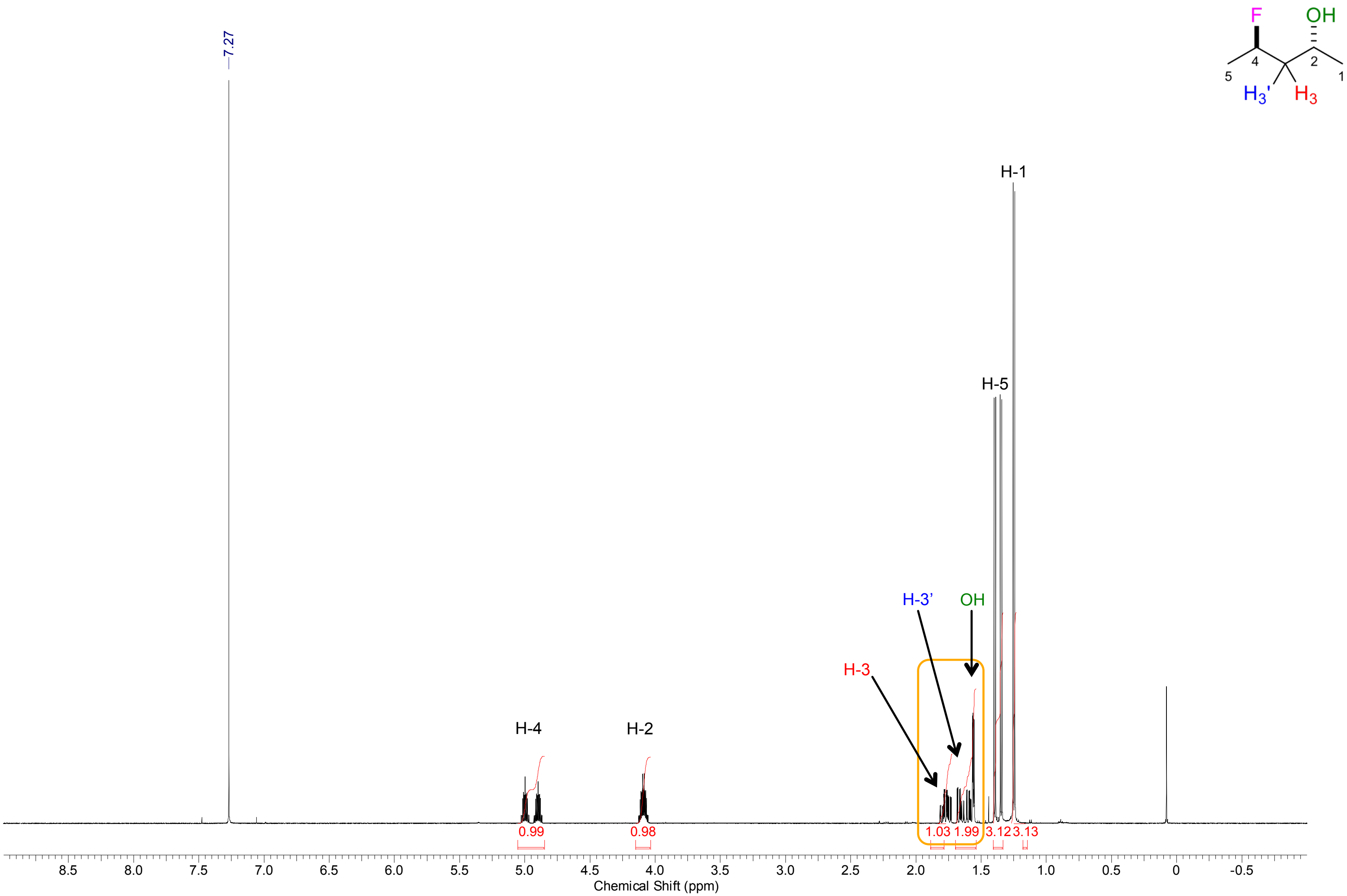

5.3.2 Detail of ^{1}H and ^{1}H[^{19}F] NMR of OH, H-3 and H-3' signals ($CDCl_3$, 500 MHz, 25 °C) (*anti*-A)

^{1}H[^{19}F] NMR

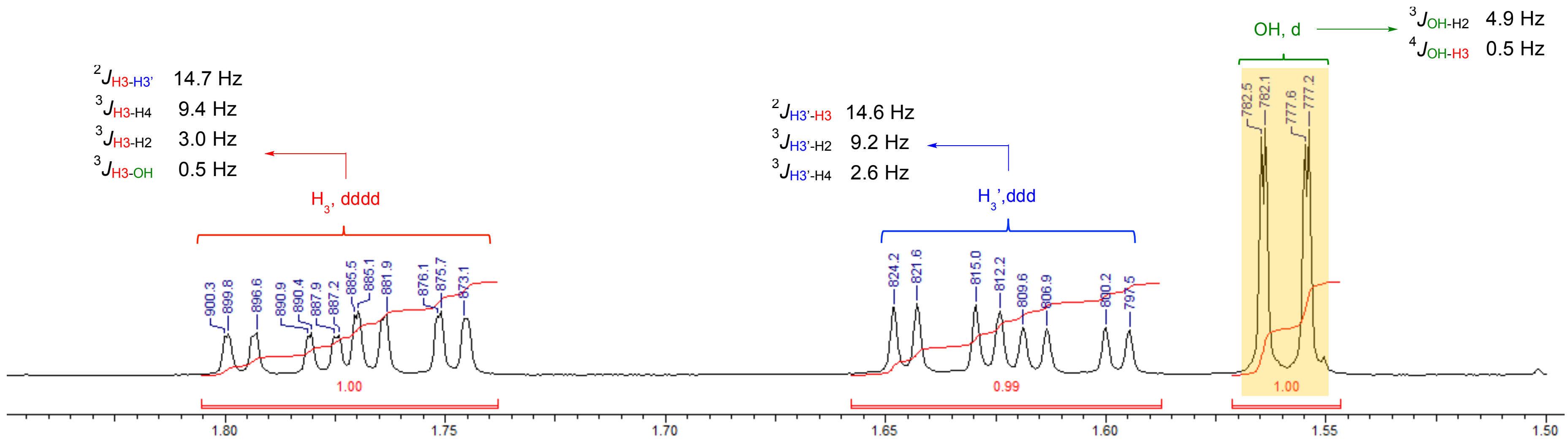

^{1}H NMR

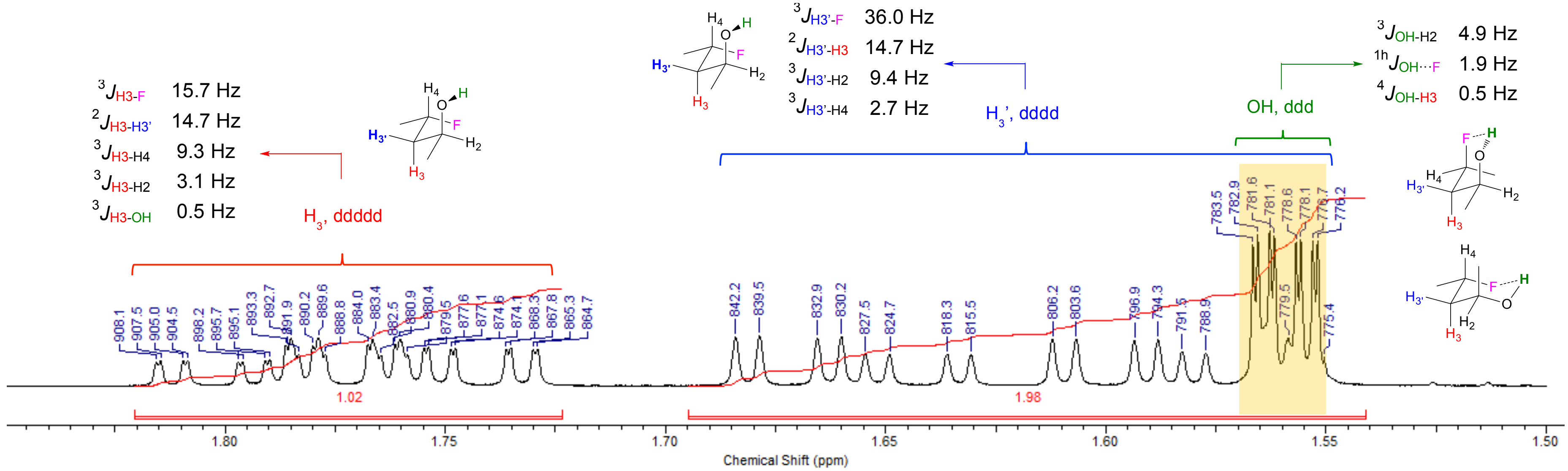

5.3.3 Comparison of ^{1}H NMR of OH, H-3 and H-3' signals at 25 °C and -50 °C ($CDCl_3$, 500 MHz) (*anti*-A)

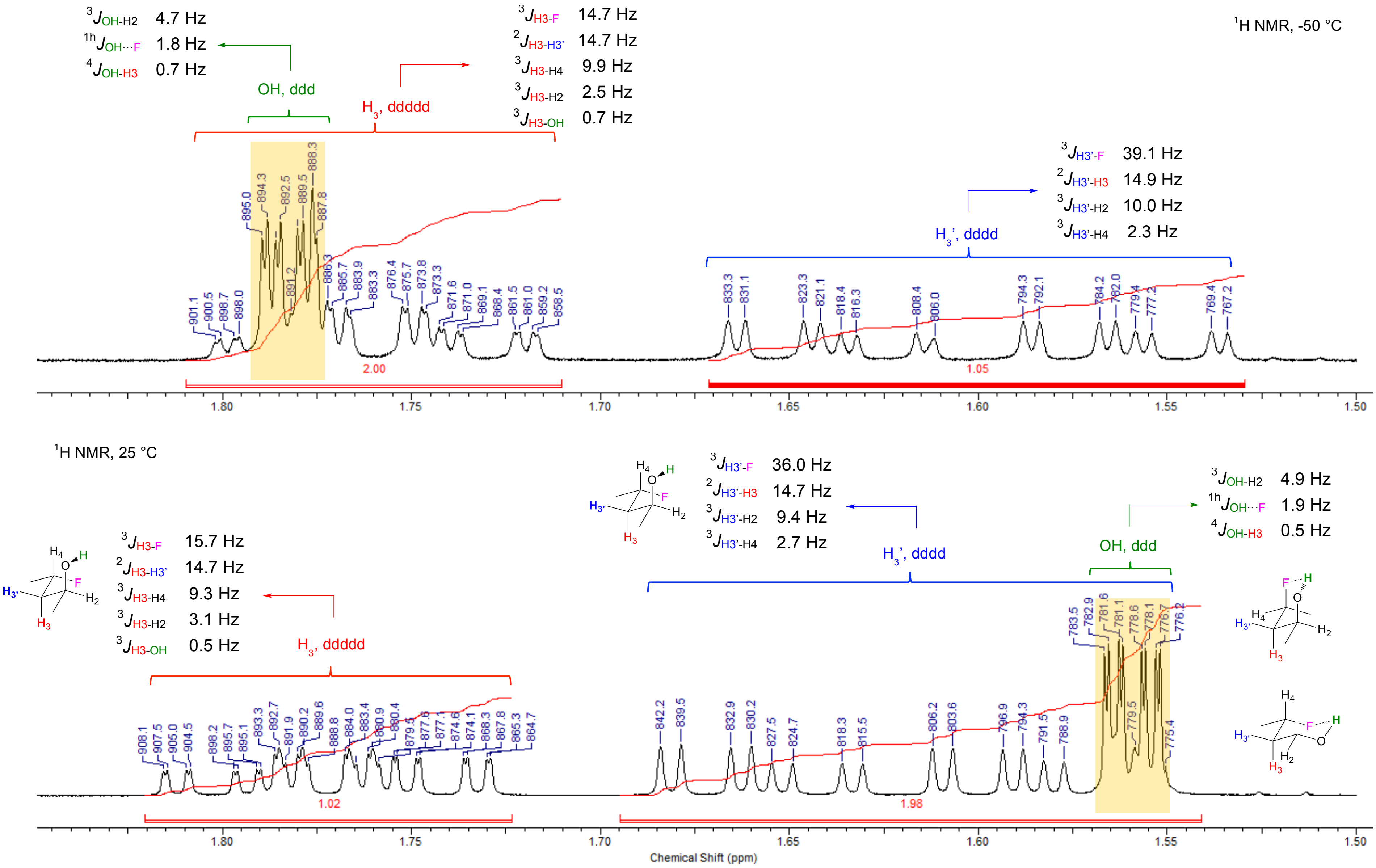

5.3.4 Detail of ^{1}H and ^{1}H[^{19}F] NMR of OH, H-3 and H-3' signals at -50 °C ($CDCl_3$, 500 MHz) (*anti*-A)

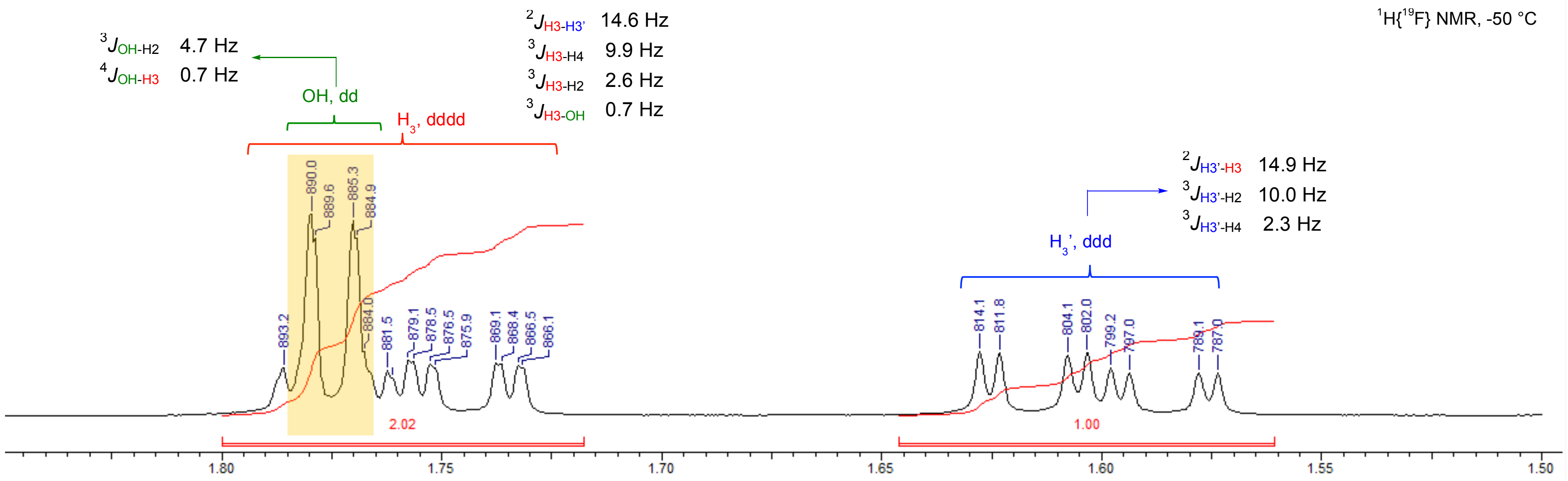

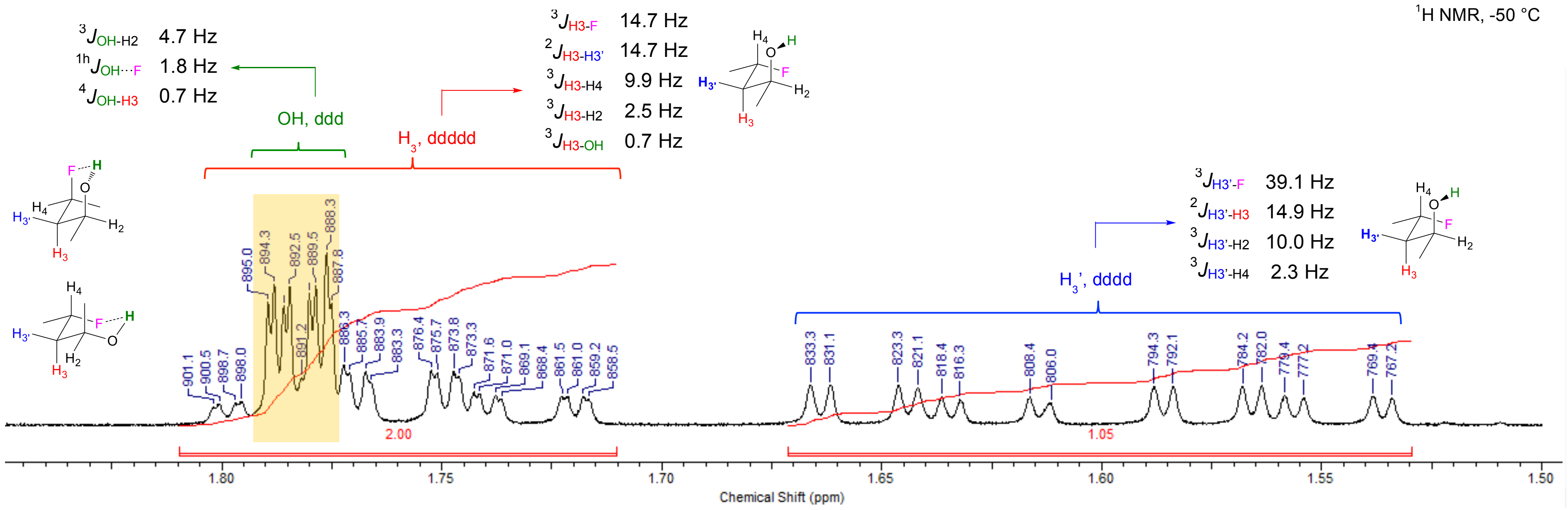

5.3.5 ^{19}F NMR spectrum (CDCl$_3$, 470 MHz, 25 °C) (*anti*-A)

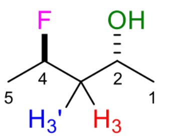

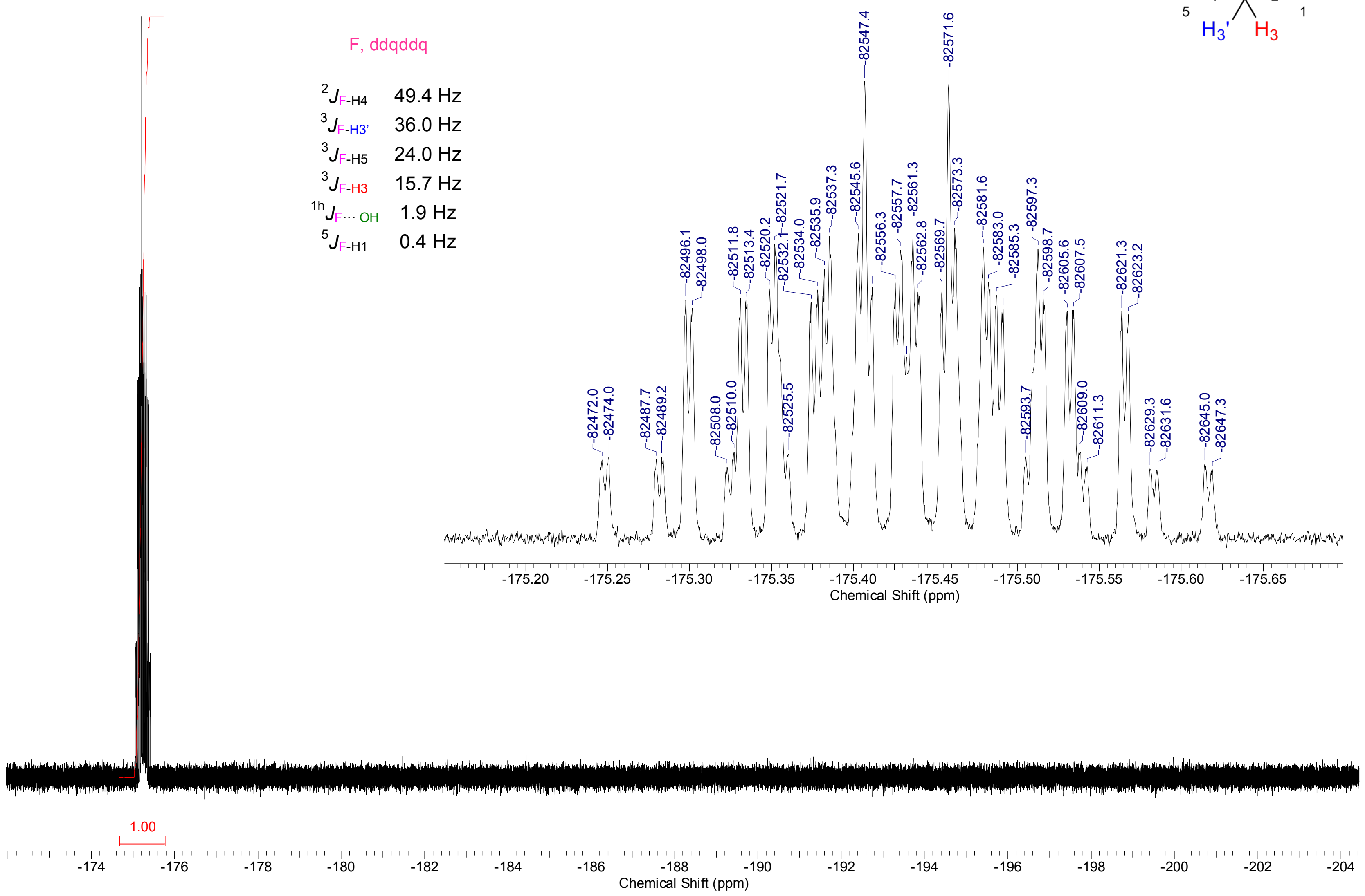

5.3.6 ^{19}F NMR spectrum ($CDCl_3$, 470 MHz, –50 °C) (*anti*-A)

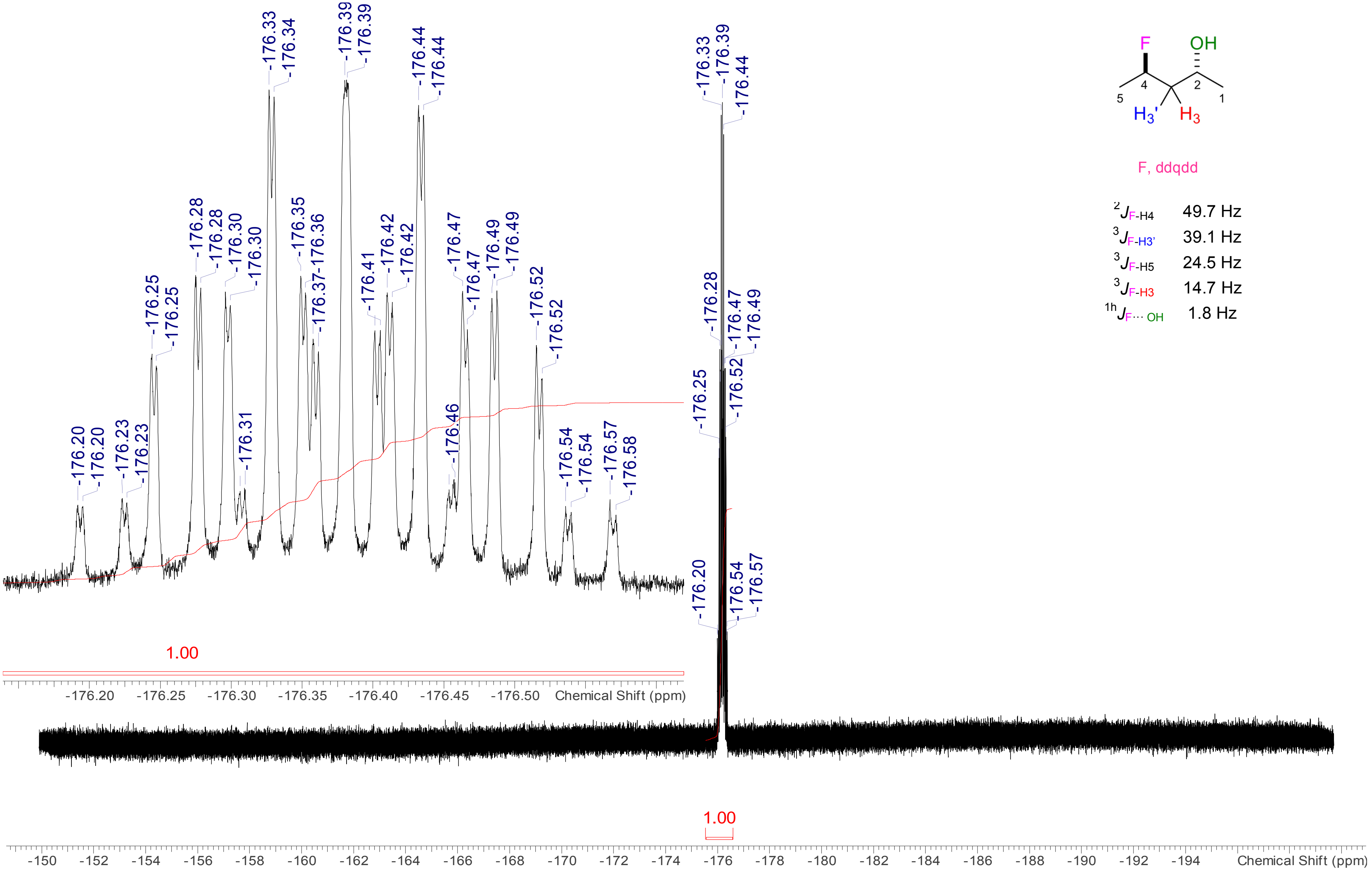

5.4 4-fluorobutan-2-ol (B)

5.4.1 ^{1}H NMR spectrum ($CDCl_3$, 500 MHz, 25 °C)

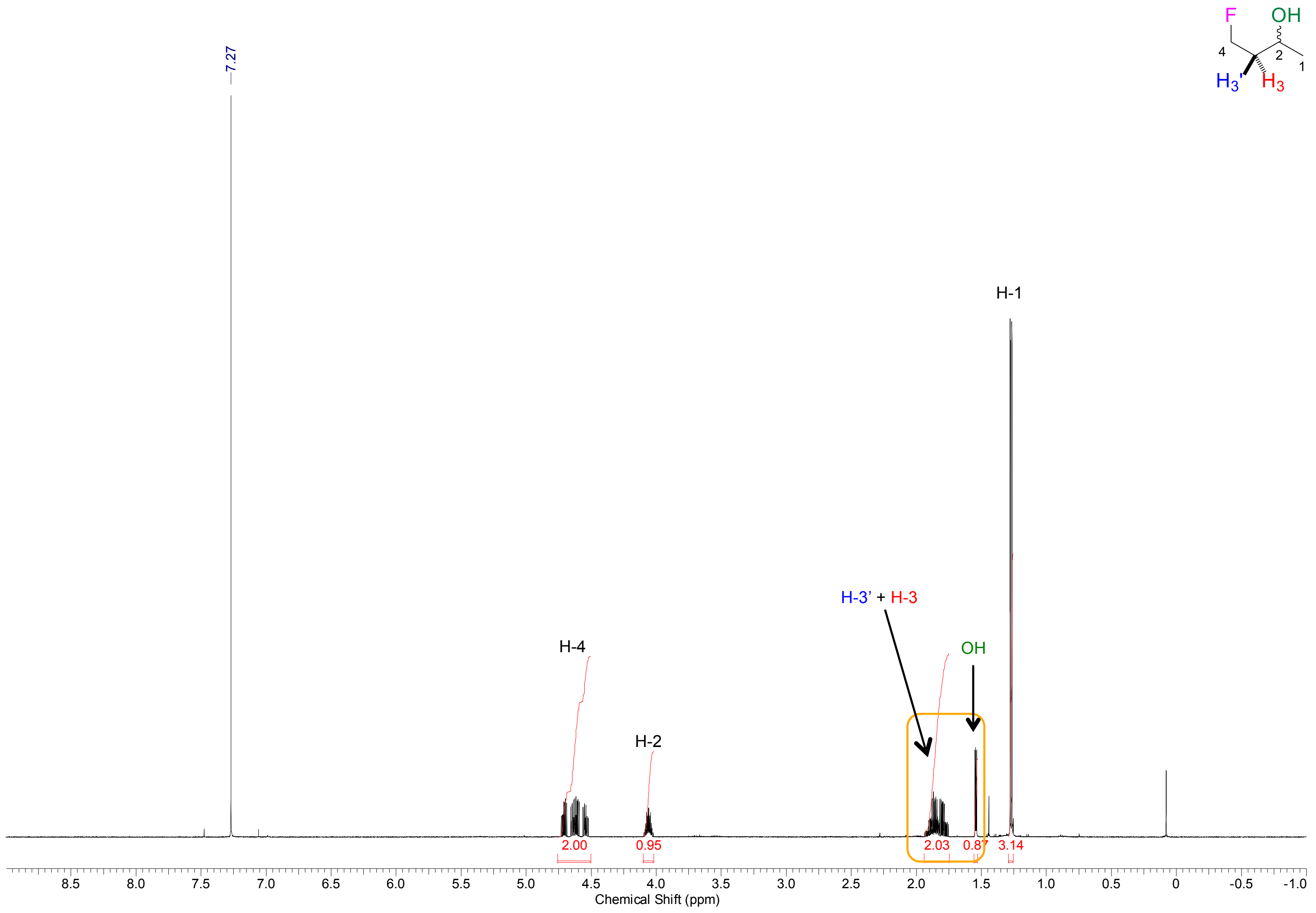

5.4.2 Detail of ^{1}H and ^{1}H[^{19}F] NMR of OH, H-3 and H-3' signals ($CDCl_3$, 500 MHz, 25 °C) (B)

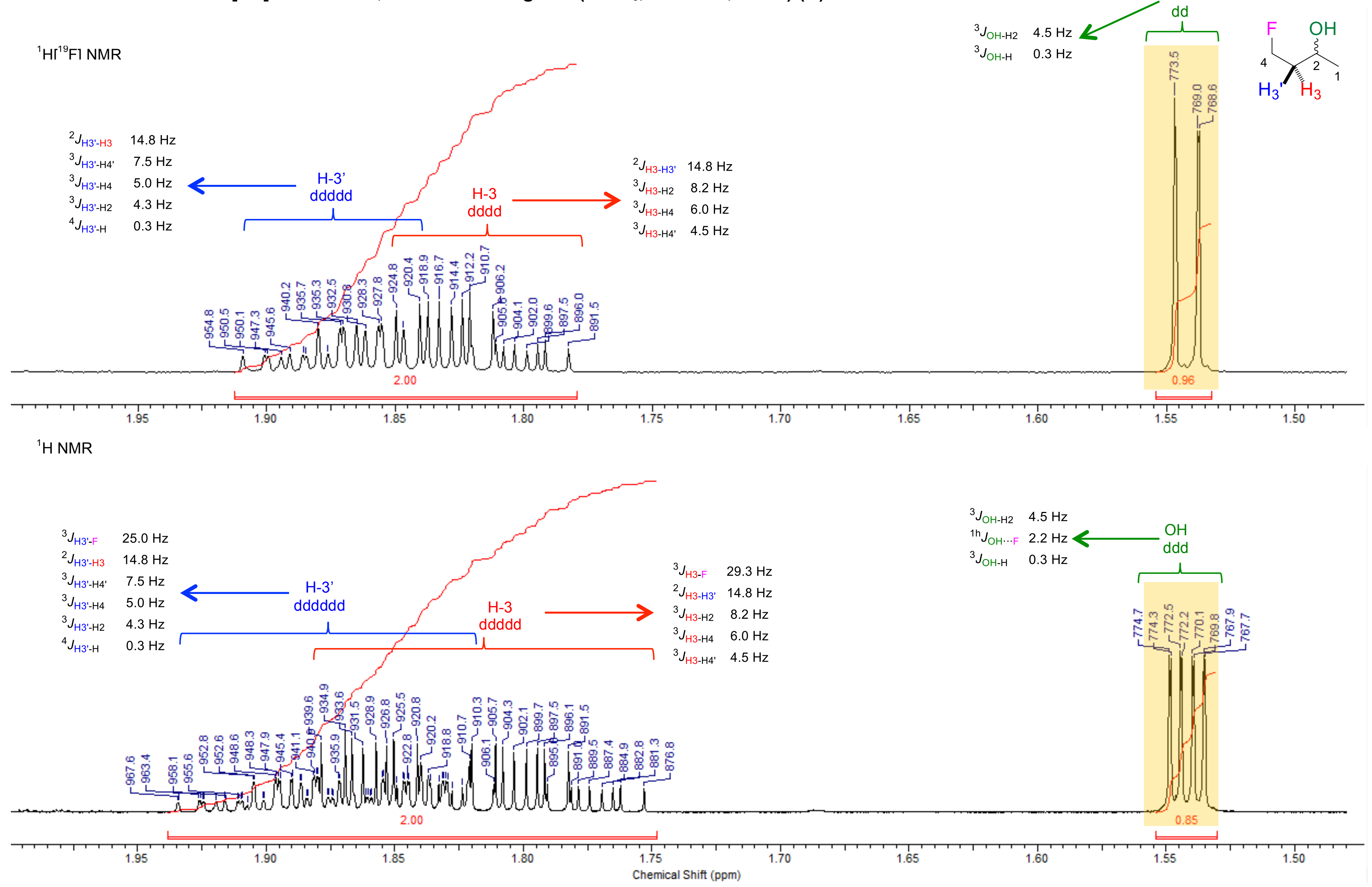

5.4.3 ^{19}F NMR spectrum ($CDCl_3$, 470 MHz, 25 °C) (B)

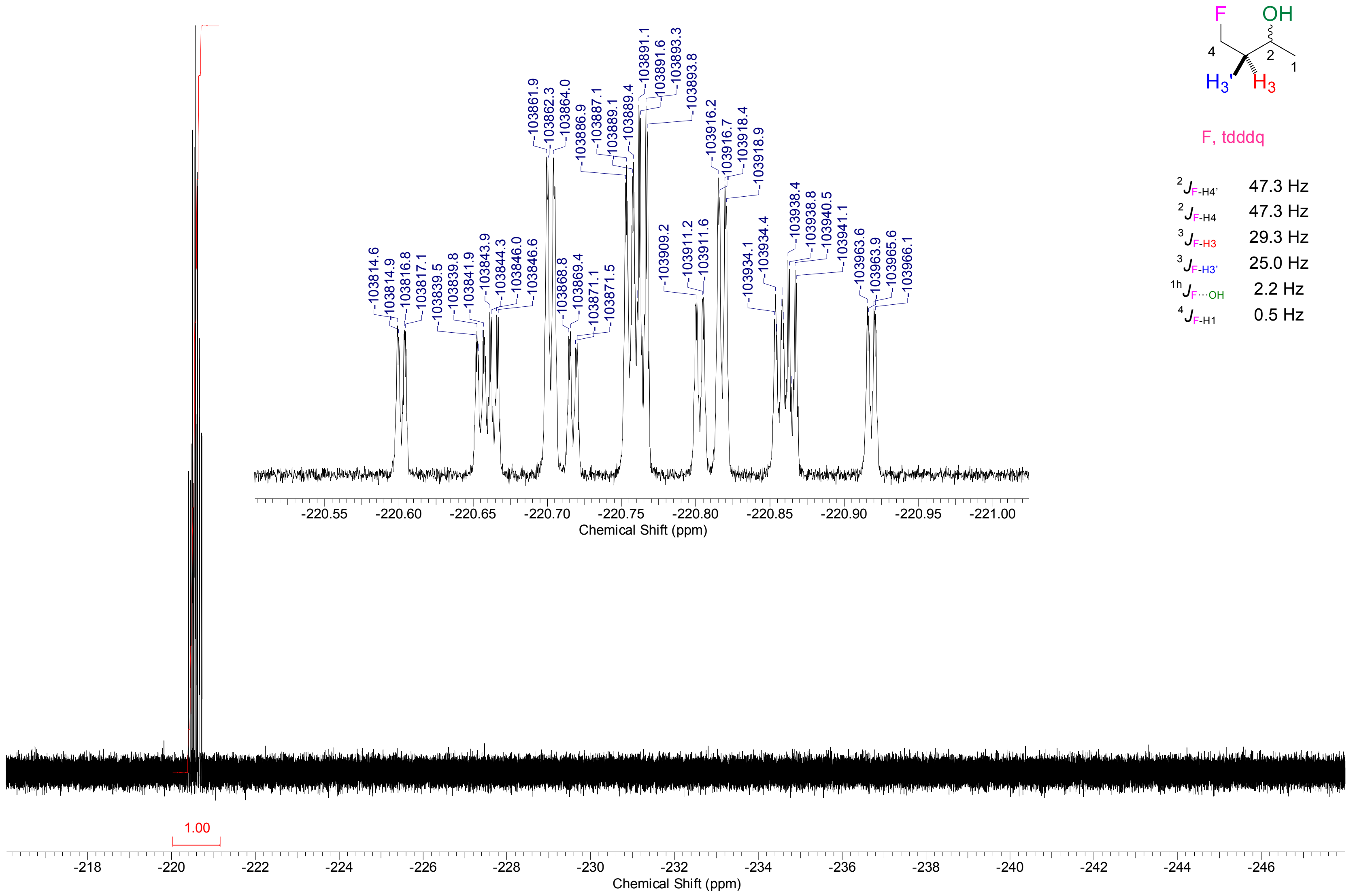

5.5 3-fluoro-2,2-dimethylpropan-1-ol (C)

5.5.1 ^{1}H NMR spectrum (CDCl$_3$, 500 MHz, 25 °C)

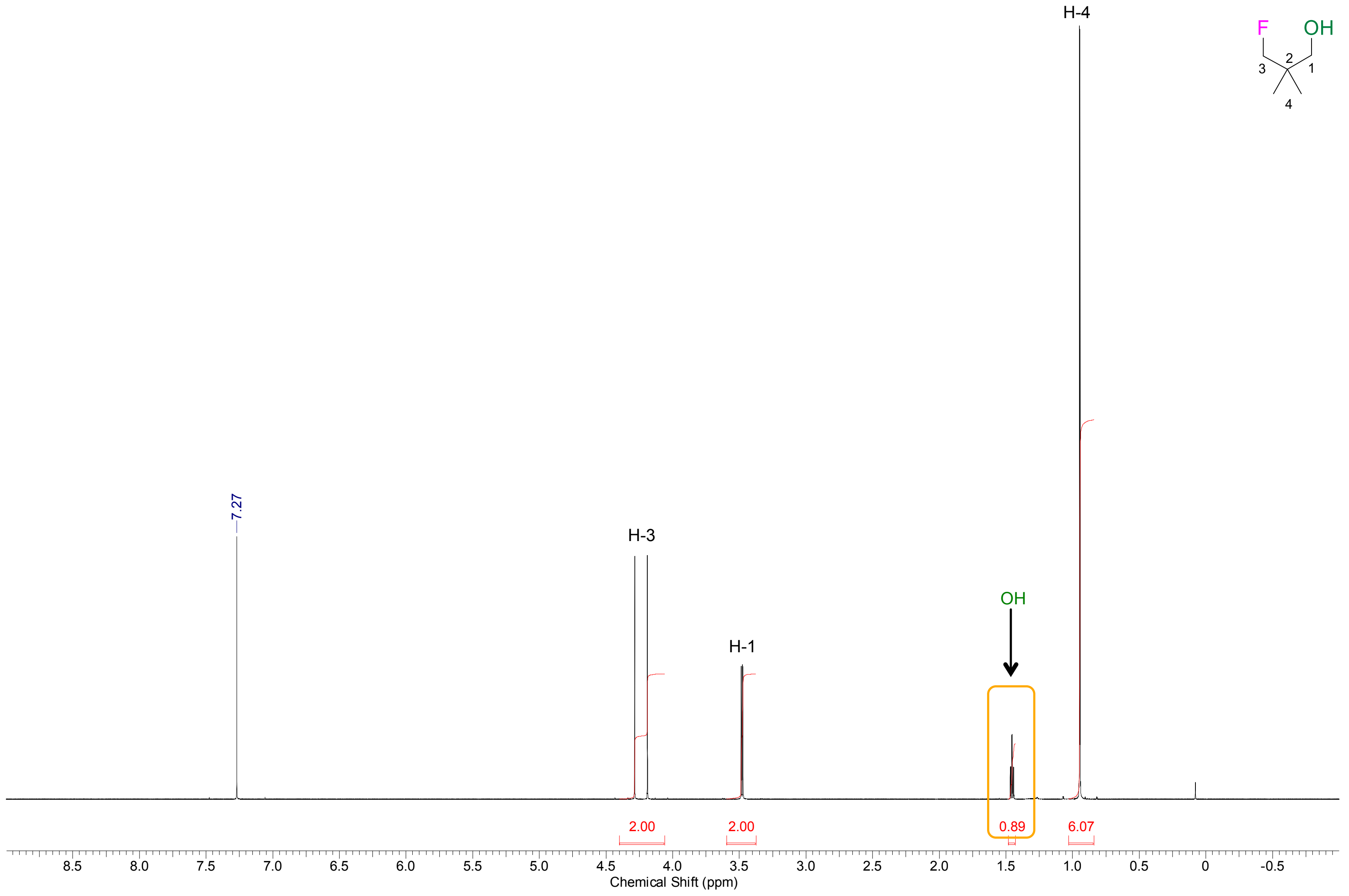

5.5.2 Detail of ^{1}H and $^{1}H[^{19}F]$ NMR of OH signal ($CDCl_3$, 500 MHz, 25 °C) I (C)

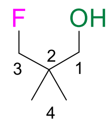

$^{1}H[^{19}F]$ NMR

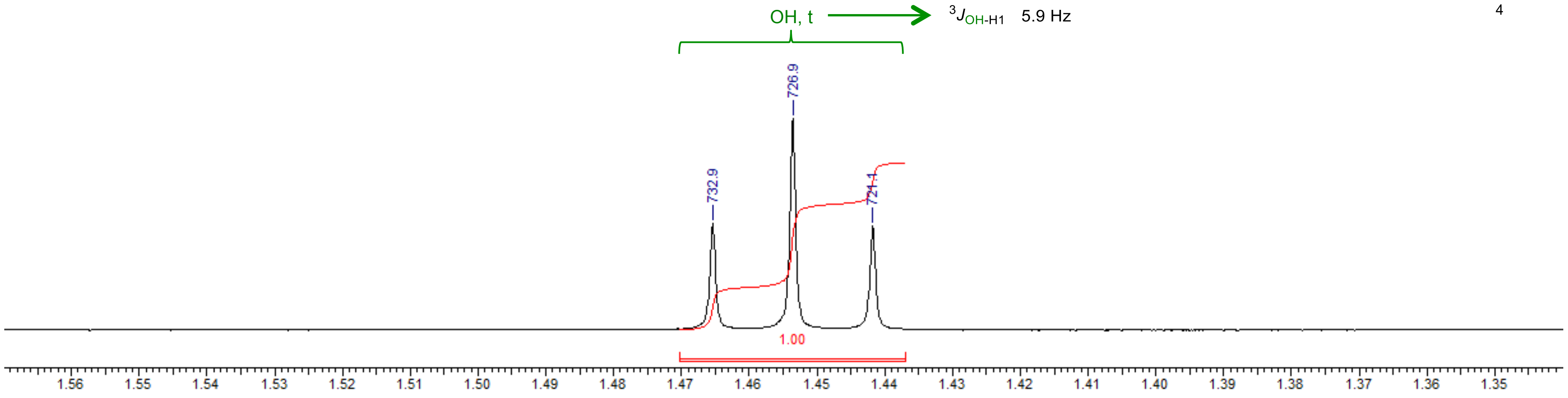

^{1}H NMR

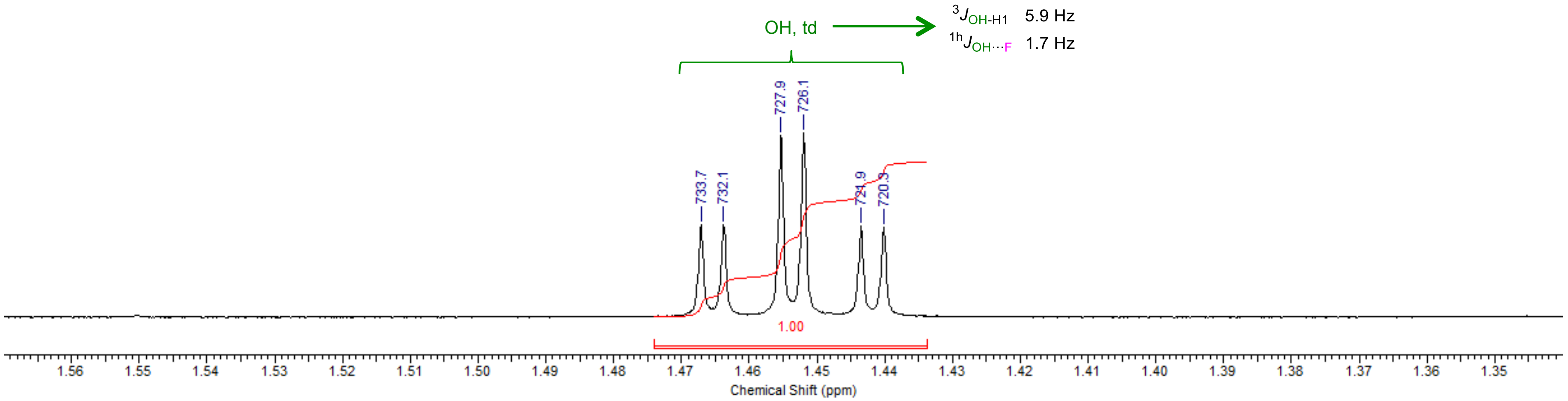

5.5.3 ^{19}F NMR spectrum ($CDCl_3$, 470 MHz, 25 °C) (C)

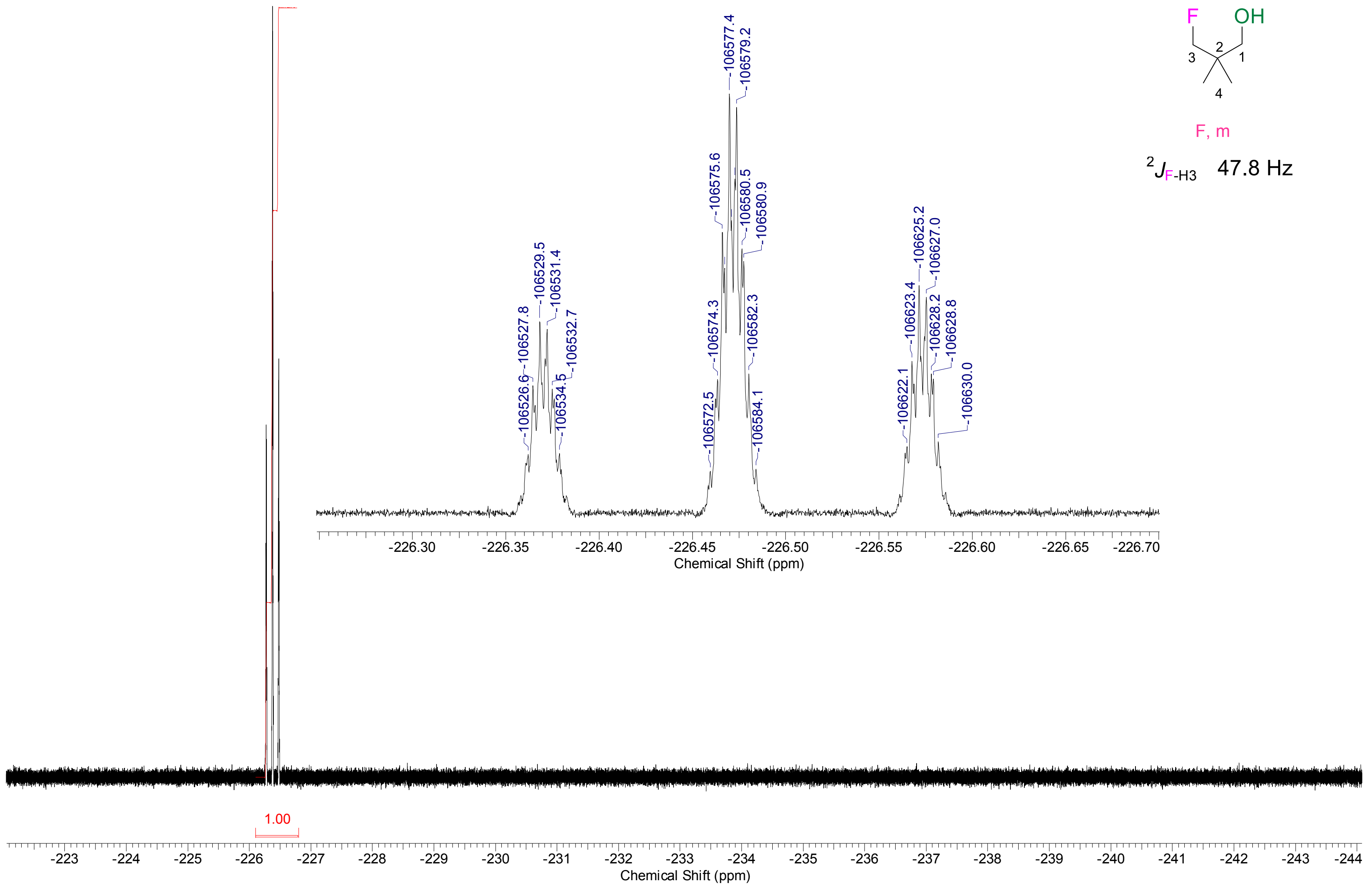

5.6 3-fluoropropan-1-ol (D)

5.6.1 ^{1}H NMR spectrum ($CDCl_3$, 500 MHz, 25 °C)

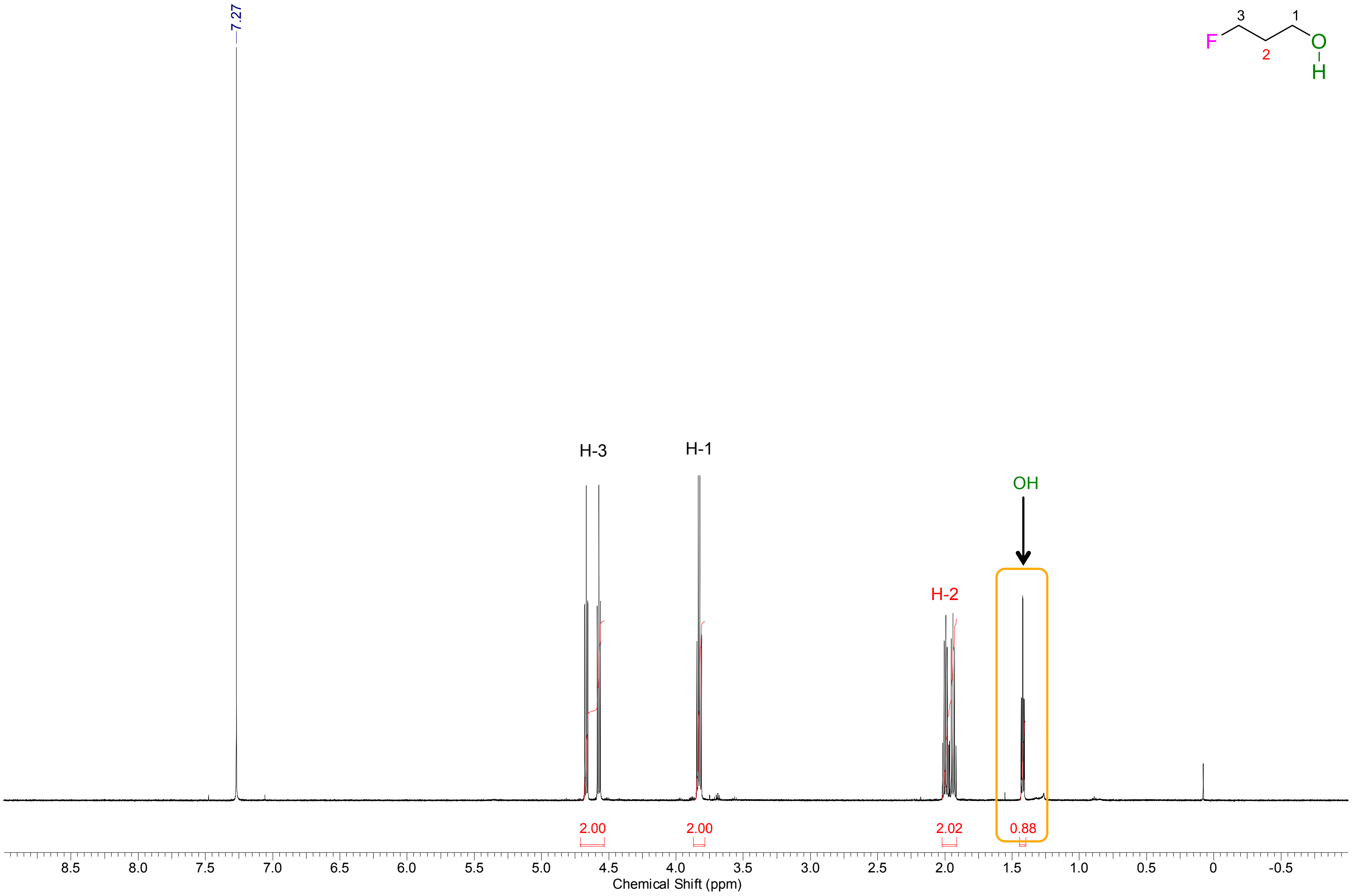

5.6.2 Detail of ^{1}H and ^{1}H[^{19}F] NMR of OH signal ($CDCl_3$, 500 MHz, 25 °C) (D)

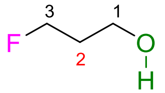

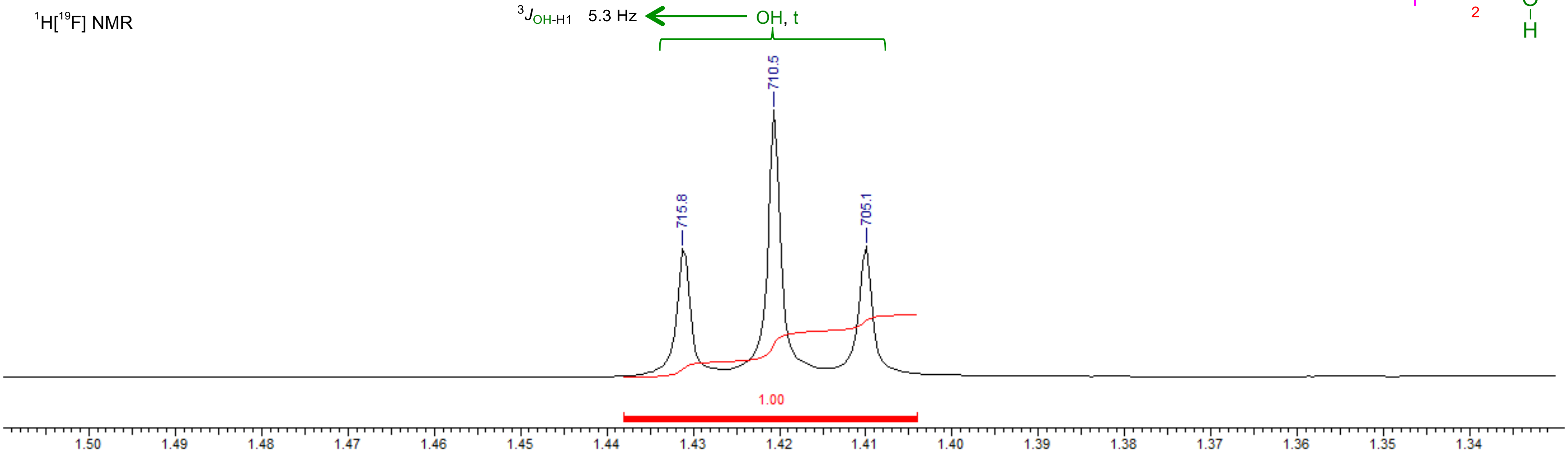

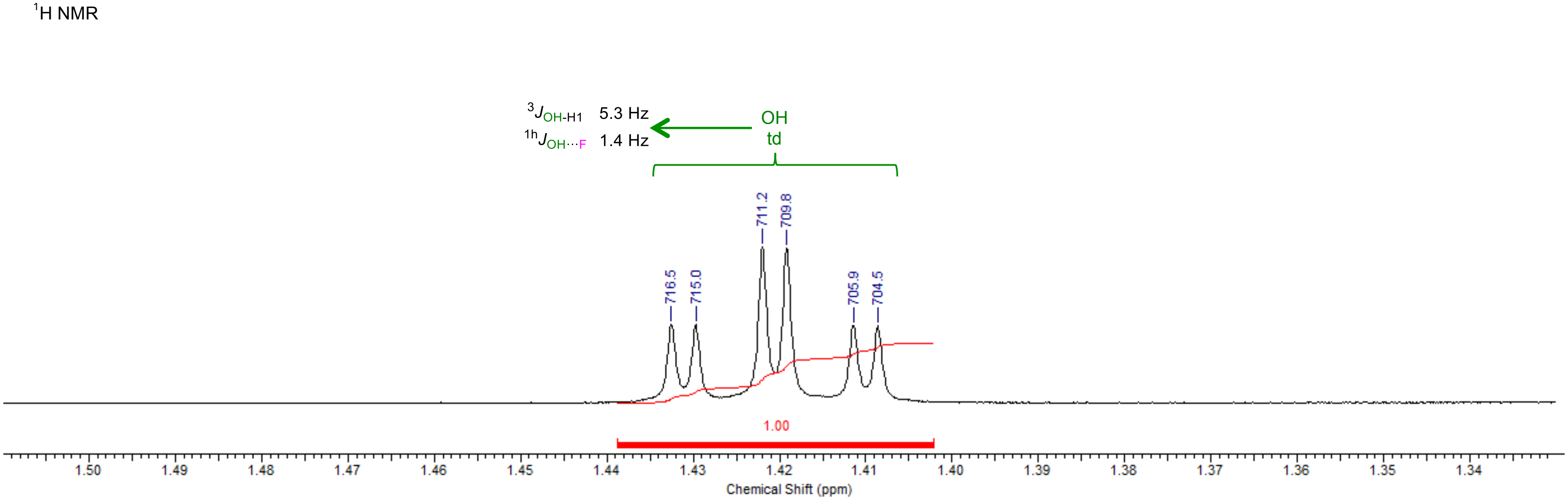

5.6.3 Comparison of ^{1}H NMR of OH signal at 25 °C and -50 °C ($CDCl_3$, 500 MHz) (D)

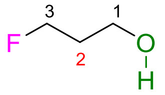

^{1}H NMR, -50 °C

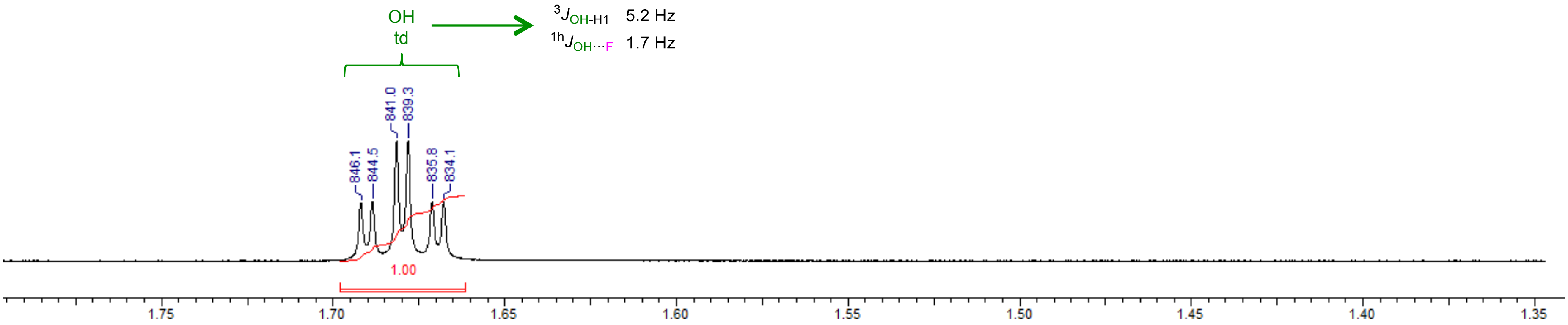

^{1}H NMR, 25 °C

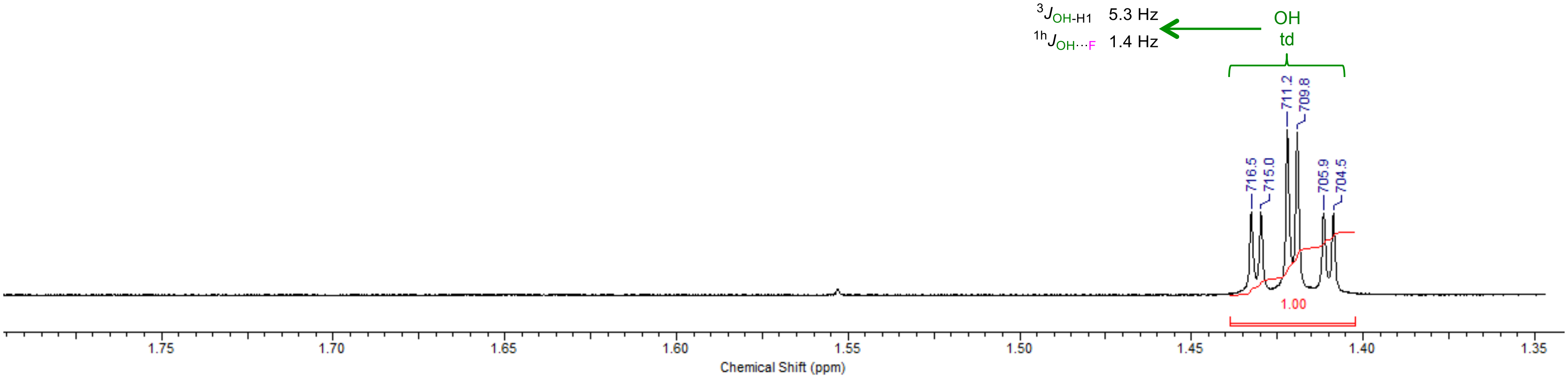

5.6.4 Detail of ^{1}H and ^{1}H[^{19}F] NMR of OH signals at -50 °C (CDCl$_3$, 500 MHz) (D)

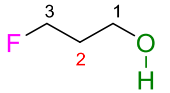

^{1}H{^{19}F} NMR, -50 °C

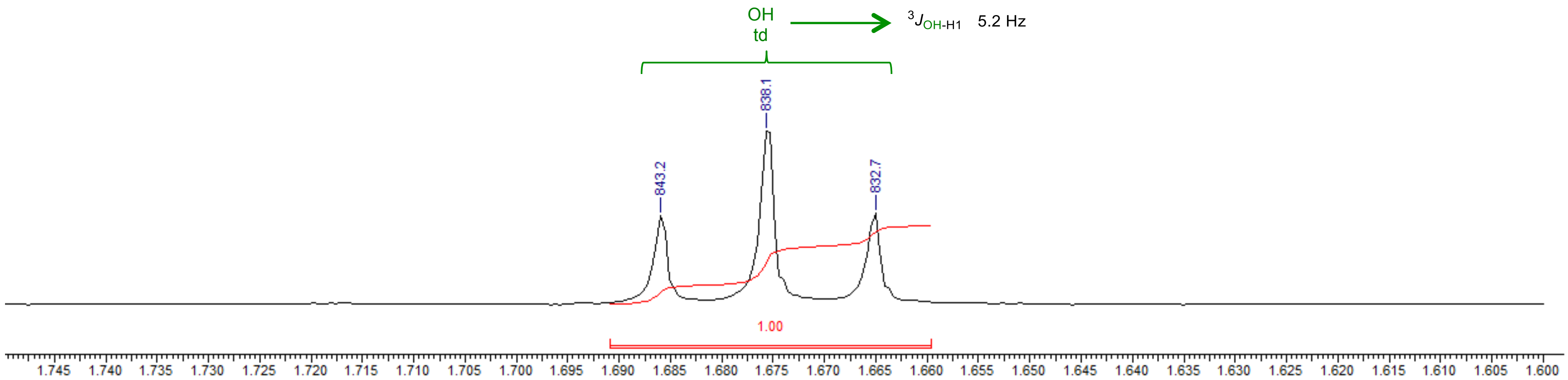

^{1}H NMR, -50 °C

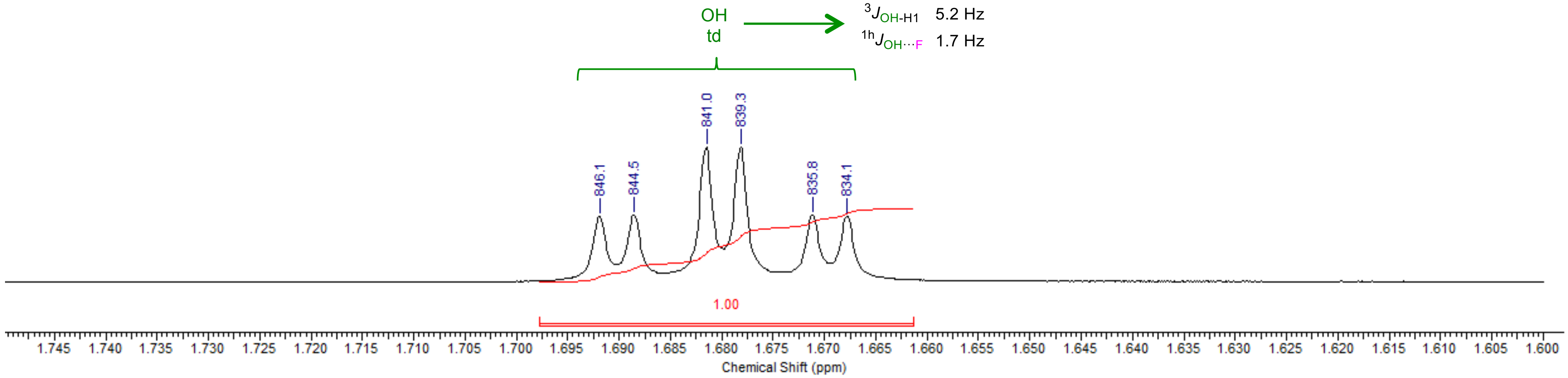

5.6.5 ^{19}F NMR spectrum ($CDCl_3$, 470 MHz, 25 °C) (D)

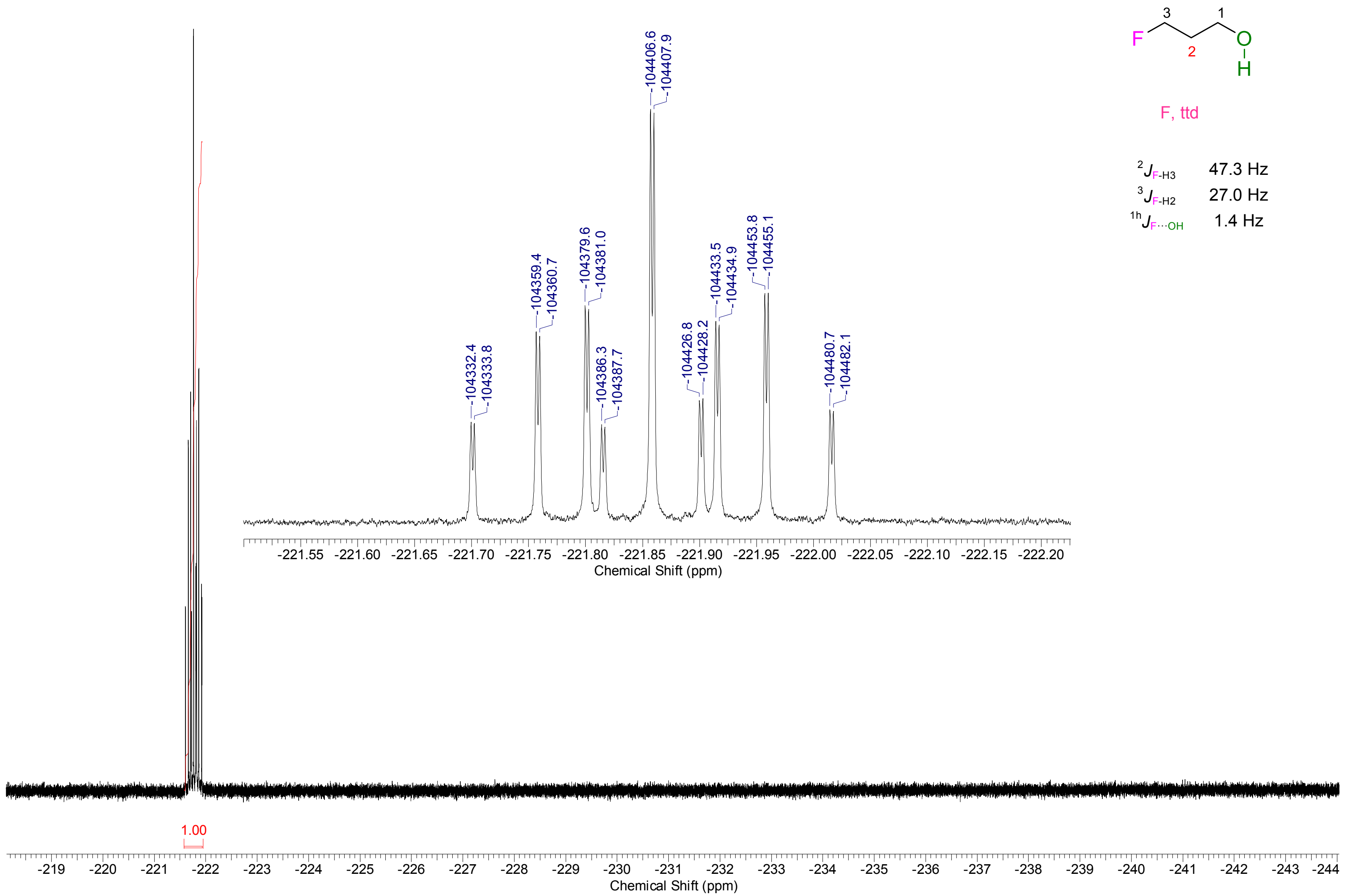

5.6.6 ^{19}F NMR spectrum ($CDCl_3$, 470 MHz, –50 °C) (D)

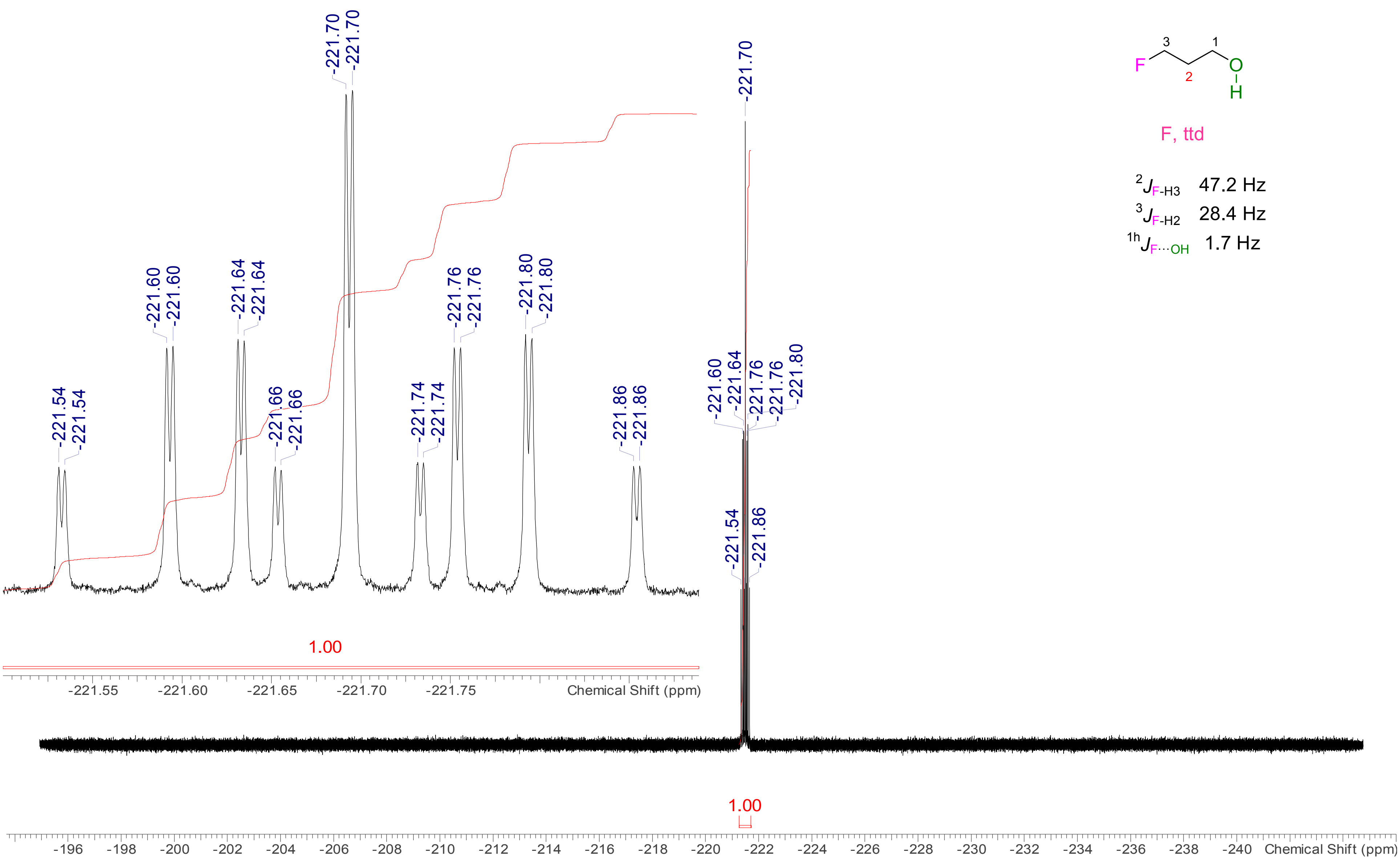

5.6.7 ^{1}H NMR spectrum (CD_2Cl_2, 500 MHz, 25 °C) (D)

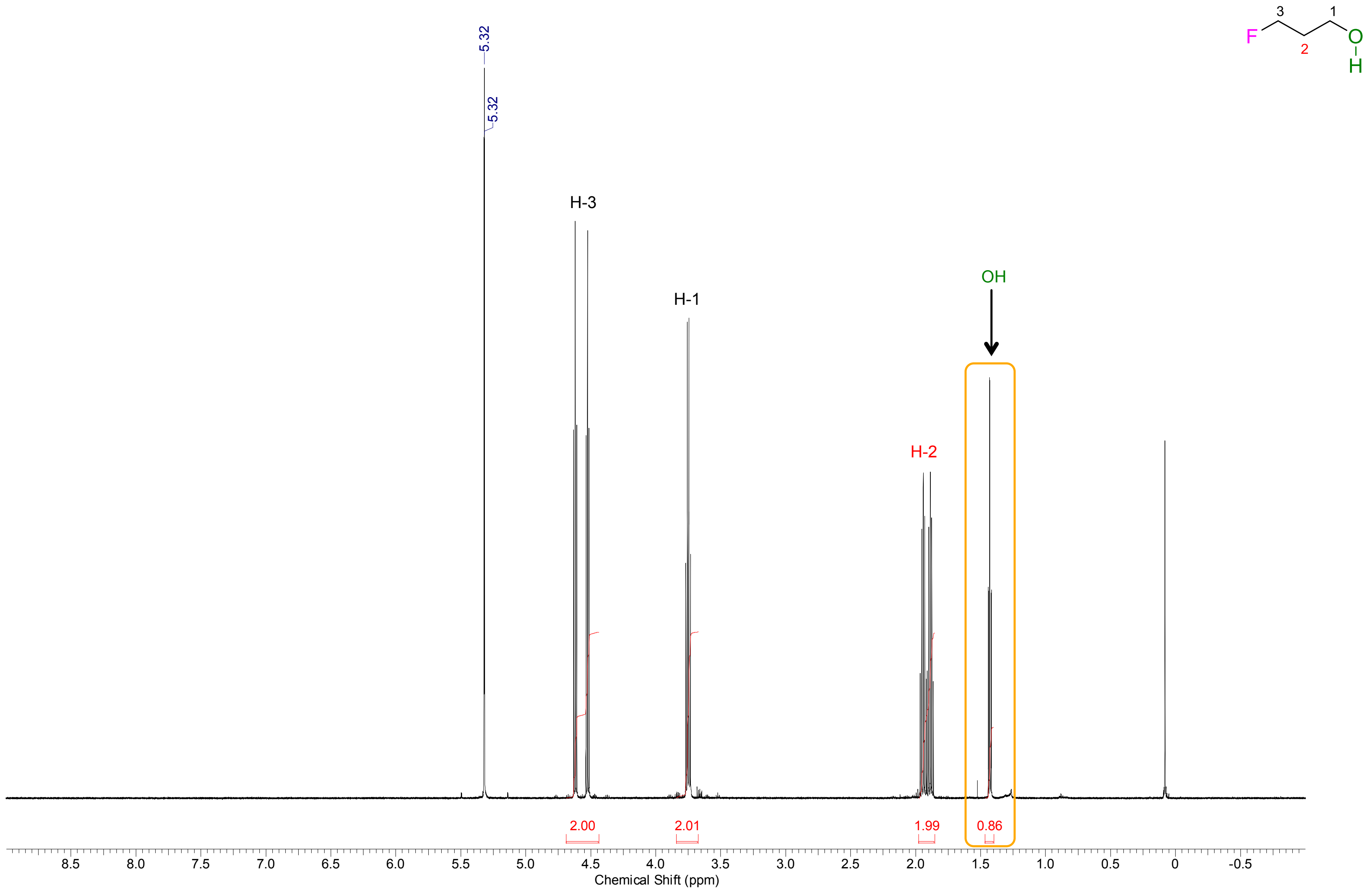

5.6.8 Detail of ^{1}H and $^{1}H[^{19}F]$ NMR of OH signal (CD_2Cl_2, 500 MHz, 25 °C) (D)

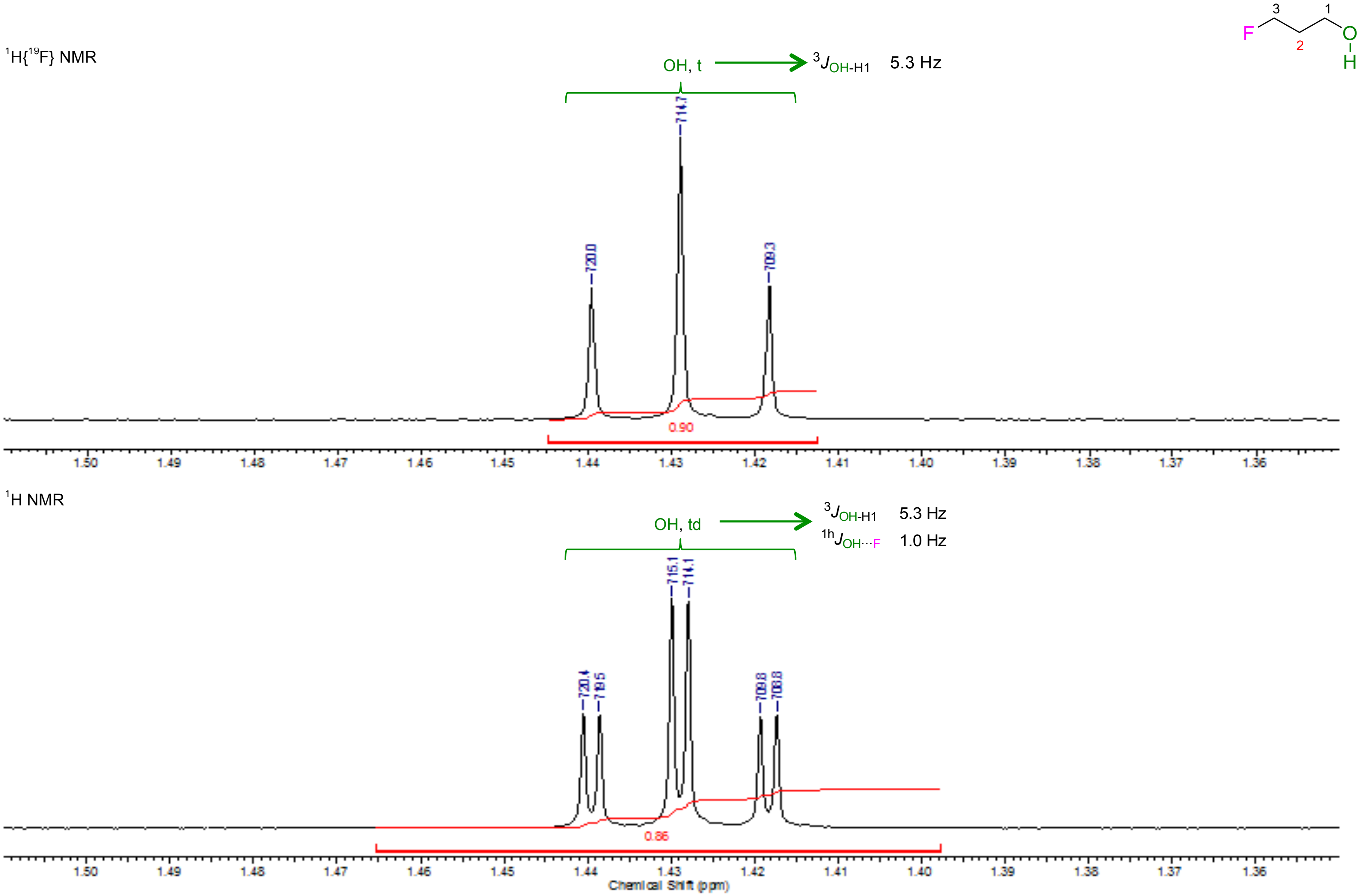

5.6.9 ^{19}F NMR spectrum (CD_2Cl_2, 470 MHz, 25 °C) (D)

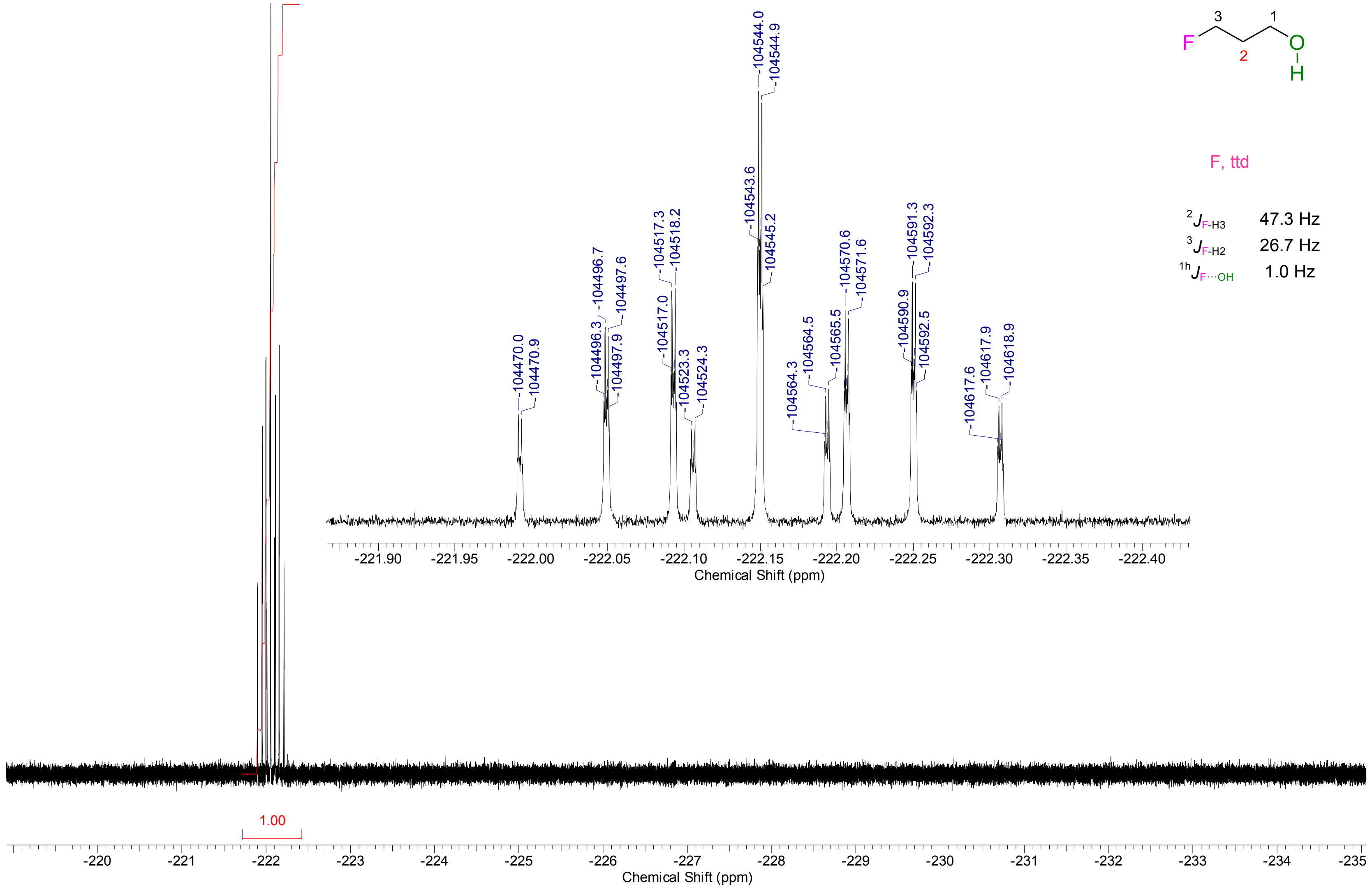

5.7 4,4-difluoropentan-2-ol (E)

5.7.1 ^{1}H NMR spectrum ($CDCl_3$, 500 MHz, 25 °C)

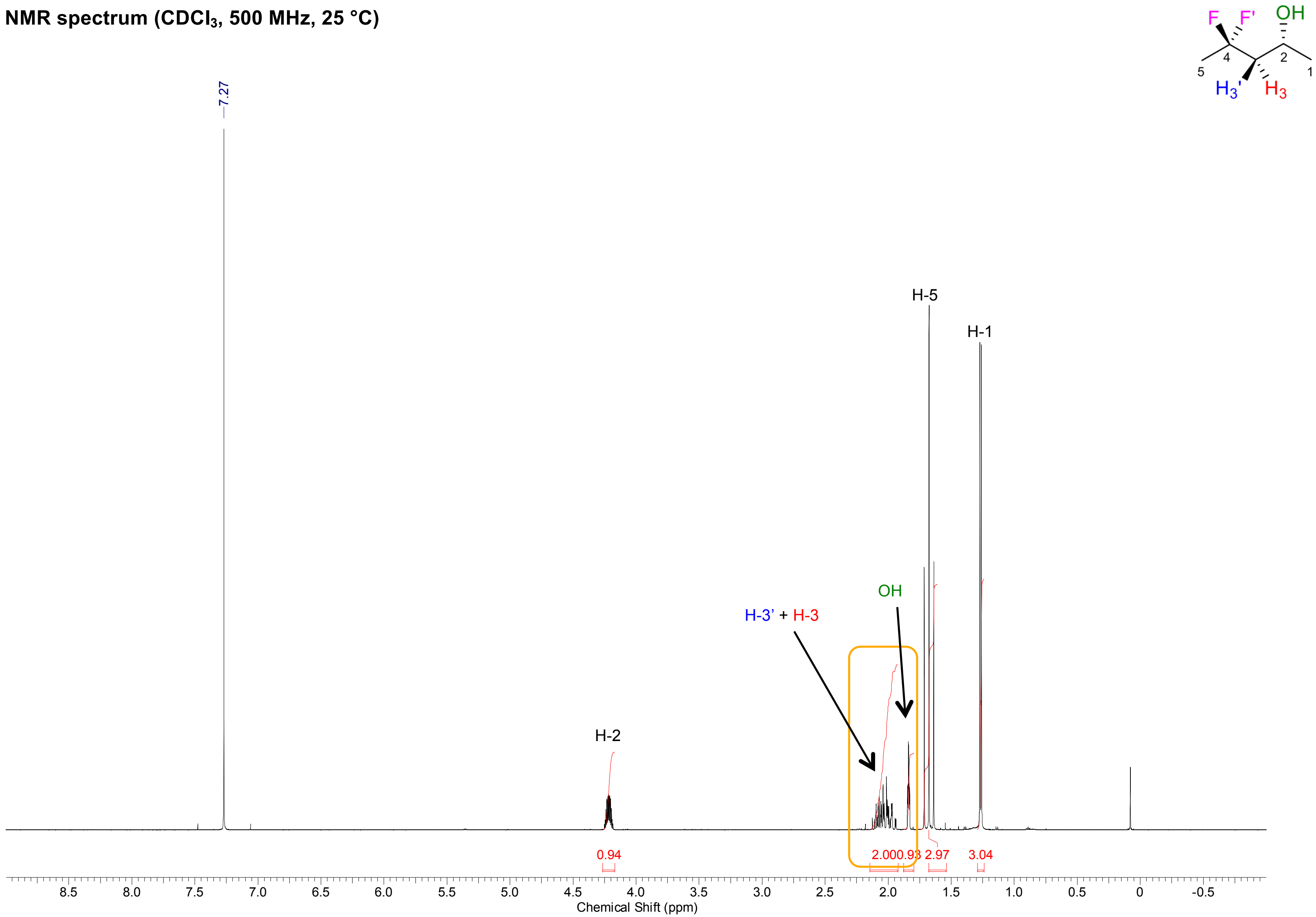

5.7.2 Detail of ^{1}H and ^{1}H[^{19}F] NMR of OH, H-3 and H-3' signals (CDCl$_3$, 500 MHz, 25 °C) (E)

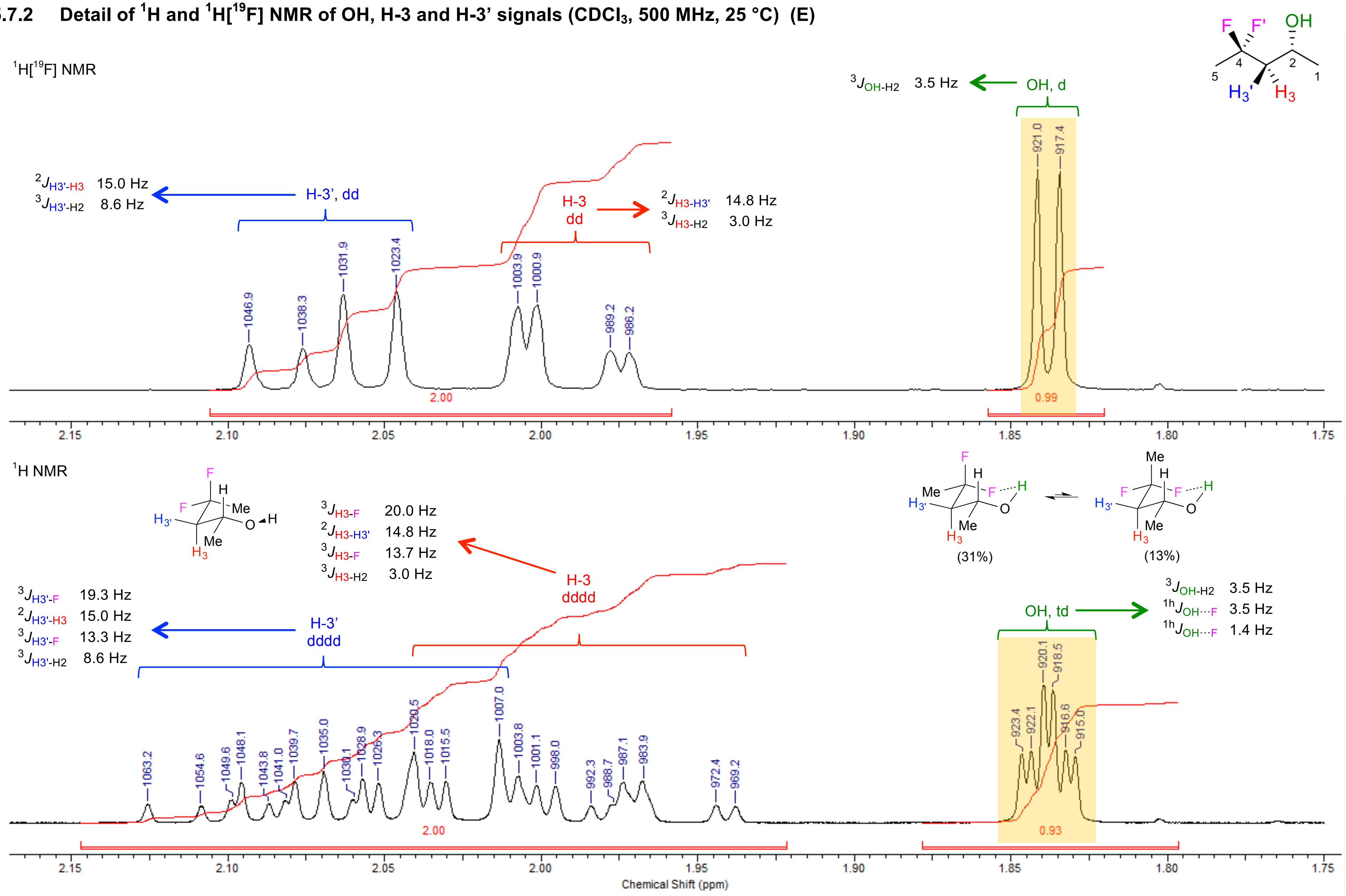

5.7.3 Homodecoupling of H-2 ($CDCl_3$, 500 MHz, 25 °C) (E)

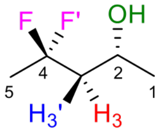

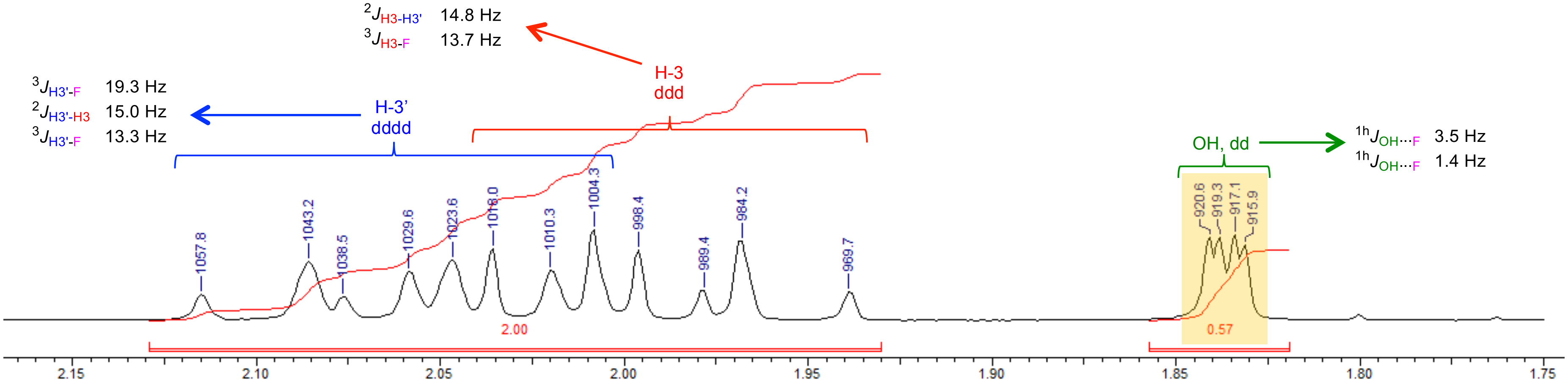

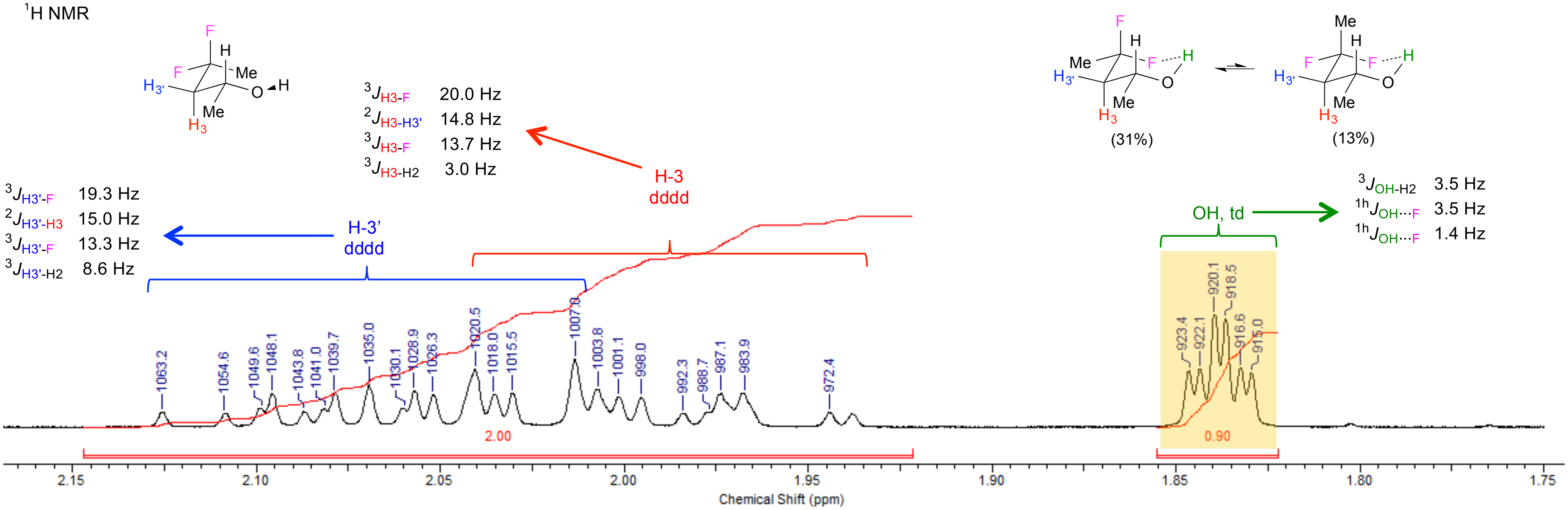

5.7.4 Comparison of ^{1}H NMR of OH, H-3' and H-3 signal at 25 °C and -50 °C ($CDCl_3$, 500 MHz) (E)

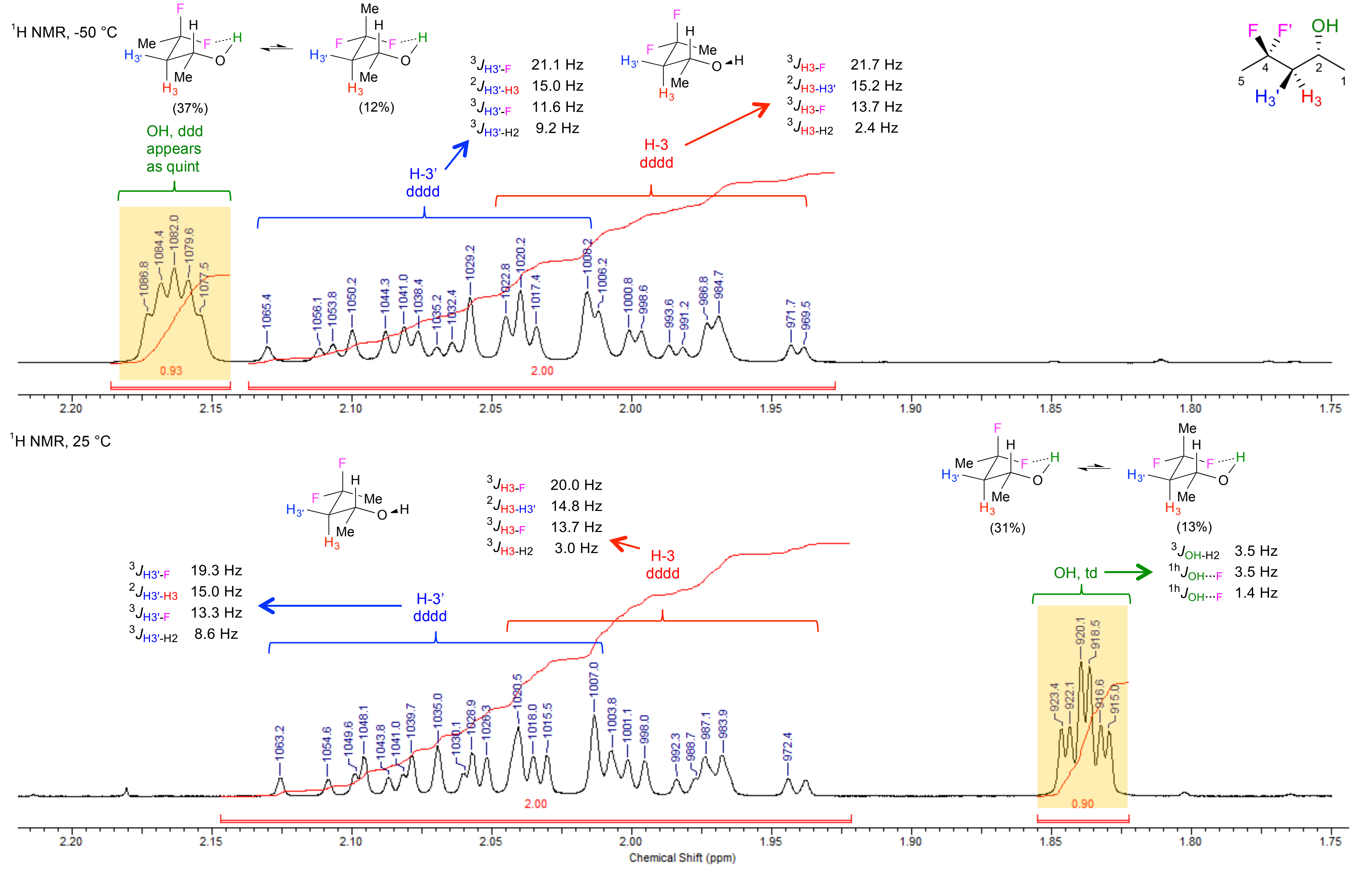

5.7.5 Detail of ^{1}H and ^{1}H[^{19}F] NMR of OH, H-3' and H-3 signals at -50 °C ($CDCl_3$, 500 MHz) (E)

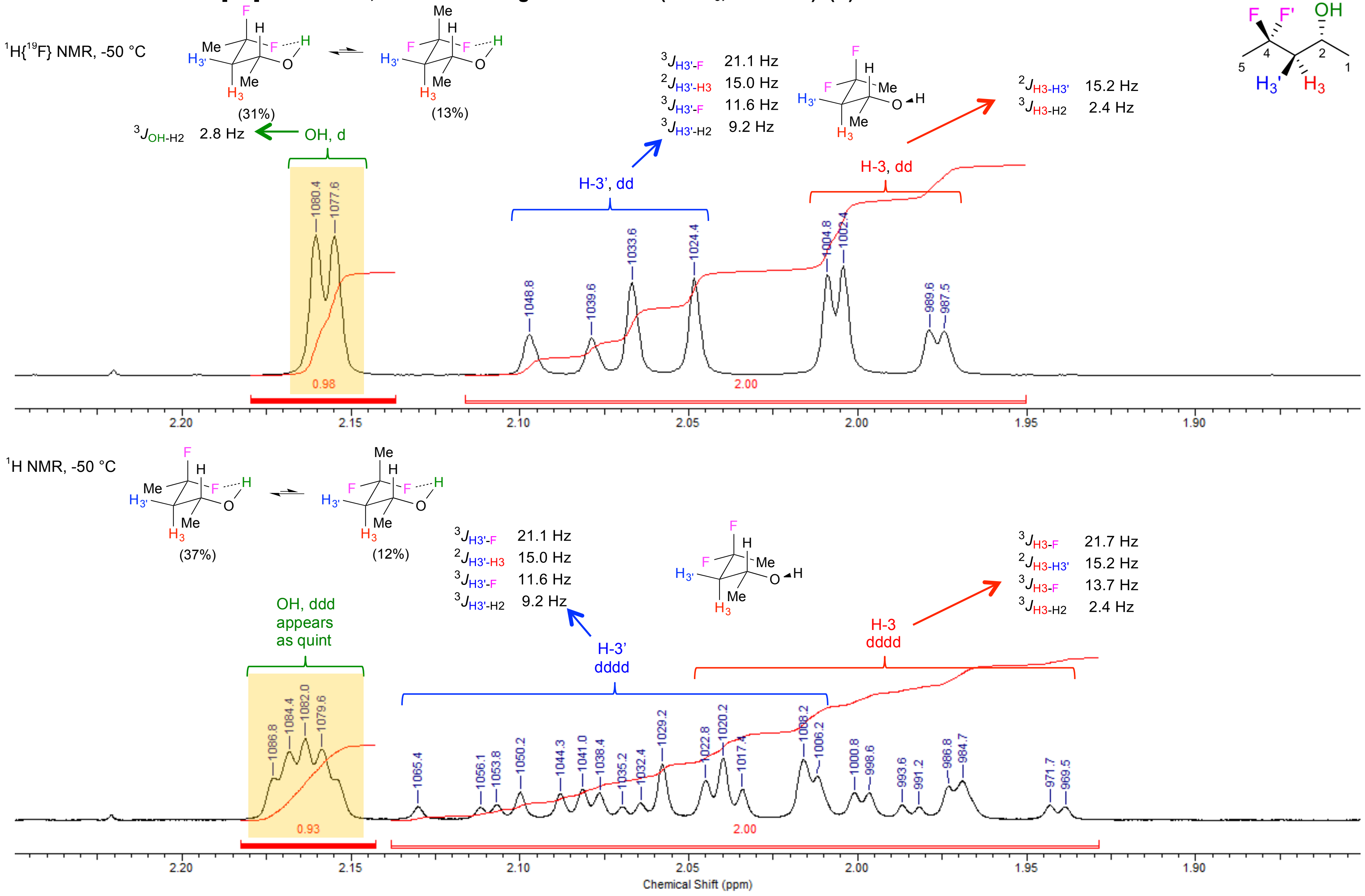

5.7.6 Homodecoupling of H-2 ($CDCl_3$, 500 MHz, -50 °C) (E)

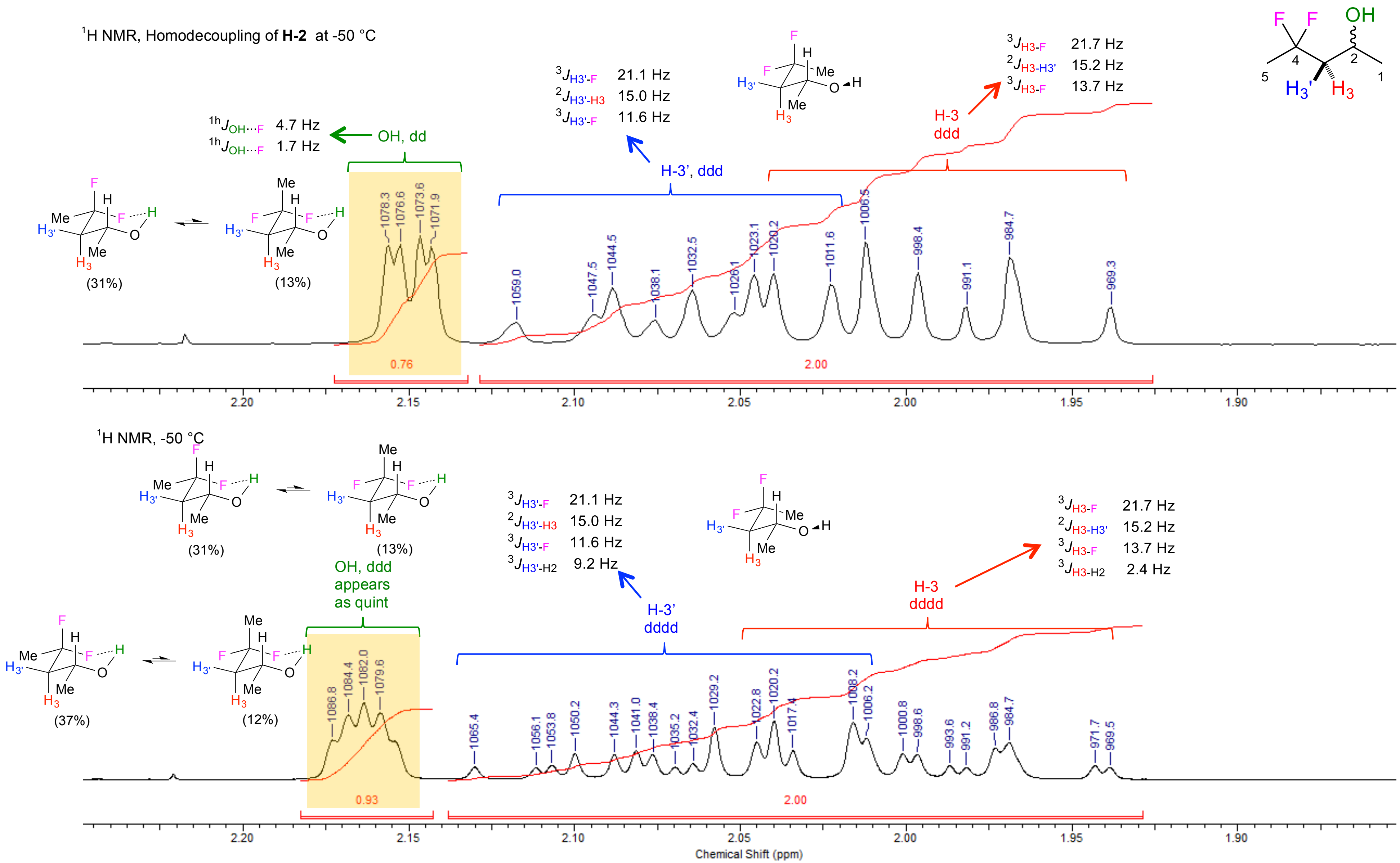

5.7.7 ^{19}F NMR spectrum ($CDCl_3$, 470 MHz, 25 °C) (E)

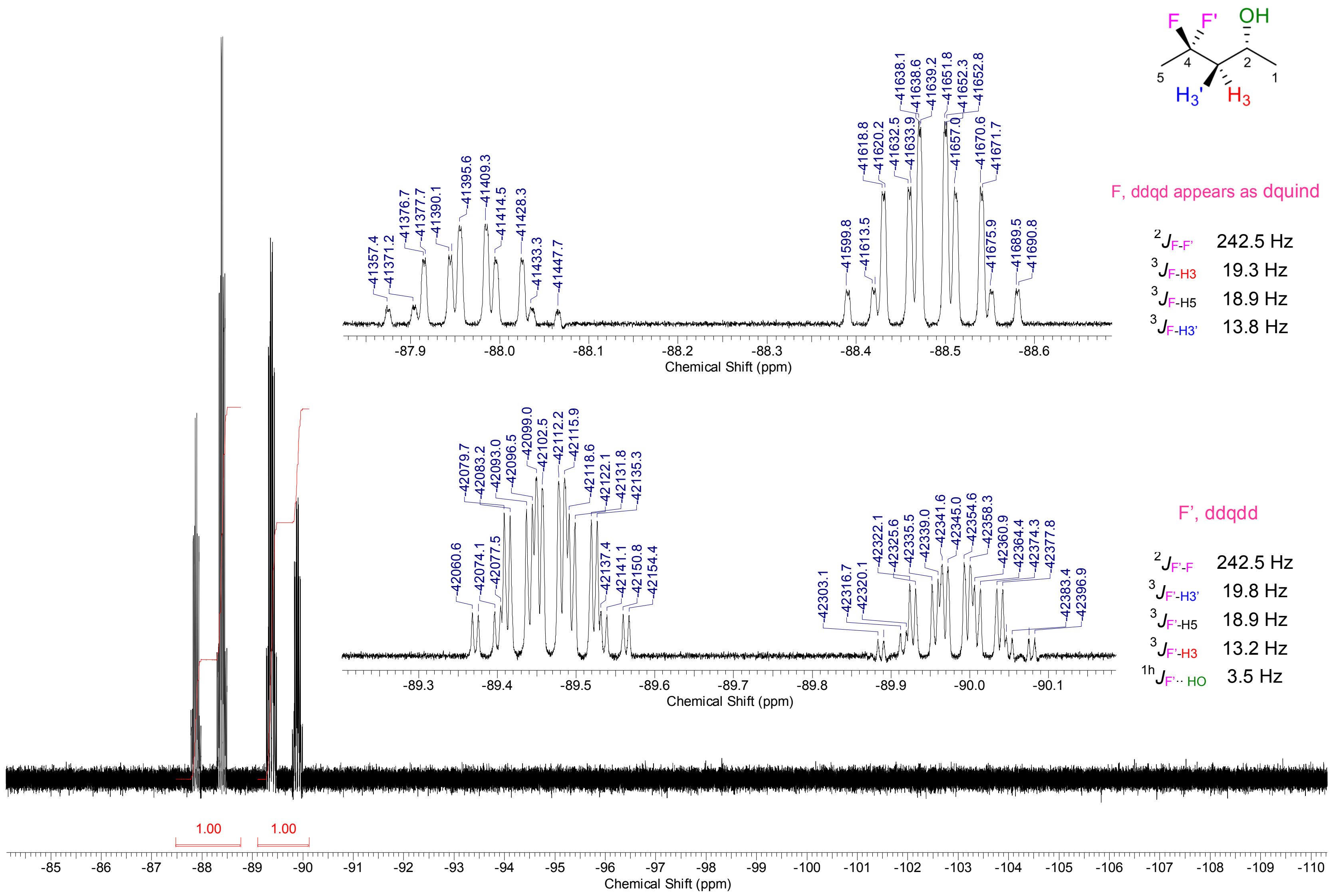

5.7.8 ^{19}F NMR spectrum ($CDCl_3$, 470 MHz, –50 °C) (E)

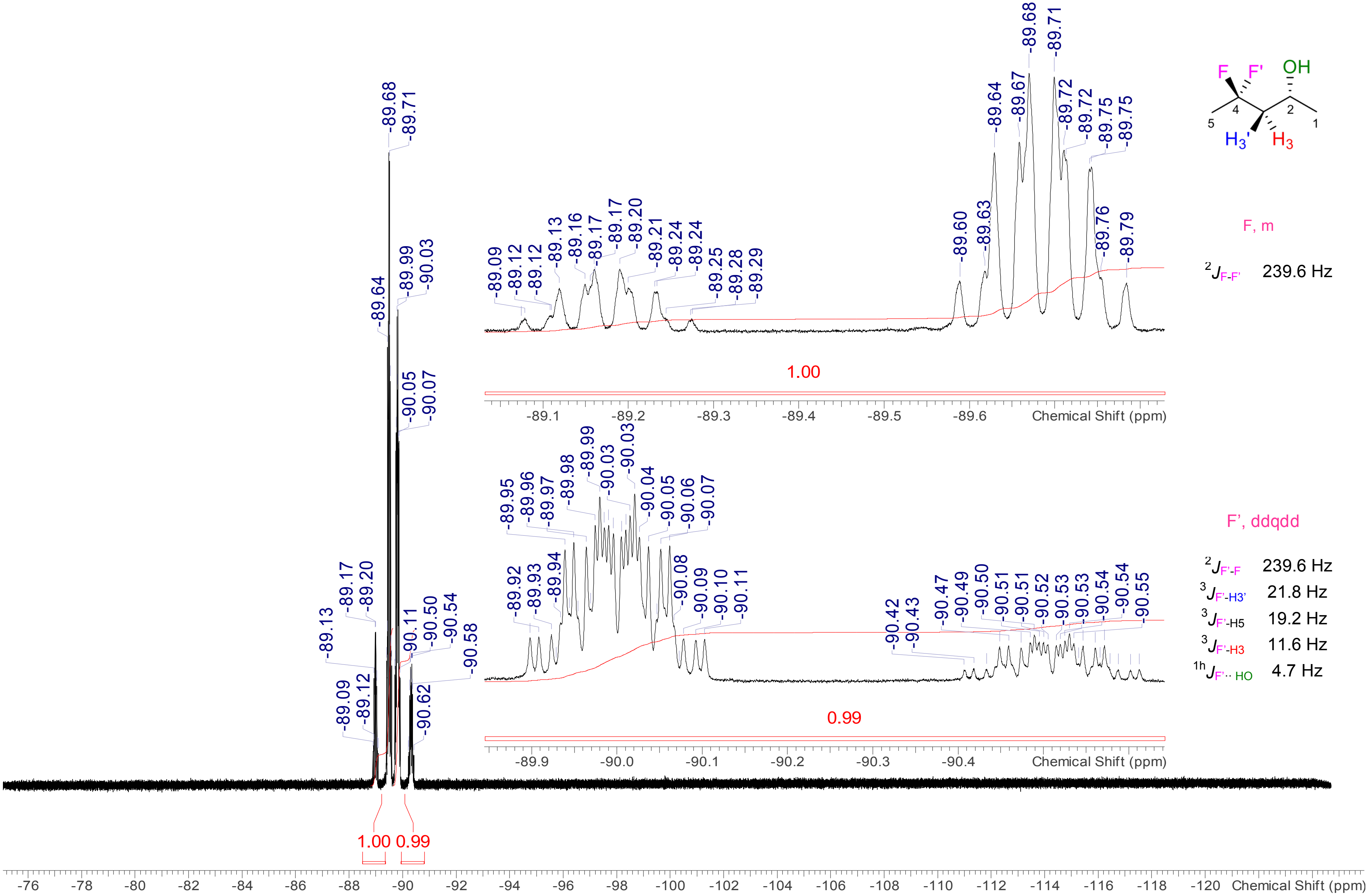

5.8 4,4-difluorobutan-2-ol (F)

5.8.1 ^{1}H NMR spectrum ($CDCl_3$, 500 MHz, 25 °C)

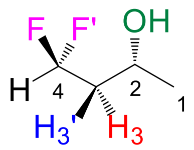

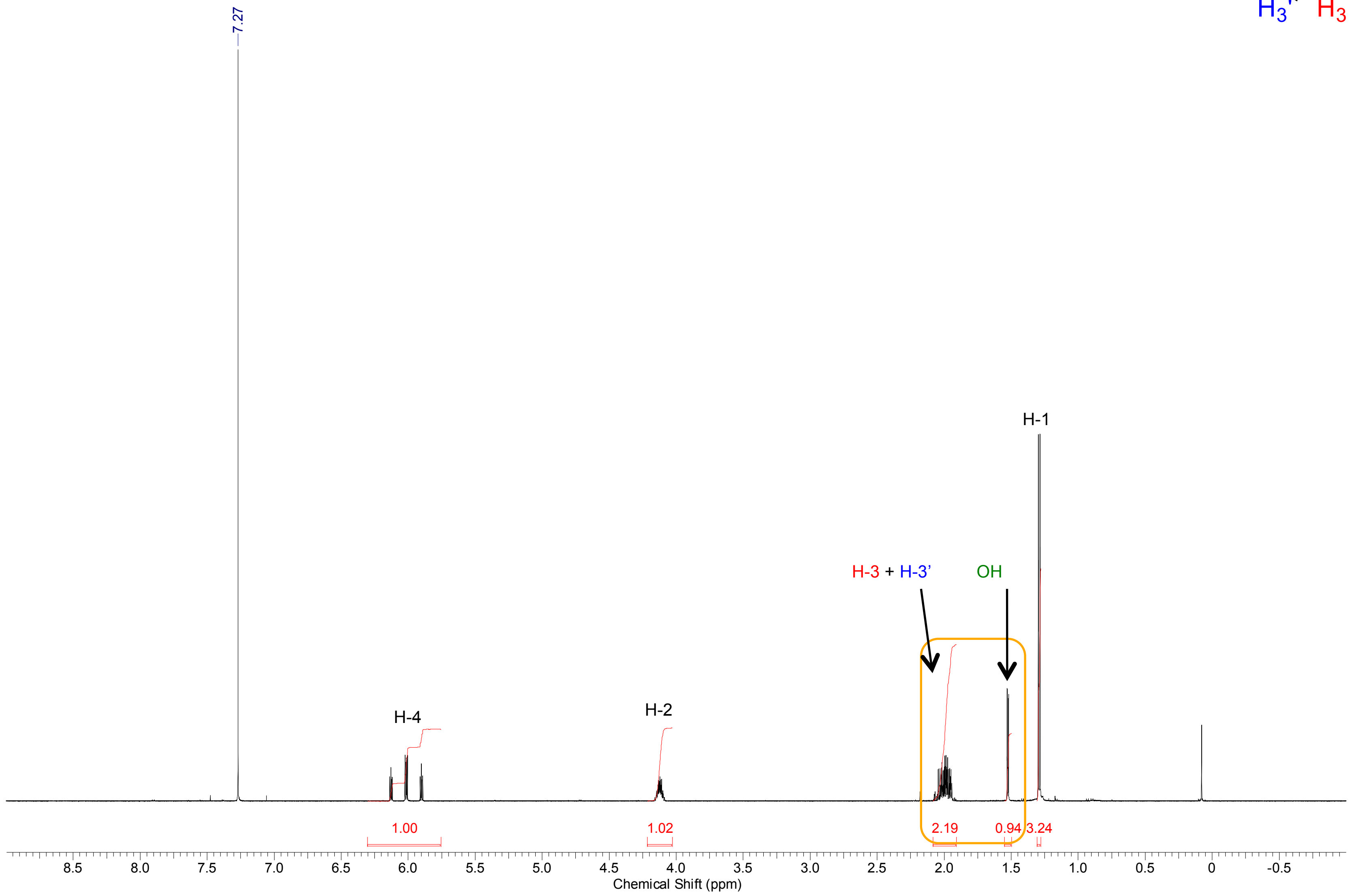

5.8.2 Detail of ^{1}H and ^{1}H[^{19}F] NMR of OH, H-3 and H-3' signals (CDCl$_3$, 500 MHz, 25 °C) (F)

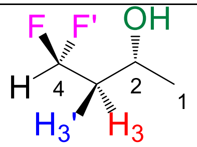

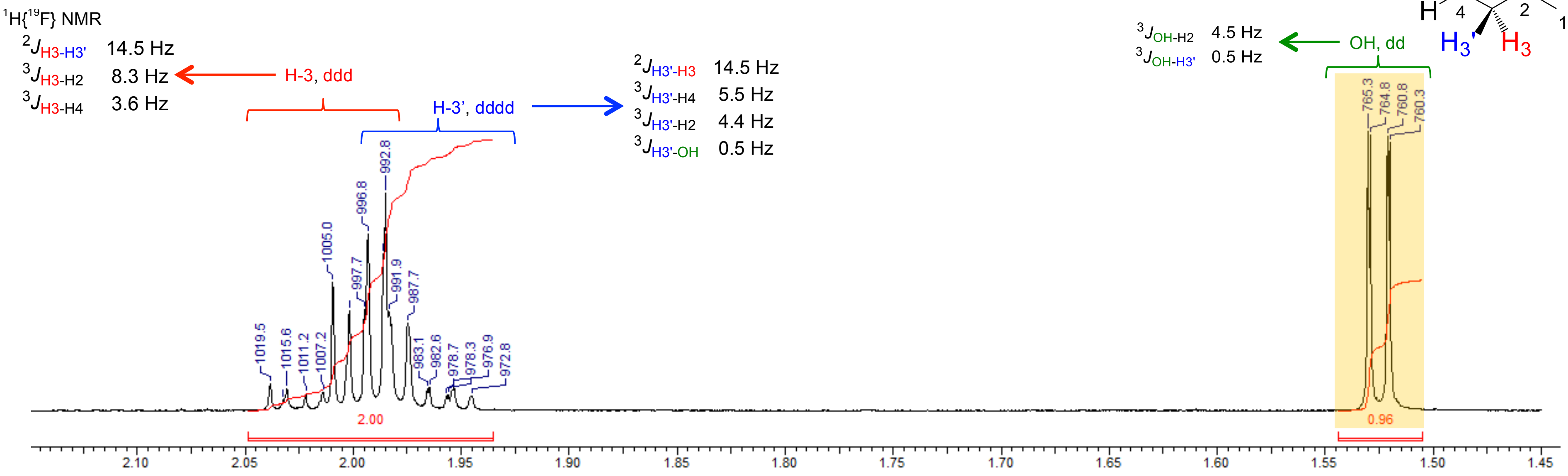

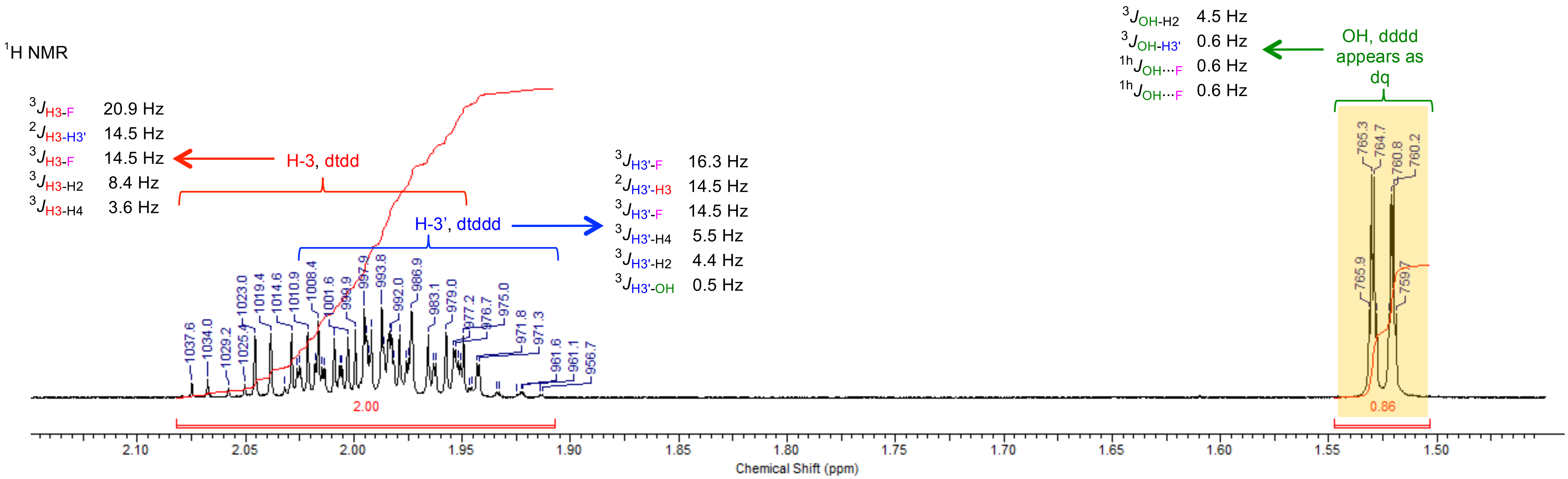

5.8.3 Detail of ^{1}H and ^{1}H[^{19}F] NMR of OH signal ($CDCl_3$, 500 MHz, 25 °C) (F)

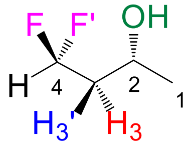

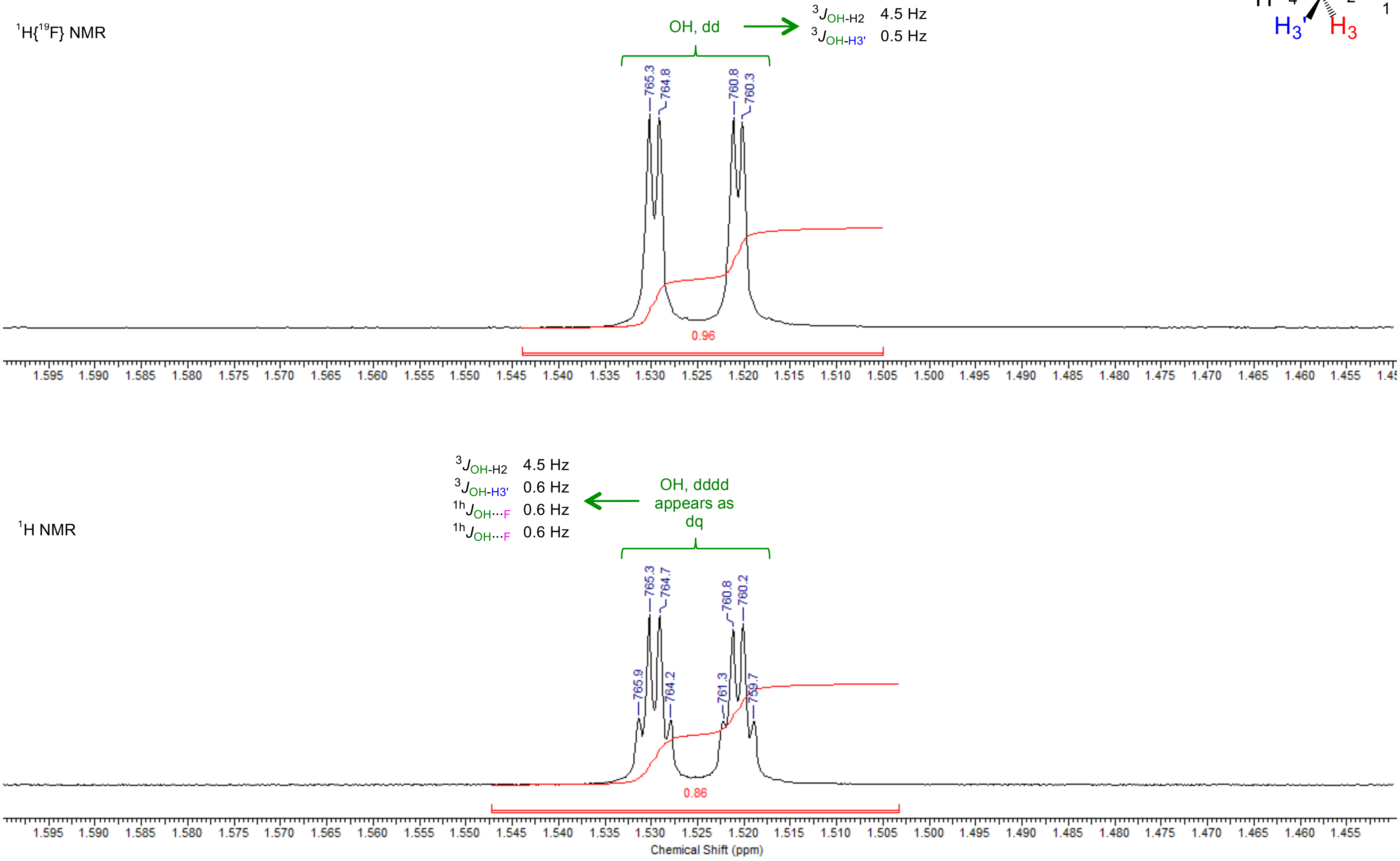

5.8.4 ^{19}F NMR spectrum ($CDCl_3$, 470 MHz, 25 °C) (F)

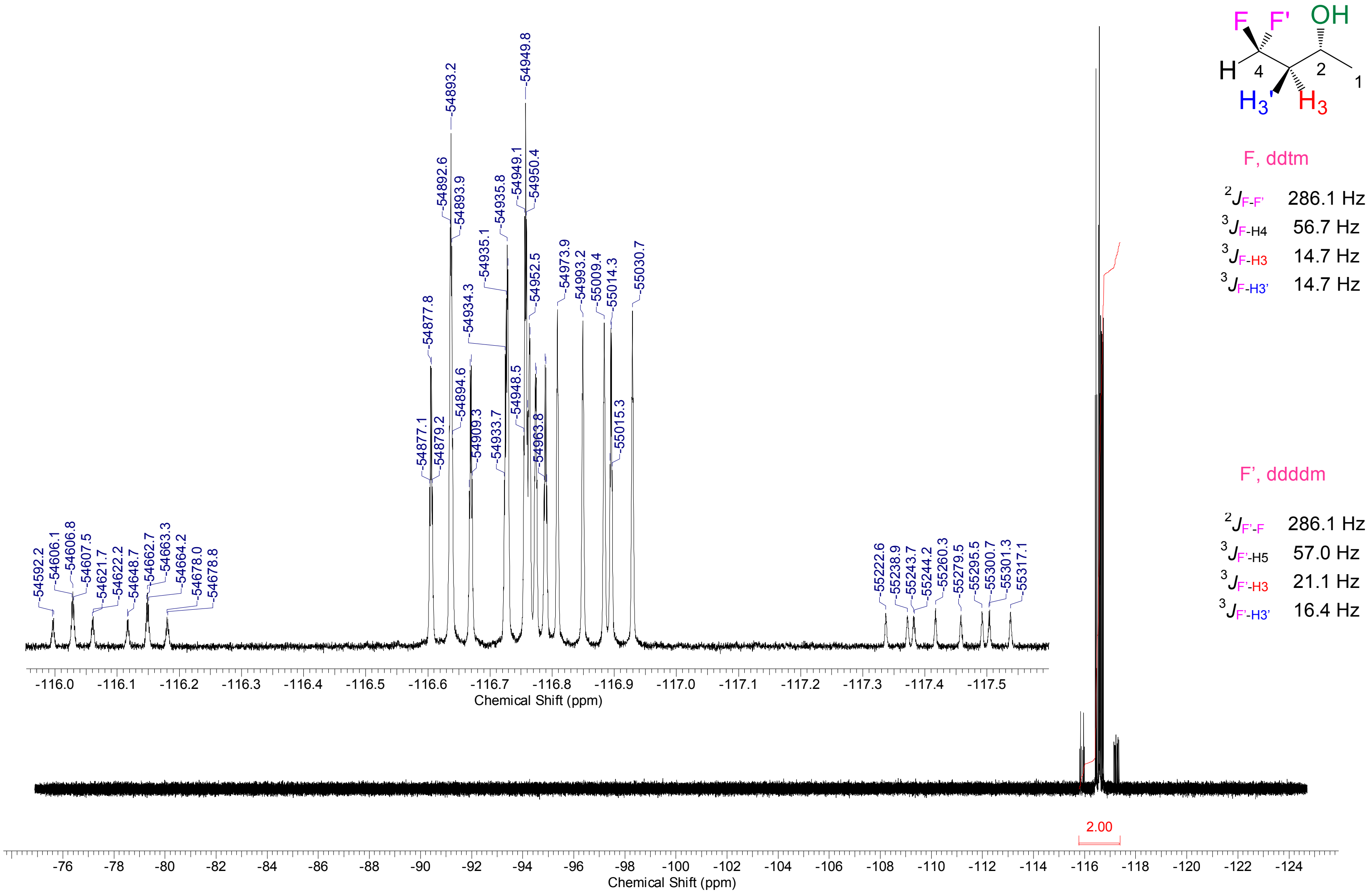

5.9 3,3-difluoropropan-1-ol (G)

5.9.1 ^{1}H NMR spectrum ($CDCl_3$, 500 MHz, 25 °C)

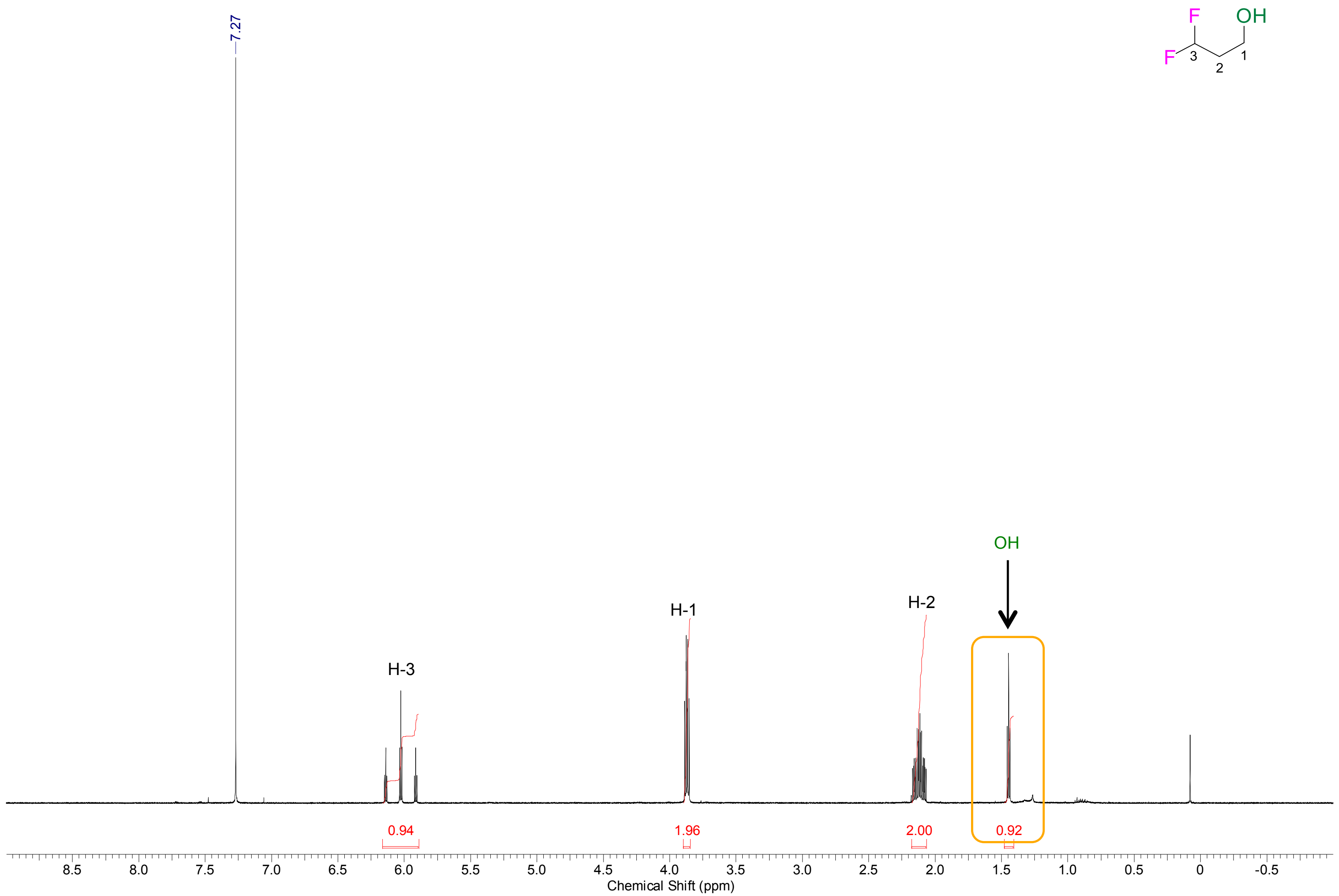

5.9.2 Detail of ^{1}H and $^{1}H[^{19}F]$ NMR of OH signal ($CDCl_3$, 500 MHz, 25 °C) (G)

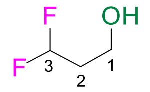

$^{1}H\{^{19}F\}$ NMR

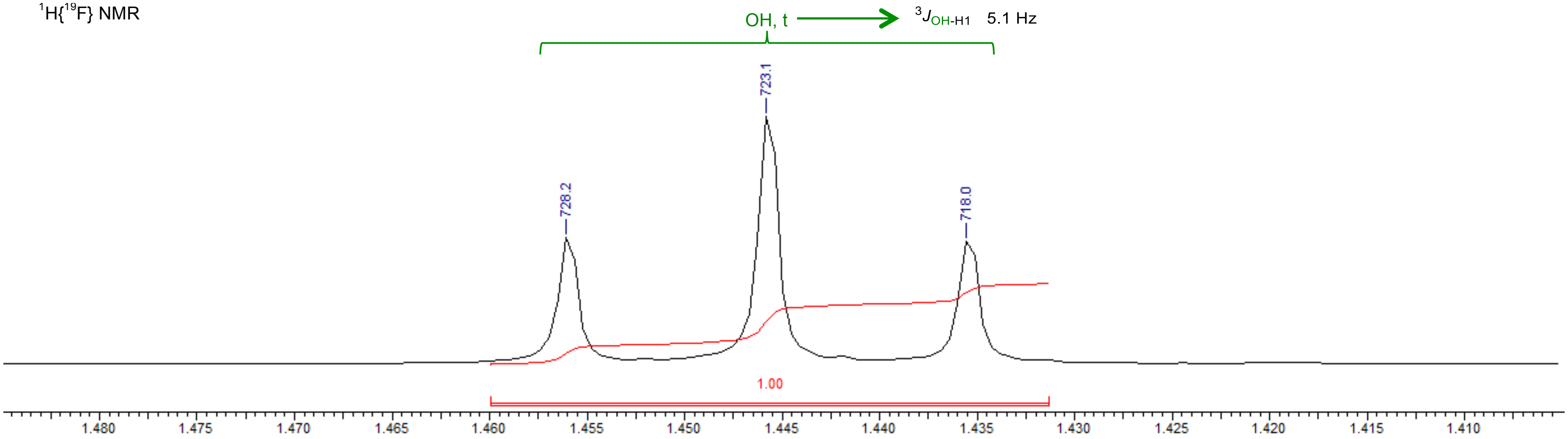

^{1}H NMR

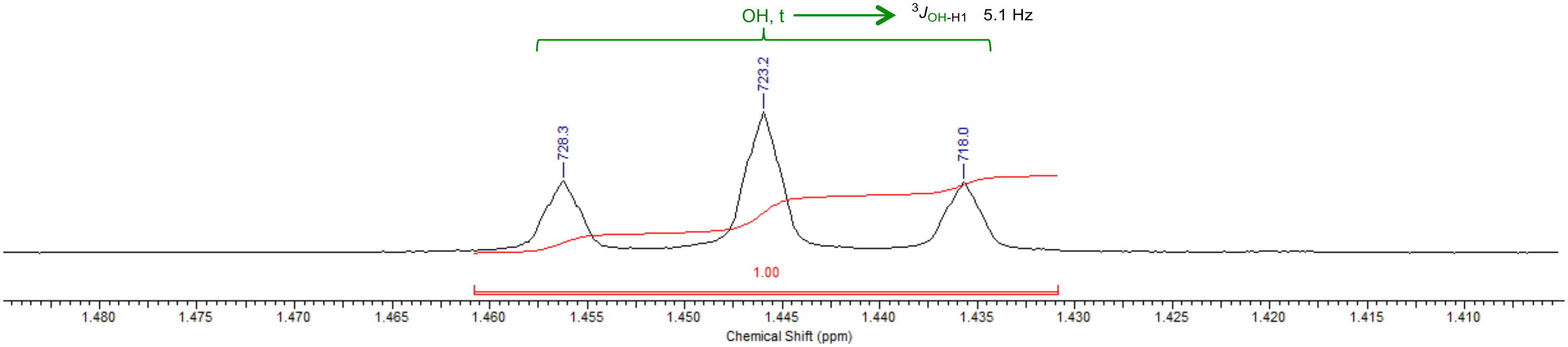

5.9.3 Detail of ^{1}H (enhanced resolution spectra) and ^{1}H[^{19}F] NMR of OH signal ($CDCl_3$, 500 MHz, 25 °C) (G)

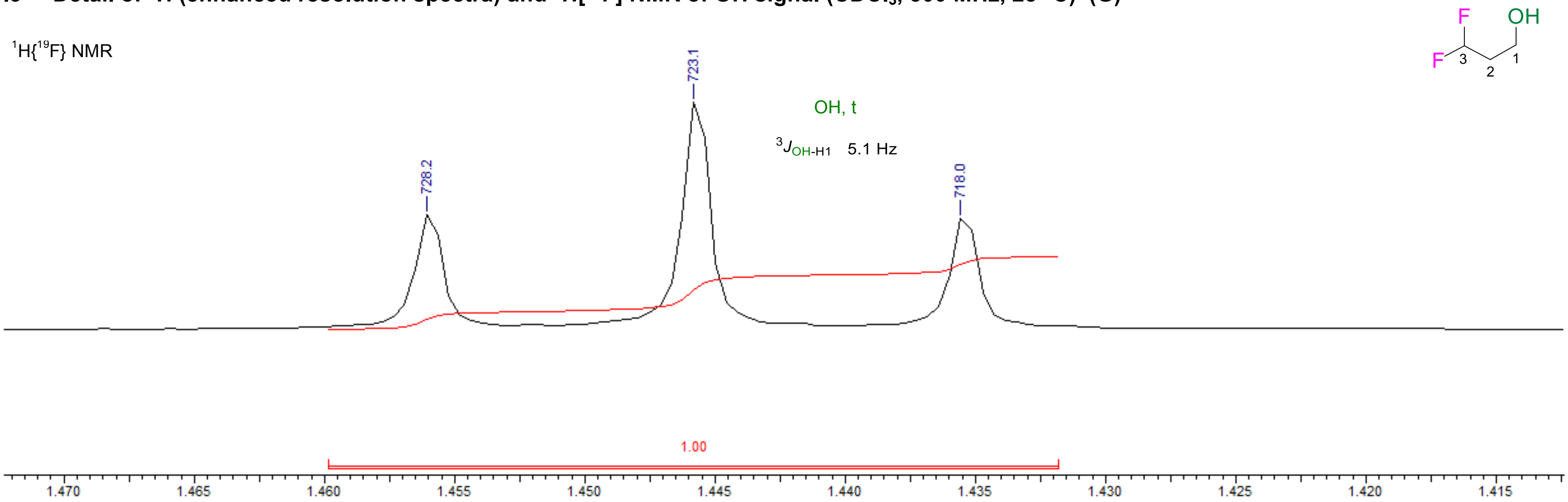

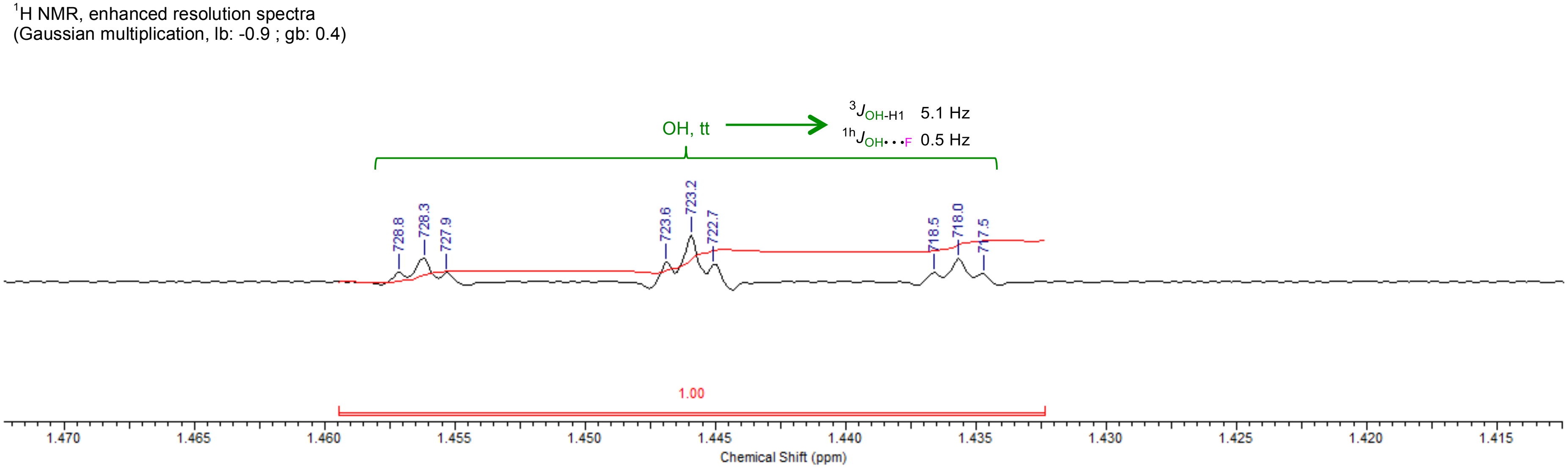

5.9.4 ^{19}F NMR spectrum ($CDCl_3$, 470 MHz, 25 °C) (G)

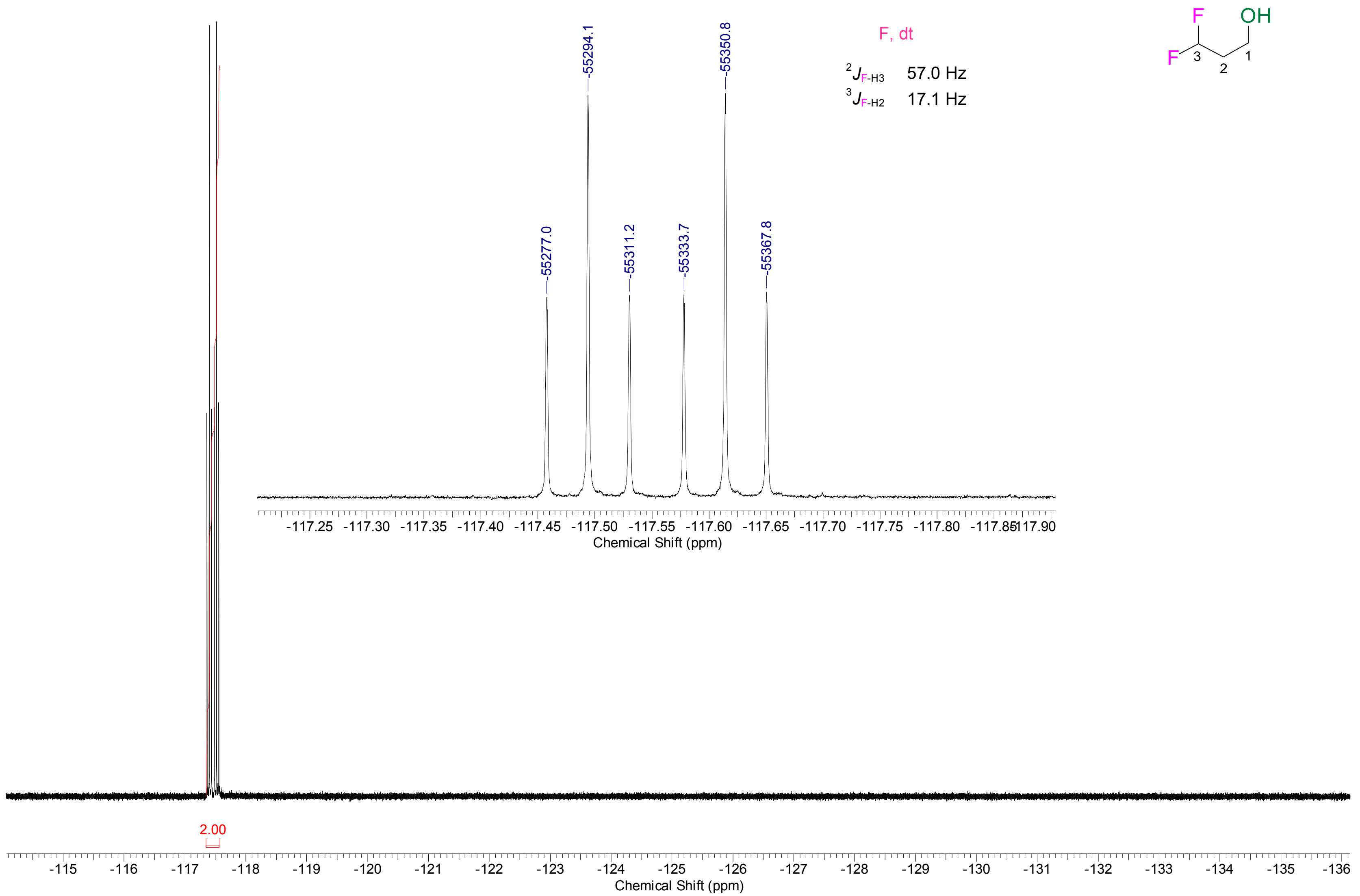

5.10 4,4,4-trifluorobutan-2-ol (H)

5.10.1 ^{1}H NMR spectrum ($CDCl_3$, 500 MHz, 25 °C)

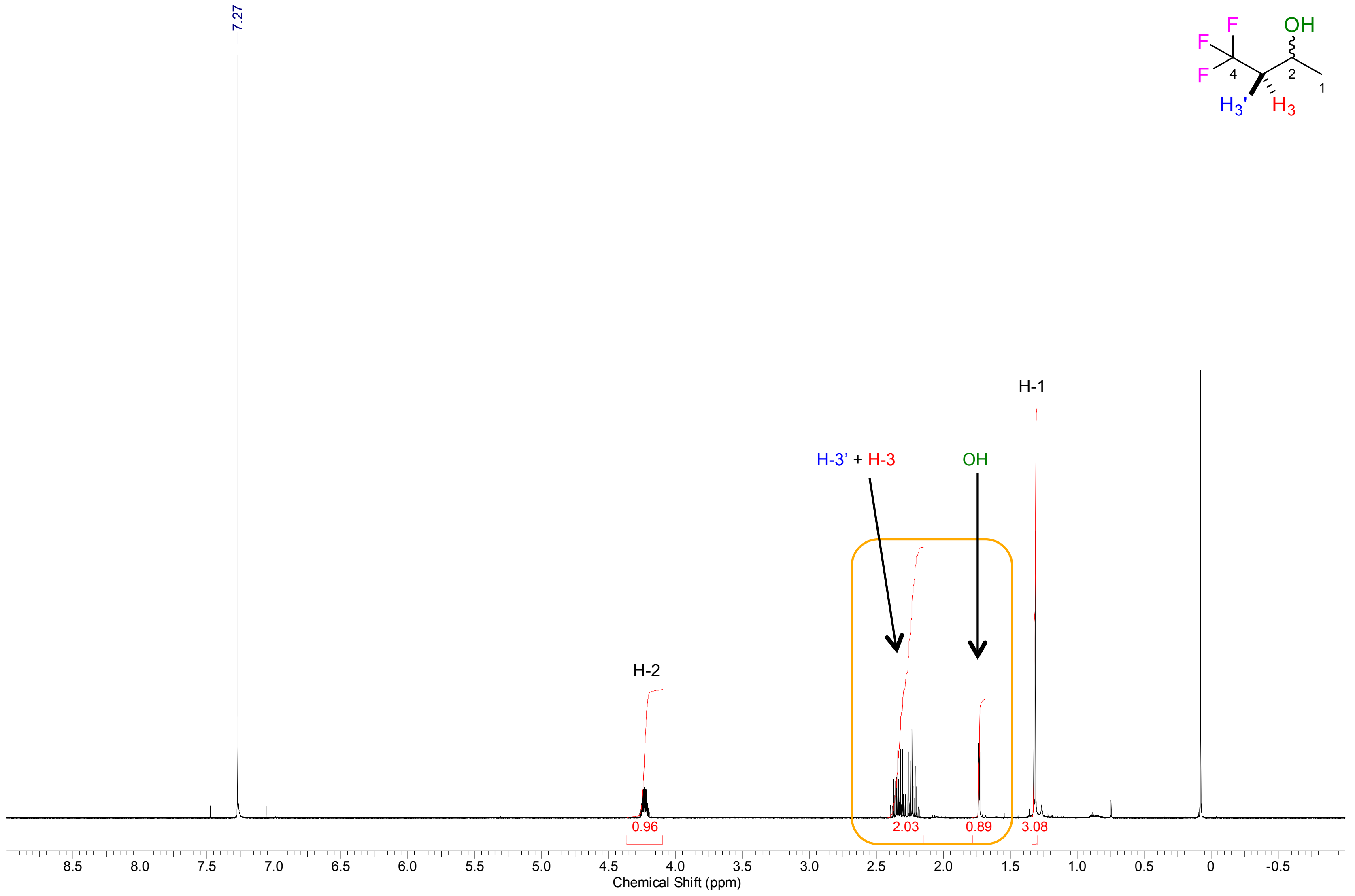

5.10.2 Detail of ^{1}H and ^{1}H[^{19}F] NMR of OH, H-3 and H-3' signals ($CDCl_3$, 500 MHz, 25 °C) (H)

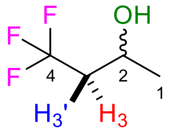

^{1}H{^{19}F} NMR

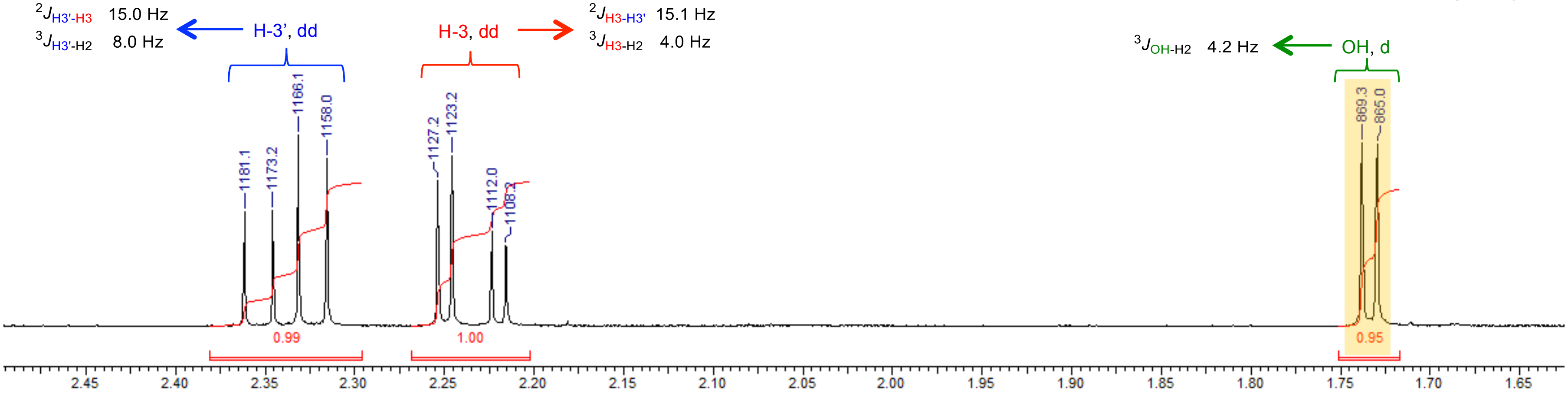

^{1}H NMR

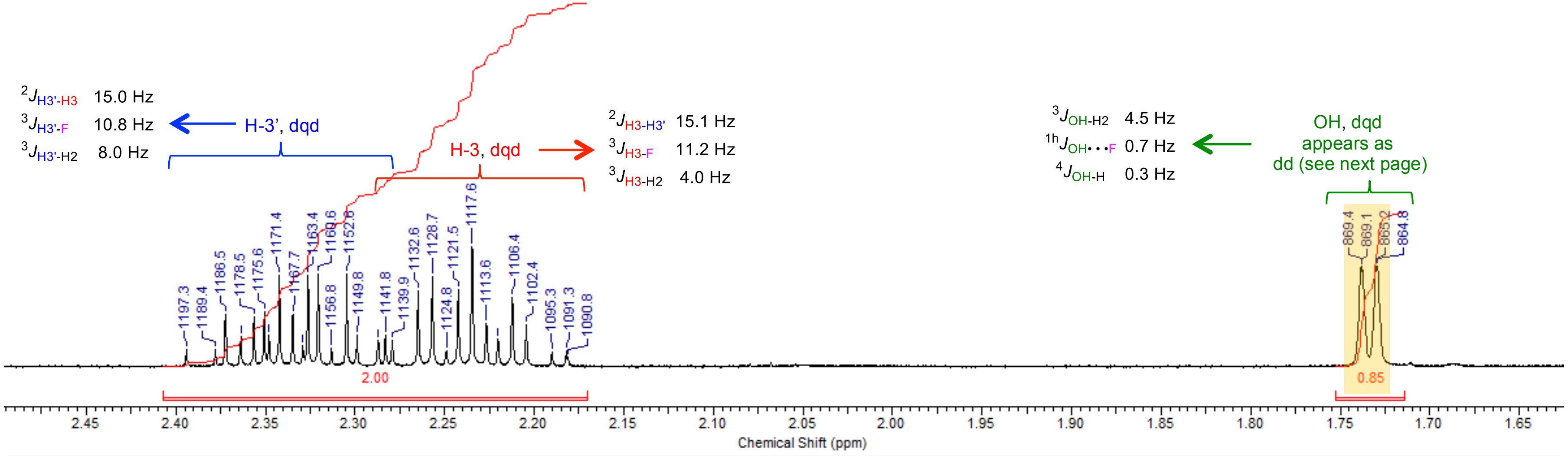

5.10.3 Detail of enhanced resolution spectra of ^{1}H and $^{1}H[^{19}F]$ NMR of OH signal ($CDCl_3$, 500 MHz, 25 °C) (H)

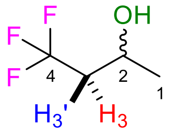

$^{1}H\{^{19}F\}$ NMR, enhanced resolution spectra
(Gaussian multiplication, lb: -1 ; gb: 0.5)

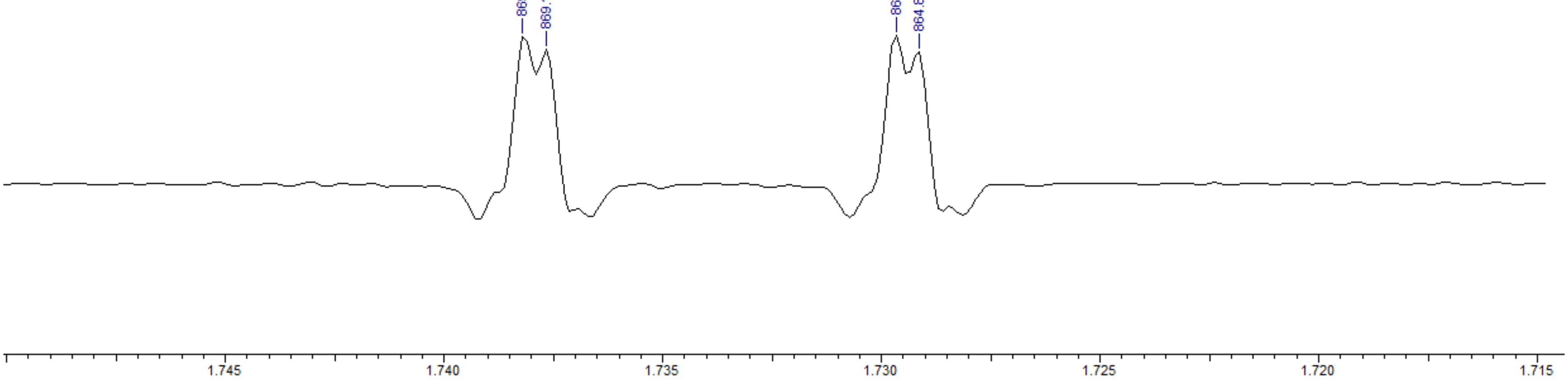

^{1}H NMR, enhanced resolution spectra
(Gaussian multiplication, lb: -0.7 ; gb: 0.35)

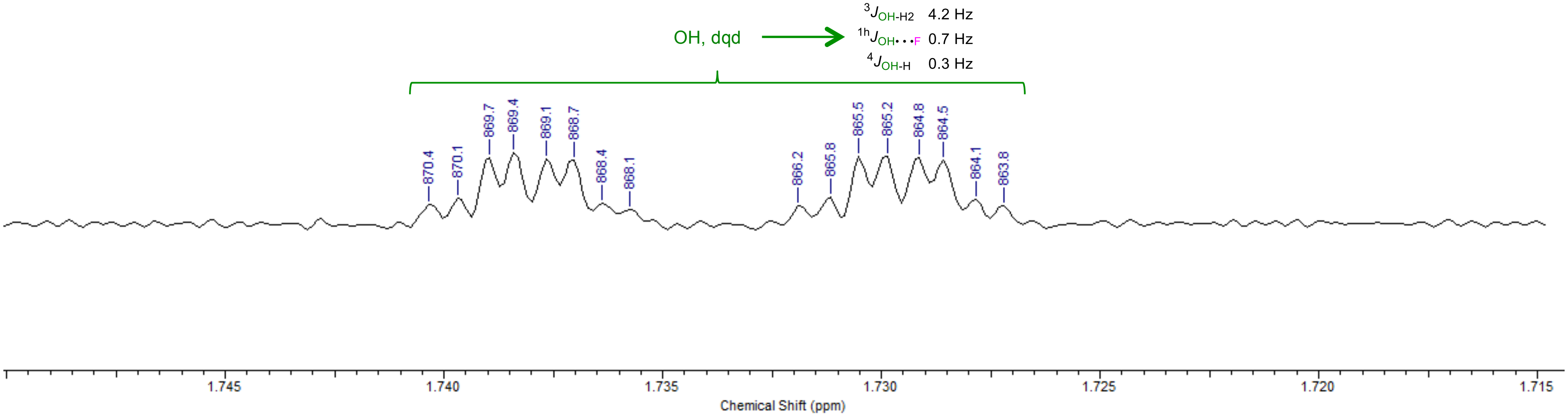

5.10.4 ^{19}F NMR spectrum ($CDCl_3$, 470 MHz, 25 °C) (H)

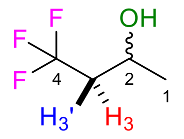

F, m

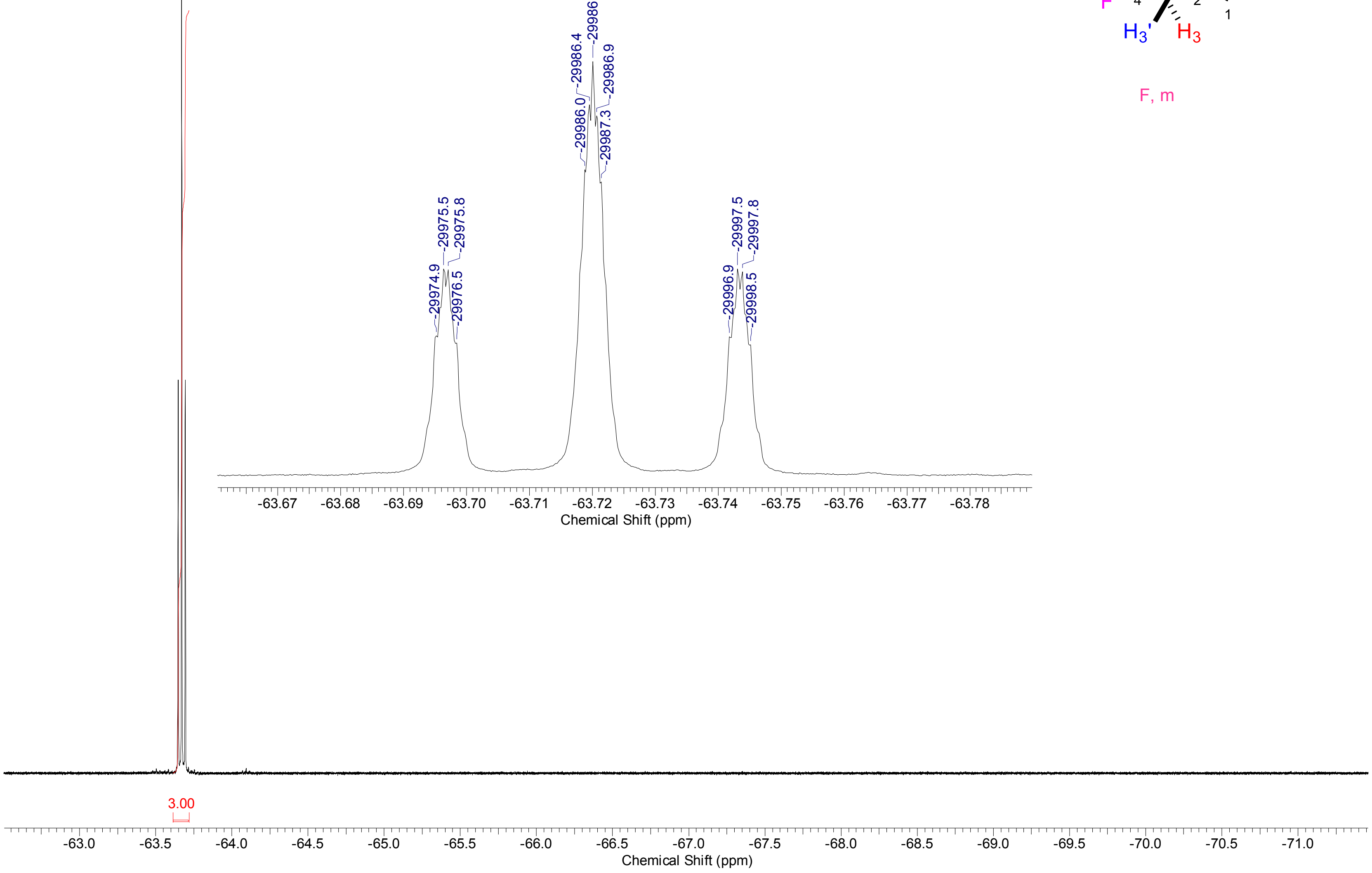

5.11 3,3,3-trifluoropropan-1-ol (I)

5.11.1 ^{1}H NMR spectrum ($CDCl_3$, 500 MHz, 25 °C)

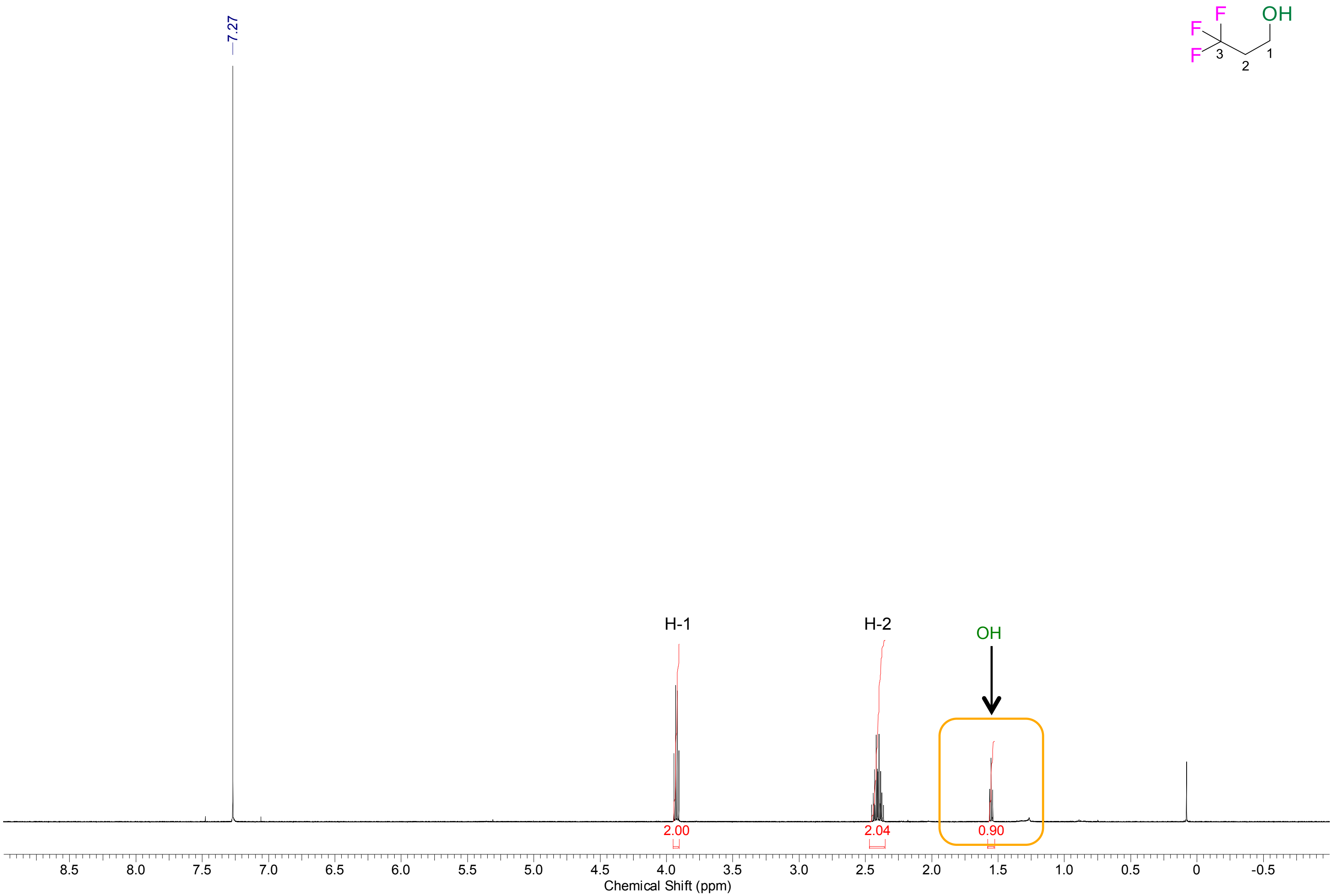

5.11.2 Detail of ^{1}H and $^{1}H[^{19}F]$ NMR of OH signal ($CDCl_3$, 500 MHz, 25 °C) (I)

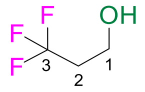

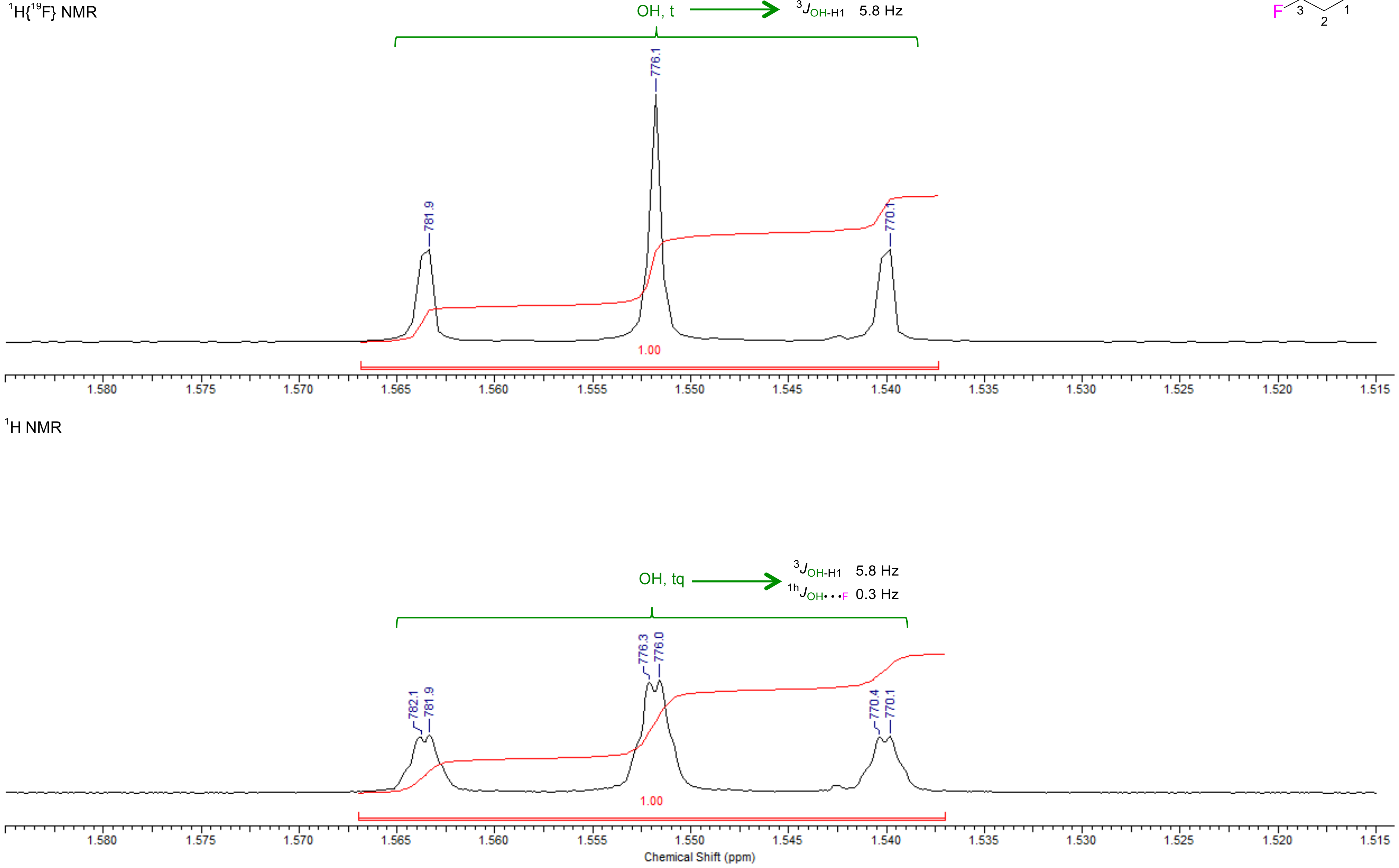

5.11.3 Detail of ^{1}H (enhanced resolution spectra) and $^{1}H[^{19}F]$ NMR of OH signal ($CDCl_3$, 500 MHz, 25 °C) (I)

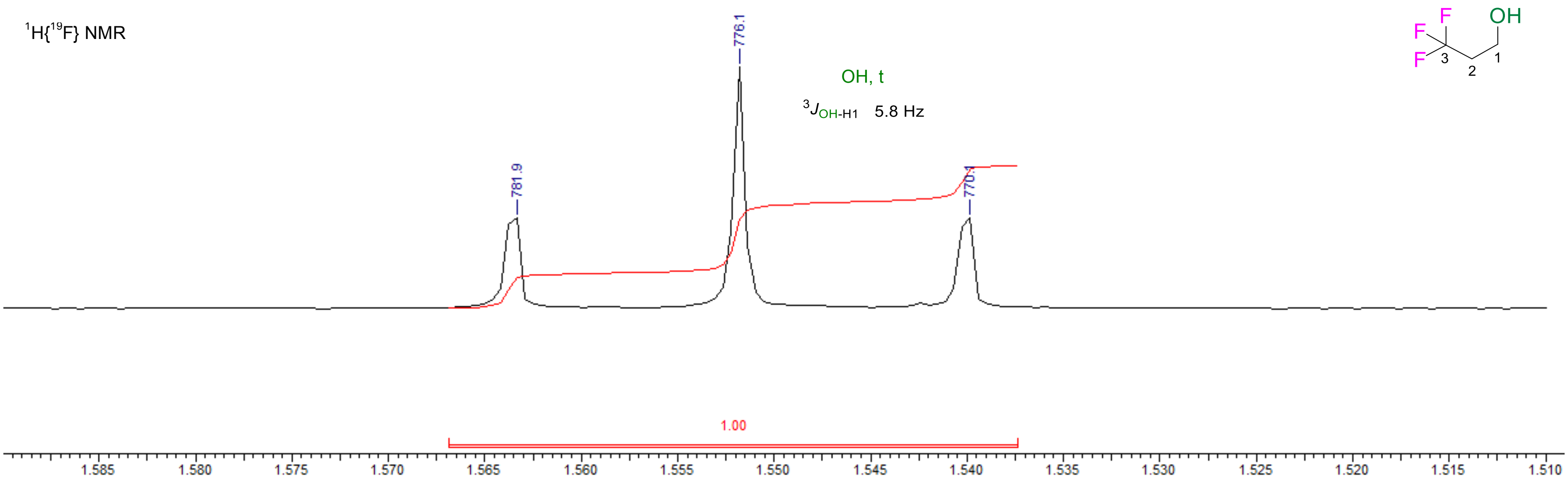

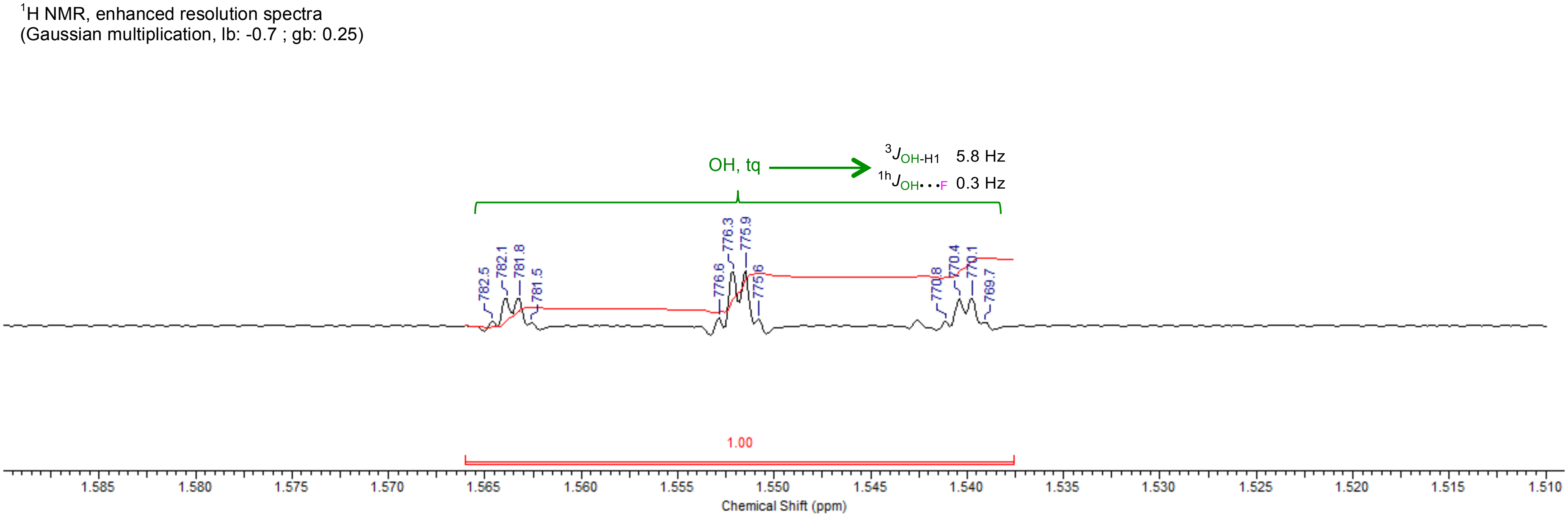

5.11.4 ^{19}F NMR spectrum ($CDCl_3$, 470 MHz, 25 °C) (I)

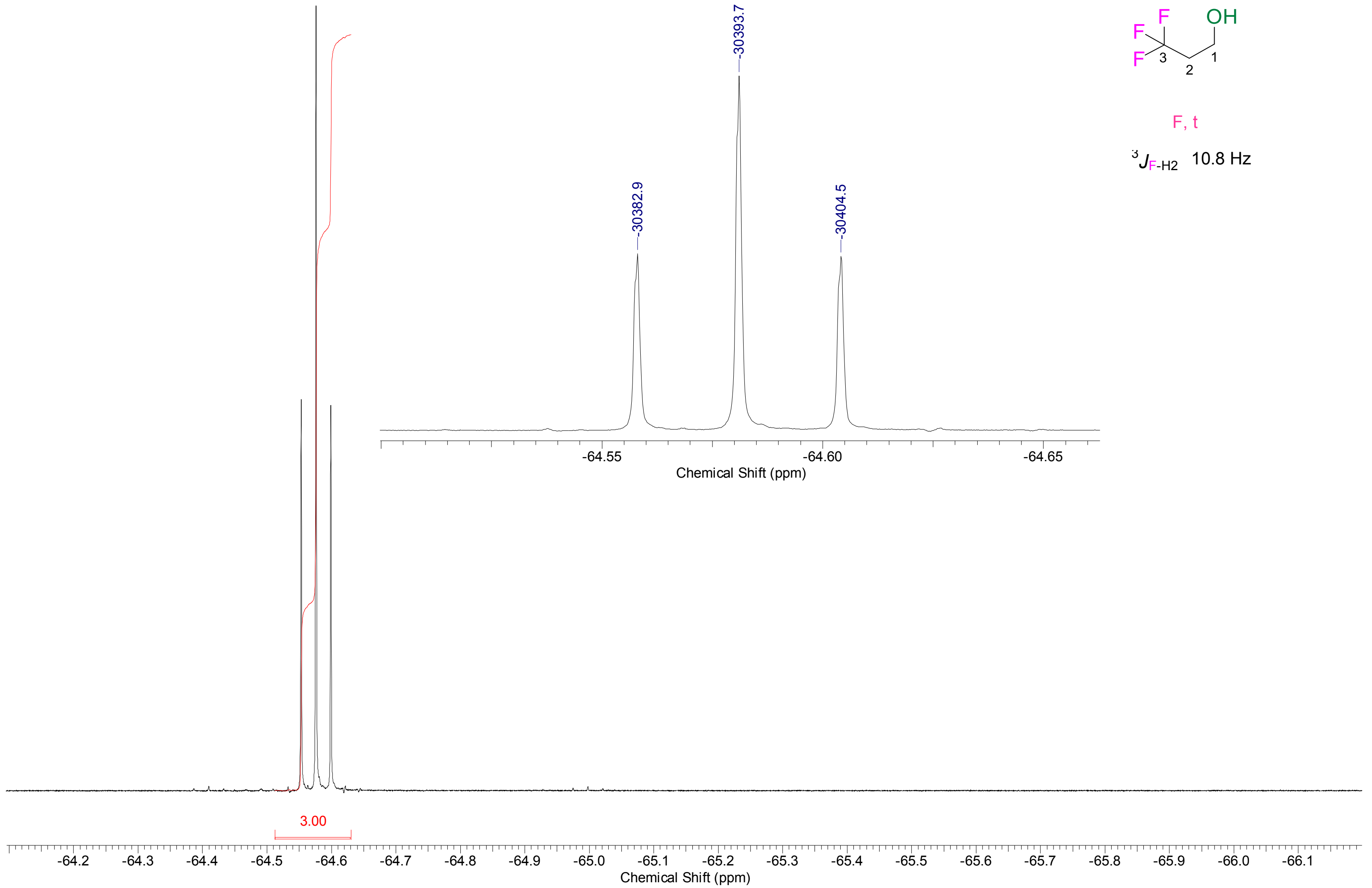

SUPPORTING INFORMATION 3
Synthesis of the fluorohydrins

6 Synthesis of the fluorohydrins 92
6.1 Synthesis of (±)-*syn* and (±)-*anti*-4-fluoropentan-2-ol ((±)-*syn*-A and (±)-*anti*-A) 92
6.1.1 Isolation of *meso*-2,4-pentanediol (*meso*-SI-1) and (±)-2,4-pentanediol ((±)-SI-2) from a commercial mixture of *meso* and *racemic* isomers 92
6.1.1.1 meso-2,4-pentanediol (meso-SI-1) 92
6.1.1.2 (±)-2,4-pentanediol ((±)-SI-2) 92
6.1.2 Synthesis of (±)-*anti* and (±)-*syn-2*-(3'-methylbenzoyloxy)-4-fluoropentane ((±)-*anti*-SI-3 and (±)-*syn*-SI-4) 92
6.1.2.1 General procedure for the mono-deoxyfluorination of diols using DFMBA in diglyme 92
6.1.2.2 (±)-syn-2-(3'-methylbenzoyloxy)-4-fluoropentane (±)-syn-SI-4 93
6.1.2.3 (±)-anti-2-(3'-methylbenzoyloxy)-4-fluoropentane (±)-anti-SI-3 93
6.1.3 Synthesis of fluorohydrins (±)-*syn*-A and (±)-*anti*-A 93
6.1.3.1 General procedure for the deprotection of (±)-anti-SI-3 and (±)-syn-SI-4 93
6.1.3.2 (±)-syn-4-fluoropentan-2-ol (±)-syn-A 94
6.1.3.3 (±)-anti-4-fluoropentan-2-ol (±)-anti-A 95
6.2 Synthesis of (±)-4-fluorobutan-2-ol (±)-B 96
6.2.1 Synthesis of 3-fluoropropanal SI-5 96
6.2.2 Synthesis of (±)-4-fluorobutan-2-ol (±)-B 96
6.3 Synthesis of 3-fluoro-2,2-dimethylpropan1-ol C 97
6.3.1 Synthesis of 3-fluoro-2,2-dimethyl-1-(*meta*-methylbenzoyloxy)-propane SI-6 97
6.3.2 Synthesis of 3-fluoro-2,2-dimethylpropan1-ol C 97
6.4 Synthesis of (±)-4,4-difluoropentan-2-ol (±)-E 98
6.4.1 Synthesis of 4,6-dimethyl-2-phenyl-1,3-dioxane *meso*-SI-7 and (±)-SI-8 98
6.4.2 Synthesis of (2*S**,4*R**)-4-benzyloxypentan-2-ol (±)-*syn*-SI-9 and (2*R**,4*R**)-4-benzyloxypentan-2-ol (±)-*anti*-SI-10 98
6.4.3 Synthesis of (±)-4-benzyloxypentan-2-one (±)-SI-11 99
6.4.4 Synthesis of (±)-2-benzyloxy-4,4-difluoropentane (±)-SI-12 99
6.4.5 Synthesis of (±)-4,4-difluoropentan-2-ol (±)-E 99
6.5 Synthesis of (±)-4,4-difluorobutan-2-ol (±)-F 100
6.5.1 Synthesis of (±)-(2S,4R)-2,4-dimethyl-1,3-dioxane (±)-SI-13 100
6.5.2 Synthesis of (±)-3-benzyloxy-butan-1-ol (±)-SI-14 101
6.5.3 Synthesis of (±)-3-benzyloxy-butanal (±)-SI-15 101
6.5.4 Synthesis of (±)-2-benzyloxy-4,4-difluorobutane (±)-SI-16 101
6.5.5 Synthesis of (±)-4,4-difluorobutan-2-ol (±)-F 102
6.6 Synthesis of 3,3-difluoropropan-1-ol G 102
6.6.1 Synthesis of 1-benzyloxy-3,3-difluoropropane SI-17 102

6.6.2 Synthesis of 3,3-difluoropropan-1-ol G 103

6 Synthesis of the fluorohydrins

6.1 Synthesis of (±)-*syn* and (±)-*anti*-4-fluoropentan-2-ol ((±)-*syn*-A and (±)-*anti*-A)

6.1.1 Isolation of *meso*-2,4-pentanediol (*meso*-SI-1) and (±)-2,4-pentanediol ((±)-SI-2) from a commercial mixture of *meso* and *racemic* isomers

meso*-SI-1** and **(±)-SI-2** were separated from a commercial mixture of *meso*- and *racemic*-2,4-pentanediols (10 g, 96.0 mmol) by flash chromatography (5 to 50% of ethyl acetate in hexane). ***meso*-SI-1** was obtained in pure form as a colorless oil (3.90 g, 37.4 mmol, 39%). The racemic isomer **(±)-SI-2** was contaminated with ***meso*-SI-1** (3.93 g, ratio **(±)-SI-2**/meso*-SI-1** 97:3). To remove traces of ***meso*-SI-1**, the corresponding mixture (3.93 g, 37.7 mmol, 1 equiv.) was treated with thionyl chloride (0.41 mL, 5.66 mmol, 0.15 equiv.) in dichloromethane (100 mL).[1] This mixture was stirred at 0 °C for 1 h and then the solvent was evaporated. As ***meso*-SI-1** reacted with thionyl chloride faster than **(±)-SI-2** to form the cyclic sulfite, after flash chromatography (10 to 50% of ethyl acetate in hexane), **(±)-SI-2** was isolated in pure form as a colorless oil (2.83 g, 27.2 mmol, 29%).

6.1.1.1 meso-2,4-pentanediol (meso-SI-1)

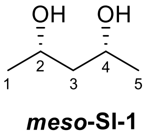

***meso*-SI-1**

IR (neat) 3329(br,m), 2967(s), 2931(m), 1455(w), 1374(w), 1121(s), 919 (m) cm^{-1}. **^{1}H NMR** (400 MHz, $CDCl_3$, 25 °C) δ 4.14–3.91 (m, 2H, H-2, H-4), 3.32 (s, 2H, OH), 1.56 (dt, *J* = 14.4, 3.2 Hz, 1H, H-3), 1.49 (dd, *J* = 14.5, 9.5 Hz, 1H, H-3), 1.20 (d, *J* = 6.2 Hz, 6H, H-1, H-5) ppm. **^{13}C NMR** (101 MHz, $CDCl_3$, 25 °C) δ 68.6 (2C, C-2, C-4), 46.2 (C-3), 23.9 (2C, C-1, C-5) ppm. **MS** (ESI+) *m/z* 105 $[M+H]^+$. **HRMS** (EI) calcd for $C_5H_{12}O_2$ $[M]^{+\cdot}$ 104.0832, found 104.0792. The NMR signals correspond to the literature.[2]

6.1.1.2 (±)-2,4-pentanediol ((±)-SI-2)

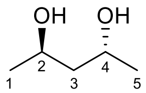

(±)-SI-2

IR (neat) 3325(br,m), 2966(s), 2931(m), 1456(w), 1374(w), 1117(s), 1042(s), 919(m) cm^{-1}. **^{1}H NMR** (400 MHz, $CDCl_3$, 25 °C) δ 4.16 (qdd, *J* = 6.1, 6.1, 5.2 Hz, 2H, H-2, H-4), 2.81 (s, 2H, OH), 1.60 (dd, *J* = 6.1, 5.2 Hz, 2H, H-3), 1.23 (d, *J* = 6.2 Hz, 6H, H-1, H-5) ppm. **^{13}C NMR** (101 MHz, $CDCl_3$, 25 °C) δ 64.7 (2C, C-2, C-4), 45.9 (C-3), 23.2 (2C, C-1, C-5) ppm. **MS** (ESI+) *m/z* 105 $[M+H]^+$. **HRMS** (ESI+) calcd for $C_5H_{12}O_2Na$ $[M+Na]^+$ 127.0730, found 127.0733**.** The NMR signals correspond to the literature.[2]

6.1.2 Synthesis of (±)-*anti* and (±)-*syn-2*-(3'-methylbenzoyloxy)-4-fluoropentane ((±)-*anti*-SI-3 and (±)-*syn*-SI-4)

6.1.2.1 General procedure for the mono-deoxyfluorination of diols using DFMBA in diglyme

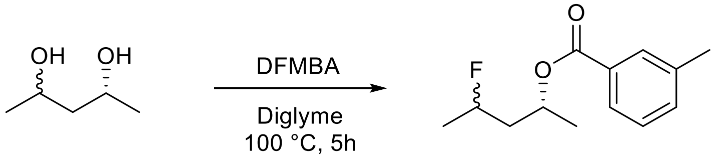

***meso*-SI-1**
(±)-SI-2

(±)-*anti*-SI-3
(±)-*syn*-SI-4

To diol ***meso*-SI-1** or **(±)-SI-2** (1 equiv.) was added a solution of *N,N*-diethyl-α,α-difluoro(*meta*-methylbenzyl)amine (DFMBA, 2 equiv.) in diglyme (0.4 mL/mmol of diol) at room temperature under argon. The reaction mixture was stirred and heated at 100 °C for 5 h under argon. After cooling, the reaction was quenched with an aqueous saturated solution of $NaHCO_3$ (1.1 mL/mmol) and stirred for 20 minutes. Subsequently, water (1.1 mL/mmol) was added to the reaction and the mixture was extracted with diethyl ether three times (2.1

mL/mmol). The combined organic phases were washed with brine (3.5 mL/mmol), dried over $MgSO_4$ and concentrated. The crude mixture was purified by flash chromatography (gradient 0 to 3% of ethyl acetate in hexane) and preparative HPLC (1.5% of ethyl acetate in hexane).

6.1.2.2 (±)-syn-2-(3'-methylbenzoyloxy)-4-fluoropentane (±)-syn-SI-4

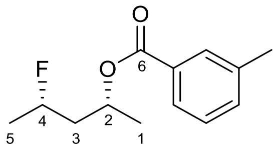

(±)-*syn*-SI-4

Starting from **(±)-SI-2** (2.01 g, 19.3 mmol) using general procedure described above, **(±)-*syn*-SI-4** was obtained as a colorless oil (3.72 g, 16.6 mmol, 86%).

IR (neat) 3022(w), 2978(s), 2938(m), 1712(s), 1457(w), 1299(s), 1199(s),1100(s), 744(s) cm^{-1}. **^1H NMR** (400 MHz, $CDCl_3$, 25 °C) δ 7.92–7.89 (m, 2H, H_{Ar}), 7.40–7.30 (m, 2H, H_{Ar}), 5.36–5.28 (m, 1H, H-2), 4.96–4.76 (m, $^2J_{H4\text{-}F}$ = 48.5 Hz, 1H, H-4), 2.42 (s, 3H, $CH_{3\text{-}Ar}$), 2.28–2.17 (m, 1H, H-3), 1.85 (ddt, *J* = 26.4, 14.4, 5.3 Hz, 1H, H-3'), 1.42 (d, *J* = 6.1 Hz, 3H, H-1), 1.39 (dd, *J* = 24.1, 6.1 Hz, 3H, H-5) ppm. **^{13}C NMR** (101 MHz, $CDCl_3$, 25 °C) δ 166.1 (C-6), 138.1 (C_{qAr}), 133.6 (CH_{Ar}), 130.4 (C_{qAr}), 130.0 (CH_{Ar}), 128.2 (CH_{Ar}), 126.7 (CH_{Ar}), 88.0 (d, *J* = 164 Hz, C-4), 68.5 (d, *J* = 5.1 Hz, C-2), 42.8 (d, *J* = 20.5 Hz, C-3), 21.2 ($CH_{3\text{-}Ar}$), 21.1 (d, *J* = 22.7 Hz, C-5), 20.1 (C-1) ppm. **^{19}F NMR** (376 MHz, $CDCl_3$, 25 °C) –173.3– –172.9 (m) ppm. **MS** (ESI+) *m/z* 225 $[M+H]^+$. **HRMS** (ESI+) calcd for $C_{13}H_{17}FO_2Na$ $[M+Na]^+$ 247.1105, found 247.1109**.** The NMR signals correspond to the literature.[3]

6.1.2.3 (±)-anti-2-(3'-methylbenzoyloxy)-4-fluoropentane (±)-anti-SI-3

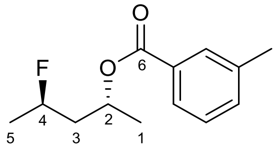

(±)-*anti*-SI-3

Starting from ***meso*-SI-1** (3.01 g, 28.9 mmol), using general procedure described above, **(±)-*anti*-SI-3** was obtained as a colorless oil (4.91 g, 21.9 mmol, 76%).

IR (neat) 3025(w), 2980(s), 2935(m), 1712(s), 1456(w), 1272(s), 1198(s),1101(s), 744(s) cm^{-1}. **^1H NMR** (400 MHz, $CDCl_3$, 25 °C) δ 7.87–7.82 (m, 2H, H_{Ar}), 7.39–7.31 (m, 2H, H_{Ar}), 5.39–5.31 (m, 1H, H-2), 4.92–4.72 (m, $^2J_{H4\text{-}F}$ = 49.4 Hz, 1H, H-4), 2.42 (s, 3H, $CH_{3\text{-}Ar}$), 2.05–1.88 (m, 2H, H-3), 1.40 (d, *J* = 6.2 Hz, 3H, H-1), 1.38 (dd, *J* = 23.7, 6.2 Hz, 3H, H-5) ppm. **^{13}C NMR** (101 MHz, $CDCl_3$, 25 °C) δ 166.1 (C-6), 138.1 (C_{qAr}), 133.6 (CH_{Ar}), 130.5 (C_{qAr}), 130.0 (CH_{Ar}), 128.2 (CH_{Ar}), 126.6 (CH_{Ar}), 87.5 (d, *J*=166 Hz, C-4), 68.3 (d, *J*=3.7 Hz, C-2), 43.5 (d, *J*=20.5 Hz, C-3), 21.4 (d, *J*=22.0 Hz, C-5), 21.2 ($CH_{3\text{-}Ar}$), 20.6 (C-1) ppm. **^{19}F NMR** (376 MHz, $CDCl_3$, 25 °C) –174.8– –174.3 (m) ppm. **MS** (ESI+) *m/z* 225 $[M+H]^+$. **HRMS** (ESI+) calcd for $C_{13}H_{17}FO_2Na$ $[M+Na]^+$ 247.1105, found 247.1104.

6.1.3 Synthesis of fluorohydrins (±)-*syn*-A and (±)-*anti*-A

6.1.3.1 General procedure for the deprotection of (±)-anti-SI-3 and (±)-syn-SI-4

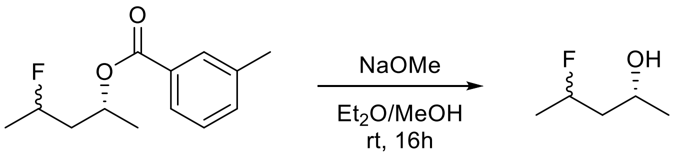

(±)-*anti*-SI-3
(±)-*syn*-SI-4

(±)-*anti*-A
(±)-*syn*-A

To a solution of **(±)-*anti*-SI-3** or **(±)-*syn*-SI-4** (1 equiv.) in dry diethyl ether (2.2 mL/mmol) was added a solution of sodium methoxide in methanol (25 wt. %, 2 equiv.) at 0 °C under argon. The reaction mixture was stirred at room temperature for 16 hours and then neutralized with Amberlite® CG-50. The resin was filtered off and rinsed with diethyl ether (4 mL/mmol). The filtrate was washed two times with an aqueous saturated solution of potassium carbonate (4 mL/mmol), then with brine (4 mL/mmol), dried over $MgSO_4$ and concentrated in vacuo (P>700 mbar). The crude mixture was purified by flash chromatography (gradient 0 to 25% of diethyl ether in pentane).

6.1.3.2 (±)-syn-4-fluoropentan-2-ol (±)-syn-A

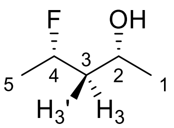

(±)-*syn*-A

Starting from **(±)-*syn*-SI-4** (3.67 g, 16.3 mmol), using general procedure described above, **(±)-*syn*-A** was obtained as a colorless oil (0.861 g, 8.11 mmol, 50%).

IR (neat) 3364(br), 2977(s), 2938(m), 1457(w), 1386(s), 1116(s),1037(s), 918(s), 820(s) cm^{-1}.

^{1}H NMR (500 MHz, $CDCl_3$, 25 °C) δ 4.89 (ddqdd, $^2J_{H4\text{-}F}$ = 49.5 Hz, $^3J_{H4\text{-}H3}$ = 9.0 Hz, $^3J_{H4\text{-}H5}$ = 6.1 Hz, $^3J_{H4\text{-}H3'}$ = 4.0 Hz, J = 0.3 Hz, 1H, H-4), 4.05 (dqdd, $^3J_{H2\text{-}H3}$ = 7.9 Hz, $^3J_{H2\text{-}H1}$ = 6.3 Hz, $^3J_{H2\text{-}H3'}$ = 4.0 Hz, $^3J_{H2\text{-}OH}$ = 3.4 Hz, 1H, H-2), 1.91 (dddd, $^2J_{H3\text{-}H3'}$ = 14.6 Hz, $^3J_{H3\text{-}F}$ = 13.6 Hz, $^3J_{H3\text{-}H4}$ = 9.0 Hz, $^3J_{H3\text{-}H2}$ = 7.9 Hz, 1H, H-3), 1.88 (dd, $^{1h}J_{OH\cdots F}$ = 6.6 Hz, $^3J_{OH\text{-}H2}$ = 3.4 Hz, 1H, OH), 1.64 (ddt, $^3J_{H3'\text{-}F}$ = 34.7 Hz, $^2J_{H3'\text{-}H3}$ = 14.6 Hz, $^3J_{H3'\text{-}H2}$ = 4.0 Hz, $^3J_{H3'\text{-}H4}$ = 4.0 Hz, 1H, H-3'), 1.38 (dd, $^3J_{H5\text{-}F}$ = 24.5 Hz, $^3J_{H5\text{-}H4}$ = 6.1 Hz, 3H, H-5), 1.25 (d, $^3J_{H1\text{-}H2}$ = 6.3 Hz, 3H, H-1) ppm.

^{1}H{^{19}F} NMR (500 MHz, $CDCl_3$, 25 °C) δ 4.89 (dqd, $^3J_{H4\text{-}H3}$ = 9.0 Hz, $^3J_{H4\text{-}H5}$ = 6.1 Hz, $^3J_{H4\text{-}H3'}$ = 4.0 Hz, 1H, H-4), 4.05 (dqdd, $^3J_{H2\text{-}H3}$ = 7.7 Hz, $^3J_{H2\text{-}H1}$ = 6.2 Hz, $^3J_{H2\text{-}H3'}$ = 4.3 Hz, $^3J_{H2\text{-}OH}$ = 3.4 Hz, 1H, H-2), 1.91 (ddd, $^2J_{H3\text{-}H3'}$ = 14.6 Hz, $^3J_{H3\text{-}H4}$ = 9.0 Hz, $^3J_{H3\text{-}H2}$ = 7.9 Hz, 1H, H-3), 1.88 (d, $^3J_{OH\text{-}H2}$ = 3.4 Hz, 1H, OH), 1.64 (dt, $^2J_{H3'\text{-}H3}$ = 14.6 Hz, $^3J_{H3'\text{-}H2}$ = 4.0 Hz, $^3J_{H3'\text{-}H4}$ = 4.0 Hz, 1H, H-3'), 1.38 (d, $^3J_{H5\text{-}H4}$ = 6.1 Hz, 3H, H-5), 1.25 (d, $^3J_{H1\text{-}H2}$ = 6.2 Hz, 3H, H-1) ppm.

^{13}C NMR (101 MHz, $CDCl_3$, 25 °C) δ 90.8 (d, J = 162 Hz, C-4), 66.2 (d, J = 4.4 Hz, C-2), 45.8 (d, J = 19.1 Hz, C-3), 23.4 (C-1), 21.4 (d, J = 22.7 Hz, C-5) ppm.

^{19}F NMR (470 MHz, $CDCl_3$, 25 °C) –173.6 (ddqdd, $^2J_{F\text{-}H4}$ = 49.5 Hz, $^3J_{F\text{-}H3'}$ = 34.7 Hz, $^3J_{F\text{-}H5}$ = 24.5 Hz, $^3J_{F\text{-}H3}$ = 13.6 Hz, $^{1h}J_{F\cdots OH}$ = 6.6 Hz) ppm.

^{19}F{^{1}H} NMR (470 MHz, $CDCl_3$, 25 °C) δ –173.4 (s, 1F) ppm.

^{1}H NMR (500 MHz, $CDCl_3$, –50 °C) δ 4.94 (ddqd, $^2J_{H4\text{-}F}$ = 49.8 Hz, $^3J_{H4\text{-}H3}$ = 9.9 Hz, $^3J_{H4\text{-}H5}$ = 6.2 Hz, $^3J_{H4\text{-}H3'}$ = 3.3 Hz, 1H, H-4), 4.09 (dqdd, $^3J_{H2\text{-}H3}$ = 8.6 Hz, $^3J_{H2\text{-}H1}$ = 6.2 Hz, $^3J_{H2\text{-}H3'}$ = 3.3 Hz, $^3J_{H2\text{-}OH}$ = 2.4 Hz, 1H, H-2), 2.32 (dd, $^{1h}J_{OH\cdots F}$ = 9.9 Hz, $^3J_{OH\text{-}H2}$ = 2.4 Hz, 1H, OH), 1.89 (dddd, $^2J_{H3\text{-}H3'}$ = 14.8 Hz, $^3J_{H3\text{-}F}$ = 11.8 Hz, $^3J_{H3\text{-}H4}$ = 9.9 Hz, $^3J_{H3\text{-}H2}$ = 8.6 Hz, 1H, H-3), 1.65 (ddt, $^3J_{H3'\text{-}F}$ = 39.6 Hz, $^2J_{H3'\text{-}H3}$ = 14.8 Hz, $^3J_{H3'\text{-}H2}$ = 3.3 Hz, $^3J_{H3'\text{-}H4}$ = 3.3 Hz, 1H, H-3'), 1.39 (dd, $^3J_{H5\text{-}F}$ = 25.2 Hz, $^3J_{H5\text{-}H4}$ = 6.2 Hz, 3H, H-5), 1.23 (d, $^3J_{H1\text{-}H2}$ = 6.2 Hz, 3H, H-1) ppm.

^{1}H{^{19}F} NMR (500 MHz, $CDCl_3$, –50 °C) δ 4.94 (dqd, $^3J_{H4\text{-}H3}$ = 9.6 Hz, $^3J_{H4\text{-}H5}$ = 6.3 Hz, $^3J_{H4\text{-}H3'}$ = 3.2 Hz, 1H, H-4), 4.09 (dqdd, $^3J_{H2\text{-}H3}$ = 8.8 Hz, $^3J_{H2\text{-}H1}$ = 6.2 Hz, $^3J_{H2\text{-}H3'}$ = 3.4 Hz, $^3J_{H2\text{-}OH}$ = 2.6 Hz, 1H, H-2), 2.32 (d, $^3J_{OH\text{-}H2}$ = 2.4 Hz, 1H, OH), 1.89 (ddd, $^2J_{H3\text{-}H3'}$ = 14.7 Hz, $^3J_{H3\text{-}H4}$ = 9.8 Hz, $^3J_{H3\text{-}H2}$ = 8.5 Hz, 1H, H-3), 1.65 (dt, $^2J_{H3'\text{-}H3}$ = 14.7 Hz, $^3J_{H3'\text{-}H2}$ = 3.3 Hz, $^3J_{H3'\text{-}H4}$ = 3.3 Hz, 1H, H-3'), 1.39 (d, $^3J_{H5\text{-}H4}$ = 6.2 Hz, 3H, H-5), 1.23 (d, $^3J_{H1\text{-}H2}$ = 6.2 Hz, 3H, H-1) ppm.

^{19}F NMR (470 MHz, $CDCl_3$, –50 °C) –173.7 (ddqdd, $^2J_{F\text{-}H4}$ = 49.8 Hz, $^3J_{F\text{-}H3'}$ = 39.6 Hz, $^3J_{F\text{-}H5}$ = 25.2 Hz, $^3J_{F\text{-}H3}$ = 11.8 Hz, $^{1h}J_{F\cdots OH}$ = 9.9 Hz) ppm.

^{19}F{^{1}H} NMR (470 MHz, $CDCl_3$, –50 °C) δ –173.7 (s, 1F) ppm.

HRMS (EI) calcd for $C_5H_{11}OF$ $[M]^{+\cdot}$ 106.0788, found 106.0743**.**

6.1.3.3 (±)-anti-4-fluoropentan-2-ol (±)-anti-A

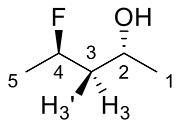

(±)-*anti*-A

Starting from **(±)-*anti*-SI-3** (1.99 g, 8.87 mmol), using general procedure described above, **(±)-*anti*-A** was obtained as a colorless oil (0.545 g, 5.13 mmol, 58%).

IR (neat) 3357(br), 2976(m), 2936(m), 1457(w), 1378(s), 1145(s),1044(s), 912(m), 816(s) cm^{-1}.

^{1}H NMR (500 MHz, $CDCl_3$, 25 °C) δ 4.95 (ddqdd, $^{2}J_{H4\text{-}F}$ = 49.4 Hz, $^{3}J_{H4\text{-}H3}$ = 9.3 Hz, $^{3}J_{H4\text{-}H5}$ = 6.2 Hz, $^{3}J_{H4\text{-}H3'}$ = 2.7 Hz, J = 0.3 Hz, 1H, H-4), 4.09 (dqddd, $^{3}J_{H2\text{-}H3'}$ = 9.3 Hz, $^{3}J_{H2\text{-}H1}$ = 6.3 Hz, $^{3}J_{H2\text{-}OH}$ = 4.9 Hz, $^{3}J_{H2\text{-}H3}$ = 3.1 Hz, J = 0.3 Hz, 1H, H-2), 1.77 (ddddd, $^{3}J_{H3\text{-}F}$ = 15.7 Hz, $^{2}J_{H3\text{-}H3'}$ = 14.7 Hz, $^{3}J_{H3\text{-}H4}$ = 9.3 Hz, $^{3}J_{H3\text{-}H2}$ = 3.1 Hz, $^{4}J_{H3\text{-}OH}$ = 0.5 Hz, 1H, H-3), 1.62 (dddd, $^{3}J_{H3'\text{-}F}$ = 36.0 Hz, $^{2}J_{H3'\text{-}H3}$ = 14.7 Hz, $^{3}J_{H3'\text{-}H2}$ = 9.4 Hz, $^{3}J_{H3'\text{-}H4}$ = 2.7 Hz, 1H, H-3'), 1.56 (ddd, $^{3}J_{OH\text{-}H2}$ = 4.9 Hz, $^{1h}J_{OH\cdots F}$ = 1.9 Hz, $^{4}J_{OH\text{-}H3}$ = 0.5 Hz, 1H, OH), 1.37 (dd, $^{3}J_{H5\text{-}F}$ = 24.0 Hz, $^{3}J_{H5\text{-}H4}$ = 6.2 Hz, 3H, H-5), 1.25 (dd, $^{3}J_{H1\text{-}H2}$ = 6.3 Hz, $^{5}J_{H1\text{-}F}$ = 0.4 Hz, 3H, H-1) ppm.

^{1}H{^{19}F}NMR (500 MHz, $CDCl_3$, 25 °C) δ 4.95 (dqdd, $^{3}J_{H4\text{-}H3}$ = 9.0 Hz, $^{3}J_{H4\text{-}H5}$ = 6.2 Hz, $^{3}J_{H4\text{-}H3'}$ = 2.6 Hz, J = 0.3 Hz, 1H, H-4), 4.09 (dqdd, $^{3}J_{H2\text{-}H3'}$ = 9.3 Hz, $^{3}J_{H2\text{-}H1}$ = 6.3 Hz, $^{3}J_{H2\text{-}OH}$ = 4.9 Hz, $^{3}J_{H2\text{-}H3}$ = 3.0 Hz, 1H, H-2), 1.77 (dddd, $^{2}J_{H3\text{-}H3'}$ = 14.7 Hz, $^{3}J_{H3\text{-}H4}$ = 9.4 Hz, $^{3}J_{H3\text{-}H2}$ = 3.0 Hz, $^{4}J_{H3\text{-}OH}$ = 0.5 Hz, 1H, H-3), 1.62 (ddd, $^{2}J_{H3'\text{-}H3}$ = 14.7 Hz, $^{3}J_{H3'\text{-}H2}$ = 9.3 Hz, $^{3}J_{H3'\text{-}H4}$ = 2.7 Hz, 1H, H-3'), 1.56 (ddd, $^{3}J_{OH\text{-}H2}$ = 4.9 Hz, $^{4}J_{OH\text{-}H3}$ = 0.5 Hz, 1H, OH), 1.37 (d, $^{3}J_{H5\text{-}H4}$ = 6.2 Hz, 3H, H-5), 1.25 (d, $^{3}J_{H1\text{-}H2}$ = 6.3 Hz, 3H, H-1) ppm.

^{13}C NMR (101 MHz, $CDCl_3$, 25 °C) δ 88.3 (d, J = 163 Hz, C-4), 64.3 (d, J = 2.9 Hz, C-2), 45.9 (d, J = 19.8 Hz, C-3), 24.0 (C-1), 21.3 (d, J = 22.7 Hz, C-5) ppm.

^{19}F NMR (470 MHz, $CDCl_3$, 25 °C) –175.4 (ddqdd, $^{2}J_{F\text{-}H4}$ = 49.4 Hz, $^{3}J_{F\text{-}H3'}$ = 36.0 Hz, $^{3}J_{F\text{-}H5}$ = 24.0 Hz, $^{3}J_{F\text{-}H3}$ = 15.7 Hz, $^{1h}J_{F\cdots OH}$ = 1.9 Hz, ($^{5}J_{F\text{-}H1}$ = 0.40 Hz not resolved)) ppm.

^{19}F{^{1}H} NMR (470 MHz, $CDCl_3$, 25 °C) δ –175.4 (s, 1F) ppm.

^{1}H NMR (500 MHz, $CDCl_3$, –50 °C) δ 4.95 (ddqd, $^{2}J_{H4\text{-}F}$ = 49.7 Hz, $^{3}J_{H4\text{-}H3}$ = 9.9 Hz, $^{3}J_{H4\text{-}H5}$ = 6.3 Hz, $^{3}J_{H4\text{-}H3'}$ = 2.3 Hz, H-4), 4.10 (dqdd, $^{3}J_{H2\text{-}H3'}$ = 10.0 Hz, $^{3}J_{H2\text{-}H1}$ = 6.3 Hz, $^{3}J_{H2\text{-}OH}$ = 4.7 Hz, $^{3}J_{H2\text{-}H3}$ = 2.5 Hz, 1H, H-2), 1.78 (ddd, $^{3}J_{OH\text{-}H2}$ = 4.7 Hz, $^{1h}J_{OH\cdots F}$ = 1.8 Hz, $^{4}J_{OH\text{-}H3}$ = 0.7 Hz, 1H, OH),1.76 (ddddd appears as tddd, $^{3}J_{H3\text{-}F}$ = 14.7 Hz, $^{2}J_{H3\text{-}H3'}$ = 14.7 Hz, $^{3}J_{H3\text{-}H4}$ = 9.9 Hz, $^{3}J_{H3\text{-}H2}$ = 2.5 Hz, $^{4}J_{H3\text{-}OH}$ = 0.7 Hz, 1H, H-3), 1.60 (dddd, $^{3}J_{H3'\text{-}F}$ = 39.1 Hz, $^{2}J_{H3'\text{-}H3}$ = 14.9 Hz, $^{3}J_{H3'\text{-}H2}$ = 10.0 Hz, $^{3}J_{H3'\text{-}H4}$ = 2.3 Hz, 1H, H-3'), 1.37 (dd, $^{3}J_{H5\text{-}F}$ = 24.5 Hz, $^{3}J_{H5\text{-}H4}$ = 6.3 Hz, 3H, H-5), 1.24 (d, $^{3}J_{H1\text{-}H2}$ = 6.3 Hz, 3H, H-1) ppm.

^{1}H{^{19}F} NMR (500 MHz, $CDCl_3$, –50 °C) δ 4.98 (dqd, $^{3}J_{H4\text{-}H3}$ = 10.0 Hz, $^{3}J_{H4\text{-}H5}$ = 6.2 Hz, $^{3}J_{H4\text{-}H3'}$ = 2.1 Hz, H-4), 4.11 (dqdd, $^{3}J_{H2\text{-}H3'}$ = 10.0 Hz, $^{3}J_{H2\text{-}H1}$ = 6.3 Hz, $^{3}J_{H2\text{-}OH}$ = 4.7 Hz, $^{3}J_{H2\text{-}H3}$ = 2.5 Hz, 1H, H-2), 1.78 (dd, $^{3}J_{OH\text{-}H2}$ = 4.7 Hz, $^{4}J_{OH\text{-}H3}$ = 0.7 Hz, 1H, OH),1.76 (dddd, $^{2}J_{H3\text{-}H3'}$ = 14.6 Hz, $^{3}J_{H3\text{-}H4}$ = 9.9 Hz, $^{3}J_{H3\text{-}H2}$ = 2.6 Hz, $^{4}J_{H3\text{-}OH}$ = 0.7 Hz, 1H, H-3), 1.60 (ddd, $^{2}J_{H3'\text{-}H3}$ = 14.8 Hz, $^{3}J_{H3'\text{-}H2}$ = 10.0 Hz, $^{3}J_{H3'\text{-}H4}$ = 2.4 Hz, 1H, H-3'), 1.37 (d, $^{3}J_{H5\text{-}H4}$ = 6.4 Hz, 3H, H-5), 1.24 (d, $^{3}J_{H1\text{-}H2}$ = 6.2 Hz, 3H, H-1) ppm.

^{19}F NMR (470 MHz, $CDCl_3$, –50 °C) –176.4 (ddqdd, $^{2}J_{F\text{-}H4}$ = 49.7 Hz, $^{3}J_{F\text{-}H3'}$ = 39.1 Hz, $^{3}J_{F\text{-}H5}$ = 24.5 Hz, $^{3}J_{F\text{-}H3}$ = 14.7 Hz, $^{1h}J_{F\cdots OH}$ = 1.8 Hz) ppm.

^{19}F{^{1}H} NMR (470 MHz, $CDCl_3$, –50 °C) δ –176.4 (s, 1F) ppm.

HRMS (EI) calcd for $C_5H_{11}OF$ $[M]^{+\cdot}$ 106.0788, found 106.0745**.**

6.2 Synthesis of (±)-4-fluorobutan-2-ol (±)-B

6.2.1 Synthesis of 3-fluoropropanal SI-5

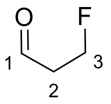

SI-5

Trichloroisocyanuric acid (4.58 g, 19.7 mmol, 0.37 equiv) was added to a vigorously stirred mixture of 3-fluoropropan-1-ol (4.0 mL, 53.3 mmol, 1 equiv), $NaHCO_3$ (4.46 g, 53.3 mmol, 1 equiv), and TEMPO (0.091 g, 0.586 mmol, 1.1 mol%) in CH_2Cl_2 (80 mL) and water (2.7 mL). The temperature was kept at 20~25 ^{0}C (water bath). After completion of the addition, stirring was continued until the orange color faded to a pale yellow (± 30 min). The resulting solution was decanted from the gummy white residue, filtered through a pad of silica gel (1.33 g), and dried over $MgSO_4$ (2.66 g) for 30 min. This provided a solution of 3-fluoropropanal in CH_2Cl_2, the concentration of which was determined by ^{1}H-NMR using the formula: [3-fluoropropanal] = (integral of δ 9.83)/(integral of δ 5.28)*32. This procedure provided an approximately 0.4 M solution of 3-fluoropropanal **SI-5** in CH_2Cl_2, which was used without further purification.

^{1}H NMR (400 MHz, $CDCl_3$) δ 9.84 (td, $^3J_{HH}$ = 1.5, $^4J_{HF}$ = 1.2 Hz, 1H, H-1), 4.81 (dt, $^2J_{HF}$ = 46.5, $^3J_{HH}$ = 5.9 Hz, 2H, H-3), 2.86 (dtd, $^3J_{HF}$ = 25.7, $^3J_{HH}$ = 5.9, $^3J_{HH}$ = 1.5 Hz, 2H, H-2) ppm. The spectral data matched with the literature.[4] **^{19}F NMR** (376 MHz, $CDCl_3$) δ –221.2 (tt, $^2J_{HF}$ = 46.5, $^3J_{HF}$ = 25.7 Hz, 1F) ppm ($^4J_{HF}$ not visible).

6.2.2 Synthesis of (±)-4-fluorobutan-2-ol (±)-B

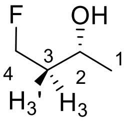

(±)-B

To a solution of **SI-5** in CH_2Cl_2 (0.4 M, 20 mL, 8 mmol, 1 equiv) at –78 °C was added dropwise a solution of MeMgBr in Et_2O (3 M, 2.8 mL, 8.4 mmol, 1.05 equiv) and the resulting mixture was stirred at – 78 °C for 1.5 h. The reaction mixture was then quenched with sat. aq. NH_4Cl (15 mL) and allowed to warm to room temperature. Et_2O (25 mL) was added and layers were separated. The aqueous phase was extracted with Et_2O (2 × 45 mL) then the combined organic layers were dried ($MgSO_4$), filtered and evaporated at 31 °C for ≈670 mbar. The crude product was purified by column chromatography on silica gel eluting with pentane/Et_2O (80:20 to 70:30) to give after evaporation (±)-4-fluorobutan-2-ol **(±)-B** together with pentane and Et_2O. The mass of product was calculated by ^{1}H NMR to give ≈460 mg (4.99 mmol, 62%). This fraction was combined with another fraction obtained similarly from a 25.7 mmol scale reaction and pentane and Et_2O were distilled off to give 663 mg (7.20 mmol, 21%) of pure (±)-4-fluorobutan-2-ol **(±)-B** as a pale yellow oil.

R_f 0.13 (CH_2Cl_2). **IR** (neat) 3355 (br m), 2971 (m), 1377 (w), 1137 (m), 1041 (s) cm^{-1}.

^{1}H NMR (500 MHz, $CDCl_3$, 25 °C) δ 4.66 (ddddd, $^2J_{H4\text{-}F}$ = 47.2, $^2J_{H4\text{-}H4'}$ = 9.3, $^3J_{H4\text{-}H3}$ = 7.5, $^3J_{H4\text{-}H3'}$ = 4.6, J_{HH} = 0.3 Hz, 1H, H-4), 4.59 (ddddd, $^2J_{H4'\text{-}F}$ = 47.2, $^2J_{H4'\text{-}H4}$ = 9.3, $^3J_{H4'\text{-}H3'}$ = 6.0, $^3J_{H4'\text{-}H3}$ = 5.0, J_{HH} = 0.3 Hz, 1H, H-4'), 4.10 – 4.02 (m, 1H, H-2), 1.88 (dddddd, $^3J_{H3'\text{-}F}$ = 25.0, $^2J_{H3'\text{-}H3}$ = 14.8, $^3J_{H3'\text{-}H4}$ = 7.5, $^3J_{H3'\text{-}H4'}$ = 5.0, $^3J_{H3'\text{-}H2}$ = 4.3, $^4J_{H3'\text{-}HO}$ = 0.3 Hz, 1H, H-3'), 1.82 (ddddd, $^3J_{H3\text{-}F}$ = 29.3, $^2J_{H3\text{-}H3'}$ = 14.8, $^3J_{H3\text{-}H2}$ = 8.1, $^3J_{H3\text{-}H4'}$ = 6.0, $^3J_{H3\text{-}H4}$ = 4.6 Hz, 1H, H-3), 1.54 (ddd, $^3J_{OH\text{-}H2}$ = 4.5, $^{1h}J_{OH\cdots F}$ = 2.2, $^4J_{OH\text{-}H3'}$ = 0.3 Hz, 1H, OH), 1.27 (dd, $^3J_{H1\text{-}H2}$ = 6.3, $^5J_{H1\text{-}F}$ = 0.5 Hz, 3H, H-1) ppm.

^{1}H{^{19}F} NMR (500 MHz, $CDCl_3$, 25 °C) δ 4.66 (dddd, $^2J_{H4\text{-}H4'}$ = 9.3, $^3J_{H4\text{-}H3}$ = 7.5, $^3J_{H4\text{-}H3'}$ = 4.6, J_{HH} = 0.3 Hz, 1H, H-4), 4.59 (dddd, $^2J_{H4'\text{-}H4}$ = 9.3, $^3J_{H4'\text{-}H3'}$ = 6.0, $^3J_{H4'\text{-}H3}$ = 5.0, J_{HH} = 0.3 Hz, 1H, H-4'), 4.10 – 4.02 (m, 1H, H-2), 1.88 (ddddd, $^2J_{H3'\text{-}H3}$ = 14.8, $^3J_{H3'\text{-}H4}$ = 7.5, $^3J_{H3'\text{-}H4'}$ = 5.0, $^3J_{H3'\text{-}H2}$ = 4.3, $J_{H3'\text{-}HO}$ = 0.3 Hz, 1H, H-3'), 1.82 (dddd, $^2J_{H3\text{-}H3'}$ = 14.8, $^3J_{H3\text{-}H2}$ = 8.1, $^3J_{H3\text{-}H4'}$ = 6.0, $^3J_{H3\text{-}H4}$ = 4.6 Hz, 1H, H-3), 1.54 (dd, $^3J_{H2\text{-}OH}$ = 4.5, $J_{OH\text{-}H3'}$ = 0.3 Hz, 1H, OH), 1.27 (d, $^3J_{H1\text{-}H2}$ = 6.3, 3H, H-1) ppm.

^{13}C NMR (101 MHz, $CDCl_3$, 25 °C) δ 81.9 (d, $^1J_{CF}$ = 162.1 Hz, C-4), 65.0 (d, $^3J_{CF}$ = 4.4 Hz, C-2), 39.3 (d, $^2J_{CF}$ = 19.1 Hz, C-3), 23.7 (s, C-1) ppm.

^{19}F NMR (470 MHz, $CDCl_3$, 25°C) δ –220.8 (tdddq, $^2J_{F\text{-}H4}$ = 47.2, $^2J_{F\text{-}H3'}$ = 29.3, $^2J_{F\text{-}H3}$ = 25.0, $^{1h}J_{F\cdots HO}$ = 2.2, $^5J_{F\text{-}H1}$ = 0.5 Hz, 1F) ppm.

^{19}F{^{1}H} NMR (470 MHz, $CDCl_3$, 25°C) δ –220.8 (s, 1F) ppm.

6.3 Synthesis of 3-fluoro-2,2-dimethylpropan1-ol C

6.3.1 Synthesis of 3-fluoro-2,2-dimethyl-1-(*meta*-methylbenzoyloxy)-propane SI-6

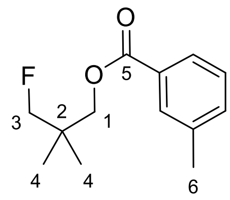

SI-6

In a microwave tube under argon was placed neopentyl glycol (208 mg, 2.0 mmol, 1 equiv) and dry diglyme (2.5 mL) and the mixture was gently heated until complete dissolution. DFMBA (852 mg, 4.0 mmol, 2 equiv) and NaF (84 mg, 2.0 mmol, 1 equiv) were then added and the resulting mixture was heated at 200 °C under microwave irradiation for 5 min. The mixture was then diluted with Et_2O (60 mL), washed successively with sat. aq. $NaHCO_3$ (20 mL), 1 M aq. HCl (20 mL) and sat. aq. $NaHCO_3$ (10 mL) then dried ($MgSO_4$), filtered and concentrated. Column chromatography eluting with petroleum ether 40-60 °C/Et_2O (99:1 to 98:2) afforded 387 mg (1.73 mmol, 86%) of the desired fluoroester **SI-6** as a colorless oil.

^{1}H NMR (400 MHz, $CDCl_3$, 25°C) δ 7.89 – 7.81 (m, 2H, H_{Ar}), 7.43 – 7.30 (m, 2H, H_{Ar}), 4.30 (d, $^{2}J_{H3\text{-}F}$ = 47.7 Hz, 2H, H-3), 4.18 (d, $^{4}J_{H1\text{-}F}$ = 1.0 Hz, 2H, H-1), 2.42 (s, 3H, H-6), 1.08 (d, $^{4}J_{H4\text{-}F}$ = 1.7 Hz, 6H, H-4) ppm. **^{13}C NMR** (101 MHz, $CDCl_3$, 25°C) δ 166.5 (C-5), 138.2 ($C_{q,Ar}$), 133.8 (CH_{Ar}), 130.11 ($C_{q,Ar}$), 130.06 (CH_{Ar}), 128.3 (CH_{Ar}), 126.6 (CH_{Ar}), 88.2 (d, $^{1}J_{C3\text{-}F}$ = 173.9 Hz, C-3), 68.9 (d, $^{3}J_{C1\text{-}F}$ = 3.7 Hz, C-1), 36.0 (d, $^{2}J_{C2\text{-}F}$ = 16.9 Hz, C-2), 21.3 (C-6), 20.8 (d, $^{3}J_{C4\text{-}F}$ = 5.1 Hz, C-4) ppm. **^{19}F NMR** (376 MHz, $CDCl_3$, 25°C) δ -226.5 (br t, $^{2}J_{F\text{-}H3}$ = 47.7 Hz) ppm. The spectral data matched with the literature.[3]

6.3.2 Synthesis of 3-fluoro-2,2-dimethylpropan1-ol C

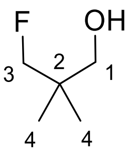

C

To a solution of **SI-6** (900 mg, 4.01 mmol, 1 equiv) in dry Et_2O (8 mL) was added a solution of MeONa (25% wt, 1.84 mL, 8.02 mmol, 2 equiv). After being stirred at room temperature for 22.5 h, the reaction was neutralized with aq. HCl (1.0 M, 8 mL) and extracted with Et_2O (3 × 24 mL). The combined organic phases were dried and filtered. The Et_2O was distilled off at 50 °C and the crude product was purified by column chromatography on silica gel eluting with CH_2Cl_2 to give after evaporation at ≈ 750 mbar and 28 °C, 290 mg (2.73 mmol, 68%) of the desired product **C** as a white solid.

R_f 0.21 (CH_2Cl_2)

^{1}H NMR (500 MHz, $CDCl_3$, 25°C) δ 4.24 (d, $^{2}J_{H3\text{-}F}$ = 47.8 Hz, 2H, H-3), 3.48 (dd, $^{3}J_{H1\text{-}OH}$ = 5.9, $^{4}J_{H1\text{-}F}$ = 1.3 Hz, 2H, H-1), 1.45 (td, $^{3}J_{OH\text{-}H1}$ = 5.9, $^{1h}J_{OH\cdots F}$ = 1.7 Hz, 1H, OH), 0.95 (d, $^{4}J_{H4\text{-}F}$ = 1.8 Hz, 6H, H-4) ppm.

^{1}H{^{19}F} NMR (500 MHz, $CDCl_3$, 25°C) δ 4.24 (s, 2H, H-3), 3.48 (d, $^{3}J_{H1\text{-}OH}$ = 5.9 Hz, 2H, H-1), 1.45 (t, $^{3}J_{OH\text{-}H1}$ = 5.9 Hz, 1H, OH), 0.95 (s, 6H, H-4) ppm.

^{13}C NMR (101 MHz, $CDCl_3$, 25°C) δ 89.0 (d, $^{1}J_{C3\text{-}F}$ = 171.7 Hz, C-3), 68.4 (d, $^{3}J_{C1\text{-}F}$ = 3.7 Hz, C-1), 36.9 (d, $^{2}J_{C2\text{-}F}$ = 16.9 Hz, C-2), 20.3 (d, $^{3}J_{C4\text{-}F}$ = 5.1 Hz, C-4) ppm.

^{19}F NMR (470 MHz, $CDCl_3$, 25°C) δ –226.4 – –226.6(m, $^{2}J_{F\text{-}H3}$ = 47.8, 1F) ppm.

^{19}F{^{1}H} NMR (470 MHz, $CDCl_3$, 25°C) δ –226.5 (s, 1F) ppm.

6.4 Synthesis of (±)-4,4-difluoropentan-2-ol (±)-E

6.4.1 Synthesis of 4,6-dimethyl-2-phenyl-1,3-dioxane *meso*-SI-7 and (±)-SI-8

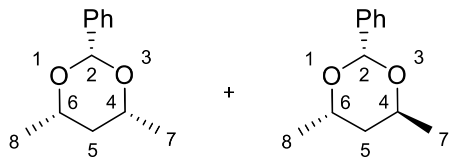

***meso*-SI-7** **(±)-SI-8**

A solution of benzaldehyde (10.1 g, 96.0 mmol, 1 equiv), a mixture of the 2,4-pentanediol diastereomers (10.0 g, 96.0 mmol, 1 equiv), and *p*-toluenesulfonic acid monohydrate (193 mg, 0.96 mmol, 1 mol%) in dry toluene (200 mL) was heated at reflux with a Dean-Stark trap for 6 h. After cooling, the solution was diluted with 80 mL of Et_2O and washed with sat. aq. $NaHCO_3$ (80 mL). The aqueous layer was extracted with Et_2O (2 × 80 mL), and the combined organic extracts were dried over $MgSO_4$, filtered, and concentrated under vacuum to give 19.0 g of the crude acetal mixture together with 7% of toluene which was used without further purification.

R_f 0.76 (petroleum ether 40–60 °C/Et_2O 80:20). **^{1}H NMR** (400 MHz, $CDCl_3$) δ 7.48 – 7.56 (m, 4H, H_{Ar}), 7.40 – 7.29 (m, 6H, H_{Ar}), 5.84 (s, 1H, H-2 (±)), 5.54 (s, 1H, H-2 meso), 4.48 (app. quin, *J* = 6.8 Hz, 1H, H-4 (±)), 4.20 (dqd, *J* = 11.9, 6.1, 2.4 Hz, 1H, H-6 (±)), 3.96 (dqd, *J* = 11.3, 6.1, 2.4 Hz, 2H, H-4 + H-6 meso), 2.01 (ddd, *J* = 13.2, 12.0, 6.1 Hz, 1H, H-5_{ax} (±)), 1.63 (dt, *J* = 13.2, 2.4 Hz, 1H, H-5_{eq} meso), 1.50 (d, *J* = 6.8 Hz, 3H, H-8 (±)), 1.45 (ddd, *J* = 13.2, 2.4, 1 Hz, 1H, H-5_{eq} (±)), 1.41 (dt, *J* = 13.2, 11.3 Hz, H-5_{ax} meso), 1.32 (d, *J* = 6.1 Hz, 6H, H-7 + H-8 meso), 1.30 (d, *J* = 6.1 Hz, 3H, H-7 (±)) ppm. **^{13}C NMR** (101 MHz, $CDCl_3$, 25°C) δ 139.1 ($C_{q,Ar}$ (±)), 138.9 ($C_{q,Ar}$ meso), 128.6 (2 × CH_{Ar}), 128.2 (4 × CH_{Ar}), 126.22 (2 × CH_{Ar}), 126.17 (2 × CH_{Ar}), 100.9 (C-2 meso), 94.0 (C-2 (±)), 73.0 (2C, C-4 + C-6 meso), 68.6 (C-6 (±)), 68.0 (C-4 (±)), 40.3 (C-5 meso), 36.7 (C-5 (±)), 21.9 (C-8 (±)), 21.6 (2C, C-7 + C-8 meso), 17.2 (C-7 (±)) ppm. The spectral data matched with the literature.[5]

6.4.2 Synthesis of (2*S**,4*R**)-4-benzyloxypentan-2-ol (±)-*syn*-SI-9 and (2*R**,4*R**)-4-benzyloxypentan-2-ol (±)-*anti*-SI-10

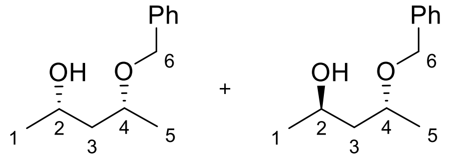

(±)-*syn*-SI-9 **(±)-*anti*-SI-10**

Diisobutylaluminium hydride (1.0 M in hexane, 52 mL, 52 mmol, 2 equiv) was added dropwise to a solution of the crude mixture of ***meso*-SI-7** and **(±)-SI-8** obtained above in dry CH_2Cl_2 at 0 °C. After stirring at 0 °C for 2 h and then at room temperature for 17 h, EtOAc (150 mL) was added at 0 °C. After stirring for 0.5 h, aq. NaOH (3 M, 300 mL) and Et_2O (125 mL) were added. The phases were separated and the aqueous phase was extracted with Et_2O (3 × 125 mL). The combined organic phases were washed with brine (150 mL), dried ($MgSO_4$) and concentrated. Column chromatography on silica gel (pentane/Et_2O 80:20 to 40:60) gave 1.17 g (6.02 mmol, 23%) of a mixture of the desired alcohols as a colorless oil. A second fraction consisting of 2.73 g of the corresponding 2-*O*-acetylated products was treated with a catalytic amount of MeONa in dry MeOH for 21.5 h. The reaction mixture was neutralized with Amberlite IR120 and evaporated to give 2.29 g (11.6 mmol, 44%) of a mixture of the desired alcohols **(±)-*syn*-SI-9** and **(±)-*anti*-SI-10** as a colorless oil leading to a combined yield of 67%.

R_f 0.12 (petroleum ether 40–60 °C/Et_2O 80:20). **^{1}H NMR** (400 MHz, $CDCl_3$, 25°C) δ 7.40 – 7.28 (m, 10H, H_{Ar}), 4.68 (d, *J* = 11.5 Hz, 1H, H-6 **SI-9**), 4.64 (d, *J* = 11.5 Hz, 1H, H-6 **SI-10**), 4.47 (d, *J* = 11.5 Hz, 1H, H-6' **SI-10**), 4.44 (d, *J* = 11.5 Hz, 1H, H-6 **SI-9**), 4.19 – 4.08 (m, 1H, H-2 or H-4 **SI-10**), 4.04 – 3.95 (m, 1H, H-2 or H-4 **SI-9**), 3.92 – 3.77 (m, 2H, H-2 or H-4, **SI-9** + **SI-10**), 3.64 (br. s, 1H, OH **SI-9**), 2.71 (dd, *J*=3.4, 2.0 Hz, 1H, OH **SI-10**), 1.76 – 1.53 (m, 4H, 2 × H-3, **SI-9** + **SI-10**), 1.28 (d, *J* = 6.1 Hz, 3H, H-1 or H-5, **SI-10**), 1.25 (d, *J* = 6.1 Hz, 3H, H-1 or H-5, **SI-9**), 1.19 (d, *J* = 6.4 Hz, 3H, H-1 or H-5, **SI-10**), 1.16 (d, *J* = 6.1 Hz, 3H, H-1 or H-5, **SI-9**) ppm. **^{13}C NMR** (101 MHz, $CDCl_3$, 25°C) δ 138.4 ($C_{q,Ar}$ **SI-10**), 138.0 ($C_{q,Ar}$ **SI-9**), 128.5 (CH_{Ar}), 128.4 (CH_{Ar}), 127.8 (CH_{Ar}), 127.75 (CH_{Ar}), 127.73 (CH_{Ar}), 127.67 (CH_{Ar}), 76.0 (C-2 or C-4, **SI-9**), 72.7 (C-2 or C-4, **SI-10**), 70.6 (C-6 **SI-10**), 70.3 (C-6 **SI-9**), 67.8 (C-2 or C-4, **SI-9**), 64.6 (C-2 or C-4, **SI-10**), 45.7 (C-3 **SI-9**), 44.4 (C-3 **SI-10**), 23.5 (2C, C-1, **SI-9** + **SI-10**), 19.6 (C-5, **SI-9**), 19.1 (C-5, **SI-10**) ppm. The spectral data matched with the literature.[6]

6.4.3 Synthesis of (±)-4-benzyloxypentan-2-one (±)-SI-11

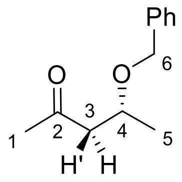

(±)-SI-11

To a solution of a mixture of alcohols **(±)-*syn*-SI-9** and **(±)-*anti*-SI-10** (290 mg, 1.49 mmol, 1 equiv) in CH_2Cl_2 was added Dess-Martin periodinane (760 mg, 1.79 mmol, 1.2 equiv). The reaction mixture was stirred at room temperature for 1.5 h then quenched with sat. aq. Na_2SO_3 (1.5 mL), followed by sat. aq. $NaHCO_3$ (6.5 mL). Et_2O (9 mL) was added and the phases were separated. The aqueous phase was extracted with Et_2O (2 × 16 mL) then the combined organic phases were dried ($MgSO_4$) and concentrated. Column chromatography on silica gel eluting with petroleum ether 40–60 °C/Et_2O (80:20 to 70:30) gave 240 mg (1.25 mmol, 84%) of the desired product **(±)-SI-11** as a colorless oil.

R_f 0.41 (petroleum ether 40–60 °C/Et_2O 70:30). **^{1}H NMR** (400 MHz, $CDCl_3$, 25 °C) δ 7.37 – 7.25 (m, 5H, H_{Ar}), 4.58 (d, $^2J_{H6\text{-}H6'}$ = 11.5 Hz, 1H, H-6), 4.46 (d, $^2J_{H6\text{-}H6'}$ = 11.5 Hz, 1H, H-6'), 4.05 (dqd, $^3J_{H4\text{-}H3}$ = 7.3, $^3J_{H4\text{-}H5}$ = 6.1, $^3J_{H4\text{-}H3'}$ = 5.4 Hz, 1H, H-4), 2.81 (dd, $^2J_{H3\text{-}H3'}$ = 15.9, $^3J_{H3\text{-}H4}$ = 7.3 Hz, 1H, H-3), 2.49 (dd, $^2J_{H3'\text{-}H3}$ = 15.9, $^3J_{H3'\text{-}H4}$ = 5.4 Hz, 1H, H-3'), 2.17 (s, 3H, H-1), 1.25 (d, $^3J_{H5\text{-}H4}$ = 6.1 Hz, 3H, H-5) ppm. **^{13}C NMR** (101 MHz, $CDCl_3$, 25 °C) δ 207.4 (C-2), 138.5 ($C_{q,Ar}$), 128.3 (2 × CH_{Ar}), 127.7 (2 × CH_{Ar}), 127.6 (CH_{Ar}), 71.6 (C-4), 70.8 (C-6), 50.8 (C-3), 31.0 (C-1), 19.8 (C-5) ppm. The spectral data matched with the literature.[7]

6.4.4 Synthesis of (±)-2-benzyloxy-4,4-difluoropentane (±)-SI-12

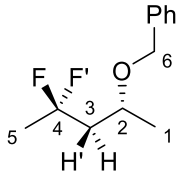

(±)-SI-12

To a solution of **(±)-SI-11** (230 mg, 1.20 mmol, 1 equiv) in CH_2Cl_2 (1.5 mL) was added diethylaminosulfur trifluoride (0.47 mL, 3.59 mmol, 3 equiv) at 0 °C and the resulting mixture was stirred at room temperature for 23 h then at 40 °C for 8 h after which some starting material could still be observed by TLC. HF-pyridine complex (3 drops) was added and the reaction mixture was stirred at 40 °C for 14 h then diluted with CH_2Cl_2 (25 mL) and quenched with sat. aq. $NaHCO_3$ (12 mL). The phases were separated and the aqueous phase was extracted with CH_2Cl_2 (2 × 25 mL). The combined organic phases were dried ($MgSO_4$) and concentrated. Column chromatography on silica gel eluting with petroleum ether 40–60 °C/Et_2O (98:2 to 70:30) gave 127 mg (0.59 mmol, 49%) of desired product **(±)-SI-12** as a pale yellow oil followed by 85 mg (0.44 mmol, 37%) of starting material.

R_f 0.89 (petroleum ether 40–60 °C/Et_2O 70:30). **IR** (neat) 2973 (w), 2935 (w), 2871 (w), 1392(m), 1376 (m), 1235 (m), 1148 (s), 1129 (s), 917 (s). **^{1}H NMR** (400 MHz, $CDCl_3$, 25 °C) δ 7.39 – 7.28 (m, 5H, H_{Ar}), 4.59 (d, $^2J_{H6\text{-}H6'}$ = 11.5 Hz, 1H, H-6), 4.47 (d, $^2J_{H6\text{-}H6'}$ = 11.5 Hz, 1H, H-6'), 3.90 – 3.80 (m, 1H, H-2), 2.22 (dddd, $^3J_{H3\text{-}F}$ = 19.8, $^2J_{H3\text{-}H3'}$ = 14.7, $^3J_{H3\text{-}F}$ = 14.7, $^3J_{H3\text{-}H2}$ = 7.1 Hz, 1H, H-3'), 2.09 – 1.94 (m, 1H, H-3'), 1.65 (t, $^3J_{H5\text{-}F}$ = 19.0 Hz, 3H, H-5), 1.30 (d, $^3J_{H1\text{-}H2}$ = 6.1 Hz, 3H, H-1) ppm. **^{13}C NMR** (101 MHz, $CDCl_3$, 25 °C) δ 138.4 ($C_{q,Ar}$), 128.4 (2 × CH_{Ar}), 127.7 (2 × CH_{Ar}), 127.6 (CH_{Ar}), 123.5 (t, $^1J_{C4\text{-}F}$ = 237.7 Hz, C-4), 70.53 (C-6), 70.5 (dd, $^2J_{C2\text{-}F}$ = 7.3, 3.7 Hz, C-2), 44.9 (t, $^2J_{C3\text{-}F}$ = 24.9 Hz, C-3), 24.1 (t, $^2J_{C5\text{-}F}$ = 27.5 Hz, C-5), 20.4 (d, $^4J_{C1\text{-}F}$ = 1.5 Hz, C-1) ppm. **^{19}F NMR** (376 MHz, $CDCl_3$, 25 °C) δ –85.0 (dqdd, $^2J_{F\text{-}F'}$ = 242.3, $^3J_{F\text{-}H5}$ = 19.0, $^3J_{F\text{-}H3/H3'}$ = 14.7, $^3J_{F\text{-}H3/H3'}$ = 13.0 Hz, 1F, F), –90.2 (m, $^2J_{F'\text{-}F}$ = 242.3, Hz, 1F, F') ppm.

6.4.5 Synthesis of (±)-4,4-difluoropentan-2-ol (±)-E

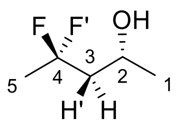

(±)-E

To a solution of benzyl ether **(±)-SI-12** (790 mg, 3.69 mmol, 1 equiv) in CH_2Cl_2/H_2O (9:1, 50 mL) was added DDQ (1.67 g, 7.37 mmol, 2 equiv). The resulting mixture was refluxed for 14 h then diluted with CH_2Cl_2 (110 mL), washed successively with sat. aq. $NaHCO_3$ (80 mL) and brine (40 mL), dried ($MgSO_4$), filtered and evaporated carefully. The product was purified by column chromatography on silica gel eluting with pentane/Et_2O (80:20 to 70:30) then distilled (kugelrohr) to give 79 mg (0.64 mmol, 17%) of pure **(±)-E** as a colorless oil.

R_f 0.37 (pentane/Et_2O 70:30).

Bp 150–160 °C

IR (neat) 3376 (br, w), 2974 (w), 2932 (w), 1392 (m), 1233 (m), 1142 (s), 919 (s).

^{1}H NMR (500 MHz, $CDCl_3$, 25 °C) δ 4.22 (dqdd, $^{3}J_{\mathrm{H2-H3}}$ = 8.6, $^{3}J_{\mathrm{H2-H1}}$ = 6.3, $^{3}J_{\mathrm{H2-OH}}$ = 3.5, $^{3}J_{\mathrm{H2-H3'}}$ = 3.0 Hz, 1H, H-2), 2.07 (dddd, $^{3}J_{\mathrm{H3-F}}$ = 19.3, $^{2}J_{\mathrm{H3-H3'}}$ = 15.0, $^{3}J_{\mathrm{H3-F'}}$ = 13.3, $^{3}J_{\mathrm{H3-H2}}$ = 8.6 Hz, 1H, H-3), 1.99 (dddd, $^{3}J_{\mathrm{H3'-F'}}$ = 20.0, $^{2}J_{\mathrm{H3'-H3}}$ = 15.0, $^{3}J_{\mathrm{H3'-F}}$ = 13.7, $^{3}J_{\mathrm{H3'-H2}}$ = 3.0 Hz, 1H, H-3'), 1.84 (ddd, appears as td, $^{3}J_{\mathrm{OH-H2}}$ = 3.5, $^{1h}J_{\mathrm{OH\cdots F'}}$ = 3.5, $^{1h}J_{\mathrm{OH\cdots F}}$ = 1.4 Hz, 1H, OH), 1.68 (t, $^{3}J_{\mathrm{H5-F/F'}}$ = 18.9 Hz, 3H, H-5), 1.27 (d, $^{3}J_{\mathrm{H1-H2}}$ = 6.3 Hz, 3H, H-1) ppm.

^{1}H{^{19}F} NMR (500 MHz, $CDCl_3$, 25 °C) δ 4.22 (dqdd, $^{3}J_{\mathrm{H2-H3}}$ = 8.6, $^{3}J_{\mathrm{H2-H1}}$ = 6.3, $^{3}J_{\mathrm{H2-OH}}$ = 3.5, $^{3}J_{\mathrm{H2-H3'}}$ = 3.0 Hz, 1H, H-2), 2.07 (dddd, $^{2}J_{\mathrm{H3-H3'}}$ = 15.0, $^{3}J_{\mathrm{H3-H2}}$ = 8.6 Hz, 1H, H-3), 1.99 (dddd, $^{2}J_{\mathrm{H3'-H3}}$ = 15.0, $^{3}J_{\mathrm{H3'-H2}}$ = 3.0 Hz, 1H, H-3'), 1.84 (d, $^{3}J_{\mathrm{OH-H2}}$ = 3.5 Hz, 1H, OH), 1.68 (s, 3H, H-5), 1.27 (d, $^{3}J_{\mathrm{H1-H2}}$ = 6.3 Hz, 3H, H-1) ppm.

^{13}C NMR (101 MHz, $CDCl_3$, 25 °C) δ 124.2 (t, $^{1}J_{\mathrm{C4-F/F'}}$ = 237.7 Hz, C-4), 63.3 (t, $^{3}J_{\mathrm{C2-F/F'}}$ = 4.4 Hz, C-2), 46.4 (t, $^{2}J_{\mathrm{C3-F/F'}}$ = 23.5 Hz, C-3), 24.1 (t, $^{2}J_{\mathrm{C1-F/F'}}$ = 27.5 Hz, C-5), 23.9 (C-1) ppm.

^{19}F NMR (471 MHz, $CDCl_3$, 25 °C) δ –88.2 (ddqdd, appears as dquind, $^{2}J_{\mathrm{F-F'}}$ = 242.5, $^{3}J_{\mathrm{F-H3}}$ = 19.3, $^{3}J_{\mathrm{F-H5}}$ = 18.9, $^{3}J_{\mathrm{F-H3'}}$ = 13.8 Hz, 1F, F ($^{1h}J_{\mathrm{F\cdots HO}}$ = 1.5 Hz not resolved)), –89.7 (ddqdd, $^{2}J_{\mathrm{F'-F}}$ = 242.5, $^{3}J_{\mathrm{F'-H3'}}$ = 20.0, $^{3}J_{\mathrm{F'-H5}}$ = 18.9, $^{3}J_{\mathrm{F'-H3}}$ = 13.2, $^{1h}J_{\mathrm{F'\cdots HO}}$ = 3.5 Hz, 1F, F') ppm.

^{19}F{^{1}H} NMR (471 MHz, $CDCl_3$, 25 °C) δ –88.2 (d, $^{2}J_{\mathrm{F-F'}}$ = 242.5 Hz, 1F, F), –89.7 (d, $^{2}J_{\mathrm{F-F'}}$ = 242.5 Hz, 1F, F') ppm.

^{1}H NMR (500 MHz, $CDCl_3$, –50 °C) δ 4.26 (dqdd, $^{3}J_{\mathrm{H2-H3}}$ = 9.2, $^{3}J_{\mathrm{H2-H1}}$ = 6.3, $^{3}J_{\mathrm{H2-OH}}$ = 2.8, $^{3}J_{\mathrm{H2-H3'}}$ = 2.4 Hz, 1H, H-2), 2.16 (m, upon homodecoupling of H-2 simplifies as dd, $^{1h}J_{\mathrm{OH\cdots F'}}$ = 4.7, $^{1h}J_{\mathrm{OH\cdots F}}$ = 1.7 Hz, upon ^{19}F decoupling simplifies as d, $^{3}J_{\mathrm{OH-H2}}$ = 2.8 Hz, 1H, OH), 2.07 (dddd, $^{3}J_{\mathrm{H3-F}}$ = 21.1, $^{2}J_{\mathrm{H3-H3'}}$ = 15.0, $^{3}J_{\mathrm{H3-F'}}$ = 11.6, $^{3}J_{\mathrm{H3-H2}}$ = 9.2 Hz, 1H, H-3), 1.99 (dddd, $^{3}J_{\mathrm{H3'-F'}}$ = 21.7, $^{2}J_{\mathrm{H3'-H3}}$ = 15.0, $^{3}J_{\mathrm{H3'-F}}$ = 13.7, $^{3}J_{\mathrm{H3'-H2}}$ = 2.4 Hz, 1H, H-3'), 1.68 (t, $^{3}J_{\mathrm{H5-F/F'}}$ = 19.2 Hz, 3H, H-5), 1.25 (d, $^{3}J_{\mathrm{H1-H2}}$ = 6.3 Hz, 3H, H-1) ppm.

^{1}H{^{19}F} NMR (500 MHz, $CDCl_3$, –50 °C) δ 4.26 (dqdd, $^{3}J_{\mathrm{H2-H3}}$ = 9.2, $^{3}J_{\mathrm{H2-H1}}$ = 6.4, $^{3}J_{\mathrm{H2-OH}}$ = 2.8, $^{3}J_{\mathrm{H2-H3'}}$ = 2.4 Hz, 1H, H-2), 2.15 (d, $^{3}J_{\mathrm{OH-H2}}$ = 2.8 Hz, 1H, OH), 2.07 (dd, $^{2}J_{\mathrm{H3-H3'}}$ = 15.1, $^{3}J_{\mathrm{H3-H2}}$ = 9.2 Hz, 1H, H-3), 1.99 (dd, $^{2}J_{\mathrm{H3'-H3}}$ = 15.1, $^{3}J_{\mathrm{H3'-H2}}$ = 2.4 Hz, 1H, H-3'), 1.68 (s, 3H, H-5), 1.25 (d, $^{3}J_{\mathrm{H1-H2}}$ = 6.4 Hz, 3H, H-1) ppm.

^{19}F NMR (471 MHz, $CDCl_3$, –50 °C) δ –89.4 (m, 1F, F), –90.3 (ddqdd, $^{2}J_{\mathrm{F'-F}}$ = 239.6, $^{3}J_{\mathrm{F'-H3'}}$ = 21.7, $^{3}J_{\mathrm{F'-H5}}$ = 19.2, $^{3}J_{\mathrm{F'-H3}}$ = 11.6, $^{1h}J_{\mathrm{F'\cdots HO}}$ = 4.7 Hz, 1F, F') ppm.

^{19}F{^{1}H} NMR (471 MHz, $CDCl_3$, –50 °C) δ –89.5 (d, $^{2}J_{\mathrm{F-F'}}$ = 239.6 Hz, 1F, F), –90.3 (d, $^{2}J_{\mathrm{F-F'}}$ = 239.6 Hz, 1F, F') ppm.

6.5 Synthesis of (±)-4,4-difluorobutan-2-ol (±)-F

6.5.1 Synthesis of (±)-(2S,4R)-2,4-dimethyl-1,3-dioxane (±)-SI-13

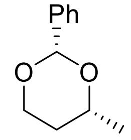

(±)-SI-13

A mixture of 1,3-butanediol (10 g, 111 mmol), benzaldehyde (13.5 mL, 133 mmol, 1.2 equiv), *p*-toluenesulfonic acid monohydrate (2.11 g, 11 mmol, 0.1 equiv) and $MgSO_4$ (26.7 g, 222 mmol, 2 equiv) in 100 mL of CH_2Cl_2 was stirred for 5 hours at room temperature after which the reaction mixture was filtered. The organic layer was washed with a sat. aq. $NaHCO_3$ followed by sat. aq. $Na_2S_2O_3$. The organic layer was dried over $MgSO_4$, filtered and evaporated to give 20.5 g (colorless oil) of a mixture consisting of the desired benzylidene actetal **(±)-SI-13** and benzaldehyde (^{1}H NMR ratio: 92/8) which was used without further purification. The NMR data matched with the literature.[8]

6.5.2 Synthesis of (±)-3-benzyloxy-butan-1-ol (±)-SI-14

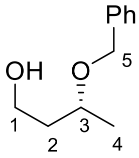

(±)-SI-14

To the mixture of benzylidene acetal **(±)-SI-13** obtained above (8.47 g, 47.5 mmol) in 42 mL of freshly distilled toluene at 0 °C was added DIBAL (1.0 M in hexane, 57 mL, 57 mmol,1.2 equiv) using a dropping funnel over a period of 30 minutes. After stirring for 5 hours at 0 °C, the reaction mixture was stirred overnight at room temperature then quenched by adding MeOH and NaOH (10% aq., 200 mL). The resulting solution was extracted three times with Et_2O. The combined organic layers were dried over $MgSO_4$, filtered and evaporated. The crude mixture was purified by flash chromatography (petroleum ether 40-60 °C/EtOAc, 90:10 to 70:30) afforded 7.03 g (39.0 mmol, 85% over two steps) **(±)-SI-14** as a colorless oil.

^{1}H NMR (400 MHz, $CDCl_3$, 25 °C) δ 7.40–7.27 (m, 5H, H_{Ar}), 4.65 (d, $^2J_{H5\text{-}H5'}$ = 11.6 Hz, 1H, H-5), 4.45 (d, $^2J_{H5'\text{-}H5}$ = 11.6 Hz, 1H, H-5'), 3.79 (m, 3H, H-1 + H-3), 2.47 (dd, $^3J_{OH\text{-}H1}$ = 6.4 Hz, $^3J_{OH\text{-}H1'}$ = 4.5 Hz, 1H, OH), 1.79 (m, 2H, H-2), 1.27 (d, $^3J_{H4\text{-}H3}$ = 6.2 Hz, 3H, H-4) ppm. **^{13}C NMR** (101 MHz, $CDCl_3$, 25 °C) δ 138.4 ($C_{q,Ar}$), 128.5 (2 × CH_{Ar}), 127.7 (2 × CH_{Ar}), 74.7 (C-3), 70.5 (CH_2, C-5), 60.9 (C-1), 38.8 (C-2), 19.4 (C-4) ppm. The NMR data matched with the literature.[9]

6.5.3 Synthesis of (±)-3-benzyloxy-butanal (±)-SI-15

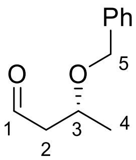

(±)-SI-15

To a solution of the alcohol **(±)-SI-14** (6.86 g, 38.2 mmol, 1 equiv) in CH_2Cl_2 (40 mL) were added TEMPO (604 mg, 3.87mmol, 0.1 equiv) and (diacetoxyiodo)benzene (13.7 g, 42.5 mmol, 1.1 equiv). The reaction mixture was stirred at room temperature for 1.5 h and was then quenched with sat. aq. $Na_2S_2O_3$. The layers were separated and the aqueous layer was extracted with CH_2Cl_2. The combined organic phases were dried over $MgSO_4$, filtered and evaporated. The crude mixture was purified by flash chromatography (petroleum ether 40-60 °C/Et_2O, 90:10 to 85:15) which afforded 6.40 g (35.9 mmol, 94%) of **(±)-SI-15** as a colorless oil.

IR (neat) 2972 (m), 2929 (m), 2866 (m), 2835 (m), 2722 (m), 1723 (s). **^{1}H NMR** (400 MHz, $CDCl_3$, 25 °C) δ 9.81 (app. t, $^3J_{H1\text{-}H2}$ = 2.0 Hz, 1H, H-1), 7.39–7.28 (m, 5H, H_{Ar}), 4.62 (d, $^2J_{H5\text{-}H5'}$ = 11.6 Hz, 1H, H-5), 4.49 (d, $^2J_{H5'\text{-}H5}$ = 11.6 Hz, 1H, H-5'), 4.10 (dqd, $^3J_{H3\text{-}H2}$ = 7.4 Hz, $^3J_{H3\text{-}H4}$ = 6.2 Hz, $^3J_{H3\text{-}H2'}$ = 5.0 Hz, 1H, H-3), 2.72 (ddd, $^2J_{H2\text{-}H2'}$ = 16.4 Hz, $^3J_{H2\text{-}H3}$ = 7.4 Hz, $^3J_{H2\text{-}H1}$ = 2.5 Hz, 1H, H-2), 2.54 (ddd, $^2J_{H2'\text{-}H2}$ = 16.4 Hz, $^3J_{H2'\text{-}H3}$ = 5.0 Hz, $^3J_{H2'\text{-}H1}$ = 1.8 Hz, 1H, H-2'), 1.31 (d, $^3J_{H4\text{-}H3}$ = 6.2 Hz, 3H, H-4) ppm. **^{13}C NMR** (101 MHz, $CDCl_3$, 25 °C) δ 201.4 (C-1), 138.2 ($C_{q,Ar}$), 128.4 (2 × CH_{Ar}), 127.7 (CH_{Ar}), 127.6 (2 × CH_{Ar}), 70.6 (C-5), 70.2 (C-3), 50.5 (C-2), 19.8 (C-4) ppm. **HRMS** (MS+) for $C_{11}H_{14}O_2$ calcd 178.0988, found 178.0986. The ^{1}H NMR data matched with the literature.[10]

6.5.4 Synthesis of (±)-2-benzyloxy-4,4-difluorobutane (±)-SI-16

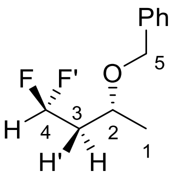

(±)-SI-16

To a solution of the aldehyde **(±)-SI-15** (5.00 g, 28.4 mmol, 1 equiv) dissolved in 100 mL of dry CH_2Cl_2 was added diethylaminosulfur trifluoride (7.50 mL, 56.7 mmol, 2 equiv). The mixture was stirred at room temperature for 45 min and was then poured in a saturated aqueous solution of Na_2CO_3. The layers were separated and the aqueous layer was extracted three times with CH_2Cl_2. The combined organic phases were dried over $MgSO_4$, filtered and evaporated. The crude mixture was purified by flash chromatography (petroleum ether 40-60 °C/Et_2O 99:1) which afforded 5.21 g (26.0 mmol, 93%) of **(±)-SI-16** as a colorless oil.

IR (neat) 3032 (w), 2976 (w), 2935 (w), 2359 (w). **^{1}H NMR** (400 MHz, $CDCl_3$, 25 °C) δ 7.40–7.28 (m, 5H, H_{Ar}), 6.00 (tdd, $^2J_{H4\text{-}F}$ = 57.0 Hz, $^3J_{H4\text{-}H3}$ = 6.6 Hz, $^3J_{H4\text{-}H3'}$ = 3.1 Hz, 1H, H-4), 4.62 (d, $^2J_{H5\text{-}H5'}$ = 11.5 Hz, 1H, H-5), 4.43 (d, $^2J_{H5'\text{-}H5}$ = 11.5 Hz, 1H, H-5'), 3.79 (m, 1H, H-2), 2.21–1.90 (m, 2H, H-3 + H-3'), 1.28 (d, $^3J_{H1\text{-}H2}$ = 6.2 Hz, 3H, H-1) ppm. **^{13}C NMR** (101 MHz, $CDCl_3$, 25 °C) δ: 138.2 ($C_{q,Ar}$), 128.4 (2 × CH_{Ar}), 127.7 (CH_{Ar}), 127.6 (2 × CH_{Ar}), 116.0 (t, $^1J_{C4\text{-}F}$ = 238 Hz, C-4), 70.6 (C-5), 70.1 (dd, $^3J_{C2\text{-}F}$ = 9 Hz, $^3J_{C2\text{-}F'}$ = 4 Hz, C-2), 41.6 (t, $^2J_{C3\text{-}F}$ = 21 Hz,

C-3), 19.7 (C-1) ppm. **^{19}F NMR** (376 MHz, $CDCl_3$, 25 °C) δ –116.7 (dddd appears as ddt, $^2J_{F\text{-}F'}$ = 284.4 Hz, $^2J_{F\text{-}H4}$ = 55.5 Hz, $^4J_{F\text{-}H3}$ = 10.4 Hz, 1F, F), –117.88 (dddd, $^2J_{F'\text{-}F}$ = 284.4 Hz, $^2J_{F'\text{-}H4}$ = 57.2 Hz, $^4J_{F'\text{-}H3}$ = 26.0 Hz, $^4J_{F'\text{-}H3'}$ = 13.9 Hz, 1F, F'). **HRMS** (MS+) for $C_{11}H_{14}F_2O$ calcd 200.0994, found 200.0998.

6.5.5 Synthesis of (±)-4,4-difluorobutan-2-ol (±)-F

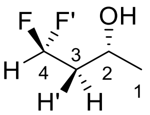

(±)-F

To a mixture of the benzylated compound **(±)-SI-16** (15.0mmol, 3.01g) in 180mL of dichloromethane and 18mL of water was added 2,3-Dichloro-5,6-dicyano-*p*-benzoquinone (30mmol, 6.81g). The mixture was heated at reflux and stirred for 24h and was then poured into a saturated aqueous solution of $NaHCO_3$ (1L). The aqueous layer was extracted with dichloromethane several times until the product is no longer detectable on TLC from the organic layer.

IR : 3356.4 (br), 2976.7 (m), 2933.5 (m), 1401.5 (m), 1378.3 (m), 1104.5 (s).

^{1}H NMR (500 MHz, $CDCl_3$, 25 °C) δ 6.01 (tdd, $^2J_{H4\text{-}F}$ = 56.8 Hz, $^3J_{H4\text{-}H3'}$ = 5.5 Hz, $^3J_{H4\text{-}H3}$ = 3.6 Hz, 1H, H-4), 4.12 (dqdd, $^3J_{H2\text{-}H3}$ = 8.4 Hz, $^3J_{H2\text{-}H1}$ = 6.3 Hz, $^3J_{H2\text{-}OH}$ = 4.5 Hz, $^3J_{H2\text{-}H3'}$ = 4.4 Hz, 1H, H-2), 2.08 – 1.95 (ddddd appears as dtdd, $^3J_{H3\text{-}F'}$ = 20.9 Hz, $^3J_{H3\text{-}H3'}$ = 14.5 Hz, $^3J_{H3\text{-}F}$ = 14.5 Hz, $^3J_{H3\text{-}H2}$ = 8.4 Hz, $^3J_{H3\text{-}H4}$ = 3.6 Hz, 1H, H-3), 1.97 (dddddd appears as dtddd, $^3J_{H3'\text{-}F'}$ = 16.3 Hz, $^3J_{H3'\text{-}F}$ = 14.5 Hz, $^2J_{H3'\text{-}H3}$ = 14.5 Hz, $^3J_{H3'\text{-}H4}$ = 5.5 Hz, $^3J_{H3'\text{-}H2}$ = 4.4 Hz, $^4J_{H3'\text{-}OH}$ = 0.5 Hz, 1H, H-3'), 1.52 (1H, dddd appears as dq, $^3J_{OH\text{-}H2}$ = 4.5 Hz, $^4J_{OH\text{-}H3'}$ = 0.6 Hz, $^{1h}J_{OH\cdots F}$ = 0.6 Hz, $^{1h}J_{OH\cdots F}$ = 0.6 Hz, OH), 1.39 (dt, $^3J_{H1\text{-}H2}$ = 6.2 Hz, $^5J_{H1\text{-}F}$ = 0.6 Hz, 3H, H-1) ppm.

^{1}H{^{19}F} NMR (500 MHz, $CDCl_3$, 25 °C) δ 6.01 (dd, $^3J_{H4\text{-}H3'}$ = 5.5 Hz, $^3J_{H4\text{-}H3}$ = 3.7 Hz, 1H, H-4), 4.12 (dqdd, $^3J_{H2\text{-}H3}$ = 8.4 Hz, $^3J_{H2\text{-}H1}$ = 6.3 Hz, $^3J_{H2\text{-}OH}$ = 4.5 Hz, $^3J_{H2\text{-}H3'}$ = 4.4 Hz, 1H, H-2), 2.01 (ddd, $^3J_{H3\text{-}H3'}$ = 14.5 Hz, $^3J_{H3\text{-}H2}$ = 8.3 Hz, $^3J_{H3\text{-}H4}$ = 3.6 Hz, 1H, H-3), 1.97 (dddd, $^2J_{H3'\text{-}H3}$ = 14.5 Hz, $^3J_{H3'\text{-}H4}$ = 5.6 Hz, $^3J_{H3'\text{-}H2}$ = 4.4 Hz, $^4J_{H3'\text{-}OH}$ = 0.5 Hz, 1H, H-3'), 1.52 (dd, $^3J_{OH\text{-}H2}$ = 4.5 Hz, $^4J_{OH\text{-}H3'}$ = 0.5 Hz, 1H, OH), 1.29 (d, $^3J_{H1\text{-}H2}$ = 6.3 Hz, 3H, H-1) ppm.

^{13}C NMR (101 MHz, $CDCl_3$, 25 °C) δ 116.3 (t, $^1J_{C4\text{-}F}$ = 238 Hz, C-4), 63.2 (dd, $^3J_{C2\text{-}F}$ = 7, 5 Hz, C-2), 42.9 (t, $^2J_{C3\text{-}F}$ = 20 Hz, C-3), 24.0 (C-1) ppm.

^{19}F NMR (471 MHz, $CDCl_3$, 25 °C) δ –116.4 (ddtm, $^2J_{F\text{-}F'}$ = 286.1 Hz, $^2J_{F\text{-}H4}$ = 56.7 Hz, $^3J_{F\text{-}H3'}$ = $^3J_{F\text{-}H3}$ = 14.7 Hz ($^{1h}J_{F\cdots HO}$ and $^5J_{F\text{-}H1}$ not resolved), 1F, F), –117.5 (ddddm, $^2J_{F'\text{-}F}$ = 286.1 Hz, $^2J_{F'\text{-}H4}$ = 57.0 Hz, $^3J_{F'\text{-}H3}$ = 21.1 Hz, $^3J_{F'\text{-}H3'}$ = 16.4 Hz, ($^{1h}J_{F\cdots HO}$ and $^5J_{F\text{-}H1}$ not resolved), 1F, F') ppm.

^{19}F{^{1}H} NMR (471 MHz, $CDCl_3$, 25 °C) δ –116.4 (d, $^2J_{F\text{-}F'}$ = 286.1 Hz, 1F, F), –117.1 (d, $^2J_{F\text{-}F'}$ = 286.1 Hz, 1F, F') ppm.

HRMS (MS+) for $C_4H_8F_2O$ calcd 110.0538, found 110.0509.

6.6 Synthesis of 3,3-difluoropropan-1-ol G

6.6.1 Synthesis of 1-benzyloxy-3,3-difluoropropane SI-17

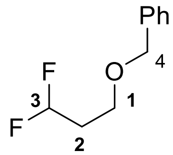

SI-17

To a round-bottom flask were added 3-benzyloxypropanal (1.00 g, 6.09 mmol, 1.0 equiv), CH_2Cl_2 (20 mL) and diethylaminosulfur trifluoride (1.61 mL, 12.2 mmol, 2.0 equiv). The resulting solution was stirred at room temperature overnight. The reaction mixture was quenched with sat. aq. $NaHCO_3$ solution (40 mL) and extracted with CH_2Cl_2 (3 × 60 mL). The combined organic layers were washed with brine (100 mL), dried over $MgSO_4$, filtered and concentrated. The crude was purified by flash chromatography (pentane/Et_2O 97:3 to 90:10) to afford 1.02 g (5.48 mmol, 90%) of **SI-17** as a colorless oil.

IR (neat) 3087 (w), 3059 (w), 3028 (w), 2970 (w), 2939 (w), 2866 (w), 2798 (w), 1492 (w), 1454 (m), 1395 (m), 1364 (m), 1095 (s), 1023 (s), 975 (s), 906 (m), 733 (s) cm^{-1}. **^{1}H NMR** (400 MHz, $CDCl_3$, 25 °C) δ 7.42–7.28 (m,

5H, H_{Ar}), 6.02 (tt, $^{2}J_{H3\text{-}F}$ = 56.9 Hz, $^{3}J_{H3\text{-}H2}$ = 4.8 Hz, 1H, H-3), 4.53 (s, 2H, H-4), 3.64 (t, $^{3}J_{H1\text{-}H2}$ = 6.1 Hz, 2H, H-1), 2.15 (ttd, $^{3}J_{H2\text{-}F}$ = 16.7 Hz, $^{3}J_{H2\text{-}H1}$ = 6.1 Hz, $^{3}J_{H2\text{-}H3}$ = 4.9 Hz, 2H, H-2) ppm. **^{13}C NMR** (101 MHz, $CDCl_3$, 25 °C) δ 137.8 ($C_{q,Ar}$), 128.4 (2 × CH_{Ar}), 127.8 (CH_{Ar}), 127.6 (2 × CH_{Ar}), 115.9 (t, $^{1}J_{C3\text{-}F}$ = 237.7 Hz, C-3), 73.2 (C-4), 64.0 (t, $^{3}J_{C1\text{-}F}$ = 6.9 Hz, C-1), 34.8 (t, $^{2}J_{C2\text{-}F}$ = 21.8 Hz, C-2) ppm. **^{19}F NMR** (376 MHz, $CDCl_3$, 25 °C) δ –117.8 (dt, $^{2}J_{F\text{-}H3}$ = 57.2 Hz, $^{3}J_{F\text{-}H2}$ = 16.5 Hz, 2F) ppm. **MS** (EI) *m/z* 186.2 ($M^{\bullet+}$, 8%). **HRMS** (MS+) for $C_{10}H_{12}F_2O$ calcd 186.0851, found 186.0848.

6.6.2 Synthesis of 3,3-difluoropropan-1-ol G

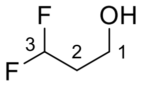

G

To a round-bottom flask were added **SI-17** (360 mg, 1.93 mmol, 1 equiv), CH_2Cl_2 (22.5 mL), water (2.50 mL) and 2,3-dichloro-5,6-dicyano-*p*-benzoquinone (1.74 g, 7.72 mmol, 4 equiv). The resulting mixture was refluxed overnight. After completion was reached, indicated by TLC analysis, the reaction mixture was directly purified by flash chromatography (pentane/Et_2O, 70:30) followed by a second column chromatography (CH_2Cl_2, 100%) to afford 30 mg (0.31 mmol, 16%) of **G** as a colorless oil.

IR (neat) 3350 (br, w), 2958 (w), 2913 (m), 2852 (w), 1430 (w), 1401 (w), 1381 (w), 1119 (s), 1070 (s), 1054 (s), 964 (s), 805 (w) cm^{-1};

^{1}H NMR (500 MHz, $CDCl_3$, 25 °C) δ 6.03 (tt, $^{2}J_{H3\text{-}F}$ = 57.0 Hz, $^{3}J_{H3\text{-}H2}$ = 4.6 Hz, 1H, H-3), 3.87 (td, $^{3}J_{H1\text{-}H2}$ = 6.0 Hz, $^{3}J_{H1\text{-}OH}$ = 5.1 Hz, 2H, H-1), 2.12 (ttd, $^{3}J_{H2\text{-}F}$ = 17.0 Hz, $^{3}J_{H2\text{-}H1}$ = 6.0 Hz, $^{3}J_{H2\text{-}H3}$ = 4.6 Hz, 2H, H-2), 1.45 (1H, tt, $^{3}J_{OH\text{-}H1}$ = 5.1, $^{1h}J_{OH\cdots F}$ = 0.4 Hz, OH) ppm.

^{1}H{^{19}F} NMR (500 MHz, $CDCl_3$, 25 °C) δ 6.03 (t, $^{3}J_{H3\text{-}H2}$ = 4.6 Hz, 1H, H-3), 3.87 (td, $^{3}J_{H1\text{-}H2}$ = 5.9 Hz, $^{3}J_{H1\text{-}OH}$ = 5.2 Hz, 2H, H-1), 2.12 (td, $^{3}J_{H2\text{-}H1}$ = 6.0 Hz, $^{3}J_{H2\text{-}H3}$ = 4.6 Hz, 2H, H-2), 1.45 (t, $^{3}J_{OH\text{-}H1}$ = 5.1 Hz, 1H, OH) ppm.

^{13}C NMR (126 MHz, $CDCl_3$, 25 °C) δ 116.1 (t, $^{1}J_{C3\text{-}F}$ = 239.4 Hz, C-3), 56.8 (t, $^{3}J_{C1\text{-}F}$ = 6.8 Hz, C-1), 36.9 (t, $^{2}J_{C2\text{-}F}$ = 20.3 Hz, C-2) ppm.

^{19}F NMR (376 MHz, $CDCl_3$, 25 °C) δ –117.8 (dt, $^{2}J_{F\text{-}H3}$ = 56.8 Hz, $^{3}J_{F\text{-}H2}$ = 16.9 Hz, 2F) ppm ($^{1h}J_{F\cdots HO}$ not resolved).

^{19}F{^{1}H} NMR (376 MHz, $CDCl_3$, 25 °C) δ –117.6 (s, 2F) ppm.

HRMS (MS+) for $C_3H_6F_2O$ calcd 96.0381, found 96.0381.

1. Caron, G.; Kazlauskas, R. J., *Tetrahedron: Asymmetry* **1994,** *5* (4), 657-664.
2. Ikeda, H.; Sato, E.; Sugai, T.; Ohta, H., *Tetrahedron* **1996,** *52* (24), 8113-8122.
3. Yoneda, A.; Fukuhara, T.; Hara, S., *Chem. Commun. (Cambridge, U. K.)* **2005,** (28), 3589-3590.
4. Ashley, G. W.; Burlingame, M.; Desai, R.; Fu, H.; Leaf, T.; Licari, P. J.; Tran, C.; Abbanat, D.; Bush, K.; Macielag, M., *J. Antibiot.* **2006,** *59* (7), 392-401.
5. Denmark, S. E.; Almstead, N. G., *J. Am. Chem. Soc.* **1991,** *113* (21), 8089-8110.
6. Hoffmann, R. W.; Stenkamp, D.; Trieselmann, T.; Göttlich, R., *Eur. J. Org. Chem.* **1999,** *1999* (11), 2915-2927.
7. Zou, H.; Zhou, L.; Li, Y.; Cui, Y.; Zhong, H.; Pan, Z.; Yang, Z.; Quan, J., *J. Med. Chem.* **2010,** *53* (3), 994-1003.
8. Soderquist, J. A.; Kock, I.; Estrella, M. E., *Org. Process Res. Dev.* **2006,** *10* (5), 1076-1079.
9. Shie, C.-R.; Tzeng, Z.-H.; Kulkarni, S. S.; Uang, B.-J.; Hsu, C.-Y.; Hung, S.-C., *Angew. Chem., Int. Ed.* **2005,** *44* (11), 1665-1668.
10. Bianchi, O.; Cesti, P.; Golini, P., *Tetrahedron* **1989,** *45* (3), 869-876.

SUPPORTING INFORMATION 4
COPIES OF SPECTRA (INTERMEDIATES)
COPIES OF ^{13}C OF NOVEL FLUOROHYDRINS

7 Copies of all spectra 107
7.1 Synthesis of (±)-*syn* and (±)-*anti*-4-fluoropentan-2-ol ((±)-*syn*-A and (±)-*anti*-A) 107
7.1.1 Isolation of *meso*-2,4-pentanediol (*meso*-SI-1) and (±)-2,4-pentanediol ((±)-SI-2) from a commercial mixture of *meso* and *racemic* isomers....................107
7.1.1.1 meso-2,4-Pentanediol (meso-SI-1) 107
7.1.1.2 (±)-2,4-Pentanediol ((±)-SI-2) 109
7.1.2 (±)-*anti* and (±)-*syn-2-*(3'-Methylbenzoyloxy)-4-fluoropentane (±)-*anti*-SI-3 and (±)-*syn*-SI-4 111
7.1.2.1 (±)-anti-2-(3'-Methylbenzoyloxy)-4-fluoropentane (±)-anti-SI-3 111
7.1.2.2 (±)-syn-2-(3'-Methylbenzoyloxy)-4-fluoropentane (±)-syn-SI-4 114
7.1.3 Fluorohydrins (±)-*anti*-A and (±)-*syn*-A 117
7.1.3.1 (±)-anti-4-Fluoropentan-2-ol (±)-anti-A 117
7.1.3.2 (±)-syn-4-Fluoropentan-2-ol (±)-syn-A 118
7.2 Synthesis of 4-fluorobutan-2-ol (±)-B 119
7.2.1 3-Fluoropropanal SI-5 119
7.2.2 4-Fluorobutan-2-ol (±)-B 121
7.3 Synthesis of 3-fluoro-2,2-dimethylpropan1-ol C 122
7.3.1 3-Fluoro-2,2-dimethyl-1-(*meta*-methylbenzoyloxy)-propane SI-6 122
7.3.2 3-Fluoro-2,2-dimethylpropan1-ol C 125
7.4 Synthesis of (±)-4,4-difluoropentan-2-ol (±)-E 126
7.4.1 4,6-Dimethyl-2-phenyl-1,3-dioxane *meso*-SI-7 and (±)-SI-8 126
7.4.2 (2*S**,4*R**)-4-Benzyloxypentan-2-ol (±)-*syn*-SI-9 and (2*R**,4*R**)-4-benzyloxypentan-2-ol (±)-*anti*-SI-10 128
7.4.3 (±)-4-Benzyloxypentan-2-one (±)-SI-11 130

7.4.4 (±)-2-Benzyloxy-4,4-difluoropentane (±)-SI-12 132
7.4.5 (±)-4,4-difluoropentan-2-ol (±)-E 135
7.5 Synthesis of (±)-4,4-difluorobutan-2-ol (±)-F 136
7.5.1 (±)-3-Benzyloxy-butanal (±)-SI-15 136
7.5.2 (±)-2-Benzyloxy-4,4-difluorobutane (±)-SI-16 138
7.5.3 (±)-4,4-Difluorobutan-2-ol (±)-F 142
7.6 Synthesis of 3,3-difluoropropan-1-ol G 143
7.6.1 1-Benzyloxy-3,3-difluoropropane SI-17 143
7.6.2 3,3-Difluoropropan-1-ol G 146

7 Copies of all spectra

7.1 Synthesis of (±)-*syn* and (±)-*anti*-4-fluoropentan-2-ol ((±)-*syn*-A and (±)-*anti*-A)

7.1.1 Isolation of *meso*-2,4-pentanediol (*meso*-SI-1) and (±)-2,4-pentanediol ((±)-SI-2) from a commercial mixture of *meso* and *racemic* isomers

7.1.1.1 meso-2,4-Pentanediol (meso-SI-1)

^{1}H NMR, $CDCl_3$, 400 MHz

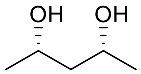

***meso*-SI-1**

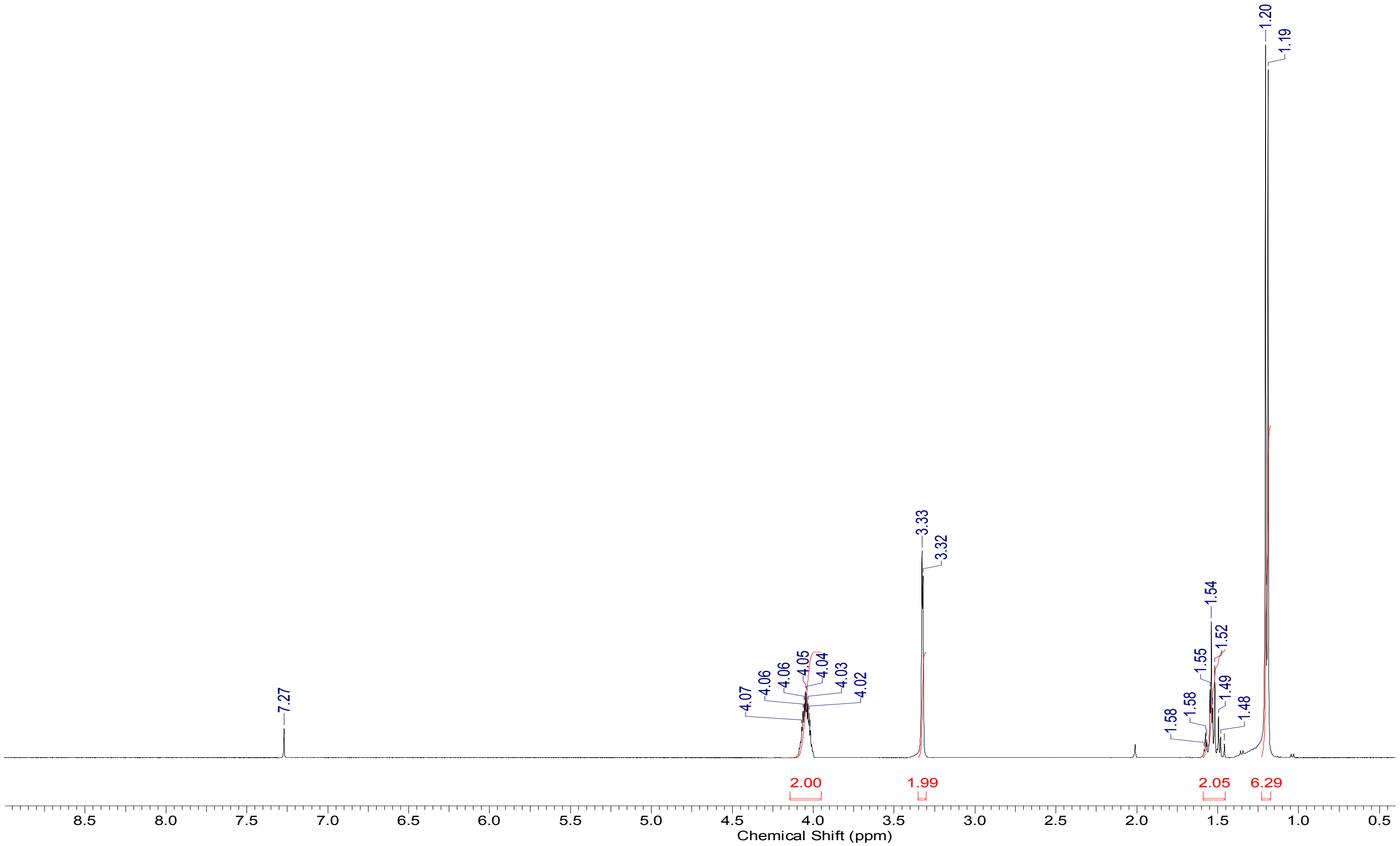

^{13}C NMR, $CDCl_3$, 101 MHz

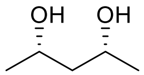

***meso*-SI-1**

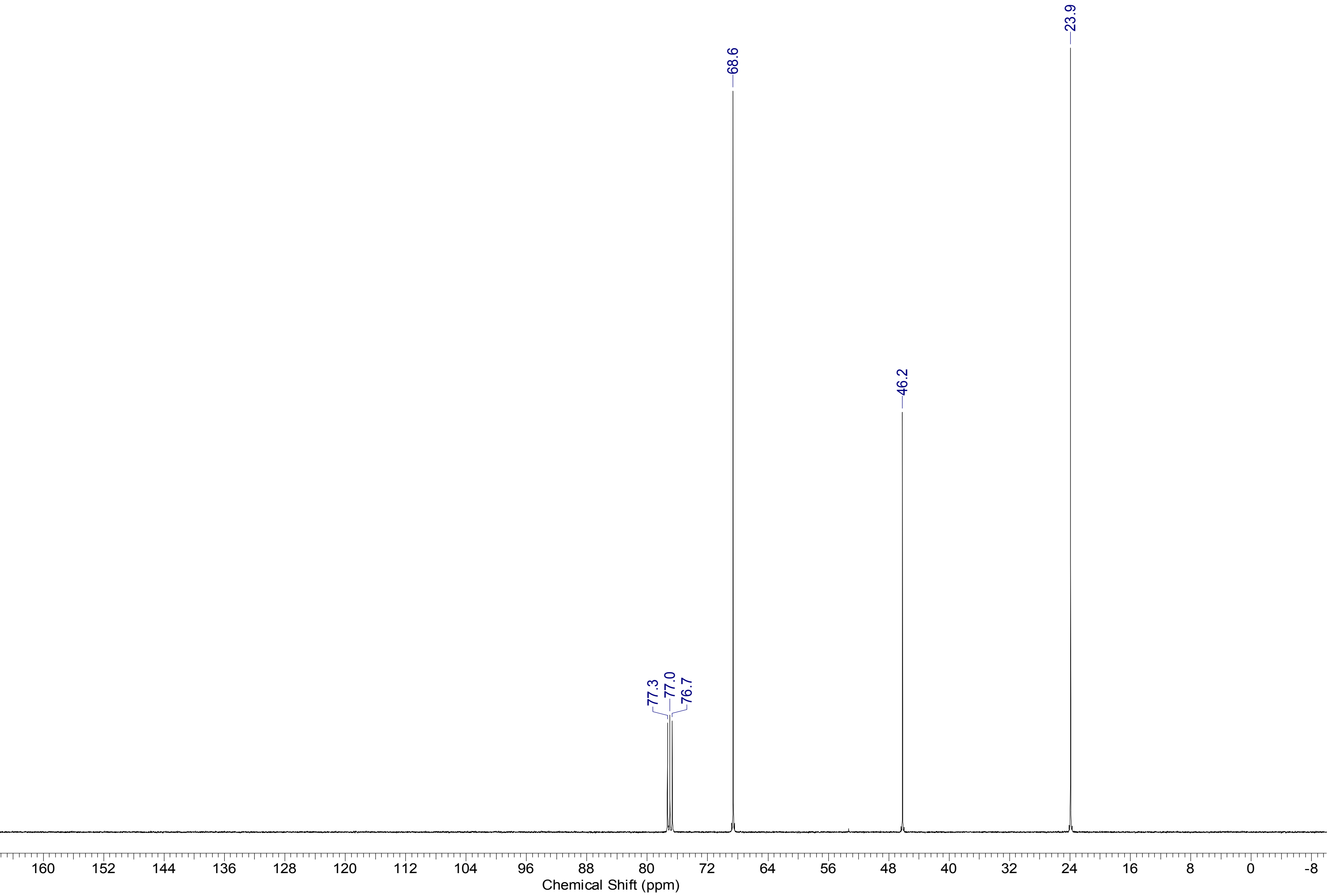

7.1.1.2 (±)-2,4-Pentanediol ((±)-SI-2)

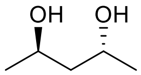

(±)-SI-2

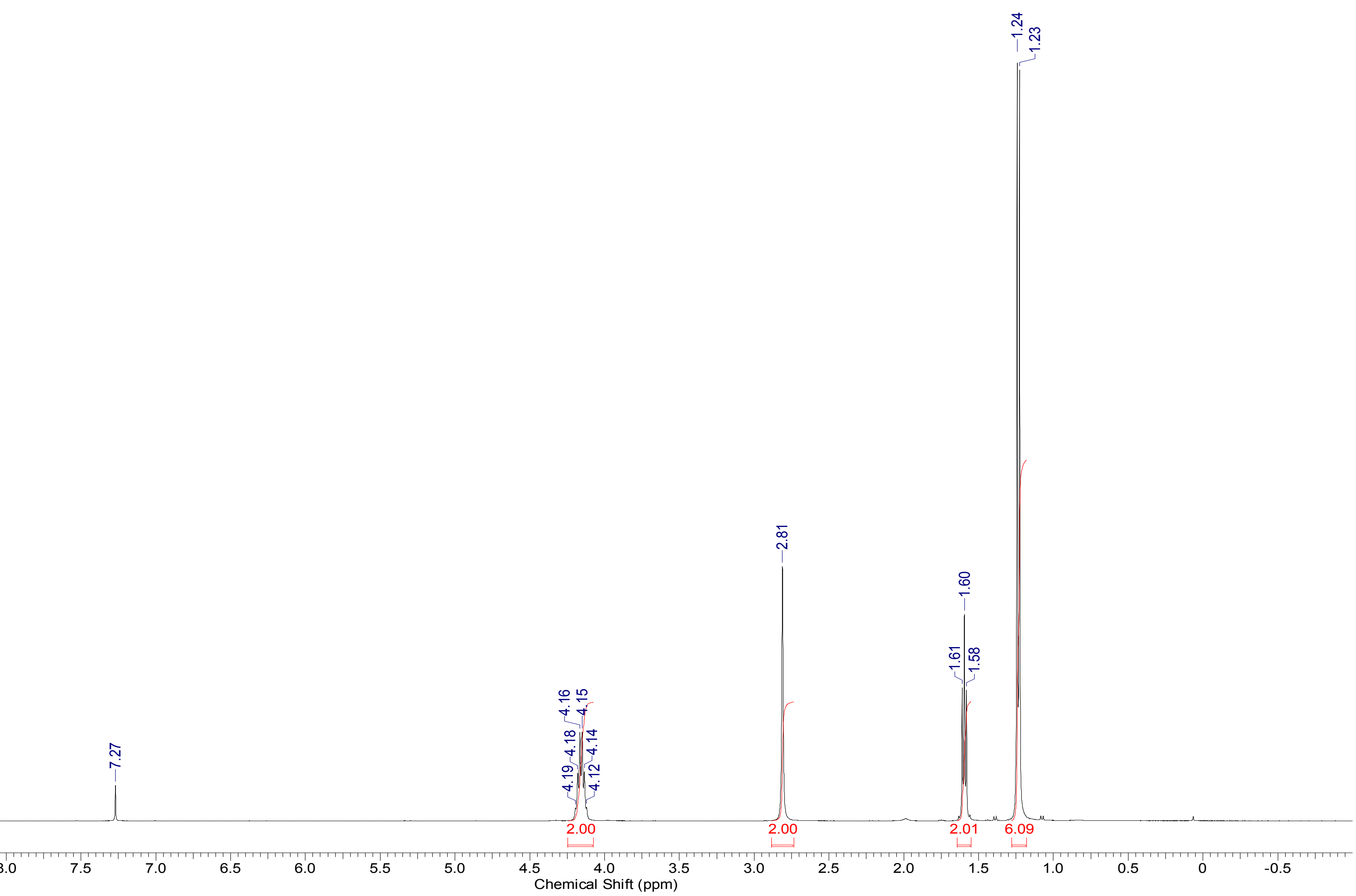

^{13}C NMR, CDCl$_3$, 101 MHz

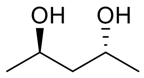

(±)-SI-2

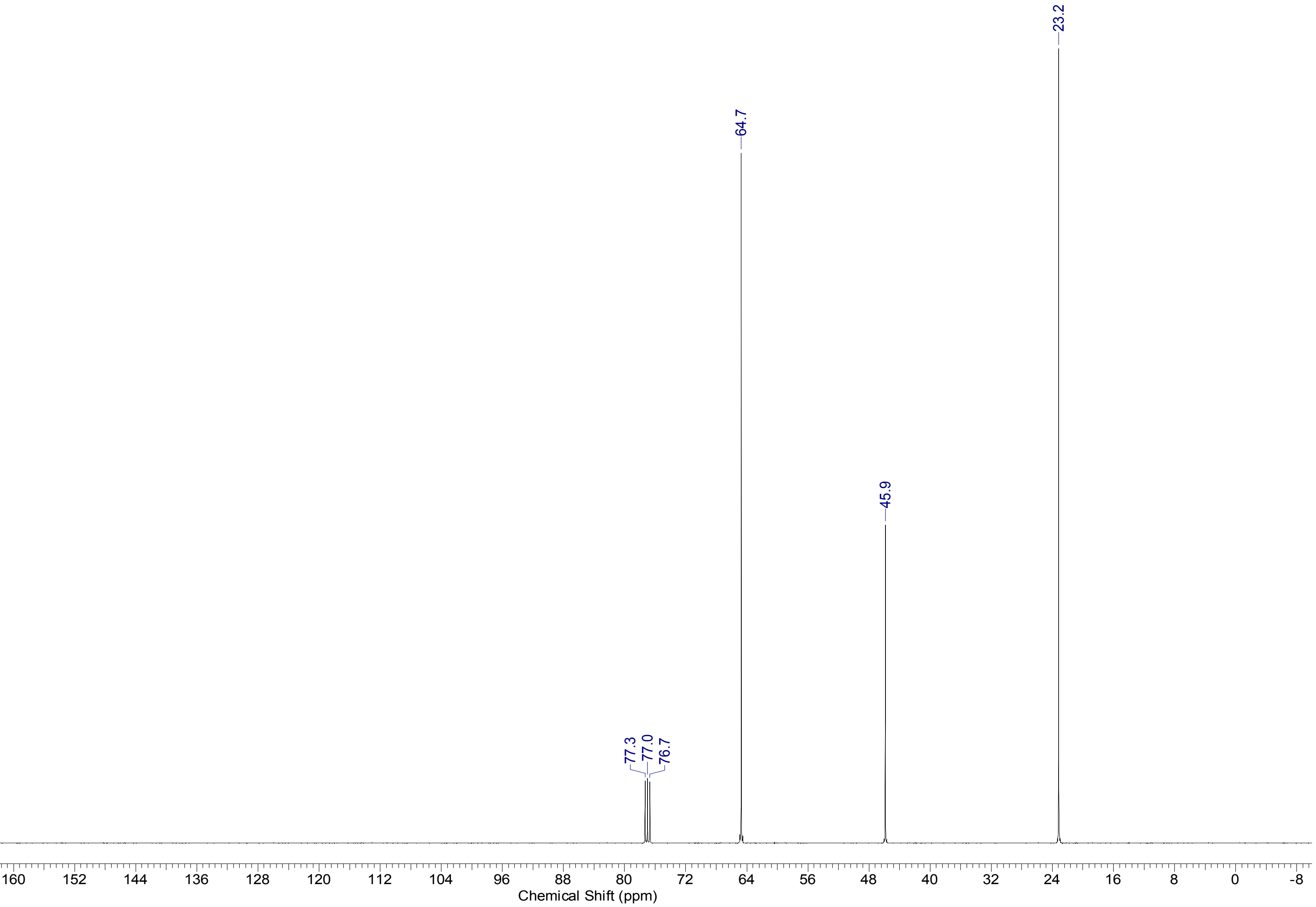

7.1.2 (±)-*anti* and (±)-*syn*-2-(3'-Methylbenzoyloxy)-4-fluoropentane (±)-*anti*-SI-3 and (±)-*syn*-SI-4

7.1.2.1 (±)-anti-2-(3'-Methylbenzoyloxy)-4-fluoropentane (±)-anti-SI-3

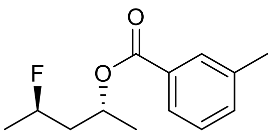

(±)-*anti*-SI-3

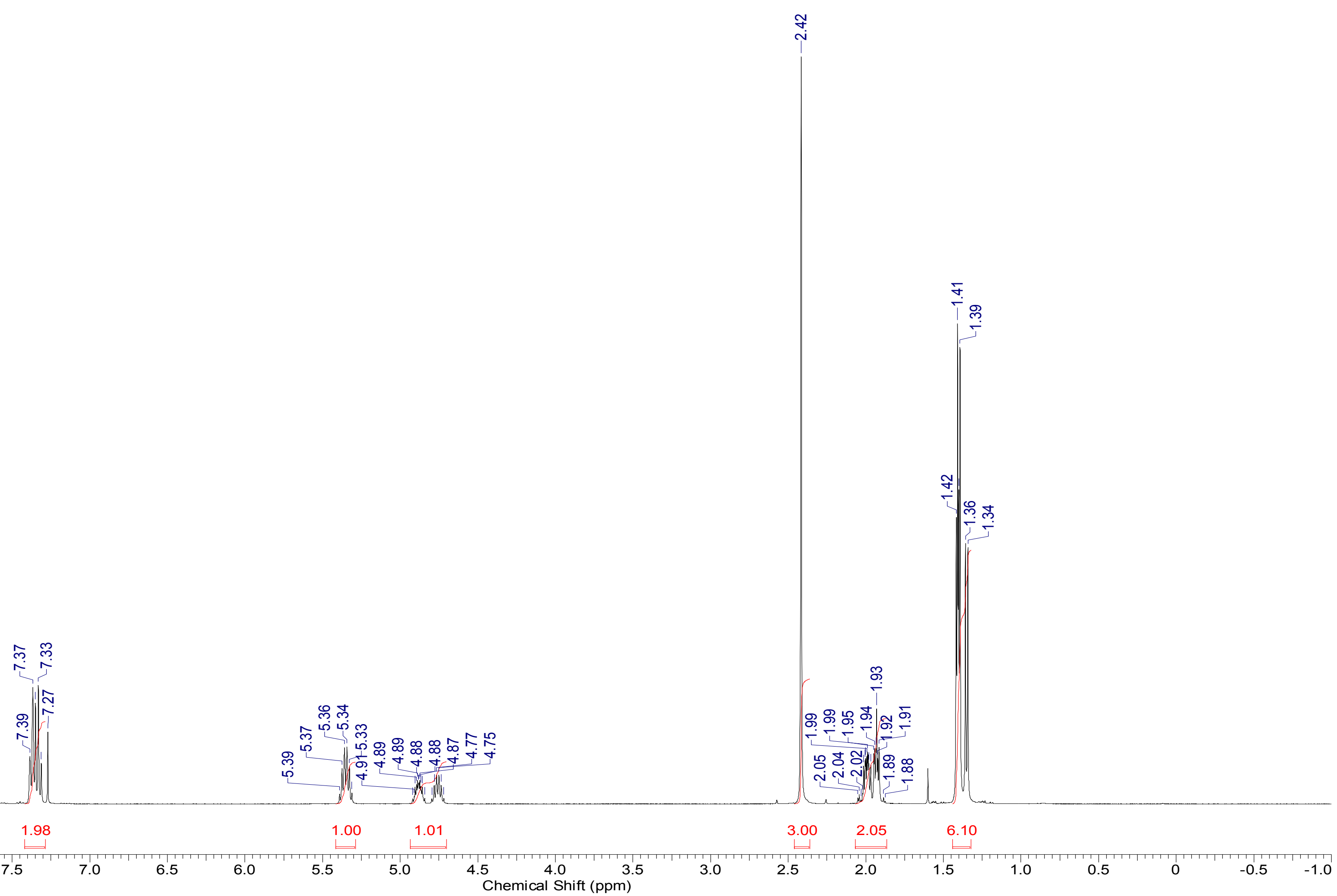

^{13}C NMR, CDCl$_3$, 101 MHz

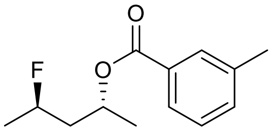

(±)-*anti*-SI-3

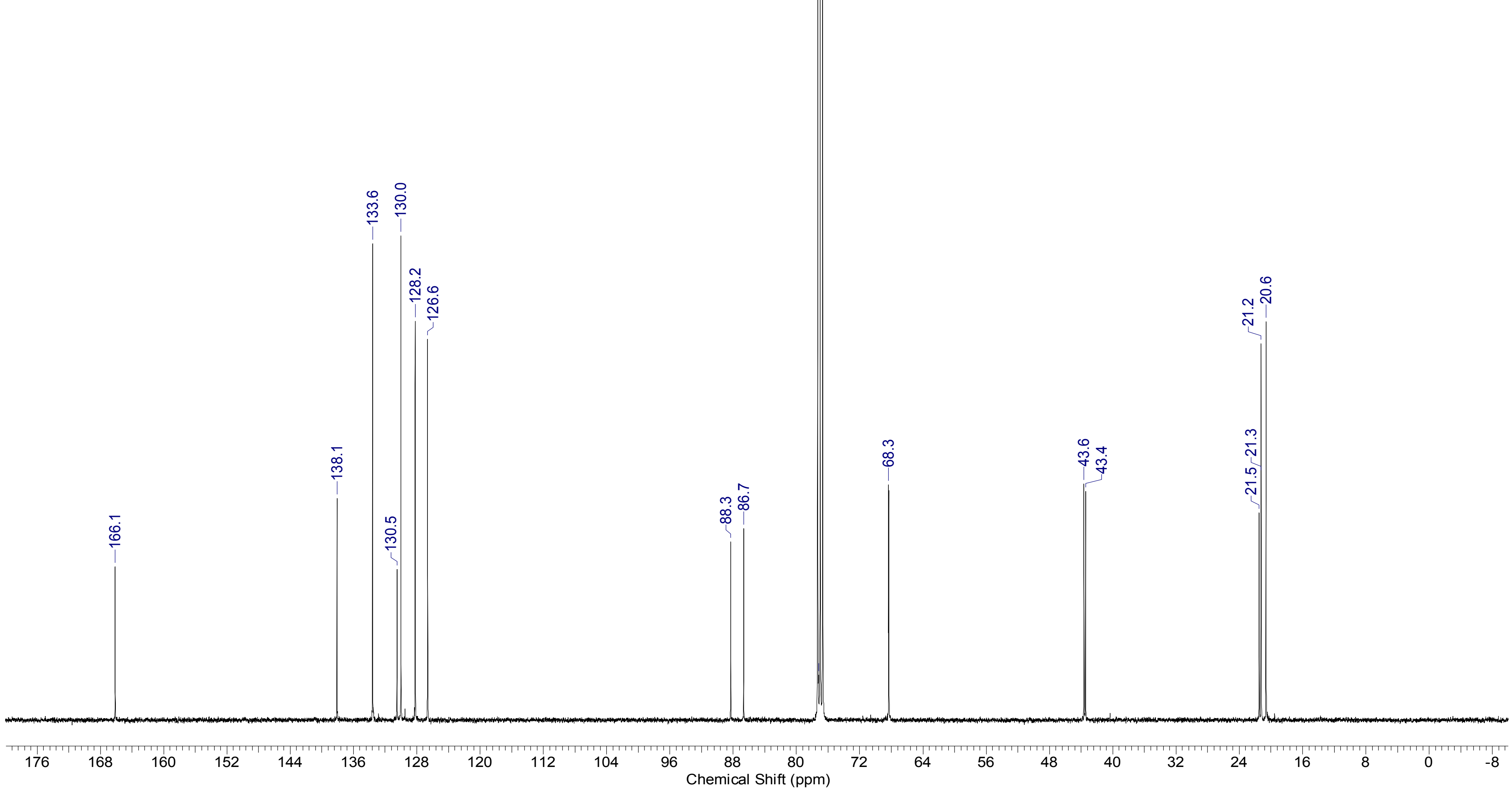

^{19}F NMR, $CDCl_3$, 376 MHz

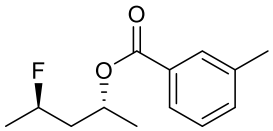

(±)-*anti*-SI-3

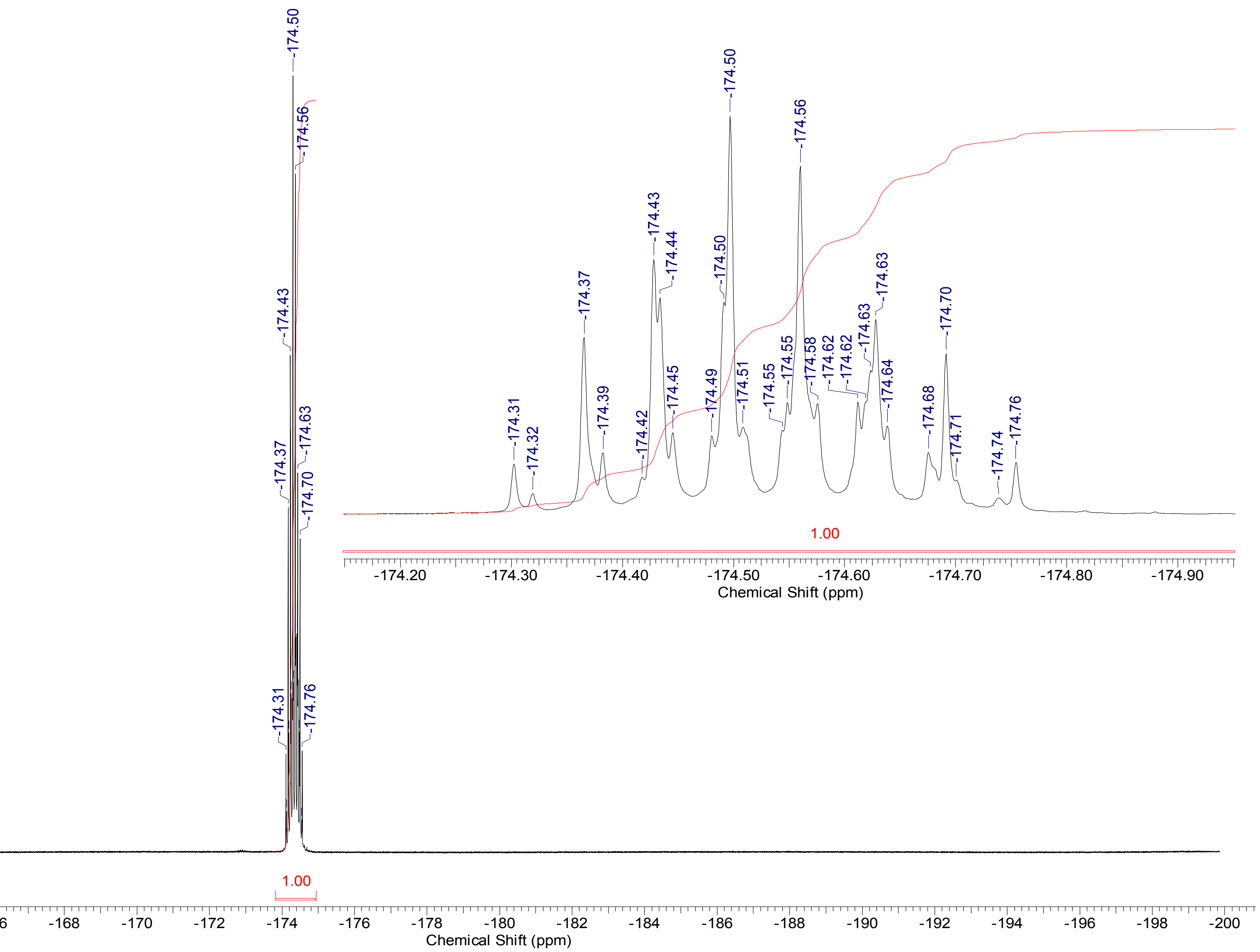

7.1.2.2 (±)-syn-2-(3'-Methylbenzoyloxy)-4-fluoropentane (±)-syn-SI-4

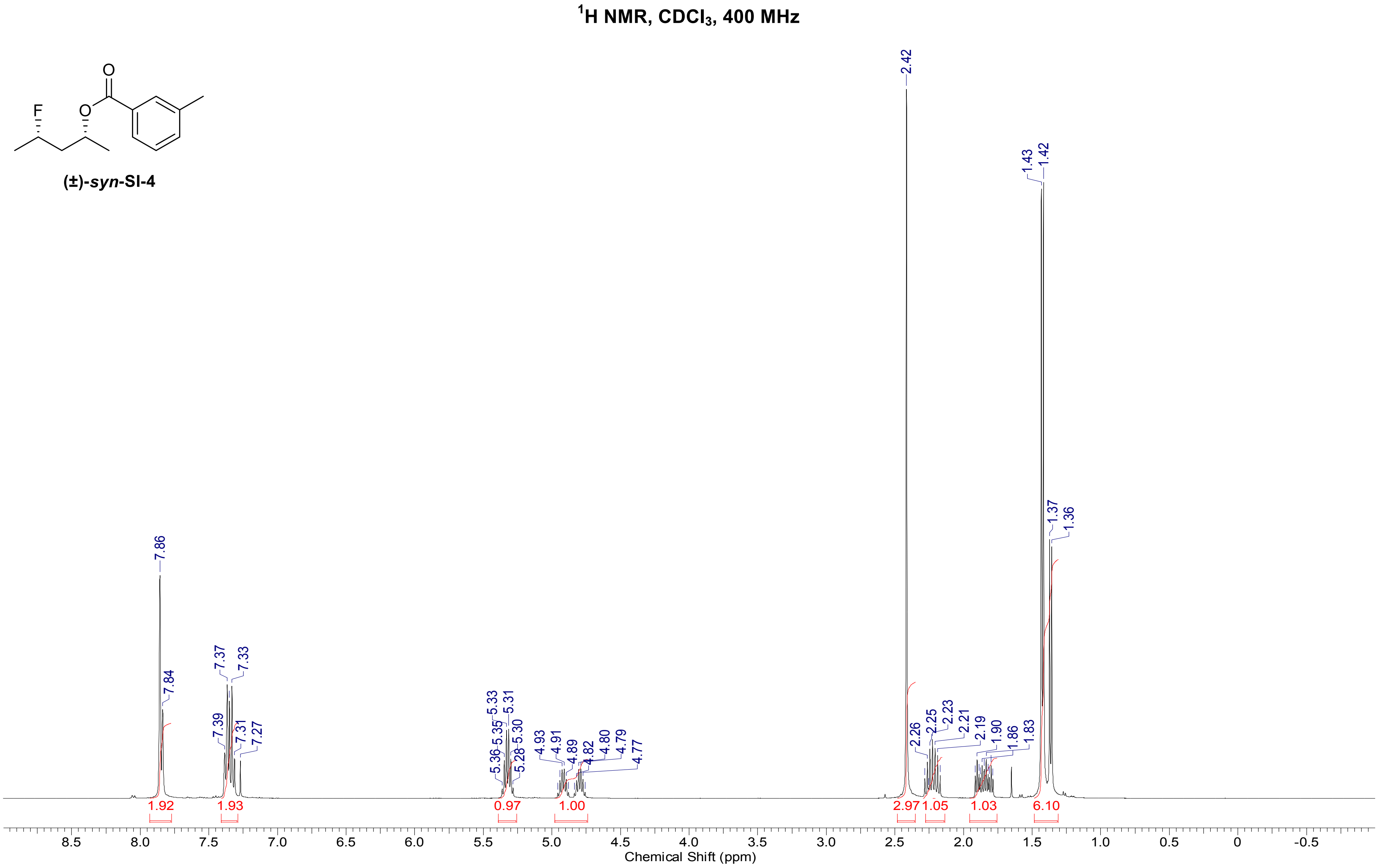

^{13}C NMR, $CDCl_3$, 101 MHz

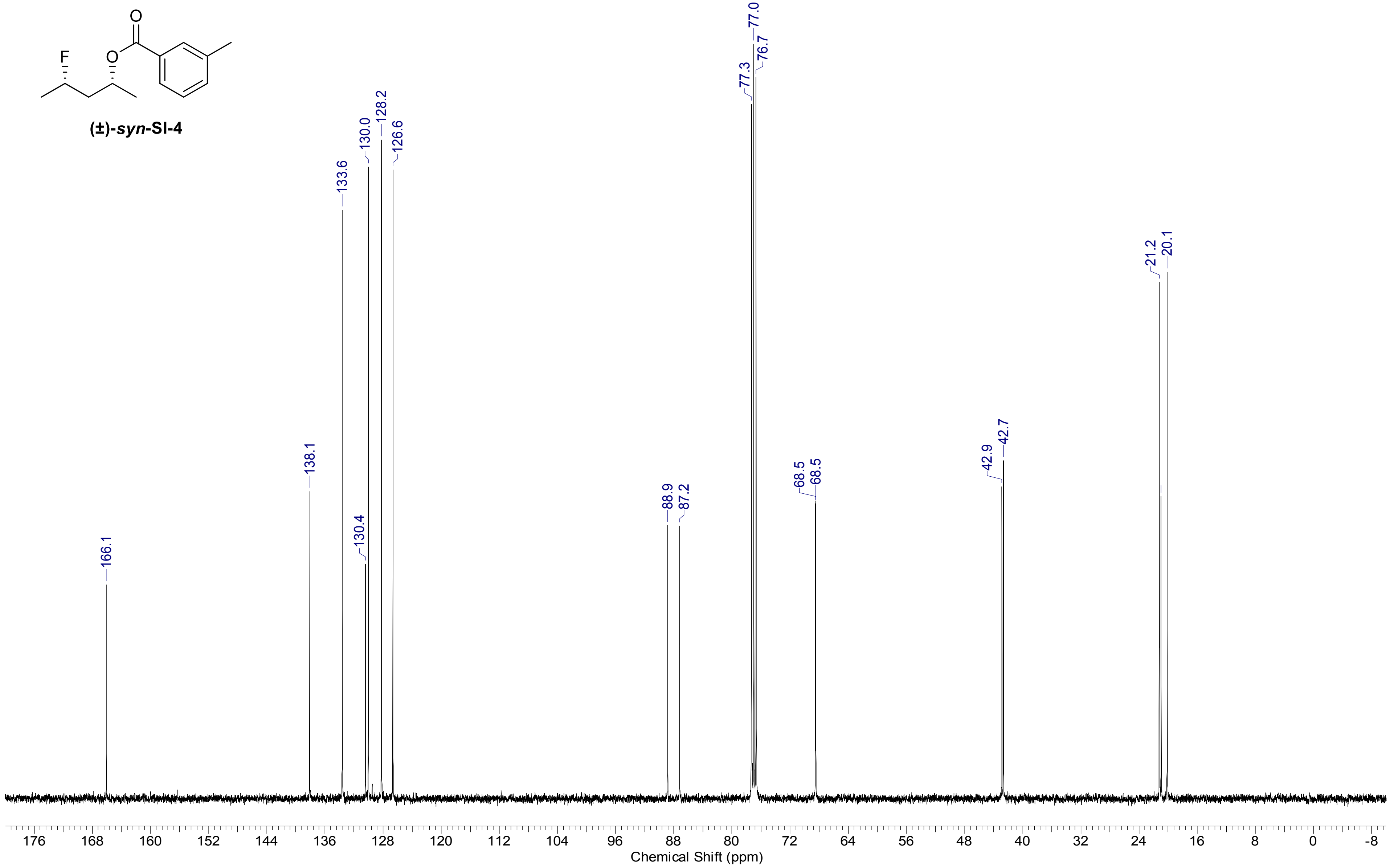

^{19}F NMR, $CDCl_3$, 376 MHz

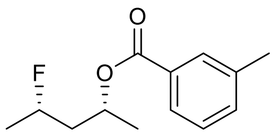

(±)-*syn*-SI-4

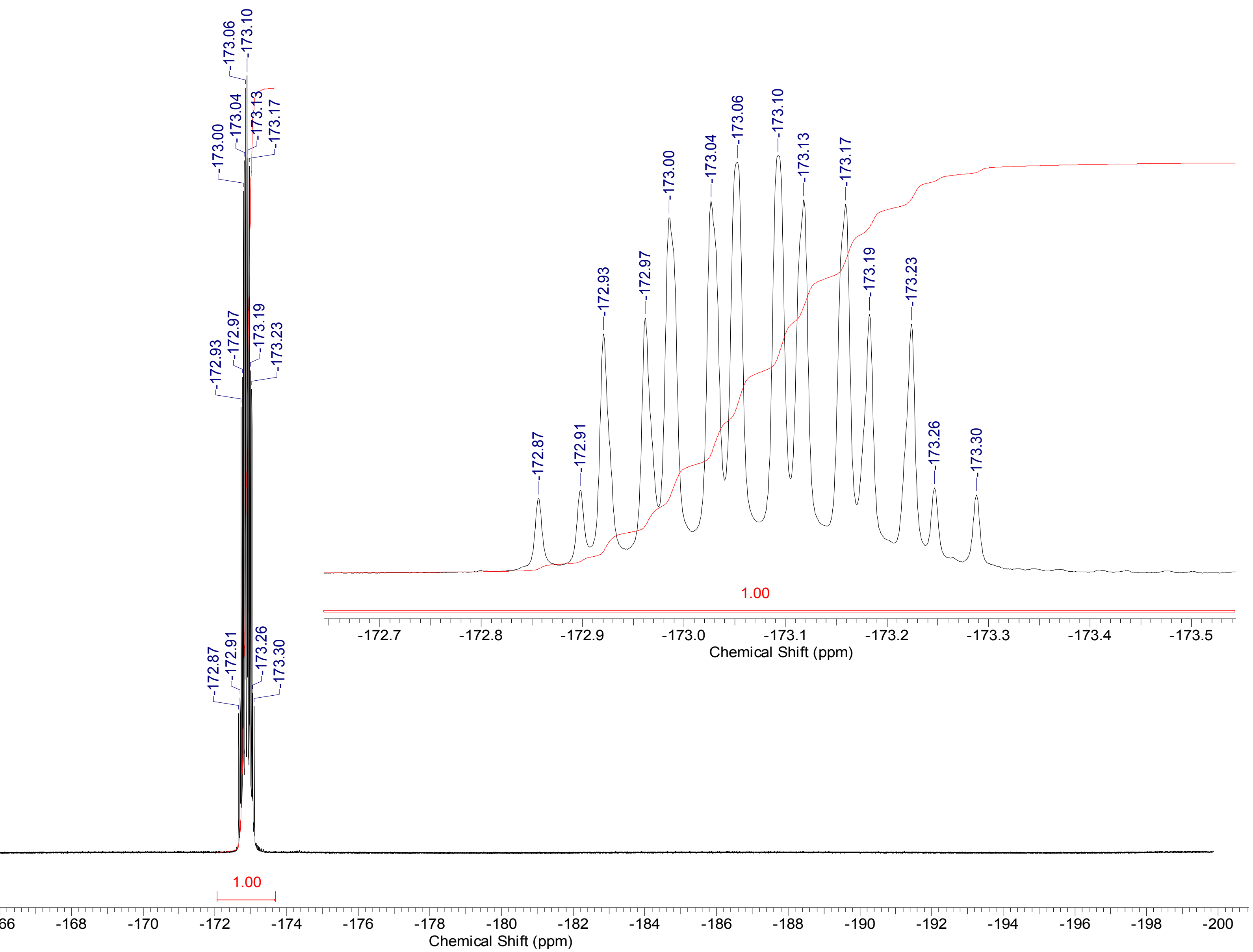

7.1.3 Fluorohydrins (±)-*anti*-A and (±)-*syn*-A

7.1.3.1 (±)-anti-4-Fluoropentan-2-ol (±)-anti-A

^{13}C NMR, CDCl$_3$, 101 MHz

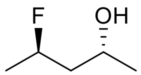

(±)-*anti*-A

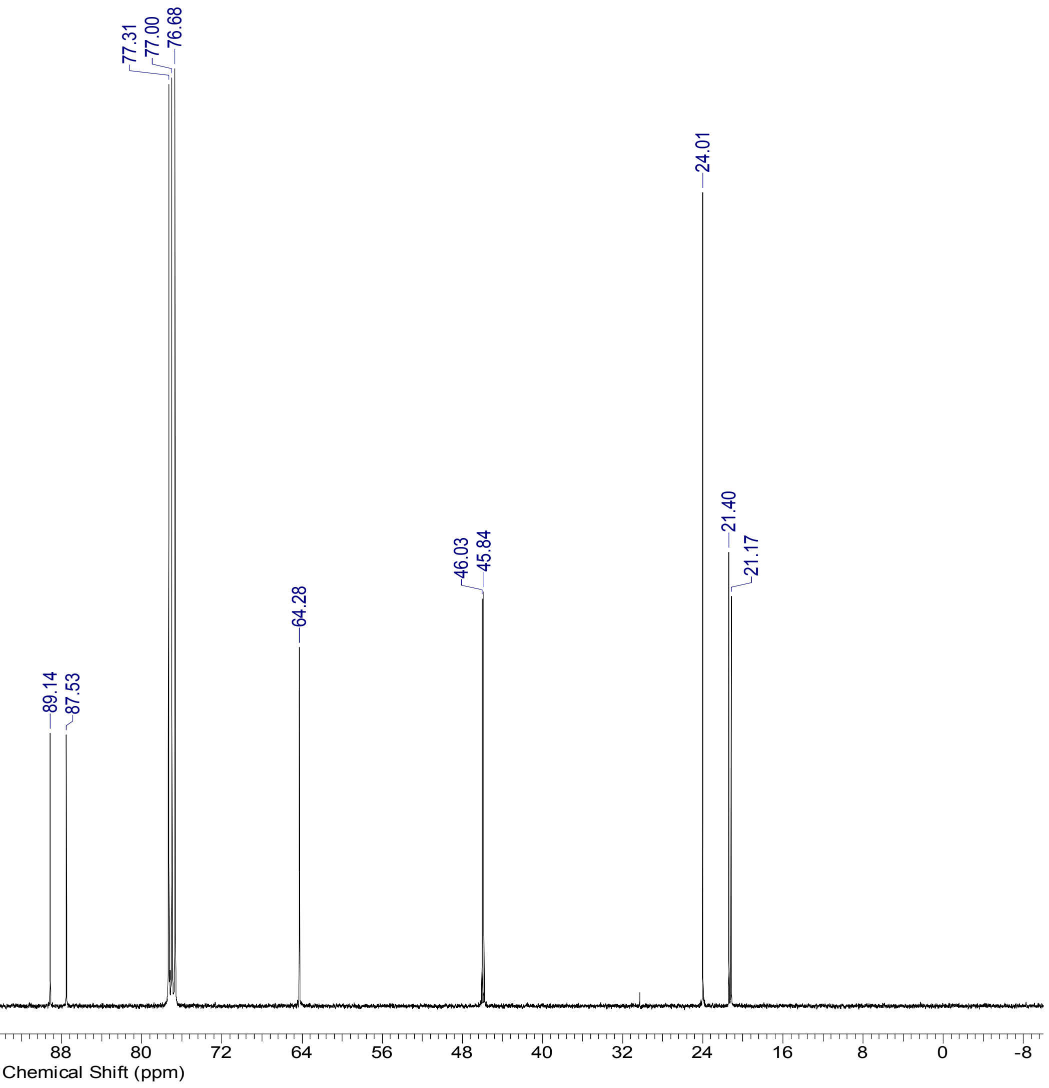

7.1.3.2 (±)-syn-4-Fluoropentan-2-ol (±)-syn-A

^{13}C NMR, $CDCl_3$, 101 MHz

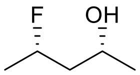

(±)-*syn*-A

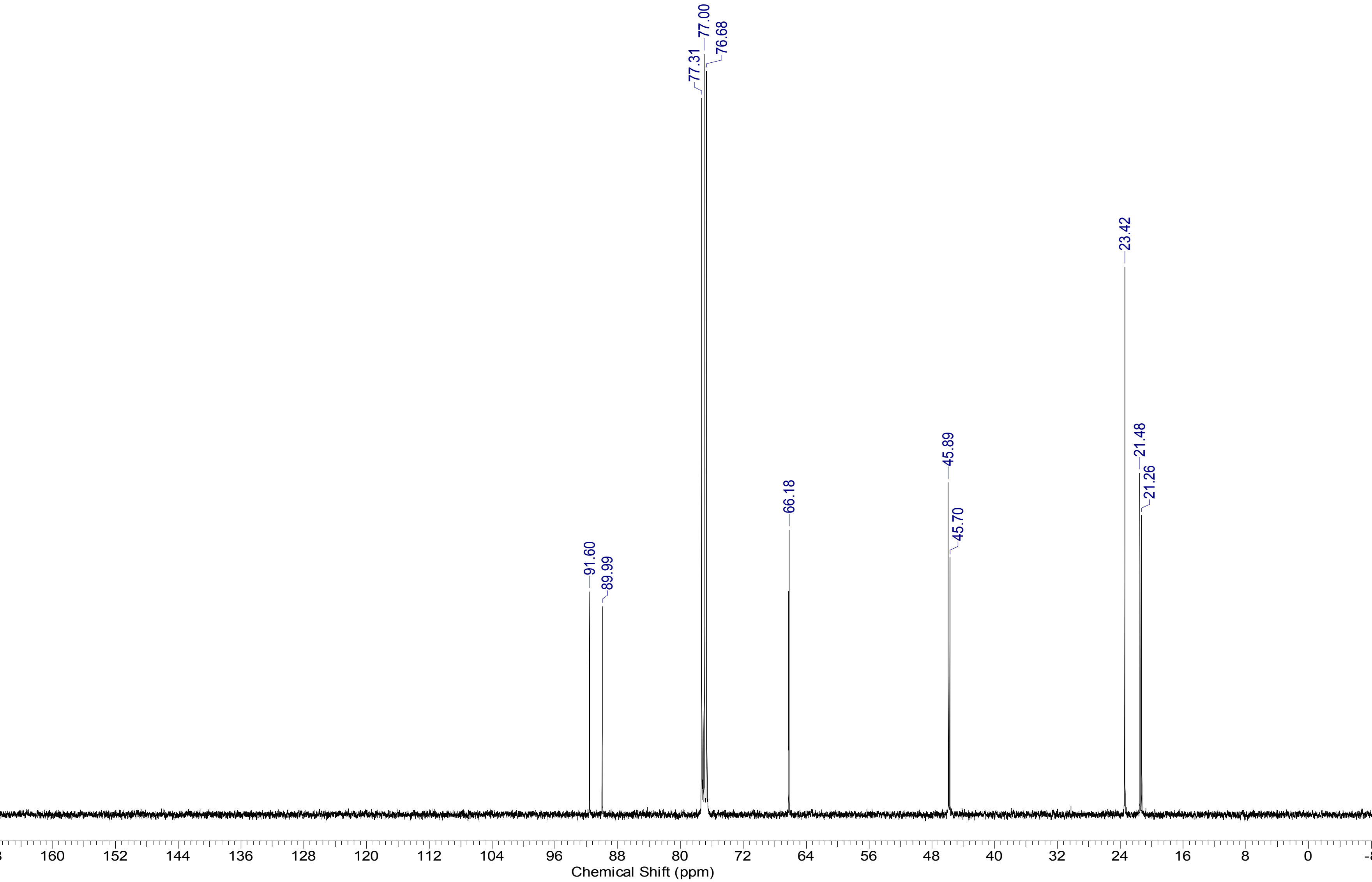

7.2 Synthesis of 4-fluorobutan-2-ol (±)-B

7.2.1 3-Fluoropropanal SI-5

^{1}H NMR, CDCl$_3$, 400 MHz

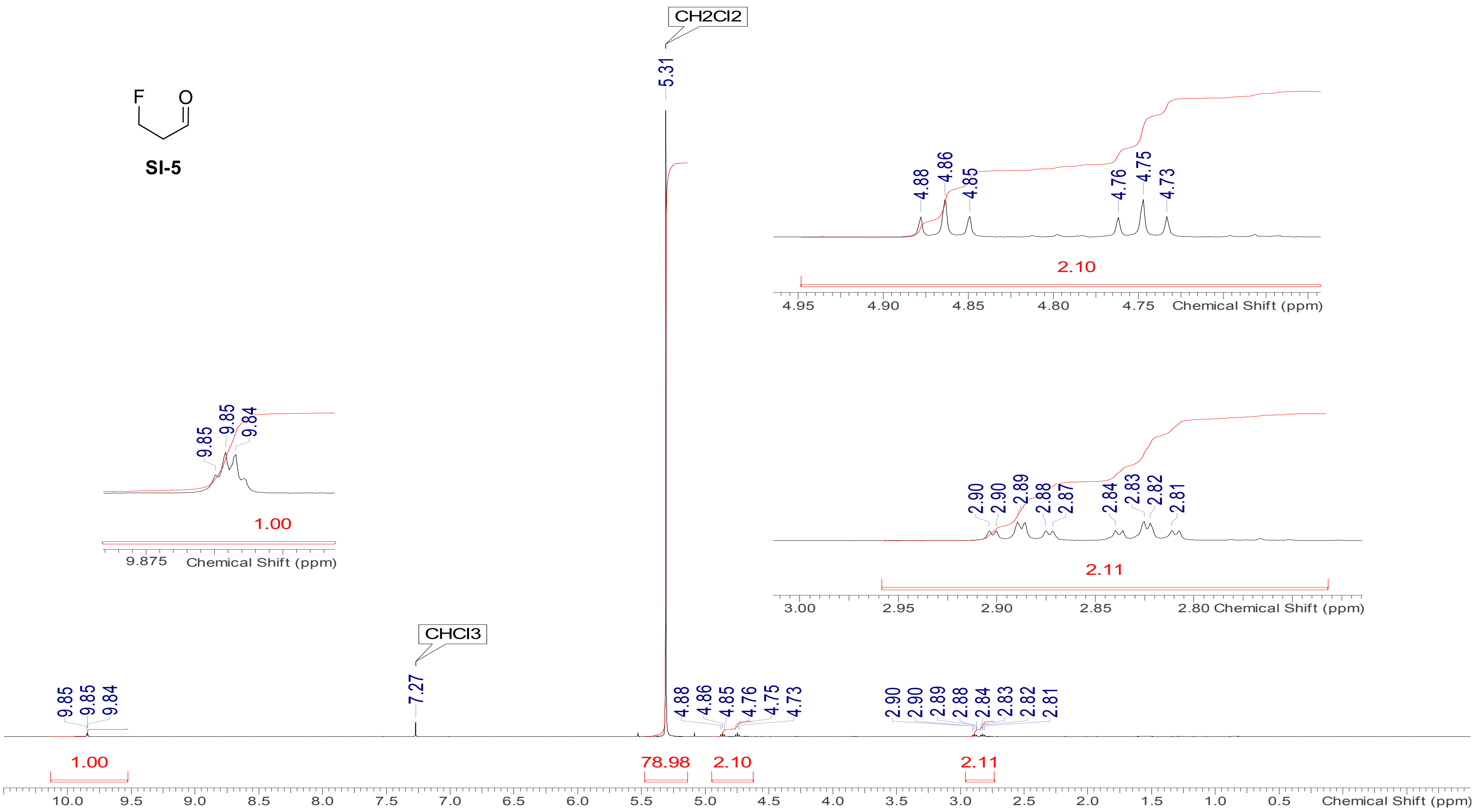

^{19}F NMR, CDCl$_3$, 376 MHz

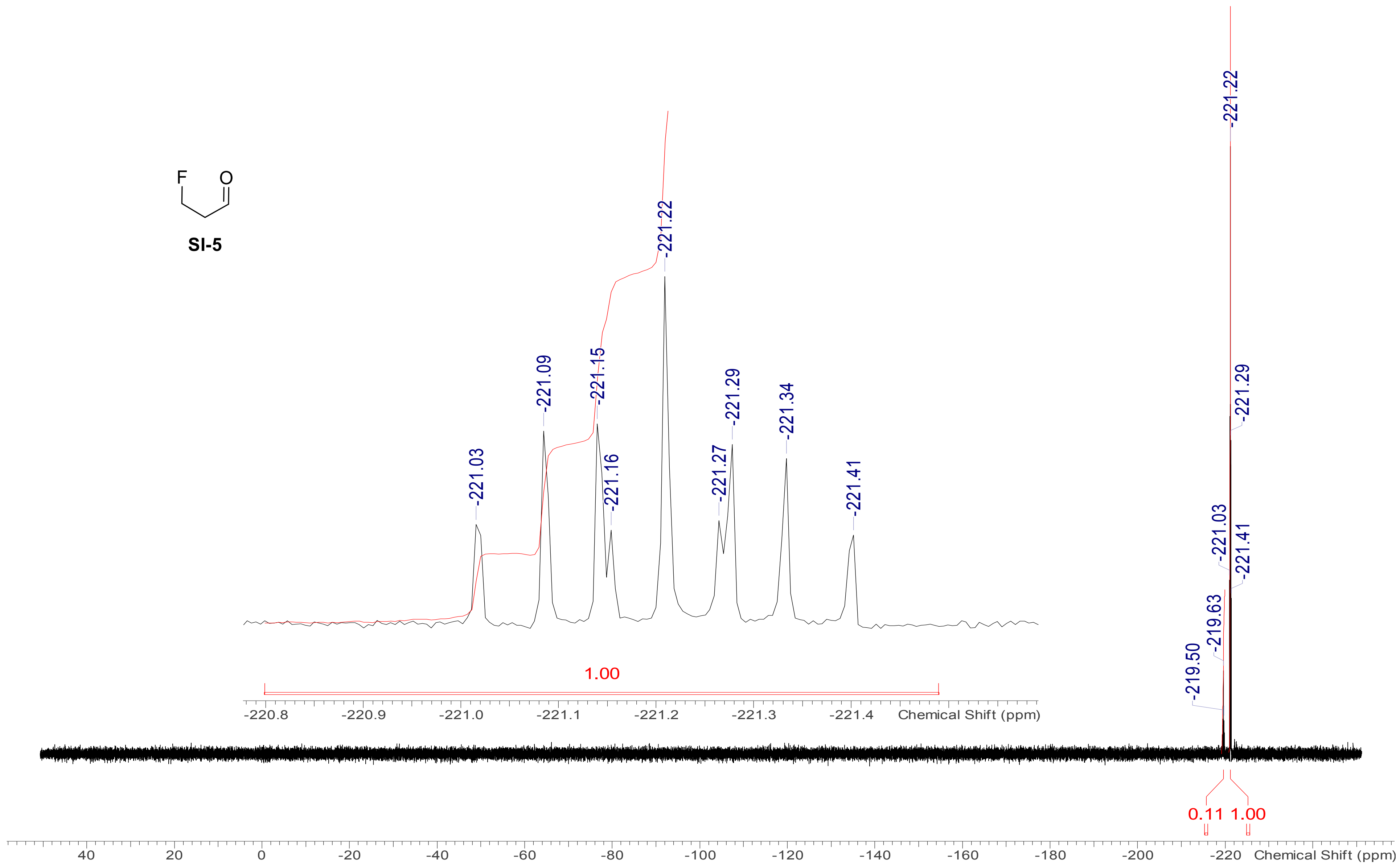
F
O
SI-5
-221.03
-221.09
-221.15
-221.16
-221.22
-221.27
-221.29
-221.34
-221.41
1.00
-220.8
-220.9
-221.0
-221.1
-221.2
-221.3
-221.4
Chemical Shift (ppm)
-221.22
-221.29
-221.03
-221.41
-219.63
-219.50
0.11
1.00
40
20
0
-20
-40
-60
-80
-100
-120
-140
-160
-180
-200
-220
Chemical Shift (ppm)

7.2.2 4-Fluorobutan-2-ol (±)-B

^{13}C NMR, $CDCl_3$, 101 MHz

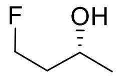

(±)-B

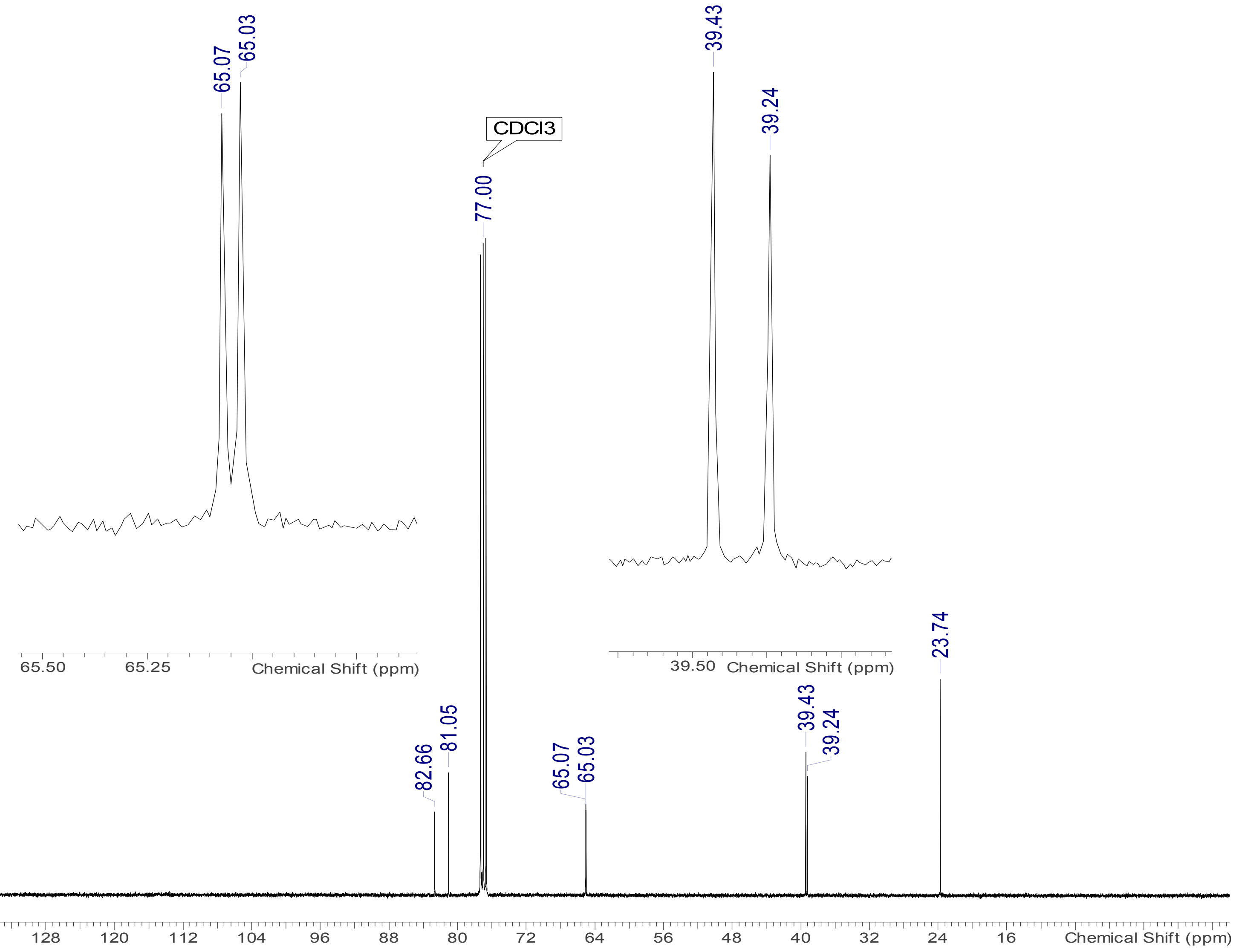

7.3 Synthesis of 3-fluoro-2,2-dimethylpropan1-ol C

7.3.1 3-Fluoro-2,2-dimethyl-1-(*meta*-methylbenzoyloxy)-propane SI-6

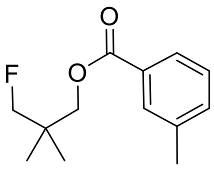

SI-6

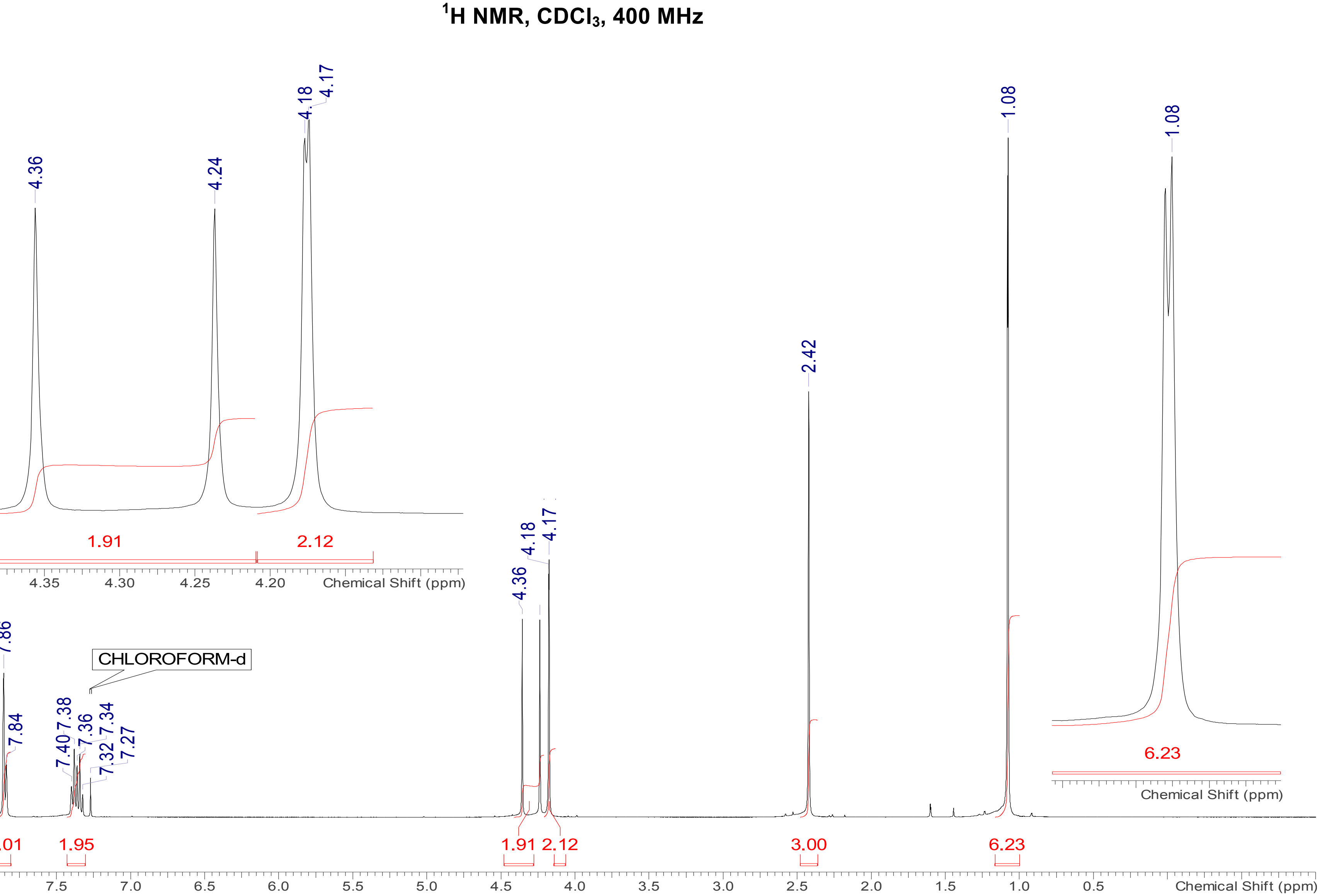

^{13}C NMR, $CDCl_3$, 101 MHz

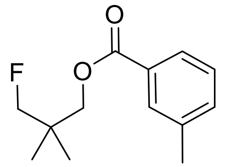

SI-6

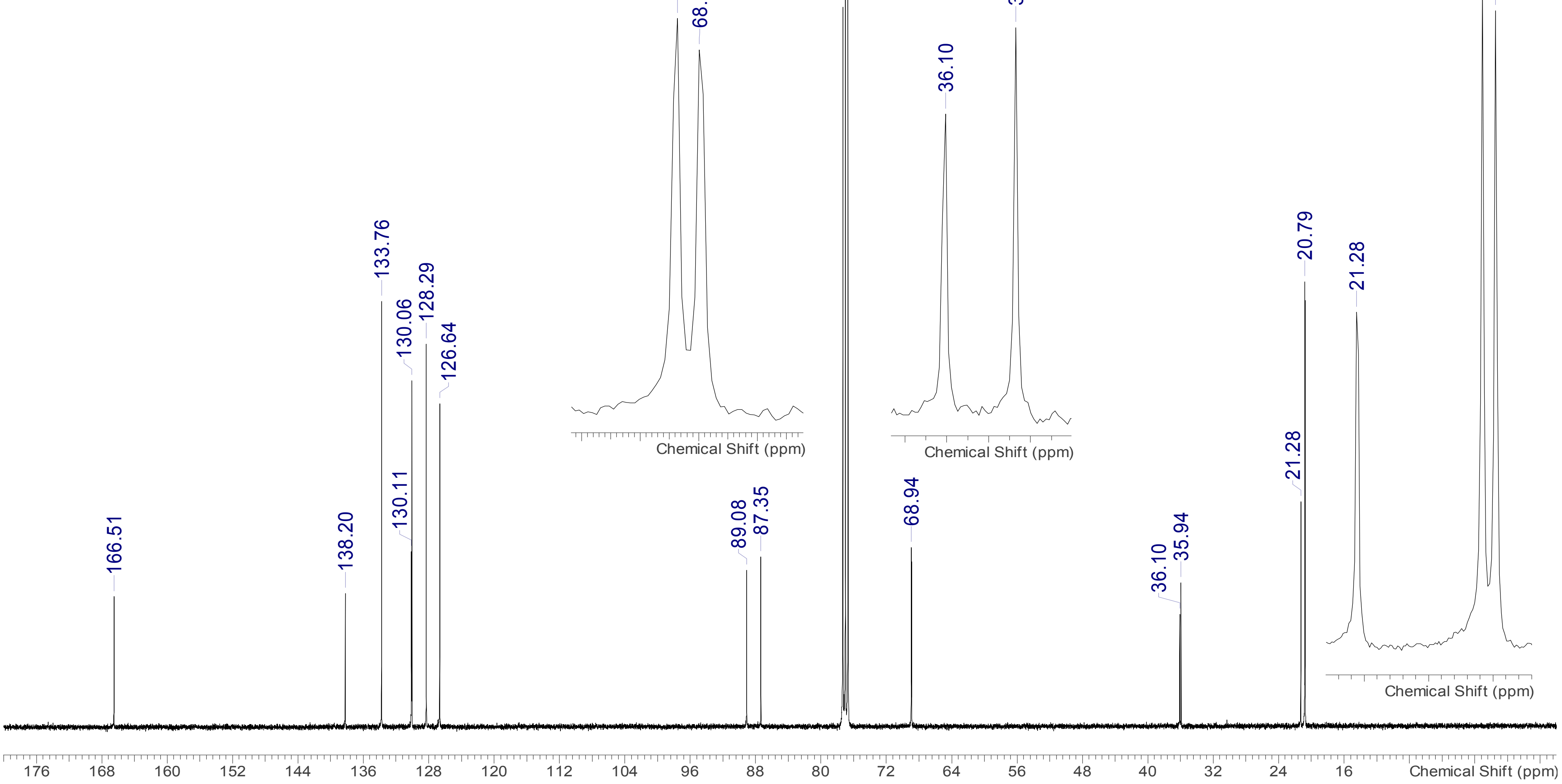

^{19}F NMR, $CDCl_3$, 376 MHz

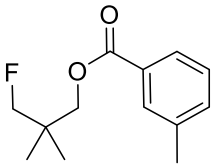

SI-6

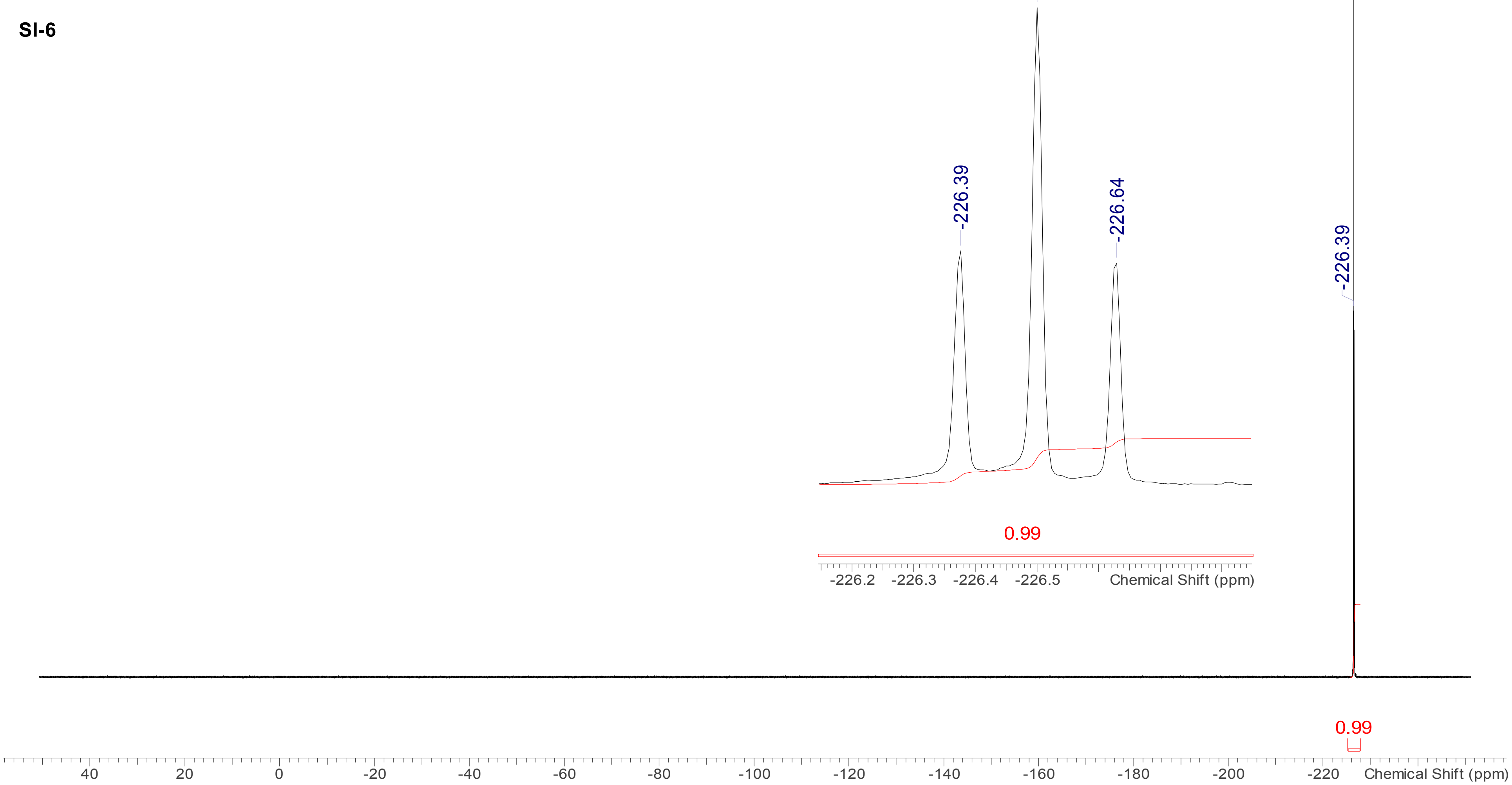

7.3.2 3-Fluoro-2,2-dimethylpropan1-ol C

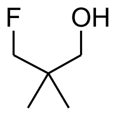

C

^{13}C NMR, $CDCl_3$, 101 MHz

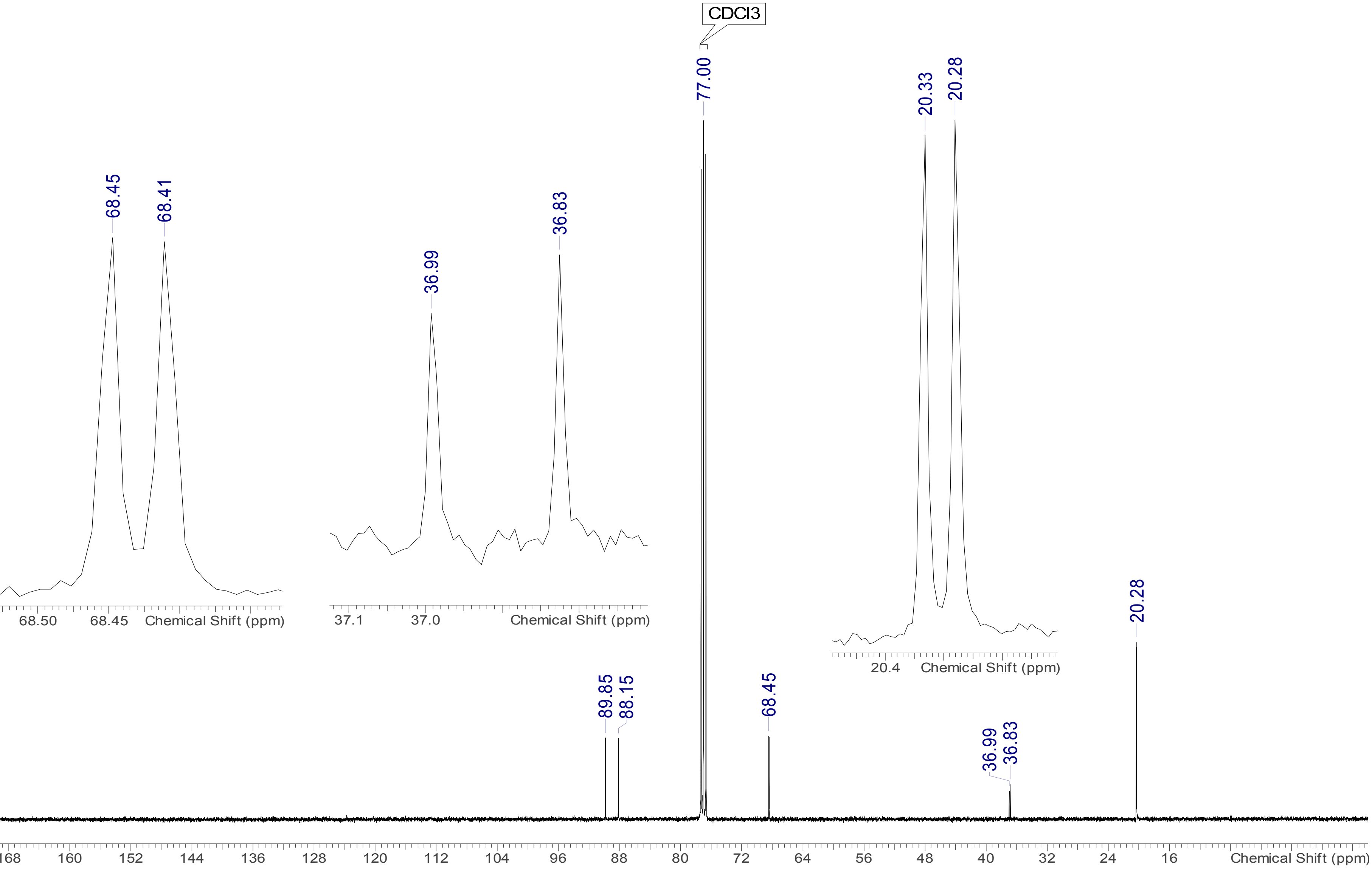

7.4 Synthesis of (±)-4,4-difluoropentan-2-ol (±)-E

7.4.1 4,6-Dimethyl-2-phenyl-1,3-dioxane *meso*-SI-7 and (±)-SI-8

^{1}H NMR, $CDCl_3$, 400 MHz

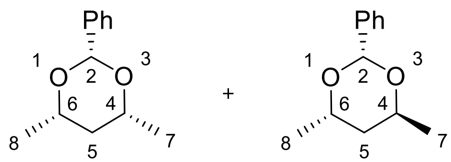

meso-SI-7 (±)-SI-8

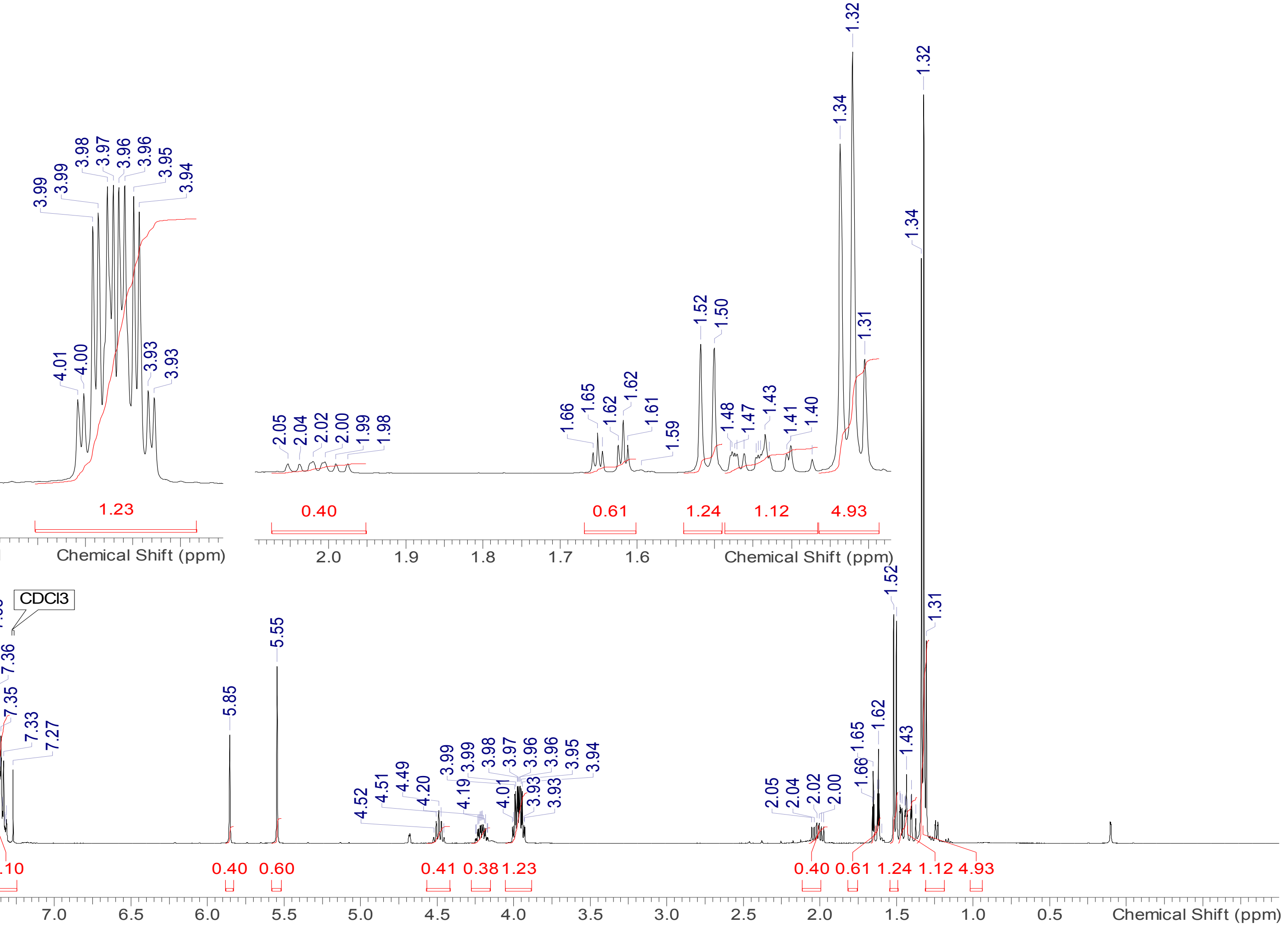

^{13}C NMR, $CDCl_3$, 101 MHz

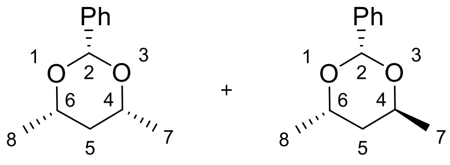

***meso*-SI-7** **(±)-SI-8**

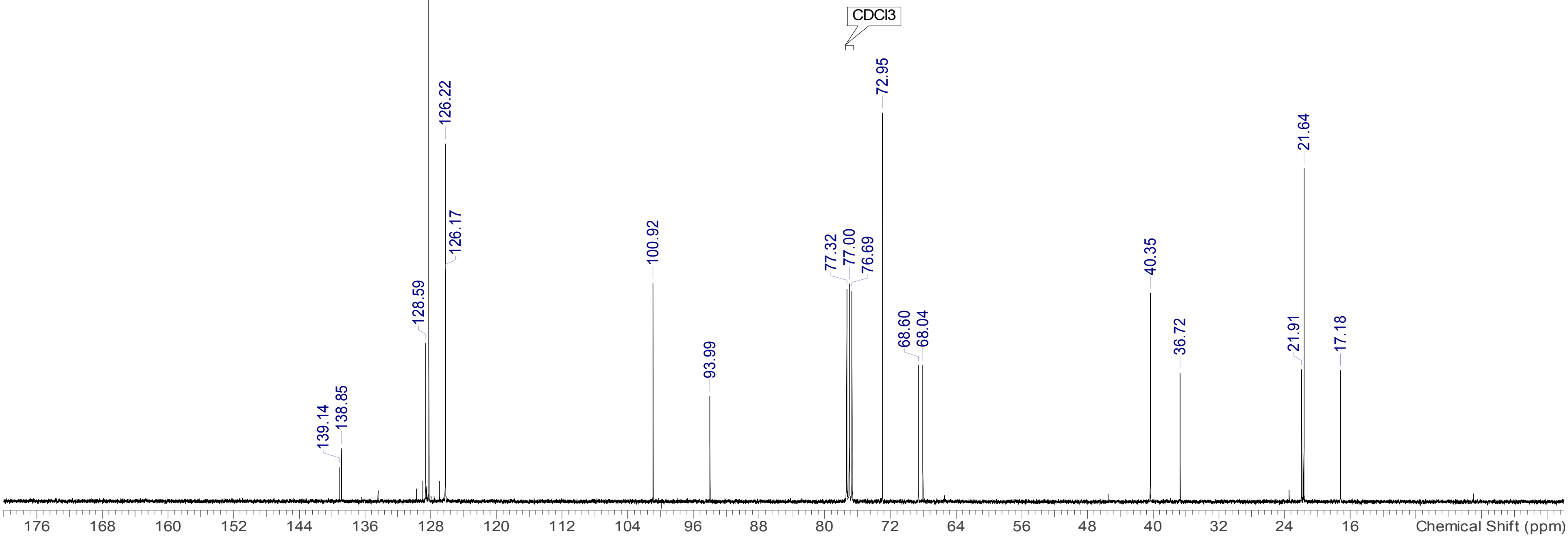

7.4.2 (2*S**,4*R**)-4-Benzyloxypentan-2-ol (±)-*syn*-SI-9 and (2*R**,4*R**)-4-benzyloxypentan-2-ol (±)-*anti*-SI-10

^{1}H NMR, CDCl$_3$, 400 MHz

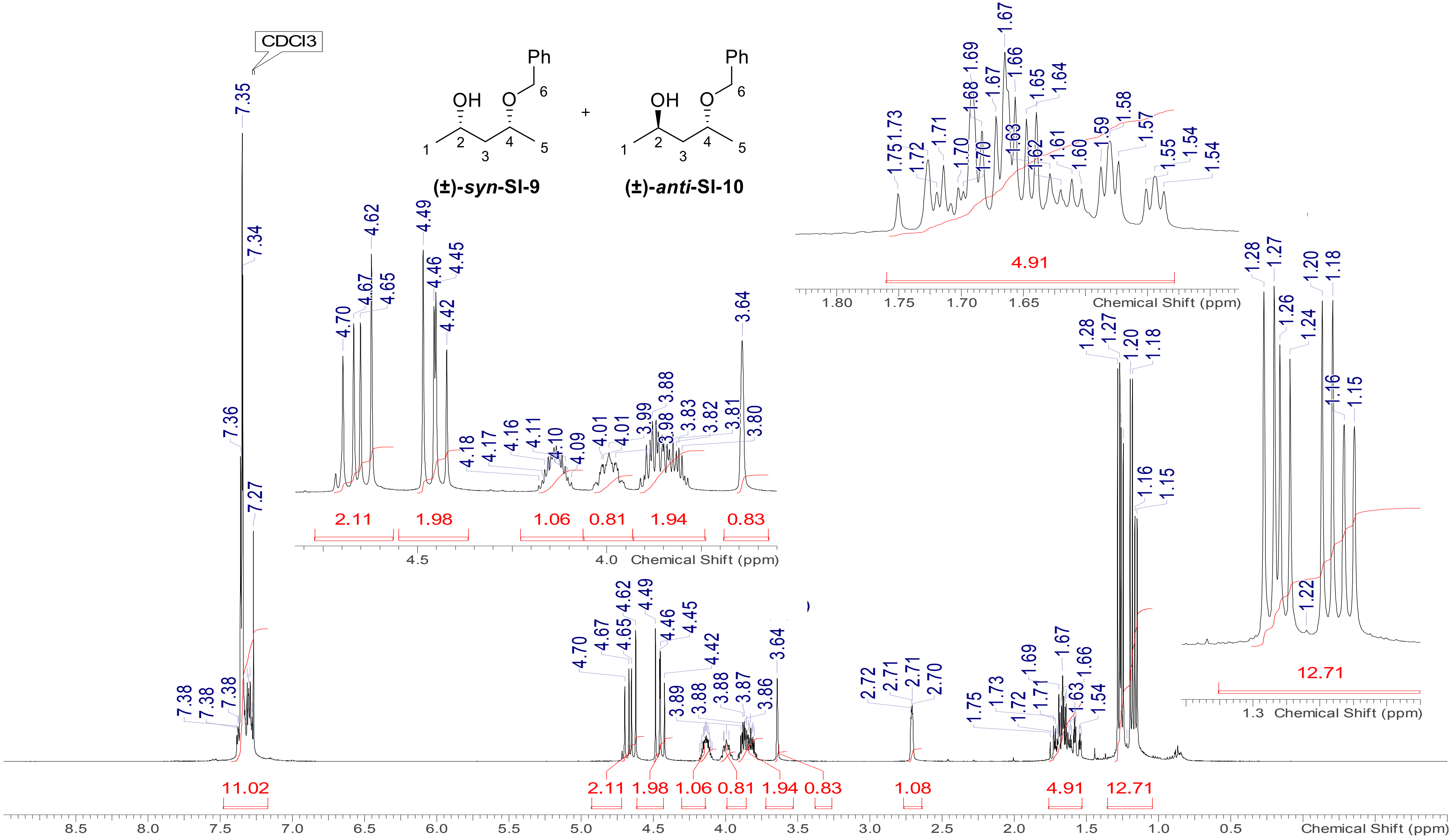

^{13}C NMR, $CDCl_3$, 101 MHz

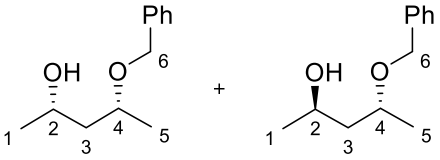

(±)-*syn*-SI-9 **(±)-*anti*-SI-10**

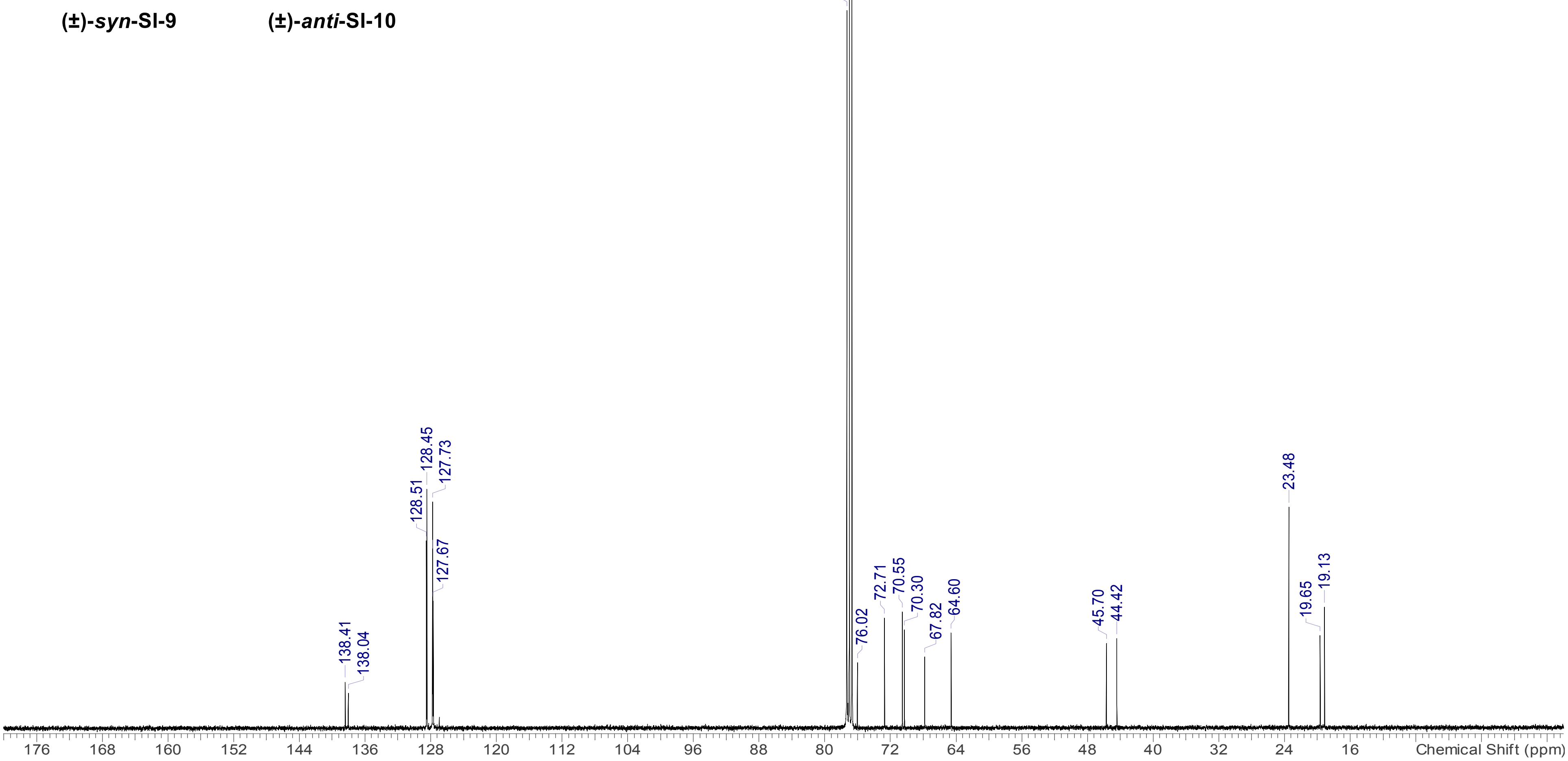

7.4.3 (±)-4-Benzyloxypentan-2-one (±)-SI-11

^{1}H NMR, $CDCl_3$, 400 MHz

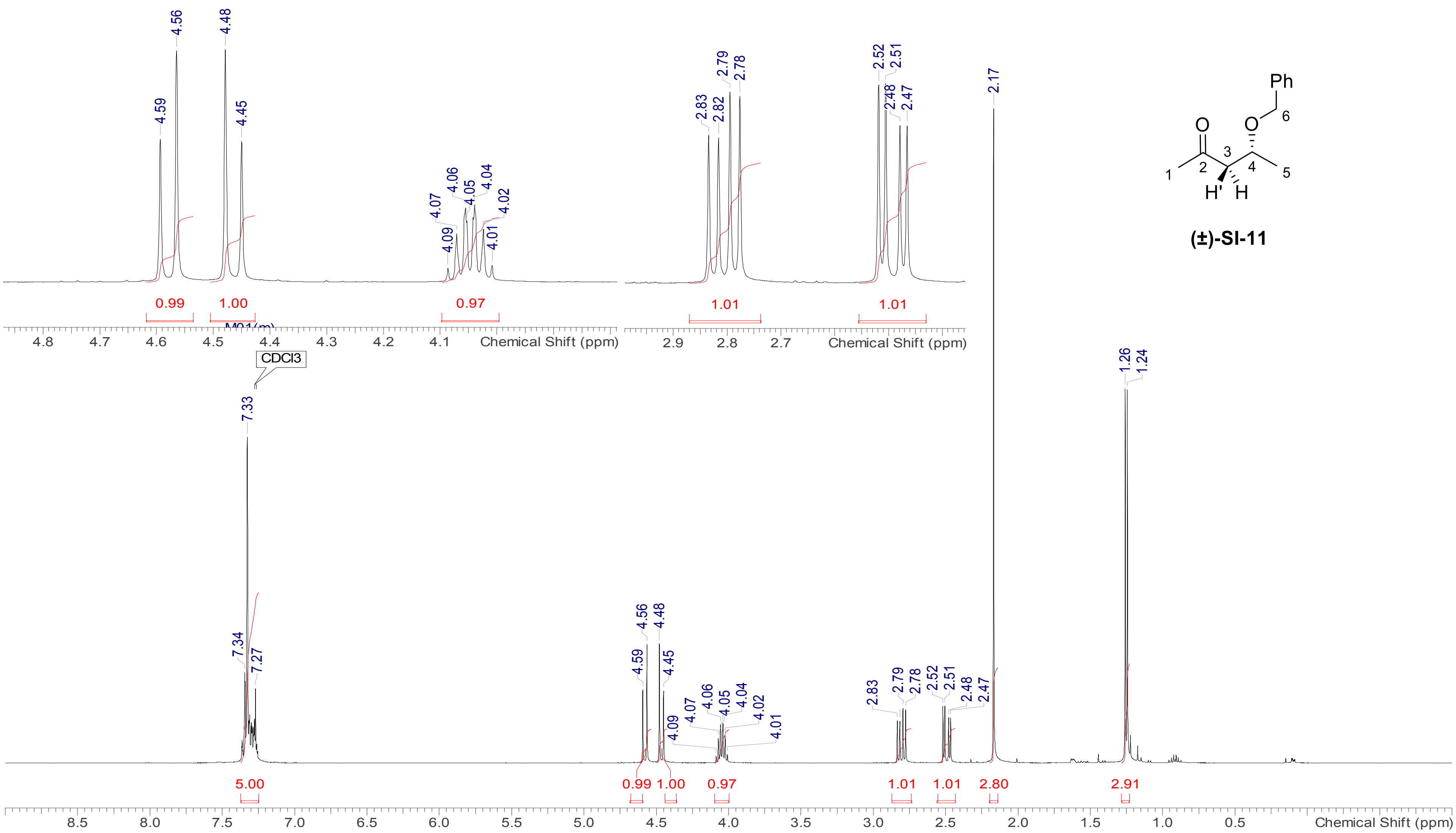

(±)-SI-11

^{13}C NMR, $CDCl_3$, 101 MHz

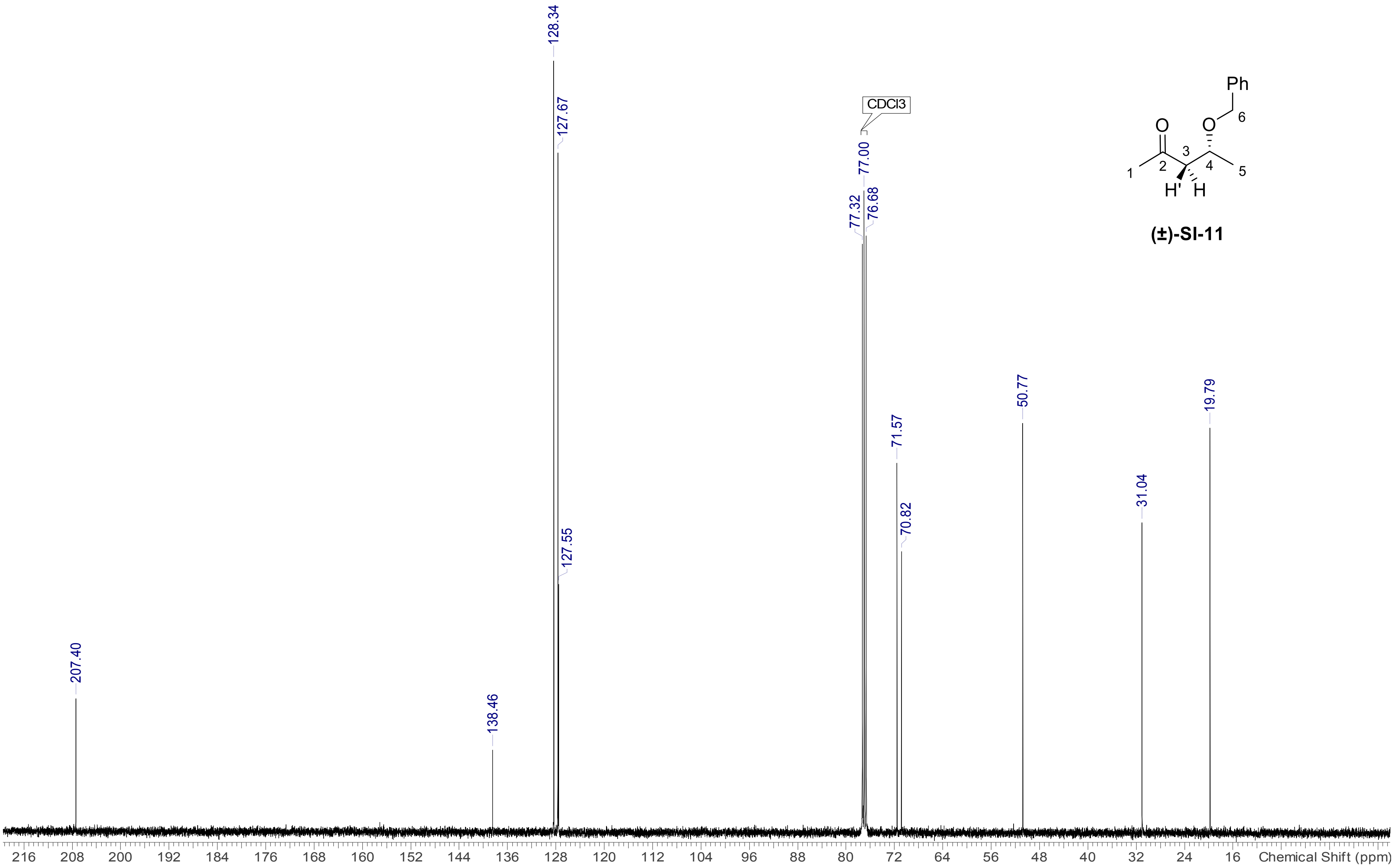

(±)-SI-11

7.4.4 (±)-2-Benzyloxy-4,4-difluoropentane (±)-SI-12

^{1}H NMR, $CDCl_3$, 400 MHz

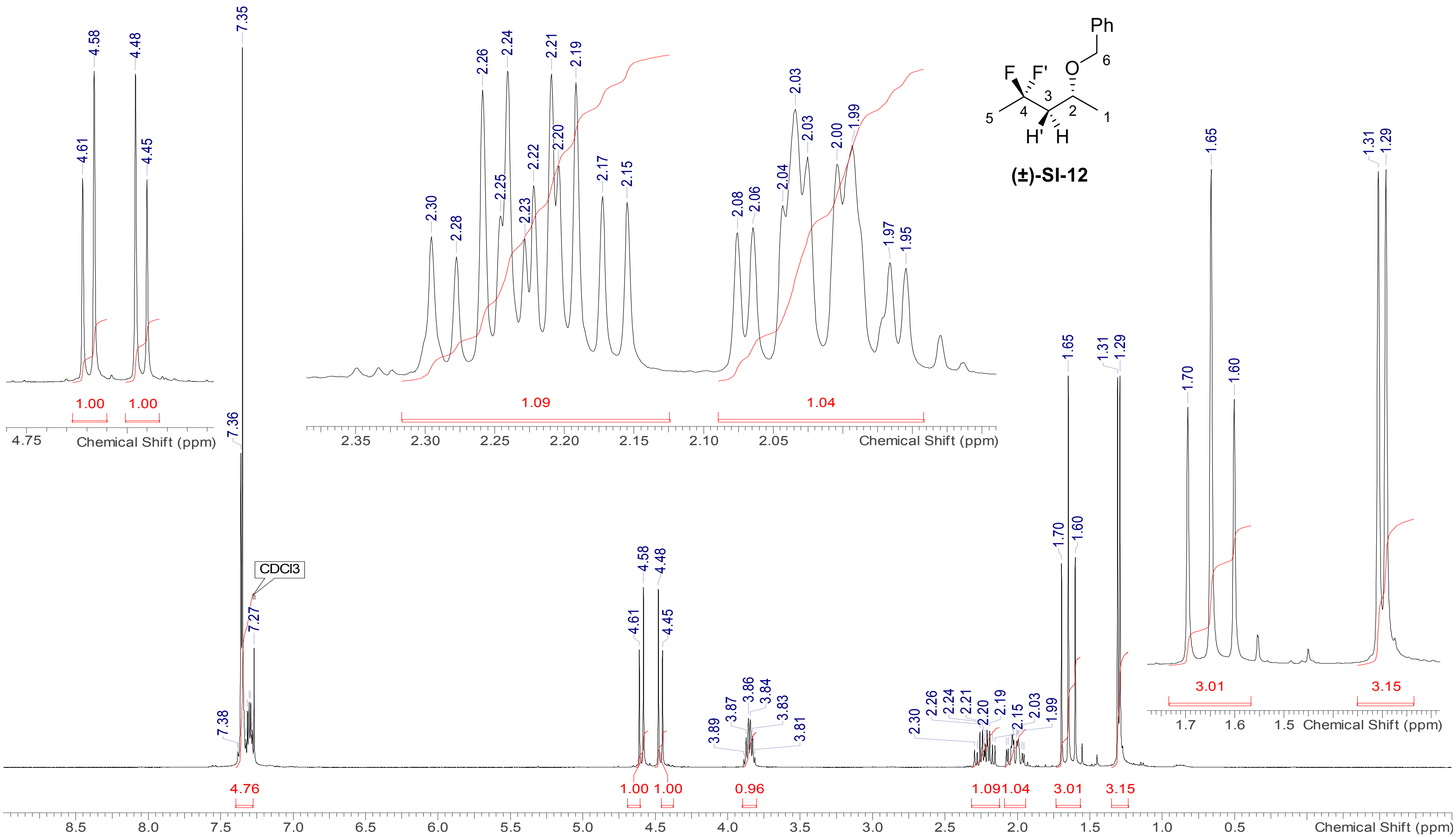

^{13}C NMR, $CDCl_3$, 101 MHz

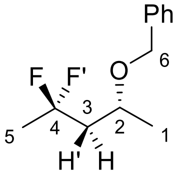

(±)-SI-12

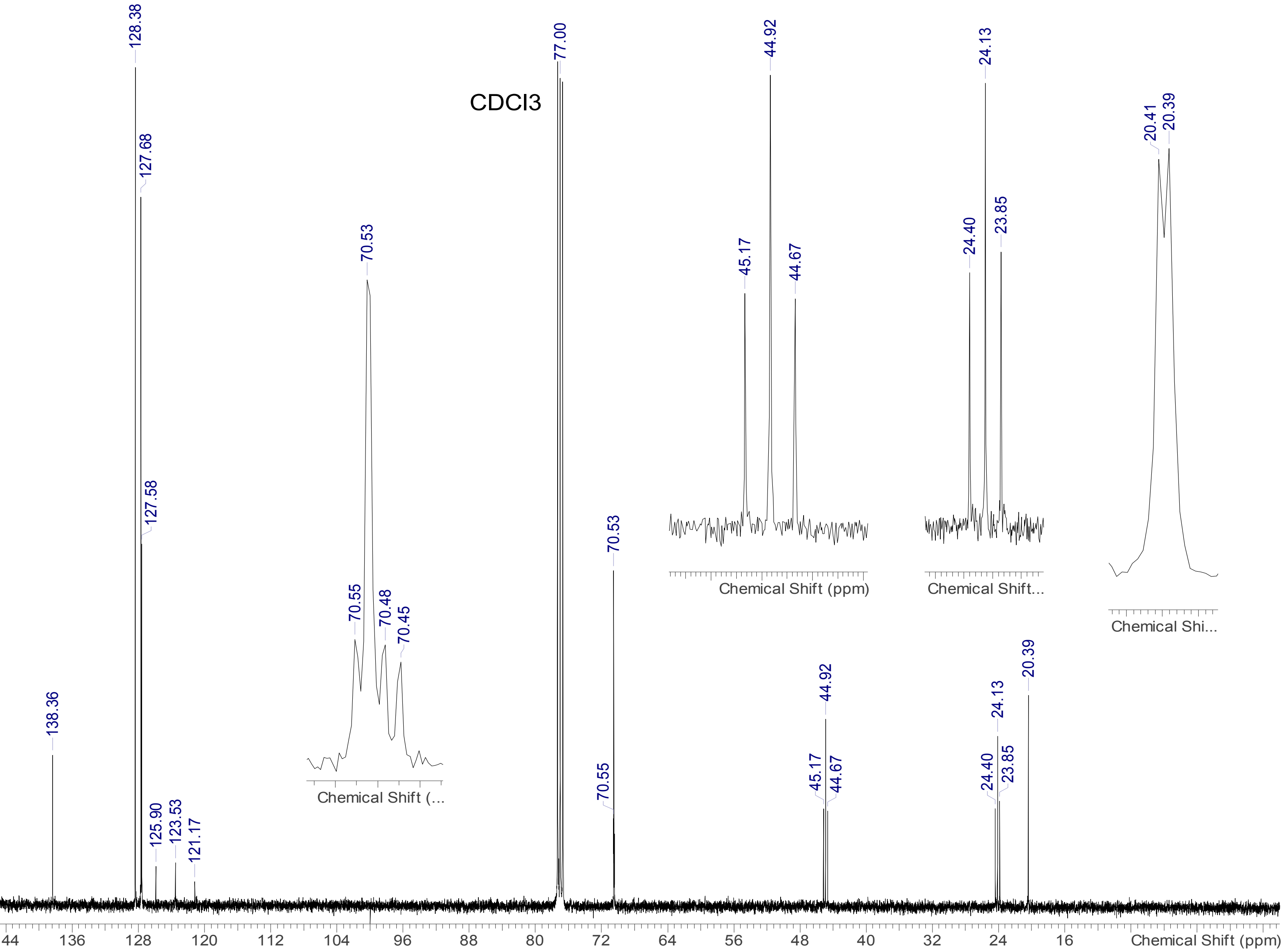

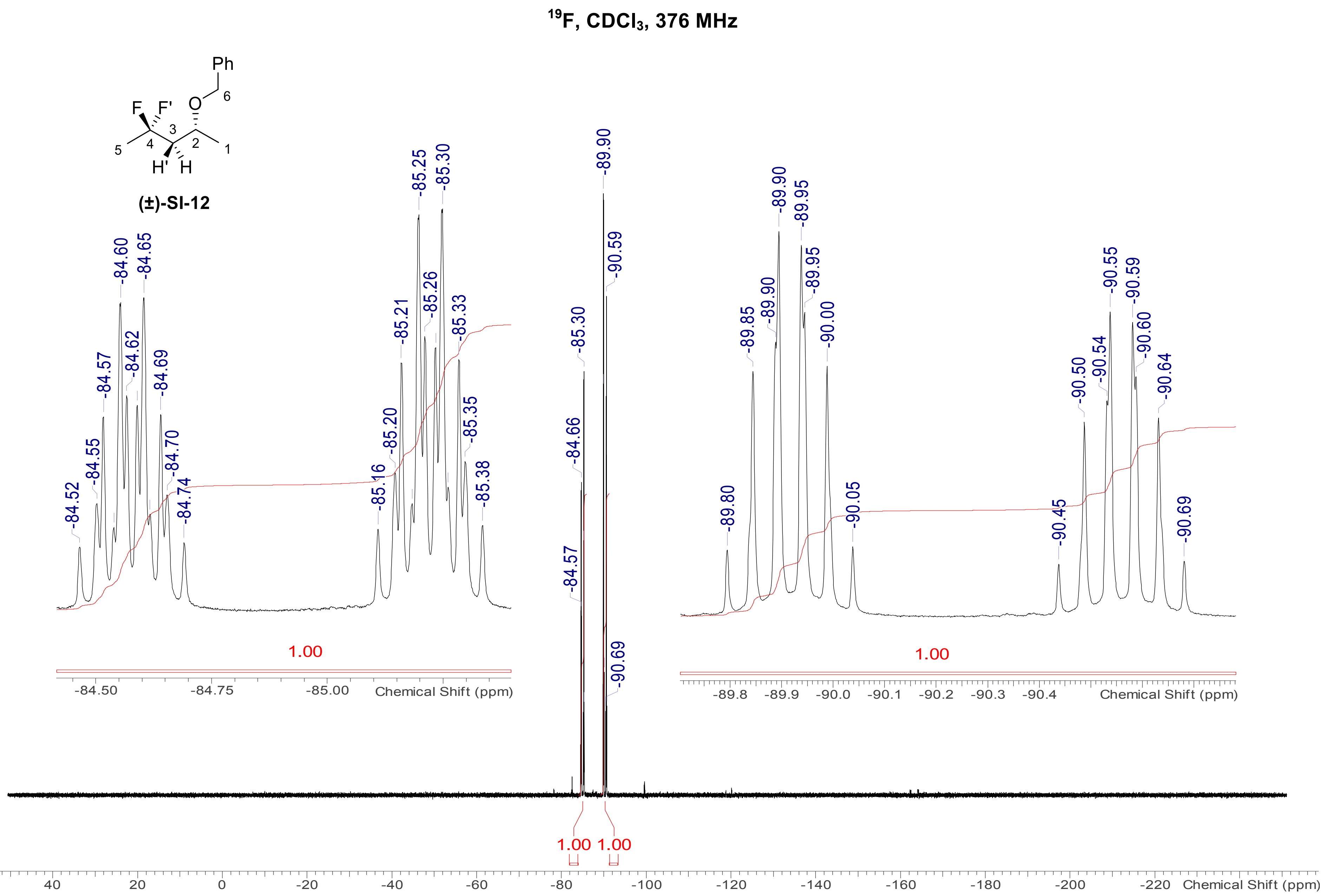

19F, CDCl3, 376 MHz
Ph
6
O
F F'
3
5 4 2 1
H' H
(±)-SI-12
-84.52
-84.55
-84.57
-84.60
-84.62
-84.65
-84.69
-84.70
-84.74
-85.16
-85.20
-85.21
-85.25
-85.26
-85.30
-85.33
-85.35
-85.38
1.00
-84.50
-84.75
-85.00
Chemical Shift (ppm)
-84.57
-84.66
-85.30
-89.90
-90.59
-90.69
-89.80
-89.85
-89.90
-89.90
-89.95
-89.95
-90.00
-90.05
-90.45
-90.50
-90.54
-90.55
-90.59
-90.60
-90.64
-90.69
1.00
-89.8
-89.9
-90.0
-90.1
-90.2
-90.3
-90.4
Chemical Shift (ppm)
1.00 1.00
40
20
0
-20
-40
-60
-80
-100
-120
-140
-160
-180
-200
-220
Chemical Shift (ppm)

7.4.5 (±)-4,4-difluoropentan-2-ol (±)-E

^{13}C NMR, $CDCl_3$, 101 MHz

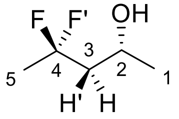

(±)-E

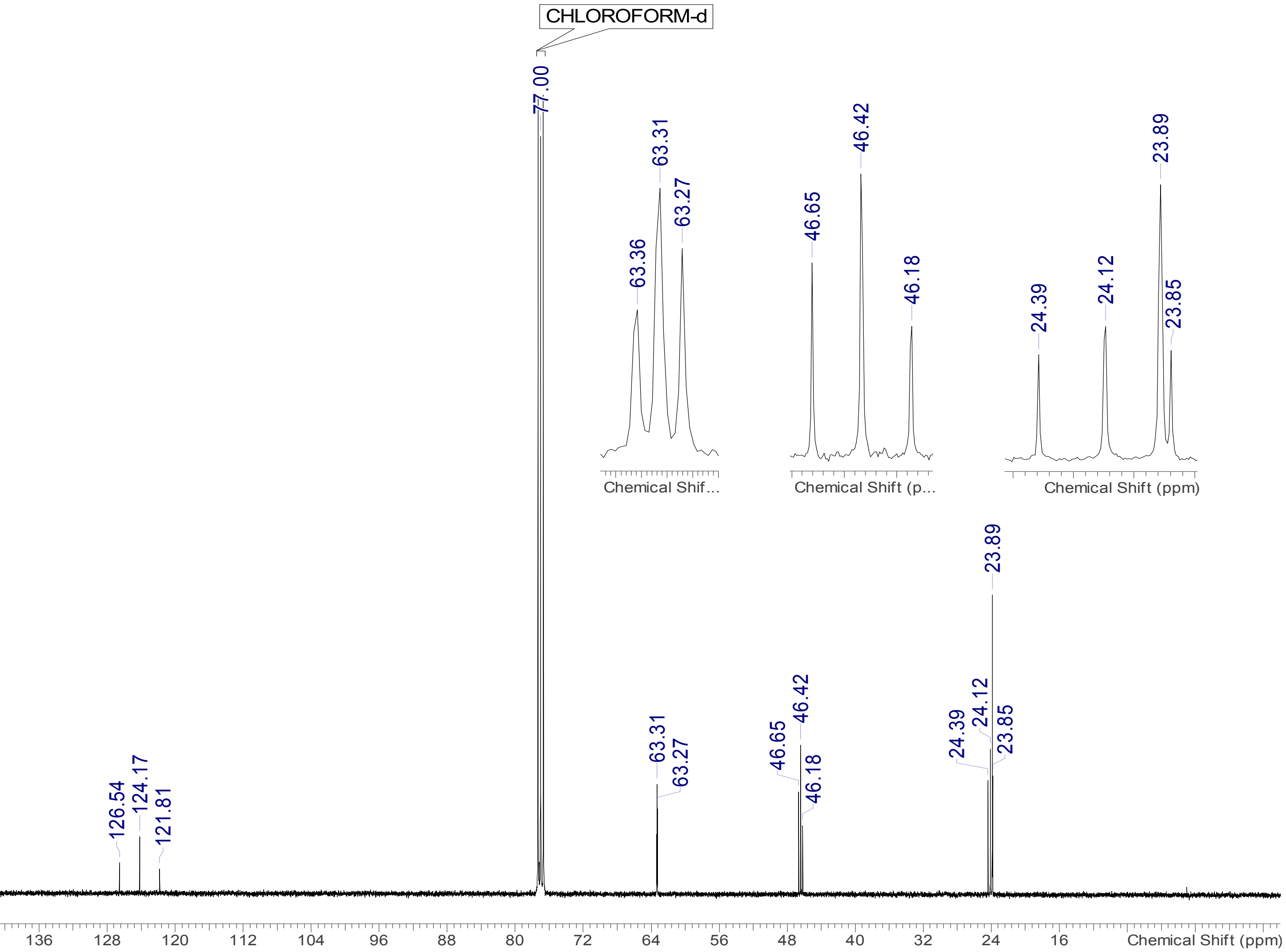

7.5 Synthesis of (±)-4,4-difluorobutan-2-ol (±)-F

7.5.1 (±)-3-Benzyloxy-butanal (±)-SI-15

^{1}H NMR, $CDCl_3$, 400 MHz

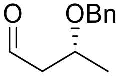

(±)-SI-15

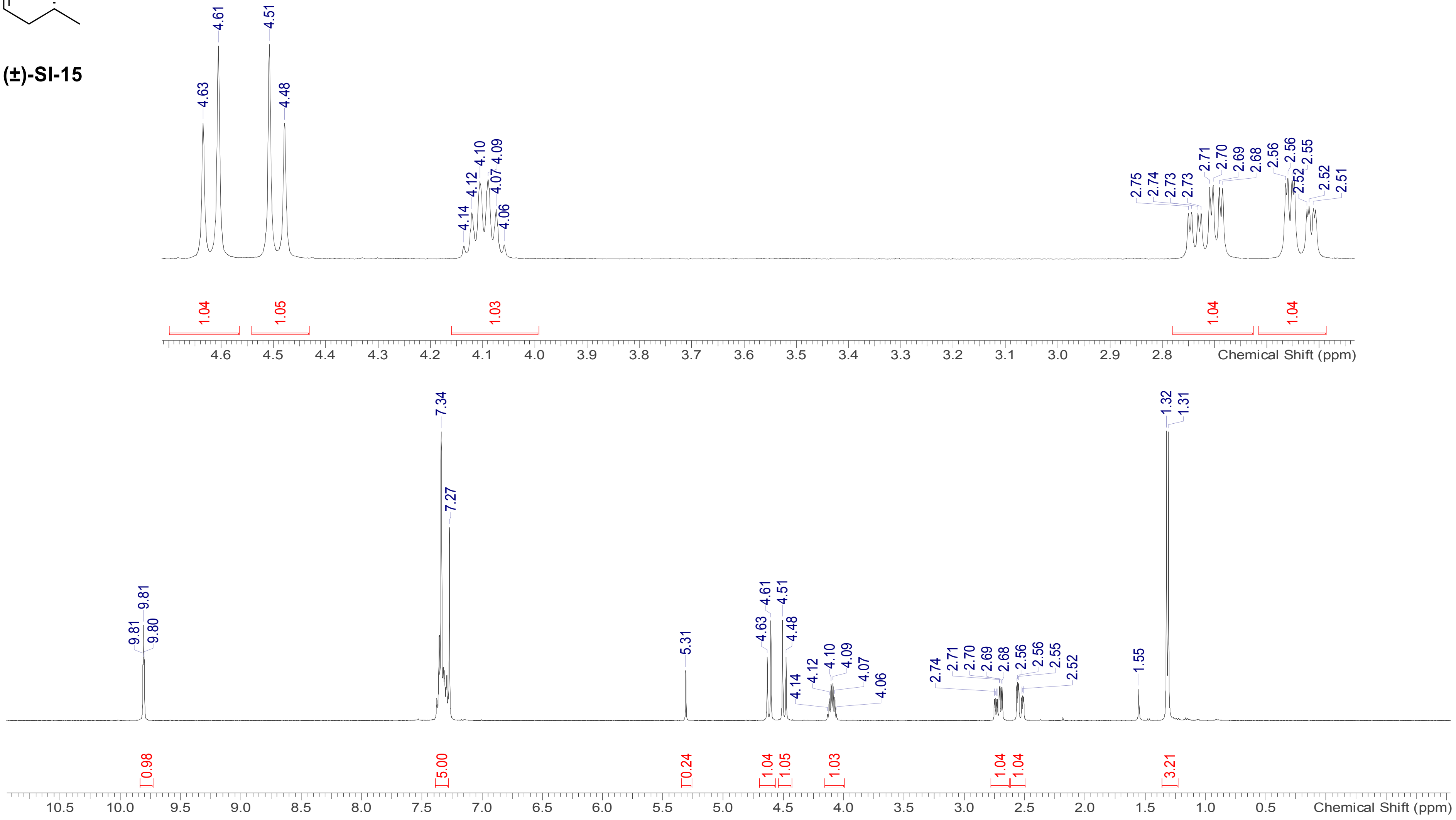

^{13}C NMR, $CDCl_3$, 101 MHz

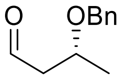

(±)-SI-15

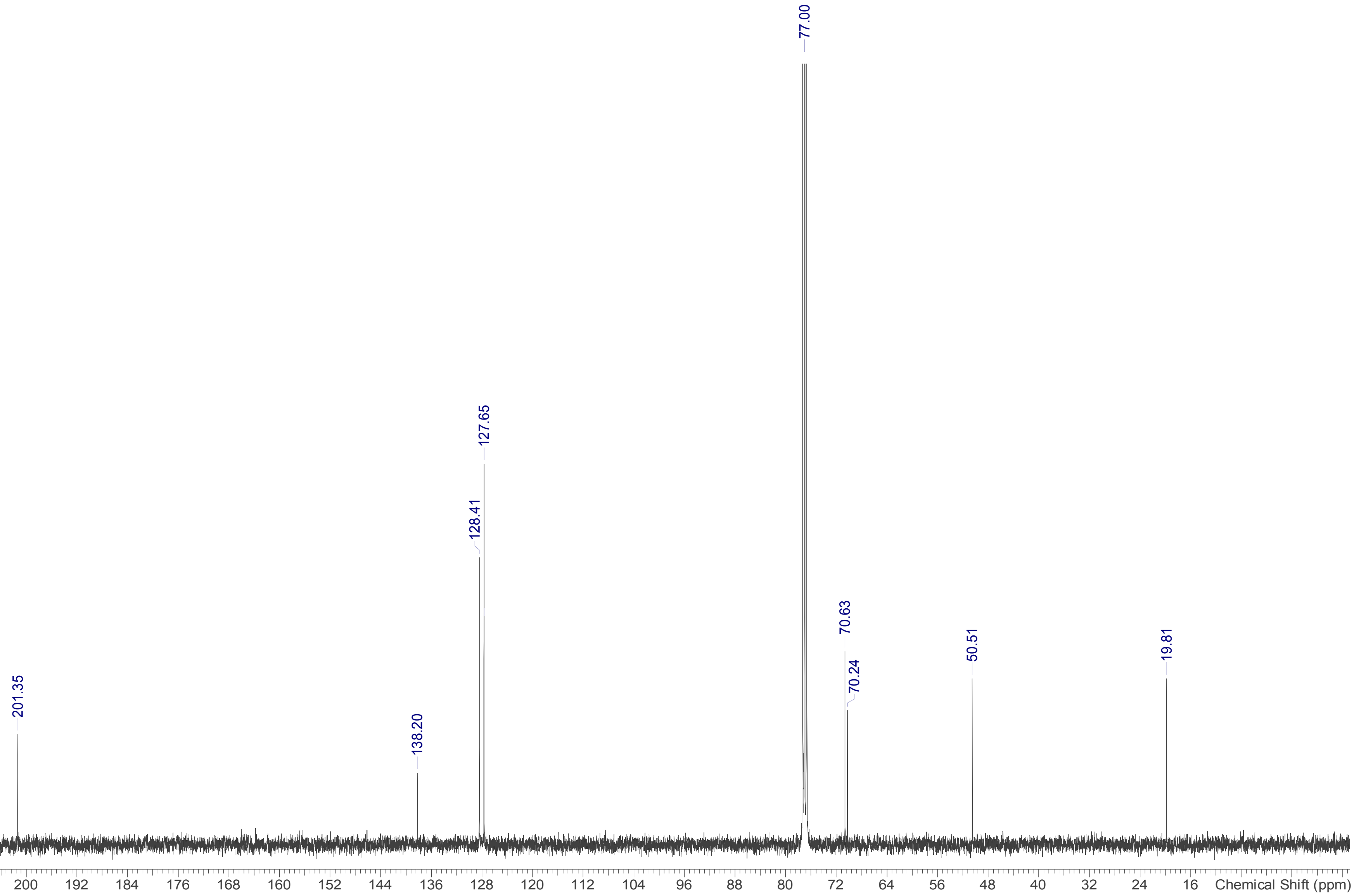

7.5.2 (±)-2-Benzyloxy-4,4-difluorobutane (±)-SI-16

^{1}H NMR, $CDCl_3$, 400 MHz

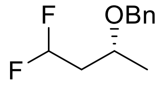

(±)-SI-16

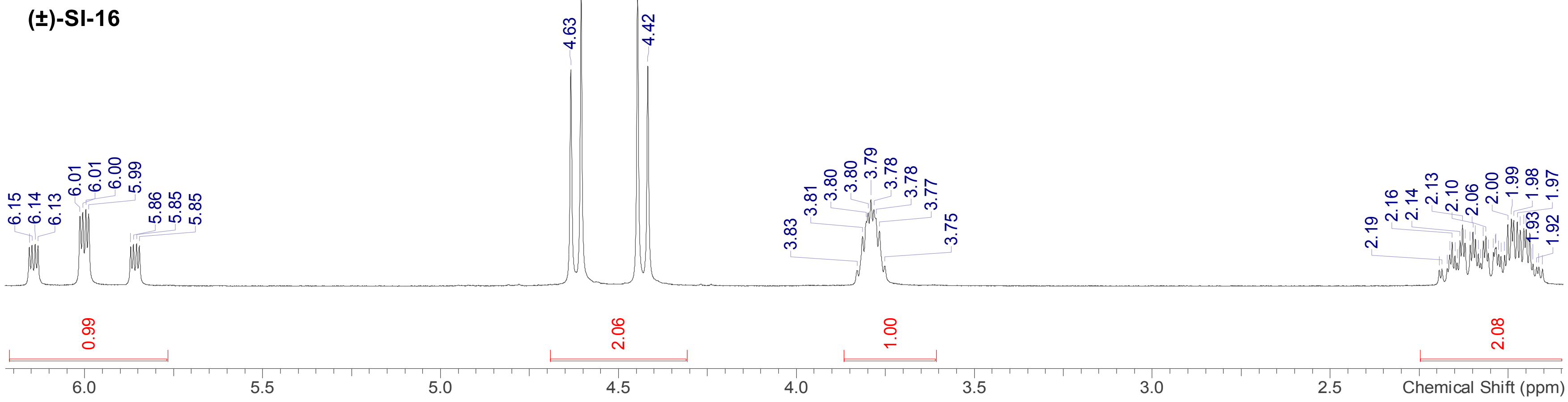

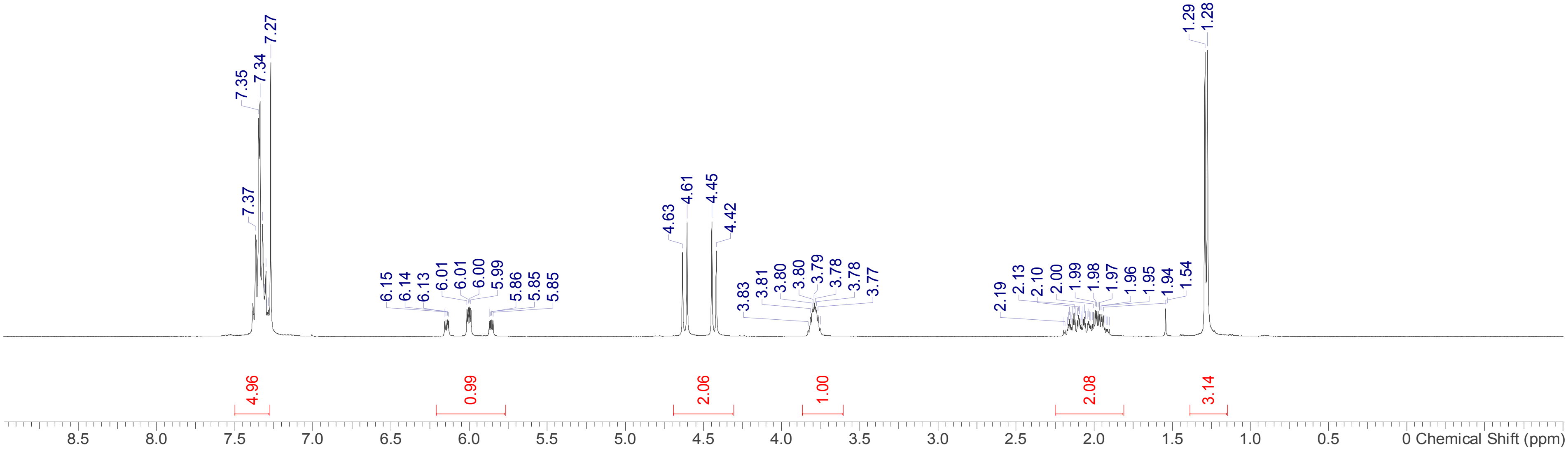

^{13}C NMR, $CDCl_3$, 101 MHz

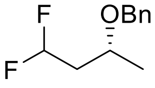

(±)-SI-16

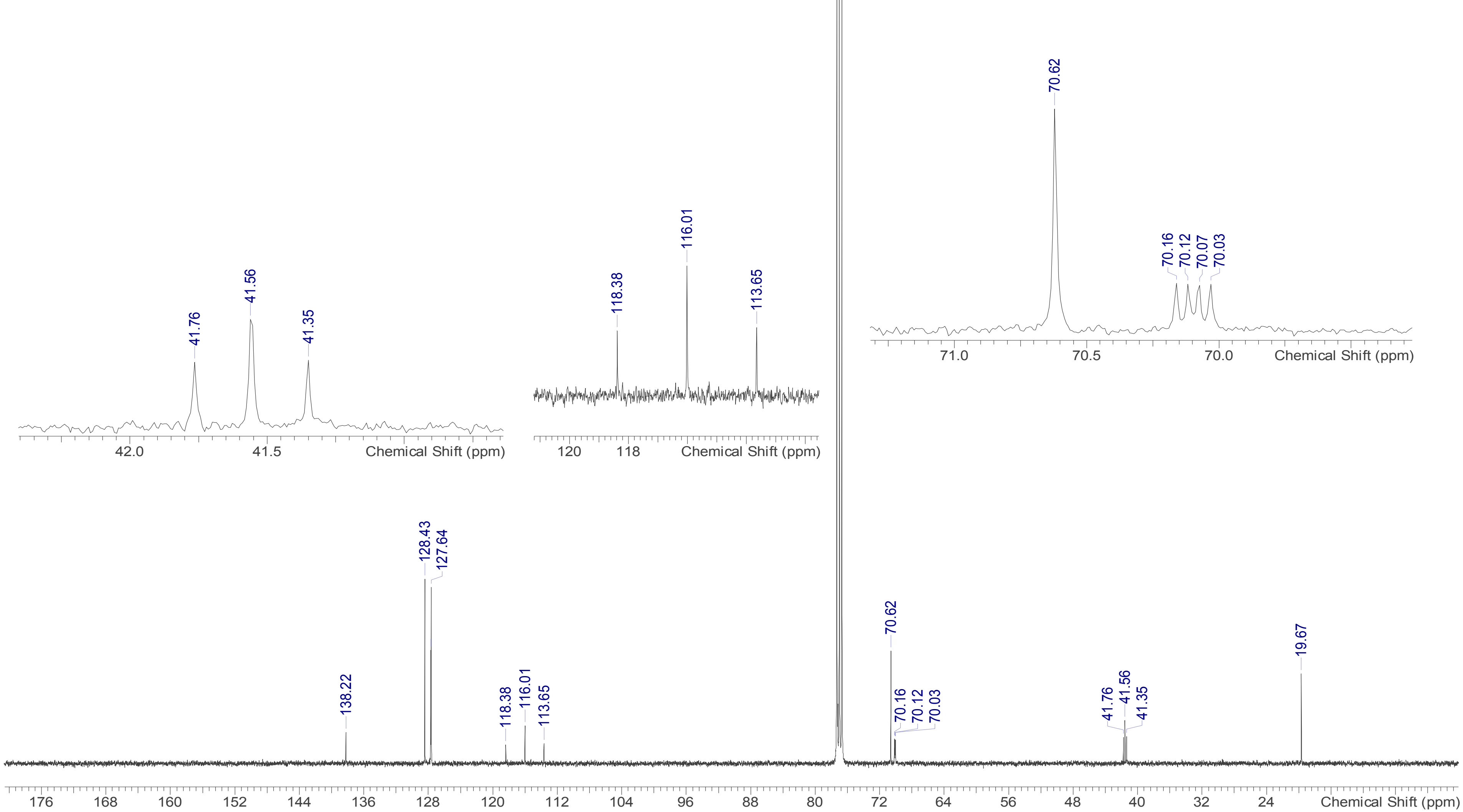

$^{19}F\{^1H\}$, $CDCl_3$, 376 MHz

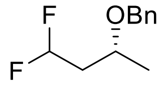

(±)-SI-16

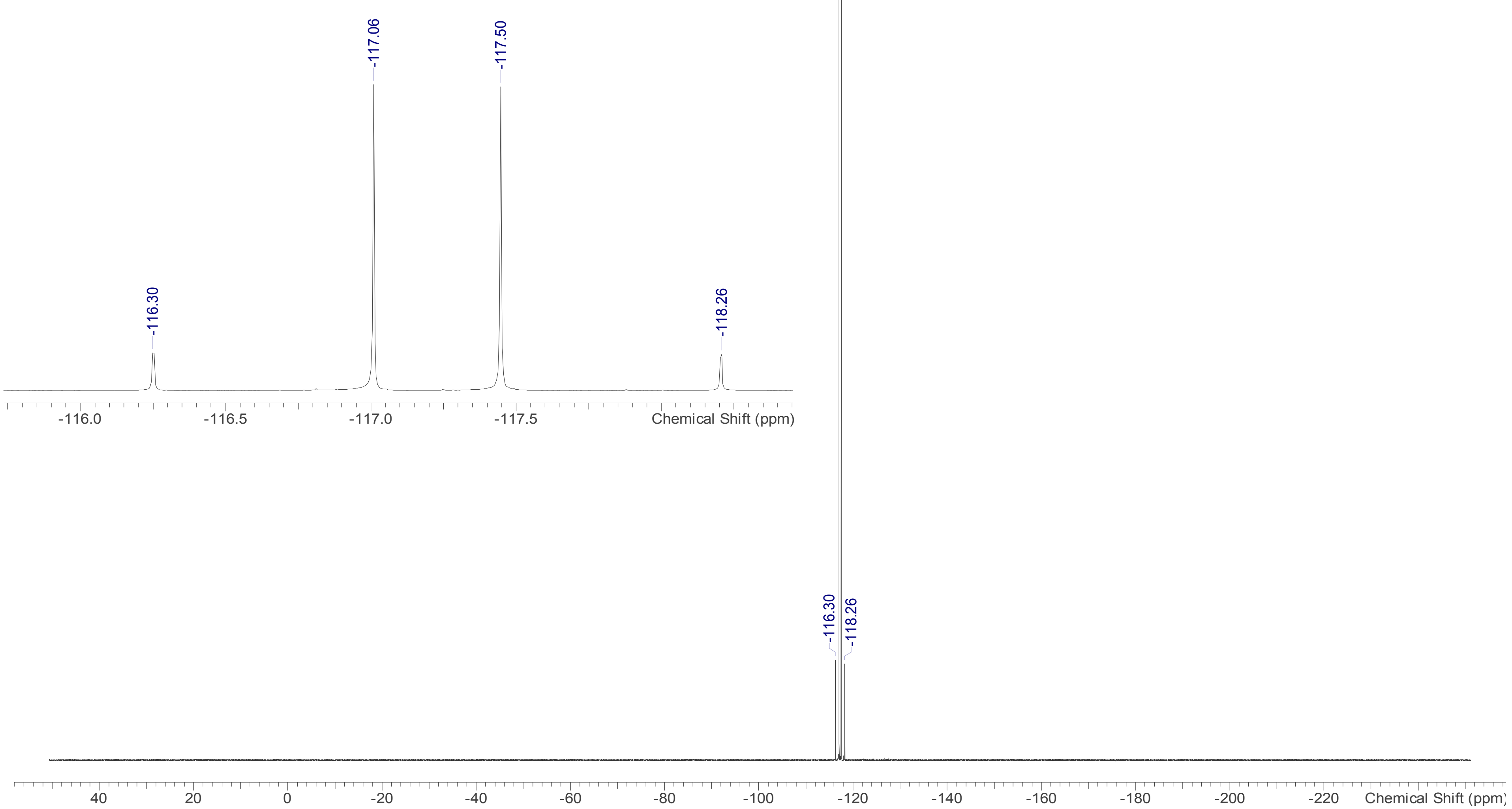

^{19}F, $CDCl_3$, 376 MHz

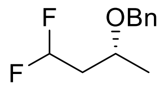

(±)-SI-16

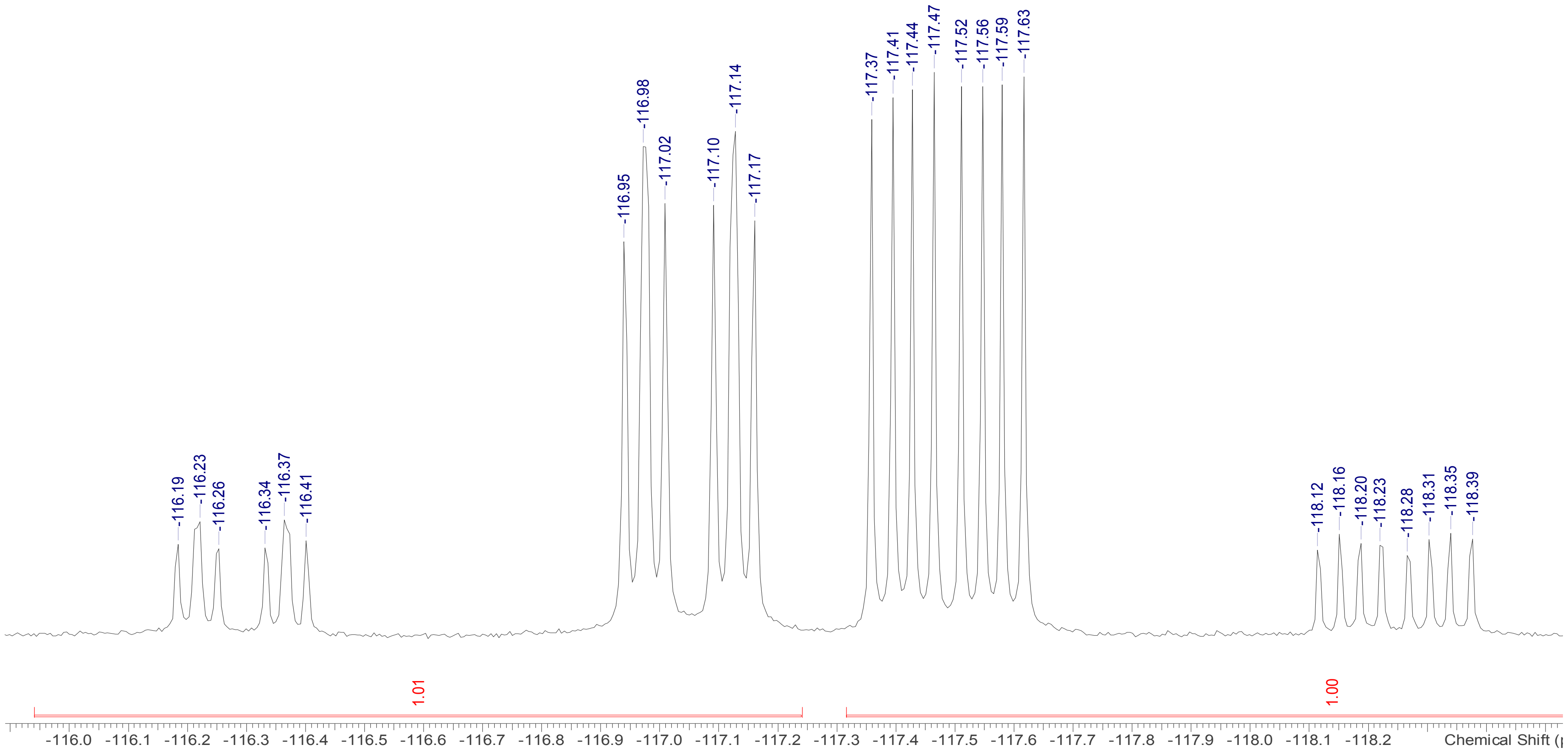

7.5.3 (±)-4,4-Difluorobutan-2-ol (±)-F

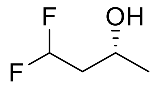

(±)-F

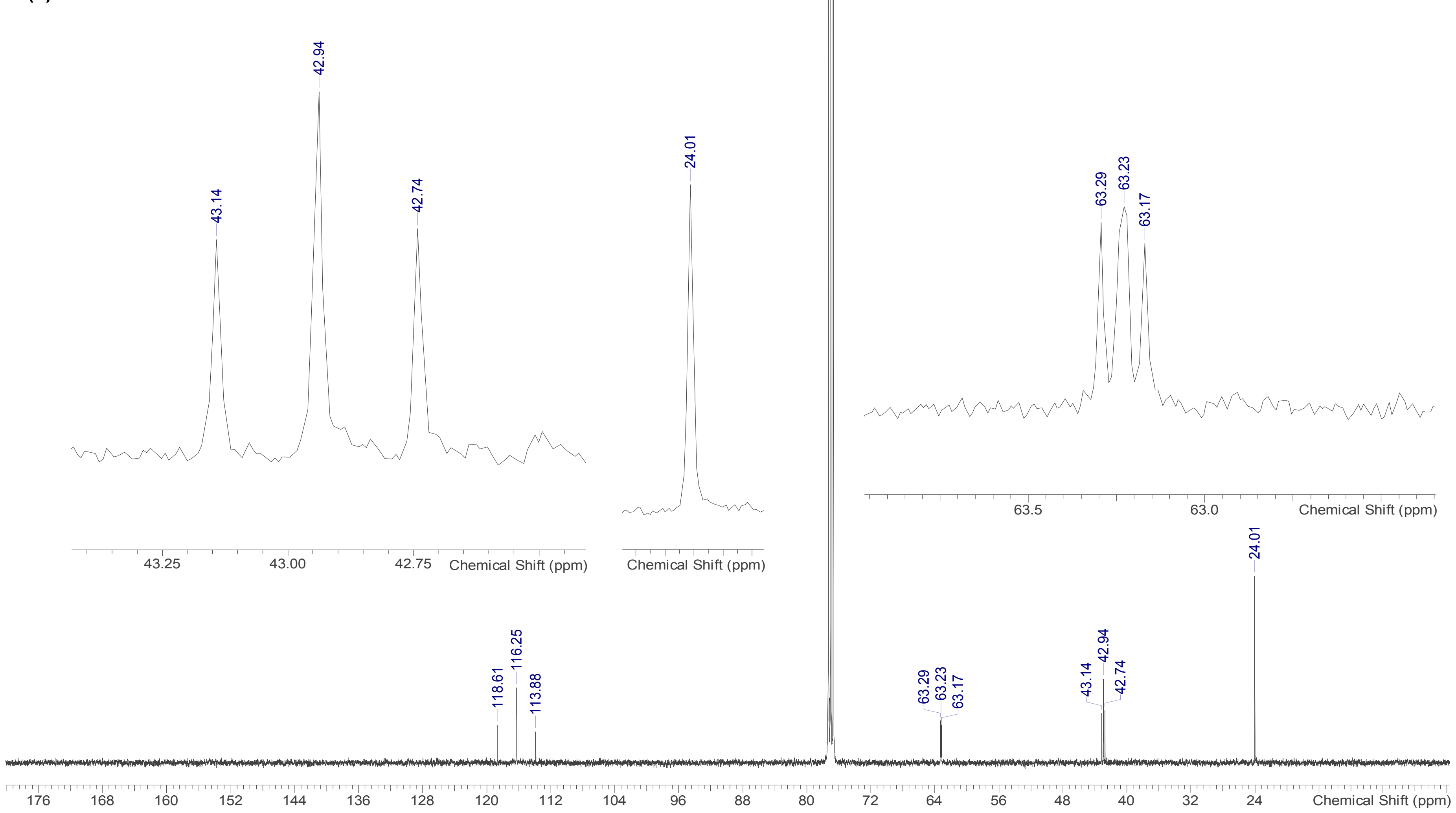

7.6 Synthesis of 3,3-difluoropropan-1-ol G

7.6.1 1-Benzyloxy-3,3-difluoropropane SI-17

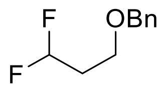

SI-17

^{1}H NMR, $CDCl_3$, 400 MHz

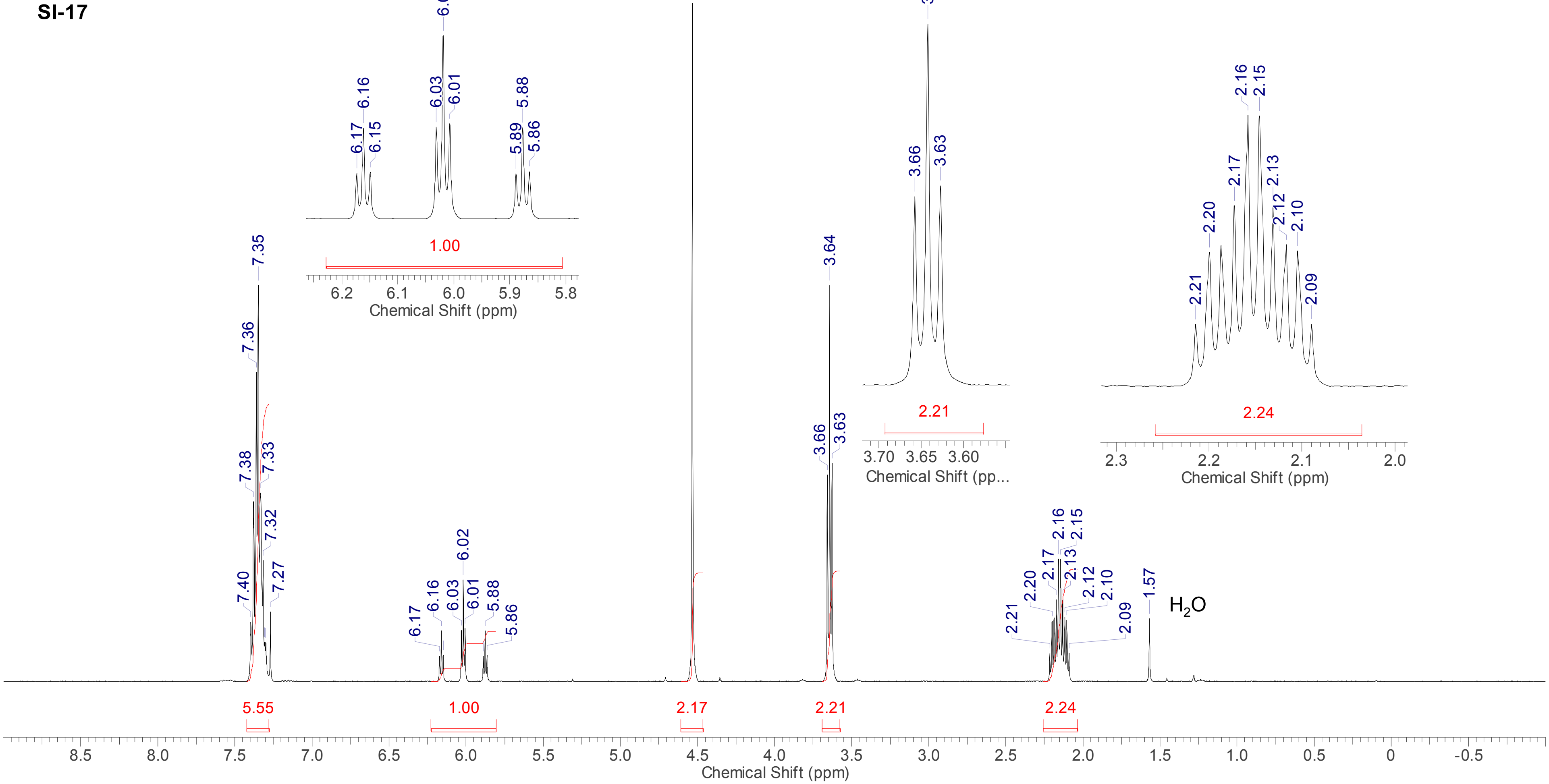

^{13}C NMR, $CDCl_3$, 101 MHz

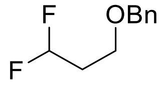

SI-17

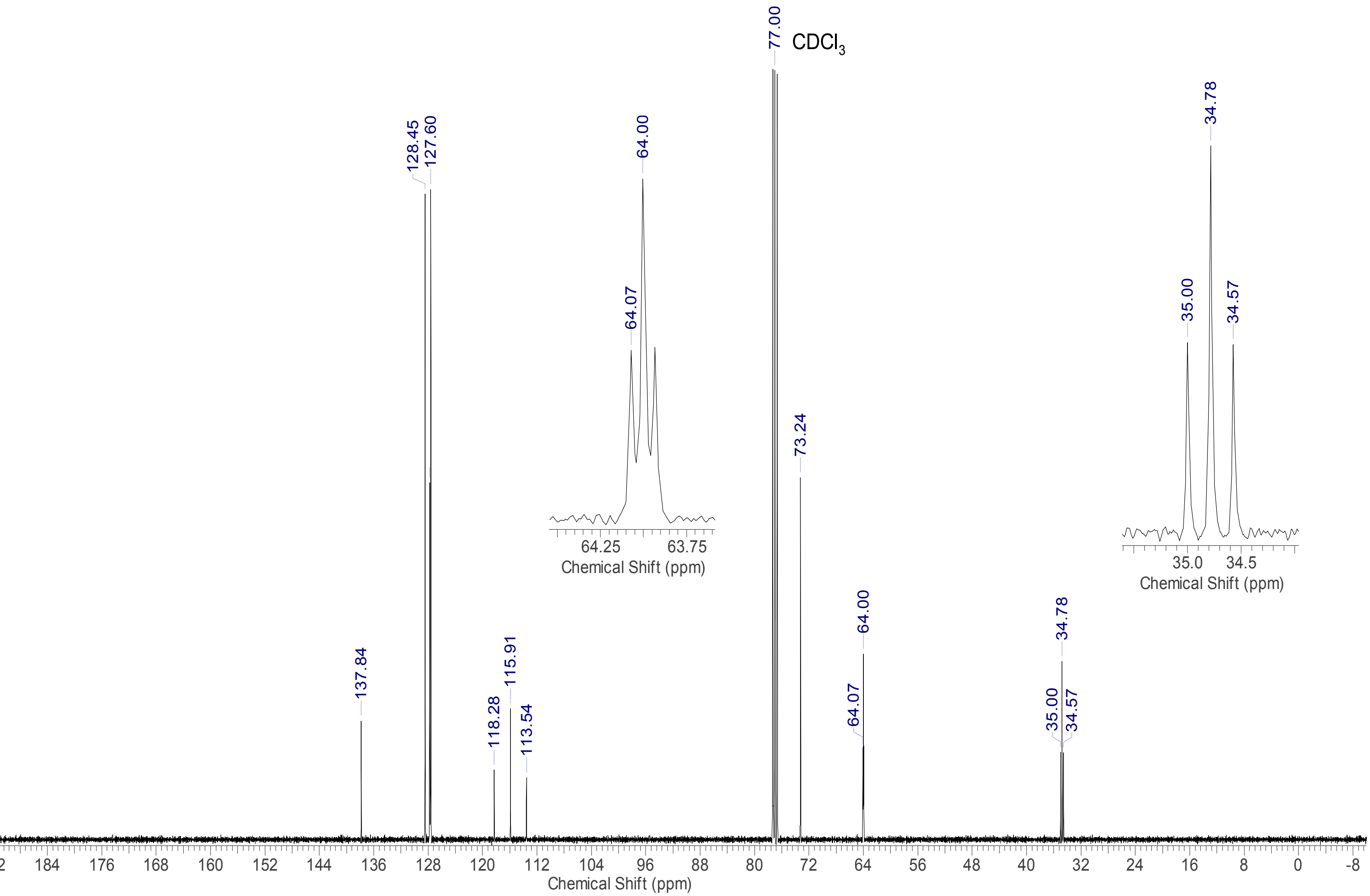

^{19}F, $CDCl_3$, 376 MHz

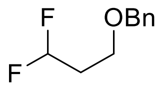

SI-17

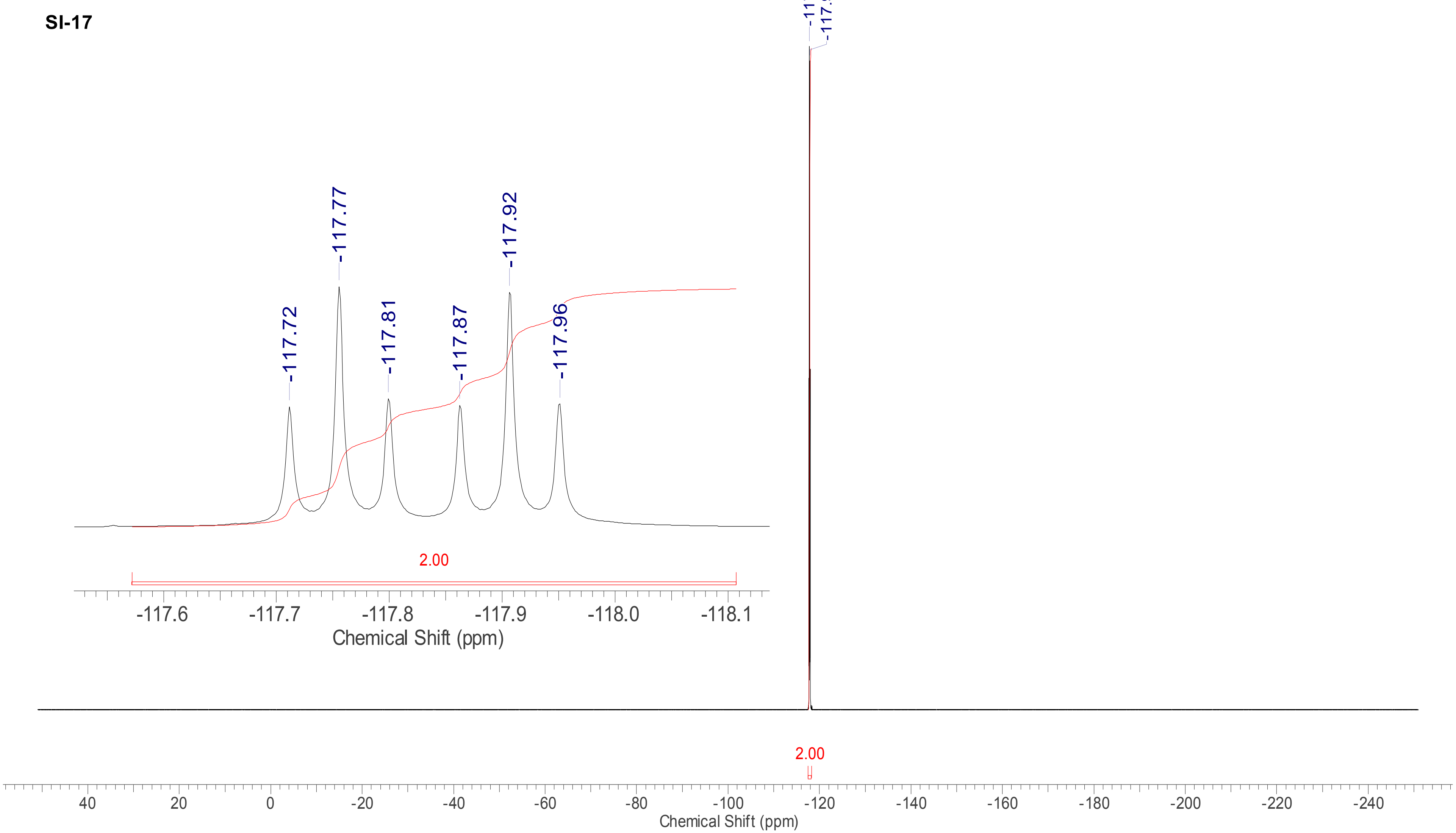

7.6.2 3,3-Difluoropropan-1-ol G

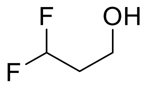
G

^{13}C NMR, $CDCl_3$, 126 MHz

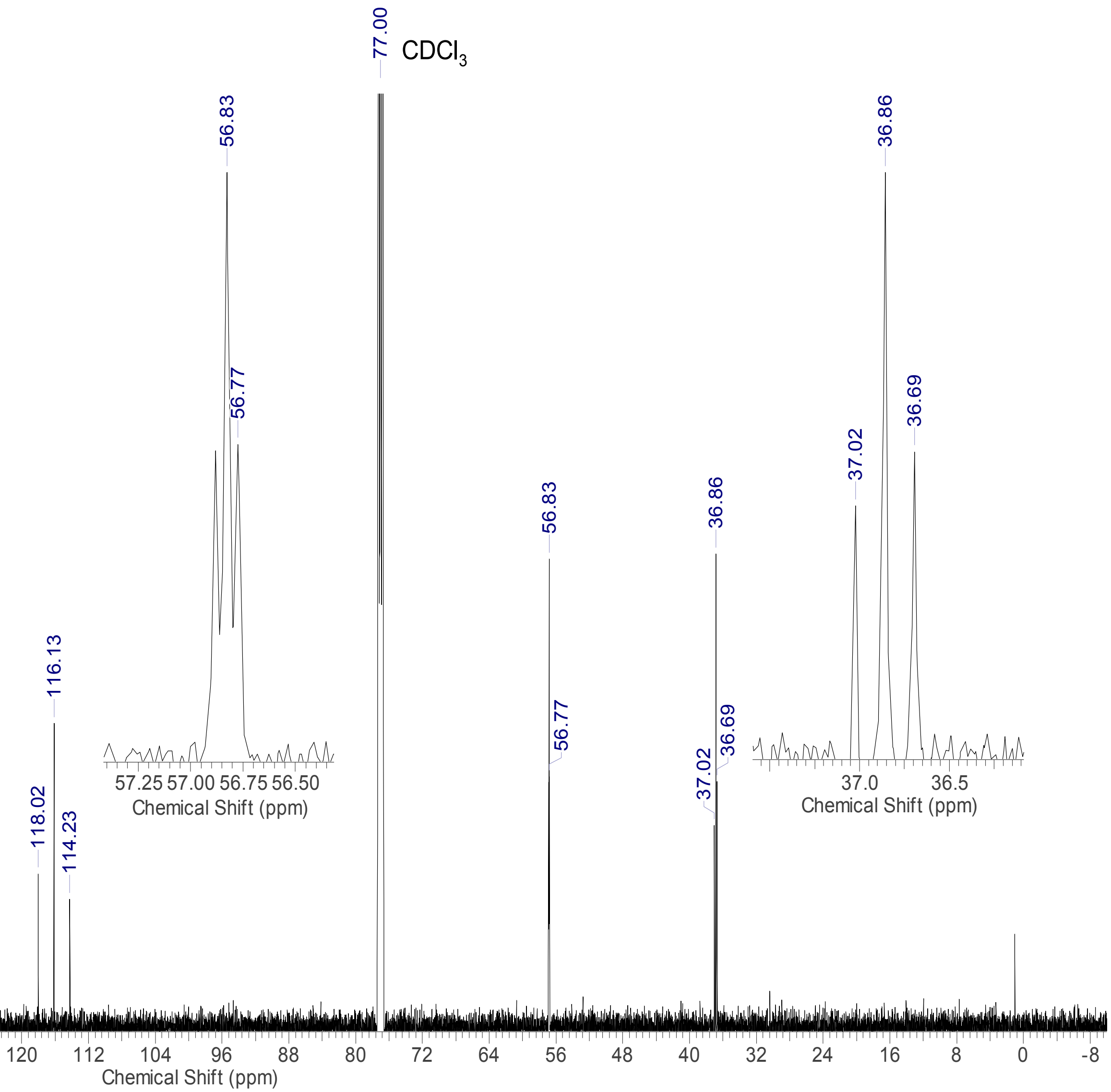

Supplement: Supplementary file 1 [file chem0021-17808-sd1.pdf]
